# Highly enantioselective catalytic synthesis of chiral pyridines

**DOI:** 10.1038/s41467-017-01966-7

**Published:** 2017-12-12

**Authors:** Ravindra P. Jumde, Francesco Lanza, Tilde Pellegrini, Syuzanna R. Harutyunyan

**Affiliations:** Stratingh Institute for Chemistry, University of Groningen, Nijenborgh 4, 9747 AG Groningen, The Netherlands

## Abstract

General methods to prepare chiral pyridine derivatives are greatly sought after due to their significance in medicinal chemistry. Here, we report highly enantioselective catalytic transformations of poorly reactive β-substituted alkenyl pyridines to access a wide range of alkylated chiral pyridines. The simple methodology involves reactivity enhancement via Lewis acid (LA) activation, the use of readily available and highly reactive Grignard reagents, and a copper-chiral diphosphine ligand catalyst. Apart from allowing the introduction of different linear, branched, cyclic, and functionalised alkyl chains at the β-position of alkenyl pyridines, the catalytic system also shows high functional group tolerance.

## Abstract (web summary)

Chiral pyridines are valuable building blocks in medicinal chemistry applications. Here, the authors report the copper-catalysed Lewis acid-assisted asymmetric alkylation of β-substituted alkenyl pyridines with Grignard reagents affording chiral pyridines with excellent enantioselectivity.

## Introduction

**Fig. 1:**
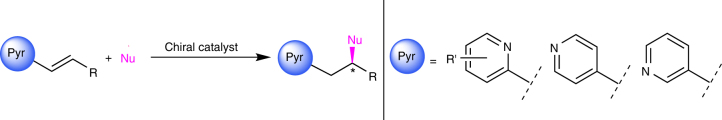
Enantioselective nucleophilic addition to alkenyl pyridines

Pyridines are among the most important classes of heterocyclic moieties, and occur in many bioactive molecules, such as natural products, pharmaceuticals, and agrochemicals^1–4^. Furthermore, pyridines are also useful structural motifs in various chemical transformations and they are widely employed as ligands^5–10^. Moreover, pyridine is the single most commonly found nitrogen-containing aromatic heterocycle among all U.S. FDA approved pharmaceuticals^11, 12^ and is present in more than 60 currently marketed drugs. As a result of their immense importance, methods to access different pyridine derivatives have been pursued for decades^13^. Although elaboration of the pyridine ring remains one of the most common and straightforward approaches, exploitation of the pyridine moiety to activate adjacent olefins towards enantioselective conjugate addition (CA) of nucleophiles has recently gained prominence (Fig. 1)^14, 15^.

Highly enantioselective copper catalysed CA of hydride nucleophiles to β,β-disubstituted alkenyl pyridines was reported by Lam and co-workers^16^. However, when carbon nucleophiles are considered, non-enantioselective CAs of carbon nucleophiles to unsubstituted vinylpyridines are well known^17^, whereas examples of general methodologies for enantioselective additions to β-substituted alkenyl pyridines are rare^18, 19^. The diminished reactivity of these β-substituted alkenyl pyridines that lies at the heart of this deficiency is a persistent issue, because of the low activation of the conjugated alkene by the pyridine ring^16^. Consequently, only a few examples of enantioselective functionalisation of an alkenyl pyridine have been reported, and these all require the use of activated pyridines, higher temperatures, and a Rh-catalyst^18, 19^. Moreover, these methodologies focus on arylation and do not allow alkylation. As the poor electrophilicity of alkenyl pyridines is one of the major problems for nucleophilic additions, we anticipated that the use of highly reactive Grignard reagents might be helpful to overcome the low reactivity of these substrates. This idea was further supported by the nickel-catalysed CA of aryl Grignard reagents to alkenyl pyridine reported in 1998, providing good yields although nearly racemic products^20^.

Here we present a Cu-catalysed, highly enantio- and chemo-selective alkylation protocol of poorly reactive alkenyl pyridines using Lewis acid (LA) activation and Grignard reagents as nucleophiles. Various pyridine derivatives can undergo CA of a wide range of Grignard reagents, both linear and branched. The catalytic system shows a remarkable functional group tolerance, providing a handle for further product transformations. Finally, mechanistic studies that allow to disclose the reaction pathway are presented as well.

## Results

### Optimisation of reaction conditions

We started out our investigations by evaluating copper catalysed conjugate alkylation of alkenyl pyridines with Grignard reagents. 4-Styryl pyridine **1a** and EtMgBr served as model reaction for the initial screening of suitable conditions (Table 1). Substrate **1a** was tested in the presence of a catalytic system derived from CuBr·SMe_2_ salt and diphosphine ferrocenyl ligand **L1** within the temperature range of −78 °C to ambient temperature. Analysis of the crude reaction mixture by ^1^H NMR revealed no conversion of the starting material, confirming the markedly lower reactivity of β-substituted alkenyl pyridines towards nucleophilic addition compared to vinylpyridines. Recently, our group developed a protocol that employs BF_3_⋅Et_2_O as a LA to activate poorly reactive heteroaromatic compounds towards nucleophilic addition of Grignard reagents^21^. We applied a similar protocol to 4-styryl pyridine **1a**, but, once again, no product was detected (Table 1, entry 1). We envisioned that stronger activation of pyridine substrates could be achieved by using TMSOTf as LA to tackle the reactivity issue.

**Table 1:** Optimisation of reaction conditions


Entry	Solvent	**L**, mol%^a^	Lewis acid, equiv.^b^	Conv. (%)^c^	ee (%)^d^
1	DCM	**L1**, 6	BF_3_⋅Et_2_O, 1.1	0	—
2	DCM	**L1**, 6	B[PhF_5_]_3,_ 1.1	0	—
3	DCM	**L1**, 6	TMSOTf, 1.5	89	89
4	DCM	**L1**, 6	TMSOTf, 2.0	85	85
5	DCM	**L1**, 6	TMSOTf, 3.0	91	89
6	DCM	**L1**, 12	TMSOTf, 1.5	91	92
7	DCM	**L1**, 12	TMSOTf, 2.0	95	93
8	DCM	**L1**, 12	TMSOTf, 3.0	100	93
9	Et_2_O	**L1**, 12	TMSOTf, 3.0	62	95
10	MTBE	**L1**, 12	TMSOTf, 3.0	67	96
11	Toluene	**L1**, 12	TMSOTf, 3.0	79	87
12	DCM	**L2**, 12	TMSOTf, 3.0	76	45
13	DCM	**L3**, 12	TMSOTf, 3.0	93	81
14	DCM	**L4**, 12	TMSOTf, 3.0	95	90

^a^6 or 12 mol% of **L** was used in combination with 5 or 10 mol% of CuBr·SMe_2_ respectively

^b^Equivalents of EtMgBr used correspond to that of the Lewis acid

^c^Determined by ^1^H NMR spectroscopy

^d^Determined by chiral HPLC

Indeed, screening of various LAs revealed that stronger activation of alkenyl pyridine does allow Grignard addition (Table 1, entries 1–5), with TMSOTf the optimal choice, providing alkylated pyridine product **2a** with 89% conversion and 89% ee (Table 1, entry 3). Importantly, TMSOTf can promote the addition reaction in the absence of the copper catalyst as well. To reach full conversion in the catalytic reaction and to minimise the effect of the non-catalytic TMSOTf-promoted pathway on the final enantioselectivity of the reaction, we studied the effect of the stoichiometry of the copper catalyst, TMSOTf and Grignard reagents (Table 1, entries 4–8). Although the enantioselectivity remained largely unchanged, full conversion was reached only when 3 equiv. of EtMgBr and TMSOTf was used (Table 1, entry 5). On the other hand, carrying out the reaction with a two-fold increase in the Cu-catalyst loading, resulted in an increase of the enantioselectivity to 93% (Table 1, entries 6–8).

Having established the optimal LA and catalyst loading for the reaction, we investigated the effect of different solvents next. All solvents tested (DCM, Et_2_O, MTBE, and toluene) were well tolerated by the alkylation protocol, providing product **2a** with excellent ee’s (Table 1, entry 8 vs entries 9–11). On the other hand, full conversion towards the addition product was reached only in DCM, which prompted us to carry out chiral ligand optimisation in this solvent.

Among the different chiral ligands studied (for detailed ligand screening, see Supplementary Table 1) all the diphosphine ferrocenyl ligands (**L1–L4**) were able to promote the reaction to a different extent. Ligands **L1**, **L3** and **L4** showed superior reactivity, delivering the desired product in excellent conversion and ee (Table 1, entries 8, 13–14). As ligand **L1** performed slightly better than **L3** and **L4**, we adopted the following optimised conditions for further substrate scope study: CuBr·SMe_2_ (10 mol%), (*R,S*_*p*_)-**L1** (12 mol%), Grignard reagent (3 equiv.), TMSOTf (3 equiv.), in DCM solvent for 16 h at −78 °C.

### Pyridine substrate scope

For studying the substrate scope we chose the reaction between 4-alkenyl pyridines (**1a–1d**) and 2-alkenyl pyridines (**3a–3g**) with EtMgBr (Table 2). 4-Alkenyl pyridines **1a**–**1d** readily underwent the nucleophilic addition reaction, irrespective of the β-substituent on the double bond (Table 2, entries 1–4). Alkyl and aryl substituents, both electron rich and electron poor, at the β-position were well tolerated, affording the alkylated products **2a**–**2d** in excellent isolated yields and enantiopurities (Table 2, entries 1–4). When 3-octenyl pyridine, 2-styryl pyridine or 2-octenyl pyridine were subjected to our optimised reaction conditions, no conversion towards the addition product was noted (Table 2 scheme). Although the poor reactivity of 3-octenyl pyridine can be attributed to the lack of resonance activation of the adjacent olefin moiety upon coordination of the substrate to the LA, the lack of reactivity of the latter two is more surprising. Interestingly, introducing a *tert*-butyldimethylsilyl ether (OTBDS) group at the δ-position enhanced the reactivity sufficiently, thus providing corresponding product **4a** with high yield and enantioselectivity (Table 2, entry 5).

**Table 2:** Substrate scope

^a^Conditions **A**: TMSOTf (3 equiv.) and EtMgBr (3 equiv.); conditions **B**: BF_3_⋅Et_2_O (1.5 equiv) and EtMgBr (1.5 equiv.)

^b^Reported yields are for isolated products

^c^Determined by chiral HPLC

^d^The absolute configurations of products were determined on the basis of single-crystal X-ray diffraction analysis of compound **2a′** (see Supplementary Information)

^e^EtMgBr diluted in toluene and added dropwise over 2 h

^f^ee of corresponding alcohol **2c'** after deprotection

^g^5 mol% of catalyst used

^h^ee of the corresponding ether **10**

^i^Small amount of unreacted substrate (≤9%) is not separable from product by chromatography, yields are calculated by purity of product in the mixed fraction by ^1^H NMR

As expected, alkenyl pyridines with an electron-withdrawing group in the aromatic ring (Tables 2, **3b** and **3c**) required milder activation. In this case, the use of 1.5 equiv. of BF_3_⋅OEt_2_ instead of 3.0 equiv. of TMSOTf provided the corresponding products **4b** and **4c** with good yields and excellent enantioselectivities (Table 2, entries 6, 7).

**Table 3:** Grignard scope


Entry	R′MgX	R	Product	Yield (%)^a^	ee (%)^b^
1	EtMgBr	Ph	**2a**	94	93
2	PrMgCl	Ph	**5a**	75	93
3	HexMgBr	Ph	**5b**	81	95
4	*i*-PentMgBr	Ph	**5c**	65	97
5	*i*-BuMgBr	Hexyl	**5d**	56	64
6^c^	c-PentMgBr	Hexyl	**5e**	54	89
7	CH_2_=CH(CH_2_)_2_MgBr	Hexyl	**5f**	91	93
8	CH_2_=CH(CH_2_)_3_MgBr	Ph	**5g**	66	90
9	Ph(CH_2_)_2_MgBr	Hexyl	**5** **h**	89	97
10^d^	MeMgBr	Hexyl	**5i**	50	93
11^e^	PhMgBr	Hexyl	**5j**	84	0

^a^Reported yields are for isolated products

^b^Determined by chiral HPLC

^c^4. equiv. of Grignard was used

^d^Reaction was carried out at 0 °C for 5 h

^e^Ligand **L10** was used (see Supplementary Table 1 for **L10** structure)

We were particularly interested in performing these reactions with functionalised pyridines, which can provide a handle for further modification after Grignard addition. For this purpose, several alkenyl pyridine substrates with various functional groups were prepared (Tables 2, **3d**–**3g**) and subjected to the reaction protocol using BF_3_⋅OEt_2_.

Grignard reagents are often considered incompatible with reactive functional groups. Nevertheless, the excellent chemoselectivity of our protocol allowed us to perform addition reactions not only to alkenyl pyridines that contain halogens (Tables 2, **3f**, **3g**), but also to those containing highly reactive ester and cyano groups (Tables 2, **3d**, **3e**) in the aromatic ring. The corresponding alkylated products **4d**–**4g** were obtained with good to excellent yields and ee’s (Table 2, entries 8–11).

### Grignard reagent scope

To assess the nucleophile scope, substrates **1a** and **1d** were chosen as model compounds (Table 3). Addition of all tested linear Grignard reagents afforded the products **2a**, **5a** and **5b** with good to excellent yields (75–94%) and ee’s (93–95%), independent of the chain length (Table 3, entries 1–3). The sterically demanding α-, β-, and γ-branched Grignard reagents were also tolerated, providing products **5c**–**5e** with moderate to good yields (54–65%) (Table 3, entries 4–6). Particularly the γ- and α-branched Grignard reagents delivered products **5c** and **5e** with high ee’s of 97 and 89%, respectively (Table 3, entries 4 and 6), whereas the β-branched Grignard provided product **5d** with moderate ee (64%; Table 3, entry 5). Grignard reagents bearing olefinic or aromatic substituents were also well tolerated, affording the corresponding products **5** **f**, **5** **g** and **5** **h** with good yields and excellent ee’s (Table 3, entries 7–9).

Next, we investigated the reaction between 4-alkenyl pyridine and MeMgBr, the least reactive Grignard reagent, but the most interesting for pharmaceutical applications.

We were pleased to find that, thanks to the substantial activation provided by TMSOTf, the reaction proceeded with 87% conversion within 5 h at 0 °C. The product was isolated with excellent ee (93%) and a moderate yield of 50% (Table 3, entry 10).

On the other hand, addition of PhMgBr resulted in a complex reaction mixture. Changing from ligand **L1** to **L10** allowed to obtain the addition product **5j** with good yield, but unfortunately as a racemate (Table 3, entry 11). This result is in striking contrast with the 89% enantioselectivity obtained in our previous work for the addition of PhMgBr to alkenyl benzoxazole^21^ and could be attributed to an inability of the Cu-catalysed addition of PhMgBr to outcompete the LA promoted non-catalysed addition.

### Practical aspects and products functionalisation

**Fig. 2:**
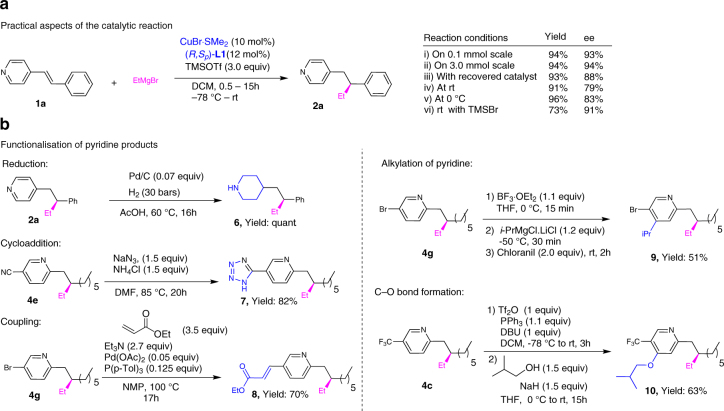
Practical aspects of CA to alkenyl pyridines and functionalisation of chiral pyridine products. **a** Scaling up, raising the reaction temperature, as well as use of recovered Cu-catalyst, are well tolerated by the present catalytic system. **b** The presence of a reactive substituent on the pyridine ring allowed the addition products to be further functionalised

To evaluate the robustness of our methodology, a series of experiments was carried out for the addition of EtMgBr to **1a** (Fig. 2a). Performing the reaction on a 30-fold larger scale (3 mmol vs 0.1 mmol) did not affect the outcome of the reaction and product **2a** was isolated with the same excellent yield (94%) and ee (94%) as for the 0.1 mmol reaction. Remarkably, the catalyst recovered (as a copper complex) from this reaction could be recycled with only minor deviations from the original results. Moreover, to assess the effect of temperature on the reaction and on the compatibility of the LA and the Grignard, the reaction was carried out at room temperature. To our surprise, the reaction proceeded to completion in 35 min and the product **2a** was isolated with 91% yield, although a significant drop in ee from 94 to 79% was noted. Assuming that the drop in enantioselectivity is due to the competing non-catalysed blank reaction, we performed the reaction at 0 °C, which improved the ee to 83%. Finally, we found that TMSBr is also an effective LA for this transformation, allowing the reaction to be carried out at room temperature and affording the final product with 73% yield and 91% ee (Fig. 2a).

Owing to the high functional group tolerance of our catalytic system, we were able to perform further synthetic elaborations of our chiral pyridine products (Fig. 2b). Piperidine derivative **6** was prepared quantitatively by selectively reducing the pyridine ring in **2a** in the presence of Pd catalyst and molecular hydrogen. The tetrazole ring is another heterocycle often encountered in the structures of approved pharmaceuticals^11^. To access tetrazole derivative **7** in good yield, we functionalised the cyano-substituted chiral pyridine **4e** using [3 + 2] cycloaddition with NaN_3_. Product **4** **g**, which has a bromo substituent on the pyridine ring, can be employed in several transformations.

Upon treatment of **4** **g** with ethyl acrylate in the presence of Pd catalyst, the corresponding Heck coupling product **8** was obtained with good yield. Moreover, using **4** **g** it is possible to achieve direct functionalisation of the pyridine ring (product **9**) using the protocol developed by Knochel and co-workers^22^. Another direct functionalisation of the pyridine ring of **4c**, leading to the formation of pyridyl-ether **10** through pyridyl-phosphonium salt, could be achieved using a very recent methodology developed by McNally and co-workers^23^.

### Mechanistic studies

The mechanism of this LA promoted Cu-catalysed addition of Grignard reagents to alkenyl pyridines might follow the same pathway as has been proposed for non-catalytic CA of organocuprates to α,β-unsaturated carbonyl compounds^24–26^. That implies formation of a reversible copper-alkene π-complex, followed by oxidative addition to form a Cu(III)-species, and reductive elimination to form the addition product enolate. Our catalytic system employs reagents and reactions conditions similar to those used for CAs to carbonyl based Michael acceptors^27, 28^, but contrary to these, alkenyl pyridines are unreactive towards organometallics in the absence of LA, even at non-cryogenic conditions. Furthermore, if the addition to alkenyl pyridines follows the same pathway, the aromaticity of the pyridines will be altered in several intermediate species. The necessity of LA to accomplish enantioselective CA to alkenyl pyridines adds another level of mechanistic complexity. To gain insight into the reaction mechanism, a series of experiments and NMR spectroscopic studies were carried out.

**Fig. 3:**
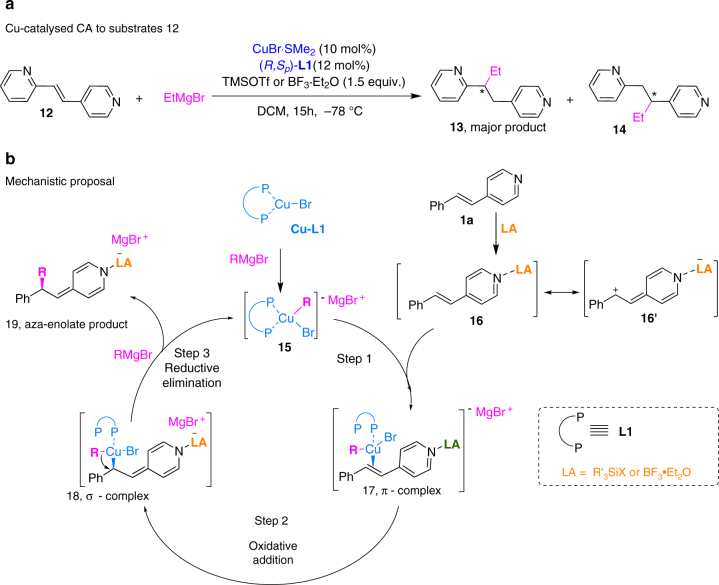
Cu-catalysed CA to substrates **12** and proposed catalytic cycle. **a** These experiments clarify the difference in reactivity between 4-substituted and 2-substituted pyridines. **b** Proposed tentative catalytic cycle for LA promoted Cu-catalysed CA of Grignard reagents to alkenyl pyridines **1a**

The experimental data discussed so far demonstrate that TMSOTf is the best LA for 4-substituted alkenyl pyridines, whereas BF_3_·Et_2_O is the most suitable LA for 2-substituted alkenyl pyridines. In addition, we found that no reaction occurs with non-activated 2-substituted alkenyl pyridines in the presence of either LA. Only after introducing an electron-withdrawing group (EWG) in the pyridine ring, CA occurs in the presence of BF_3_·Et_2_O (Table 2, entries 6–11). In contrast, using TMSOTf as LA, full conversion to side products (predominantly derived from the attack to the pyridine ring) occurs. On the other hand, non-activated 4-substituted alkenyl pyridine can only be converted to the CA product using TMSOTf (Table 2, entries 1, 2 and 4). This trend was further supported by the results obtained upon subjecting substrate **12**, which contains both a 4- and a 2-substituted pyridine ring, to the CA reaction (Fig. 3a). In the presence of TMSOTf the major product (**13**) corresponds to CA with respect to the 4-pyridine site, whereas in the presence of BF_3_·Et_2_O the products resulting from CA with respect to both the 4- and 2-pyridine sites (**13** and **14**, respectively) were observed in a 10:1 ratio. These results clearly indicate that 4-substituted pyridine is intrinsically more reactive towards CA than its 2-substituted analogue.

Further confirmation of the reactivity of pyridine substrates and the effect of LAs was obtained from ^1^H NMR spectroscopic studies (for details see Supplementary Methods). We investigated the interaction between various reagents (LAs and EtMgBr) and alkenyl pyridine substrates **1a** and **3b** at −60 °C (Supplementary Figs. 98 and 99). In all the mixed samples the signals corresponding to the pyridine are shifted with respect to the pure substrate sample. On the basis of the shifts of olefinic protons for substrate **1a** upon mixing with either TMSOTf, BF_3_·Et_2_O, TMSCl or EtMgBr (1–2 equiv.) we can rank these reagents in terms of their activating Lewis acidic strength as: ${\mathrm{TMSOTf}} >{\mathrm{BF}}_3\cdot {\mathrm{Et}}_2{\mathrm{O}} >{\mathrm{TMSCl}} > {\mathrm{EtMgBr}}$. Similar trends were observed for substrate **3b**.

To study the effect of the strength and bulkiness of LAs on the CA of EtMgBr to substrate **1a**, two sets of experiments were carried out, either in the presence or the absence of the chiral Cu-catalyst (Table 4). As disclosed above, in either case no conversion occurs when using BF_3_·Et_2_O as LA, whereas full conversion is observed for TMSOTf (Table 4, compare entries 1–4). The enantioselectivity of 93% obtained with TMSOTf is particularly remarkable, as the LA promoted background reaction in the absence of a copper catalyst is complete at −78 ^o^C in 4 h. Varying the counterion of the silicon based LA allowed a closer look at the LA strength and confirmed that with the very weak TMSCl and somewhat stronger TMSBr no conversion was observed after 15 h without catalyst (Table 4, entries 5 and 6). Remarkably though, in the presence of the Cu-catalyst full conversion to the CA product was observed with TMSBr (Table 4, entry 8) and 60% conversion with TMSCl (Table 4, entry 7) and in both cases only one enantiomer was found. These results were unexpected, especially for the rather weak TMSCl.

**Table 4:** Effect of different LAs in Cu(I)-catalysed addition of EtMgBr to **1a**


Entry	LA	*T* (°C)	Cu/**L1** (%)	Conv. (%)^a^	Yield (%)^b^	ee (%)^c^
1	BF_3_·OEt_2_	−78	10	0	NA	NA
2	BF_3_·OEt_2_	−78	—	0	NA	NA
3	TMSOTf	−78	10	100	94	93
4	TMSOTf	−78	—	100 (in 4 h)	ND	Rac
5	TMSCl	−78	—	0	NA	NA
6	TMSBr	−78	—	0	NA	NA
7	TMSCl	−78	10	60	47	>99.9
8	TMSBr	−78	10	100	73	>99.9
9	TESOTf	−78	10	95	85	88
10	TBSOTf	−78	10	87	75	77
11	TBDPSOTf	−78	10	96	47	62
12	TBDPSOTf	−78	—	40	ND	Rac
13	TBSCl, TBDPSCl, TBDMSCl	(−78)–0	10–25	0	NA	NA
14	TESCl	0	10	40	ND	82
15	TESCl	0	—	0	NA	NA
16	TMSCl	0	10	91	ND	91

^a^Conversion was determined by ^1^H NMR

^b^Isolated yields

^c^Determined by chiral HPLC

The effect of the bulkiness of the LA was investigated by testing a number of different steric variations of silyl triflates (Table 4, entries 9–11). To our surprise, an increase of steric bulk at the silicon atom resulted in decreased enantioselectivity: when moving from TMS- to triethylsilyl (TES-), *tert*-butyldimethylsilyl (TBS-) and finally to *tert*-butyldiphenylsilyl (TBDPS) substituted triflates, the ee dropped from 93 to 62% (Table 4, entries 3, 9–11). However, from these experiments it is not clear whether these differences in enantioselectivity are caused by steric bulk alone. The 40% conversion observed with TBDPSOTf without catalyst (Table 4, entry 12) shows that the background reaction is relevant, and thus that differences in the relative rates of the racemic non-catalysed with respect to the enantioselective Cu-catalysed CA reactions might also affect the enantioselectivity. Interestingly, in contrast to the result with TMSCl, carrying out the Cu-catalysed reactions using TBS-, TBDMS- and TBDPS-chlorides at both −78 ^o^C and 0 ^o^C did not give any substrate conversion (Table 4, entry 13). However, after screening of various LAs and conditions we found a set of experiments that settles this matter (Table 4, entries 14–16). At 0 ^o^C the catalysed reaction with TMSCl gives an ee of 91% (Table 4, entry 16), whereas the same reaction with TESCl yields an ee of 82% (Table 4, entry 14). Importantly, without the catalyst no conversion is observed with TESCl, which unambiguously shows that the decreased enantioselectivity is not due to a background reaction and thus must be due to the larger bulk of the LA. Taken together, these results prove that the bulkiness of the LA affects both the enantioselectivity and the reactivity adversely and thus imply that the LAs are involved in both the stereo- and rate-determining step.

The geometry of alkenes is another important parameter, both for their reactivity and selectivity in addition reactions. Furthermore, when starting from less stable (*Z*)-alkenes, isomerisation to the (*E*)-stereoisomer might occur during the course of the reaction, thus affecting the overall reaction outcome while providing useful hints to understand the mechanism of the reaction^27^. Therefore, the influence of the alkene ((*Z*)/(*E*)) geometry on the enantioselective CA of EtMgBr to **1a** and **3b** in the presence of TMSOTf and BF_3_·Et_2_O, respectively, was studied (Supplementary Table 2). When subjecting (*Z*)-**1a** and (*Z*)-**3b** to the optimised reaction conditions, the enantioselectivities obtained in both reactions were lower than with (*E*)-**1a** and (*E*)-**3b**. Although the reduction in ee in the catalytic reaction could be related to the partial isomerisation of the corresponding (*Z*)-substrates’ double bond during the reaction, subsequent additional control experiments proved that the decreased enantioselectivity is intrinsic to the (*Z*)-geometry of the substrate and not due to (*Z*)–(*E*) isomerisation. This is at odds with our previous findings for Cu-catalysed CA to other *N*-containing alkenyl heteroarenes^21^, where we clearly observed isomerisation of alkenyl benzothiazole caused by all reaction components in combination, thus supporting Cu^I^/Cu^III^ redox chemistry with reversible formation of π- to σ-Cu-complexes responsible for substrate isomerisation^21^. The lack of Cu-mediated isomerisation in the present case does not rule out this mechanism, but might be suggestive of other possibilities.

Finally, we wanted to elucidate the structure of the initial product of these CA reactions before the reaction mixture is quenched. In particular, the question is whether a non-aromatic enol-like structure is formed and if so, whether it is a Mg-enolate or a LA-derived enolate (silyl enol ether for TMSOTf or boron enolate for BF_3_·OEt_2_). In the case of Cu-catalysed CA of EtMgBr to substrate **1a** in the presence of TMSOTf, we only observed the formation of silyl-enolate (Supplementary Fig. 104). In the case of CA to 2-alkenyl pyridine substrate **3b** using BF_3_·OEt_2_, our efforts to characterise the product structure before quenching of the reaction were hampered by severely broad signals observed in ^1^H NMR.

The information gathered during these investigations allows several inferences to be made regarding the mechanism of our catalytic system as well as a rationalisation of its behaviour.

The presence of LA is a necessary condition to accomplish enantioselective CA to alkenyl pyridines. The fact that LA promotes not only Cu-catalysed, but in several instances also non-catalytic background addition, makes it difficult to elucidate its role precisely. LA additives have been known to accelerate CA of organocuprates to various α,β-unsaturated carbonyl derivatives^24^. However, strong LAs, such as BF_3_·OEt_2_, were only used in combination with stoichiometric amounts of organocopper reagent (Yamamoto reagent) to avoid a compatibility problem commonly encountered between Grignard reagents and LAs^24, 29–31^. In contrast, for Cu-catalysis (as well as stoichiometric reactions) the use of TMSCl, which is more a silylating reagent than a LA, became common practice in CA^24, 32–37^. In our case, we use catalytic amounts of chiral copper complex in combination with strong LAs, which do not only activate the pyridine substrate but also interfere with the CA by reacting with the Grignard reagent or decomposing the chiral Cu-complex catalyst. As a result, the outcome of the reaction depends critically on the relative rates of the desired catalysed vs. the undesired background pathway, as well as on these competing reactions.

The proposed reaction pathway, based on experimental and spectroscopic data and taking into account the previous proposals on Cu-catalysed CA, is presented in Fig. 3. We expect that the first step in the catalytic cycle is initiated by the formation of the catalytically active complex **15** ^38^, formed from precatalyst **Cu-L1** upon transmetallation with Grignard reagent in a similar manner as has been proposed for Cu-catalysed CA to carbonyl based Michael acceptors employing similar solvent and diphosphine ligand^27, 28^. The first intermediate in the proposed cycle (**17**) is formed via π-complexation between activated alkenyl pyridine **16** and transmetallated copper complex **15**. This is then followed by the formation of a σ-complex intermediate, **18**. We anticipated the activated alkenyl pyridine to be an LA-pyridine complex, the formation of which was indeed observed by ^1^H NMR spectroscopy when using either BF_3_·OEt_2_, TMSOTf or TMSCl (Supplementary Figs. 98 and 99). Interestingly, although theoretically TMSCl is the weakest silicon based LA, especially in comparison with BF_3_·OEt_2_, it nevertheless is able to catalyse the reaction with non-activated substrate **1a**, in contrast to BF_3_·OEt_2_ (Table 4, compare entries 1 and 7). This finding indicates that the role of the LA is not limited to the initial Lewis acidic substrate activation. Rather, it is also involved in the acceleration of the reaction via silylation of the reaction intermediates, leading to the formation of the more stable, non-aromatic intermediate **18** and the silyl aza-enolate **19** (Fig. 3). The structure of **19** was confirmed by NMR spectroscopic experiments (Supplementary Fig. 104).

Another aspect of the critical function of LA in the catalytic cycle is evident from the observation that its bulkiness has an important role in defining the enantioselectivity (Table 4, entries 9–16). The decrease in enantioselectivity from 91 to 82% when moving from TMSCl to TESCl can only be attributed to the bulkiness of the LA and not to its capability of promoting non-catalysed CA, as there is no background TESCl promoted reaction in the absence of a Cu catalyst (Table 4, entries 4–16). The origin of this effect in our system is unclear, as well as which are the rate- and enantio-determining steps.

The mechanistic pathway for Cu-catalysed CA, as well the origin of the accelerations observed for reactions with TMSCl, have been the subject of considerable debate. However, so far the mechanism has been studied only for non-catalytic reactions with organocopper reagents. The current view is that the oxidative addition (π- to σ-complex) is reversible and the following reductive elimination is the rate-limiting step. This is supported by (*Z*)–(*E*) isomerisations observed in CA of organocopper reagents using (*Z*)-enones and (*Z*)-enoates, as well as by kinetic isotope effect studies. With respect to the role of TMSCl several hypotheses were raised, namely silylation of π-complex to form silyl enol ether of σ-complex, by Corey^32, 33^, LA activation of the enone substrate, by Kuwajima^35^, and stabilisation of the σ-complex by the chloride of TMSCl, by Snyder and Bertz^36^. Kinetic isotope experiments by Singleton and co-workers^37^ resolved this question for the systems studied, with the data consistent with rate-limiting silylation of an intermediate π-complex. This also explains the lack of (*Z*)–(*E*)-isomerisations observed in the presence of TMSCl, which is then due to the rate-limiting step occurring earlier than in the system without TMSCl.

Our experiments differ from these mechanistic studies in two aspects, namely the fact that we use a catalytic system and a more Lewis basic substrate. In this case any LA we use is involved in the catalytic cycle from the start of the reaction, through complexation with the pyridine substrate (Supplementary Figs. 98 and 99). In principle steps 1–3 (Fig. 3) all lead to the formation of a chiral intermediate, and the fact that the bulkiness of the LA influences the ee indicates its involvement in the stereo-determining step. Similarly, any of these steps can be rate-determining, and the lack of isomerisation caused by LA/Cu/RMgBr does not distinguish between the steps. On the other hand, if the reductive elimination (Fig. 3, step 3) is the rate-limiting step, acceleration by the LA could prevent accumulation of σ-complex **18**, thus quickly converting it to the final aza-enolate product **19** and preventing any Cu-mediated isomerisation to occur. Although steps 1 and 2 cannot be excluded, we currently believe that the reductive elimination is the rate- and stereo-determining step, facilitated by the presence of LA in the σ-complex **18** and leading to the formation of a stable aza-enolate **19**. It is likely that the bulkiness of the LA affects the stability of non-aromatic intermediates **18** and product enolate **19** with the smallest LA being the most stable and in this manner contributes in both the reactivity and enantioselection. If the reductive elimination is the rate-limiting step, the rate of the reaction should also be dependent on the rate of re-forming the active catalyst **15**. If this process occurs simultaneously with the reductive elimination process it will also be affected by the bulkiness of the LA in the intermediate **18** with the smallest LA allowing the fastest reaction.

## Discussion

We have demonstrated that less reactive alkenyl pyridines can be used as Michael acceptors for enantioselective nucleophilic addition of Grignard reagents. The process exhibits a high functional group tolerance, a broad substrate scope including 4-alkenyl and 2-alkenyl pyridines with various substituents both on the aromatic ring and the β-position, as well as a broad Grignard scope including linear, branched, and functionalised examples. Importantly, enantioselective methylation of conjugated alkenyl pyridines is possible by using the least reactive Grignard reagent (MeMgBr), which is generally considered very difficult. Finally, these reactions can be carried out in the most common solvents and on comparatively large scales, while cryogenic conditions can be avoided. Several of the obtained chiral pyridine products could be transformed straightforwardly into diverse products, due to the remarkable functional group tolerance exhibited by this catalytic system.

Using NMR-guided spectroscopic studies, as well as variation of reaction parameters, we were able to identify that Cu-catalysed addition to alkenyl pyridines proceeds through a CA mechanism leading to aza-enolate products. We established that LA is involved in the catalytic cycle from the start of the reaction, and that its strength and bulkiness affect both the reactivity and the enantioselectivity of the CA, respectively. Furthermore, studies on the olefin geometry showed that Cu-induced (*Z*)–(*E*)-isomerisation does not occur in this catalytic system.

All together, these results prove that the reaction follows the common pathway of Cu-catalysed CA of organometallics and that the bulkiness of LAs affects both the enantioselectivity and the reactivity adversely, thus implying that the LAs are involved in both the stereo- and rate-determining step of the reaction.

## Methods

### General procedure A addition to 4-alkenyl pyridines

In a heat dried Schlenk tube equipped with septum and magnetic stirring bar, CuBr·SMe_2_ (0.10 equiv), and ligand (*R*,*S*_*p*_)-**L1** (0.12 equiv) were dissolved in CH_2_Cl_2_ (1 mL/0.1 mmol of substrate) and stirred under nitrogen atmosphere for 15 min. The substrate (1.0 equiv) was added at once. After stirring for 5 min. at RT the reaction mixture was cooled to −78 °C and TMSOTf or BF_3_·OEt_2_ (1.5–3.0 equiv, see Supplementary Note 1) was added followed by RMgX (3.0 equiv). After stirring at −78 °C for 16 h, the reaction was quenched with MeOH (1 mL) followed by saturated aqueous solution of NH_4_Cl and warmed to RT. Reaction mixture was extracted with CH_2_Cl_2_ (3 × 10 mL). Combined organic phases were dried over MgSO_4_, filtered and solvents were evaporated on rotary evaporator. The oily crude was purified by flash chromatography on silica using mixture of pentane and EtOAc as eluent.

### General procedure B for the addition to 2-alkenyl pyridines

In a heat dried Schlenk tube equipped with septum and magnetic stirring bar, CuBr·SMe_2_ (0.10 equiv), and ligand (*R*,*S*_*p*_)-**L1** (0.12 equiv) were dissolved in Et_2_O or CH_2_Cl_2_ (1 mL/0.1 mmol of substrate) and stirred under nitrogen atmosphere for 15 min. The substrate (1.0 equiv) was added at once. After stirring for 5 min. at RT the reaction mixture was cooled to −78 °C and BF_3_·OEt_2_ (1.5 equiv) was added followed by RMgX (1.5 equiv.). After stirring at −78 °C for 16 h, the reaction was quenched with MeOH (1 mL) followed by saturated aqueous solution of NH_4_Cl and warmed to RT. Reaction mixture was extracted with CH_2_Cl_2_ (3 × 10 mL). Combined organic phases were dried over MgSO_4_, filtered and solvents were evaporated on rotary evaporator. The oily crude was purified by flash chromatography on silica using mixture of pentane and EtOAc as eluent.

### General procedure C for the synthesis of racemic products

In a heat dried Schlenk tube equipped with septum and magnetic stirring bar, CuBr·SMe_2_ (0.10 equiv), and (±)-BINAP (0.12 equiv) were dissolved in CH_2_Cl_2_ (1 mL/0.1 mmol of substrate) and stirred under nitrogen atmosphere for 15 min. The substrate (1.0 equiv) was added at once. After stirring for 5 min. at RT the reaction mixture was cooled to −78 °C and TMSOTf (3.0 equiv) or BF_3_·OEt_2_ (1.5 equiv) was added followed by RMgX (1.5–3.0 equiv). After stirring at −78 °C for 16 h, the reaction was quenched with MeOH (1 mL) followed by saturated aqueous solution of NH_4_Cl and warmed to RT. Reaction mixture was extracted with CH_2_Cl_2_ (3 × 10 mL). Combined organic phases were dried over MgSO_4_, filtered and solvents were evaporated on rotary evaporator. The oily crude was purified by flash chromatography on silica using mixture of pentane and EtOAc as eluent. Unless otherwise noted all products were isolated as pale-yellow oil; for liquid/oily substrates, CuBr·SMe_2_ and ligand (*R*,*S*_*p*_)-**L1** were dissolved in 0.7 mL of Et_2_O/CH_2_Cl_2_, whereas the remaining 0.3 mL of Et_2_O/CH_2_Cl_2_ was employed to transfer the substrate in the reaction Schlenk tube.

### Data availability

The authors declare that the data supporting the findings of this study are available within the article and its Supplementary Information files. For the experimental procedures, additional mechanistic studies, spectroscopic and physical data of compounds, see Supplementary Methods. For NMR and HPLC analysis of the compounds in this article, see Supplementary Figs. 1–156. For ligands screening and isomerisation studies see Supplementary Tables 1 and 2 respectively. The CCDC 1532252 (**2a′** prepared from **2a**, see Supplementary Note 2) contains the supplementary crystallographic data for this paper (Supplementary file). These data can be obtained free of charge from The Cambridge Crystallographic Data Centre via http://www.ccdc.cam.ac.uk/data_request/cif.

## Electronic supplementary material

Supplementary Information

Description of Additional Supplementary Files

Supplementary Data 1
