# Supplementary material for: Highly enantioselective catalytic synthesis of chiral pyridines
Source: Nat Commun. 2017 Dec 12;8:2058. doi: 10.1038/s41467-017-01966-7 (PMC5727103; doi:10.1038/s41467-017-01966-7)
Supplement: Supplementary file 1 — Supplementary Information [file 41467_2017_1966_MOESM1_ESM.pdf]

# Supplementary Figures

## NMR spectra

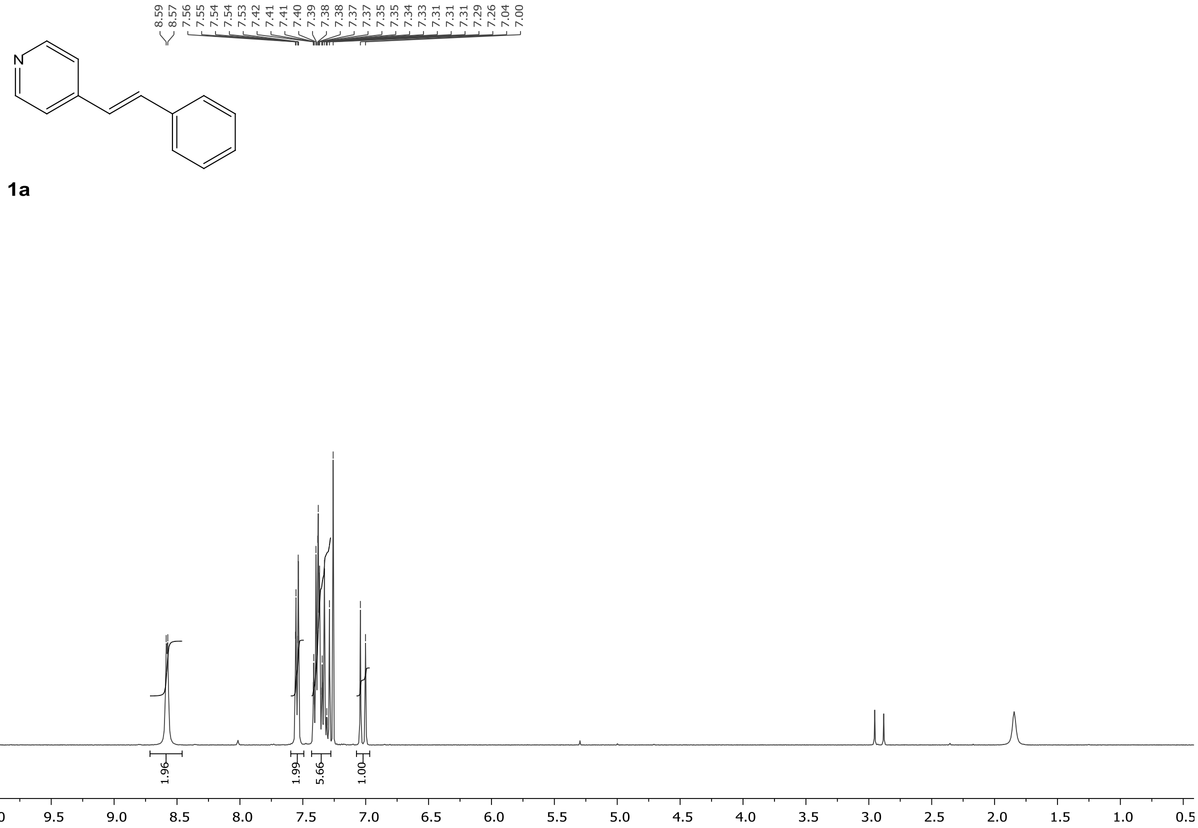


**Supplementary Figure 1** $^1$H NMR spectrum of **1a**

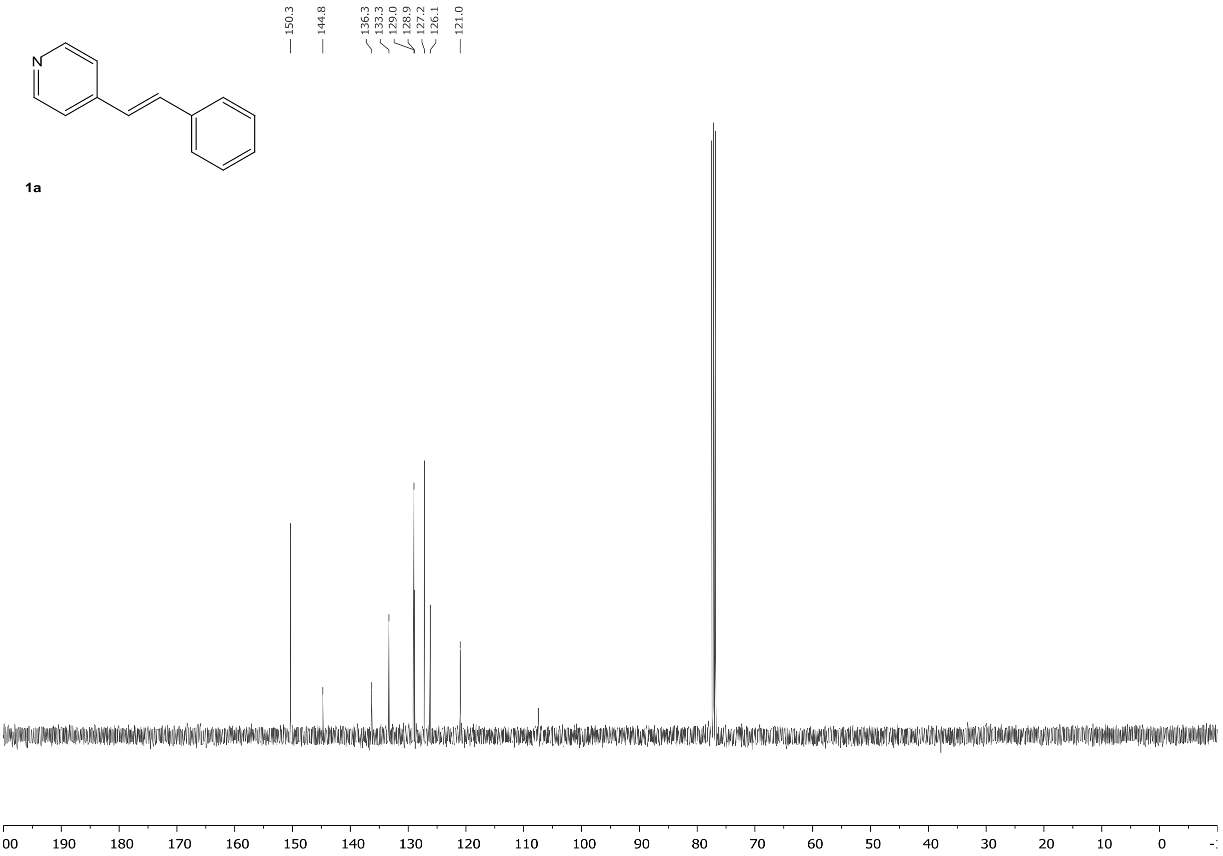


Supplementary Figure 2 $^{13}C$ NMR spectrum of **1a**

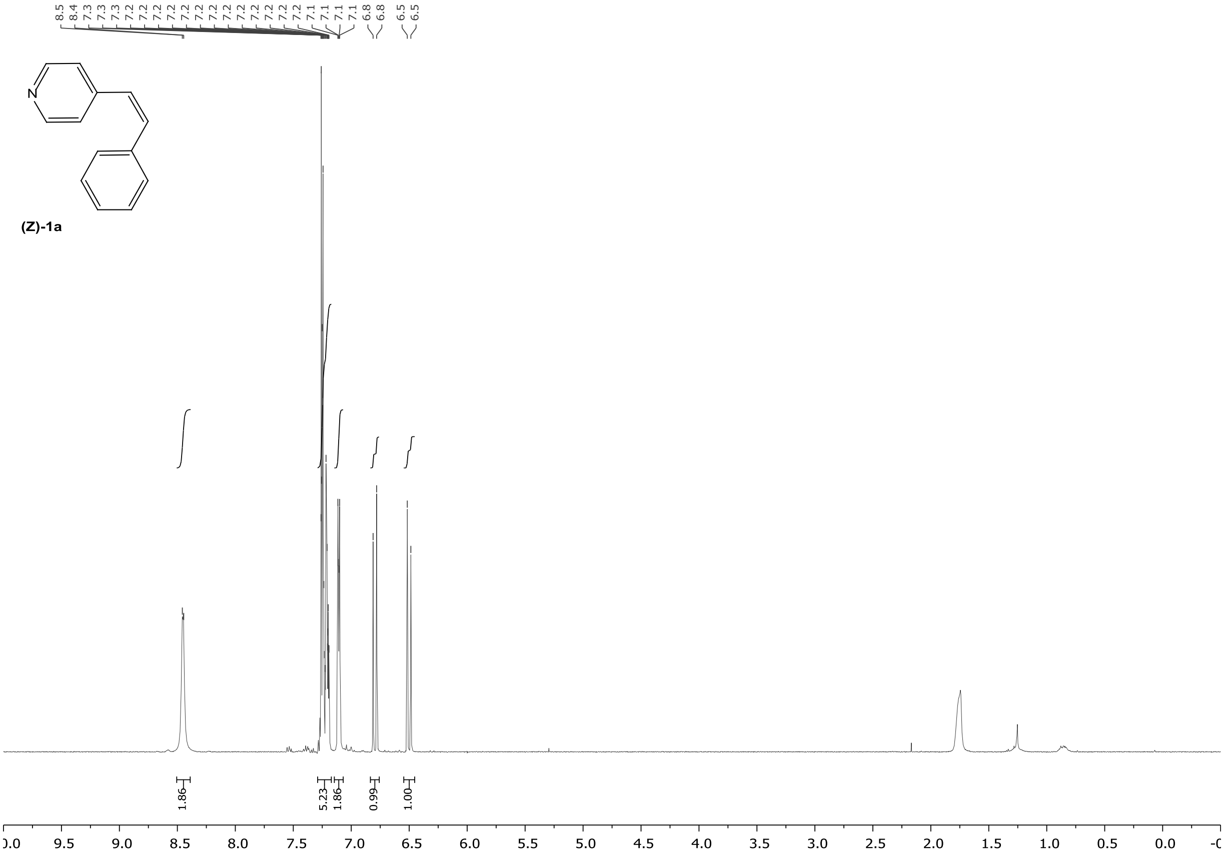


Supplementary Figure 3 $^{1}H$ NMR spectrum of ***(Z)*-1a**

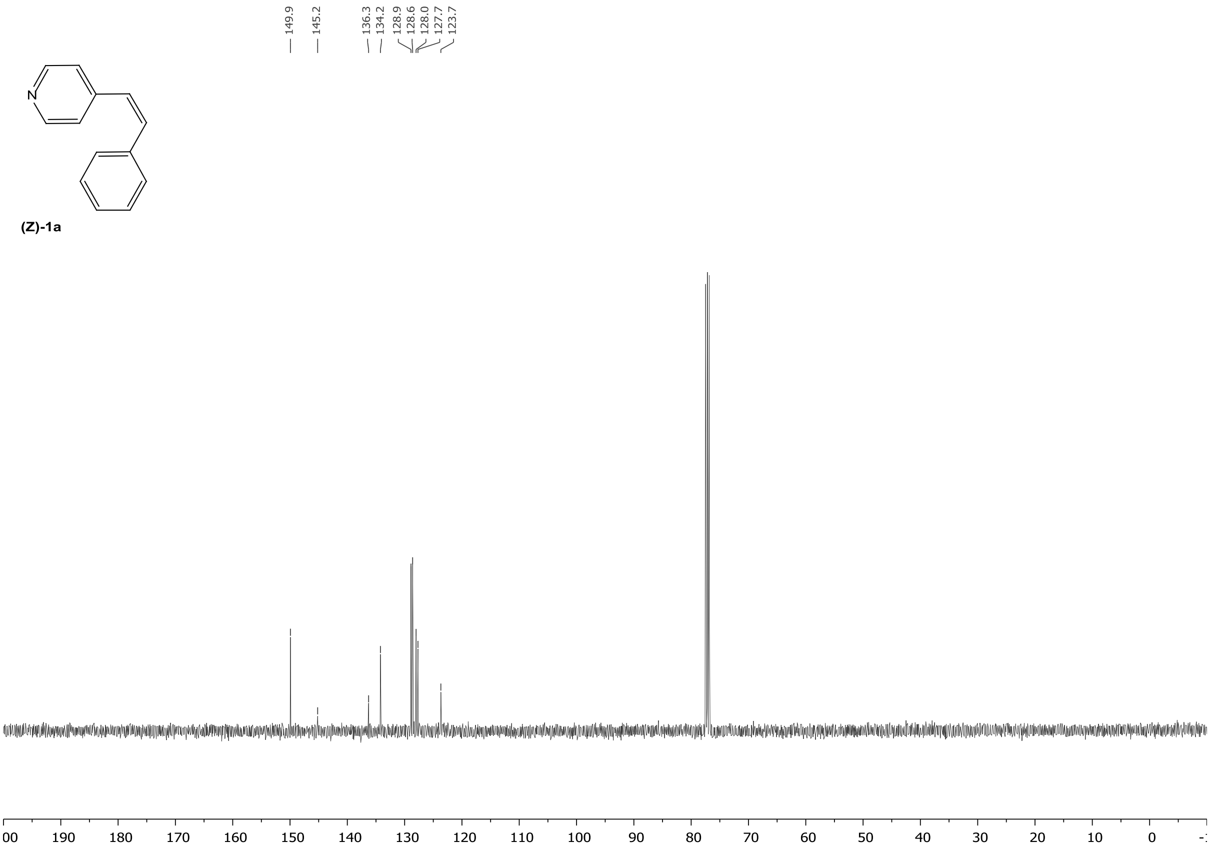


**Supplementary Figure 4** $^{13}$C NMR spectrum of **(*Z*)-1a**:

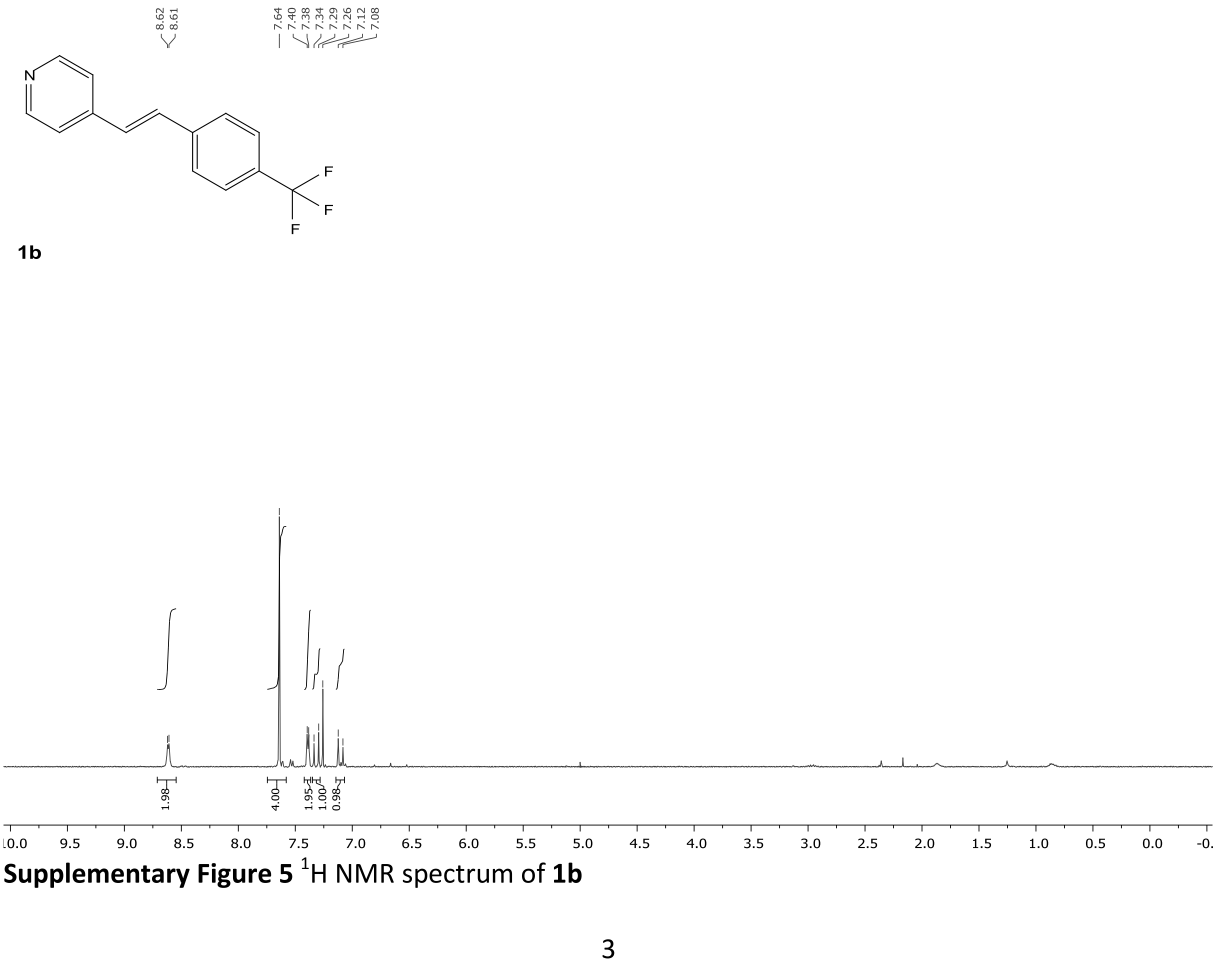


**Supplementary Figure 5** $^{1}$H NMR spectrum of **1b**

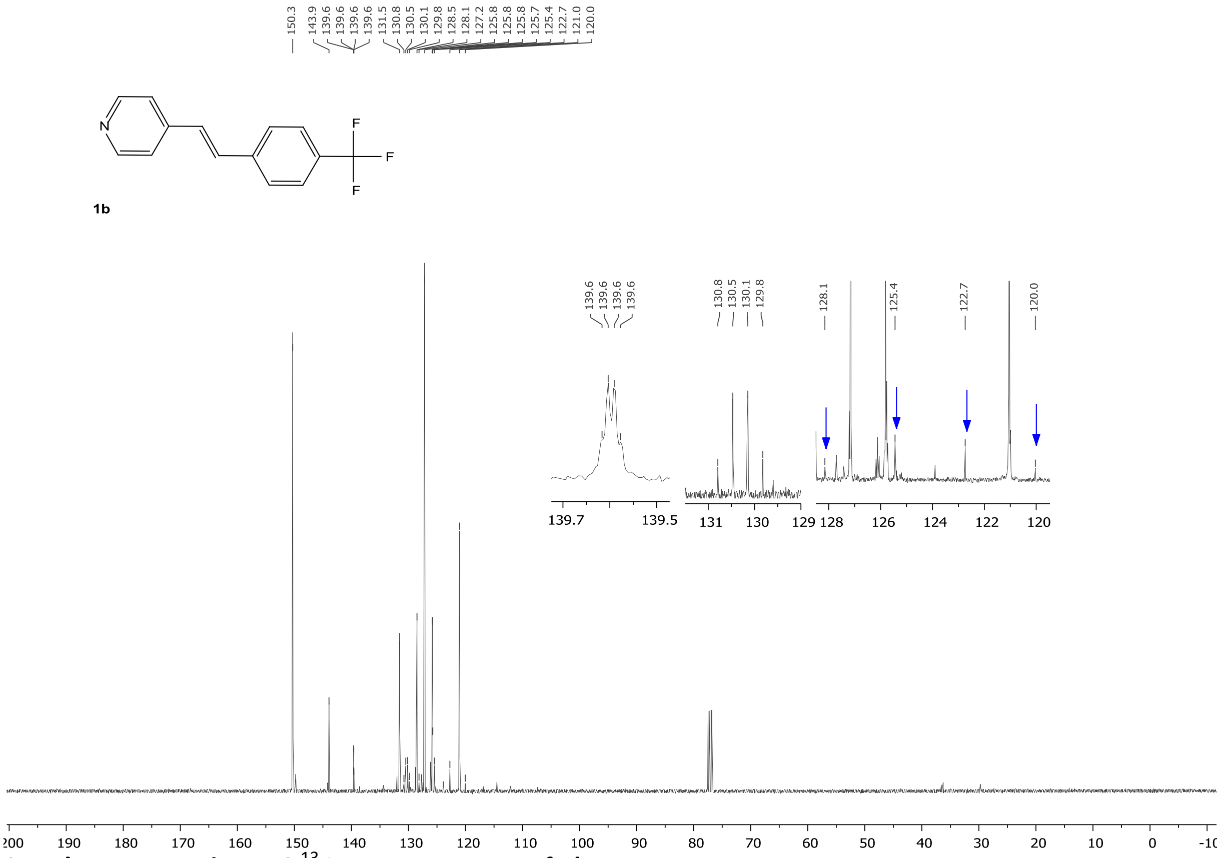

**Supplementary Figure 6** $^{13}$C NMR spectrum of **1b**

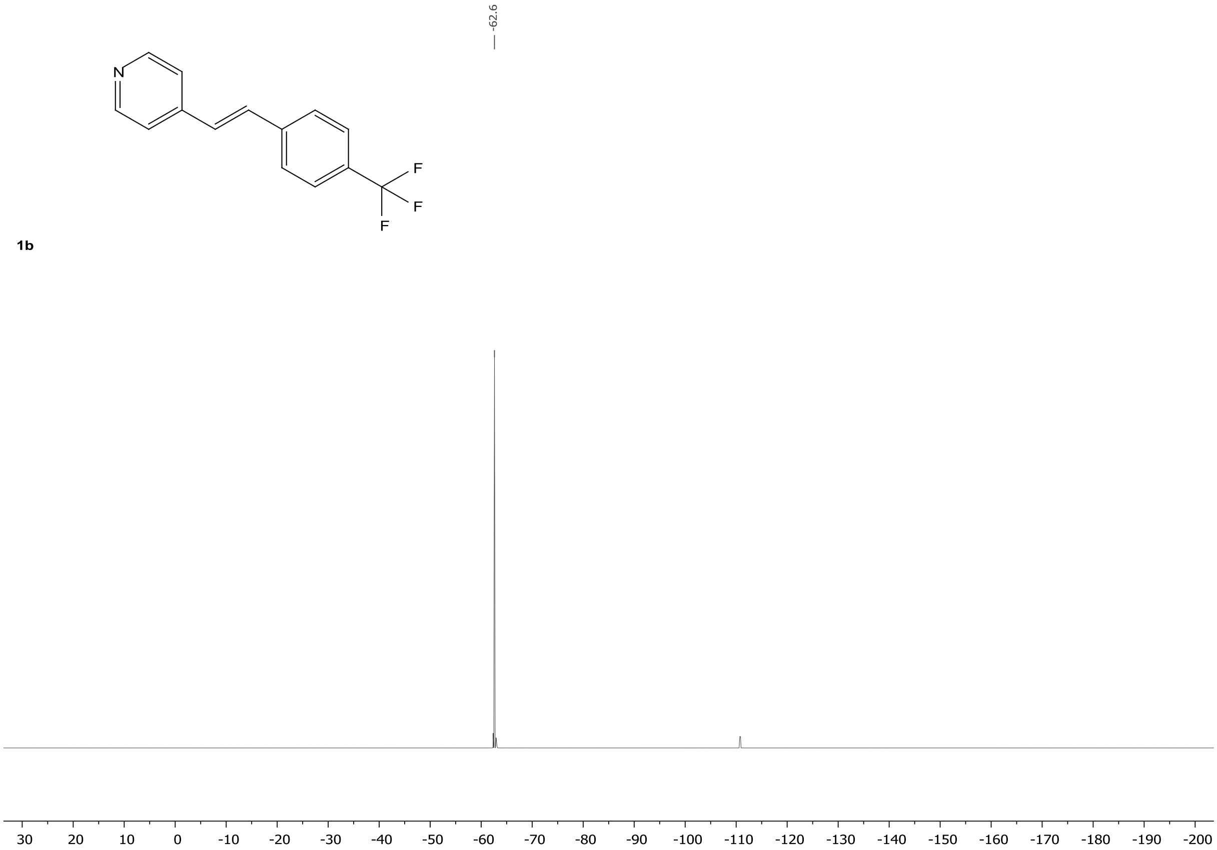

**Supplementary Figure 7** $^{19}$F NMR spectrum of **1b**

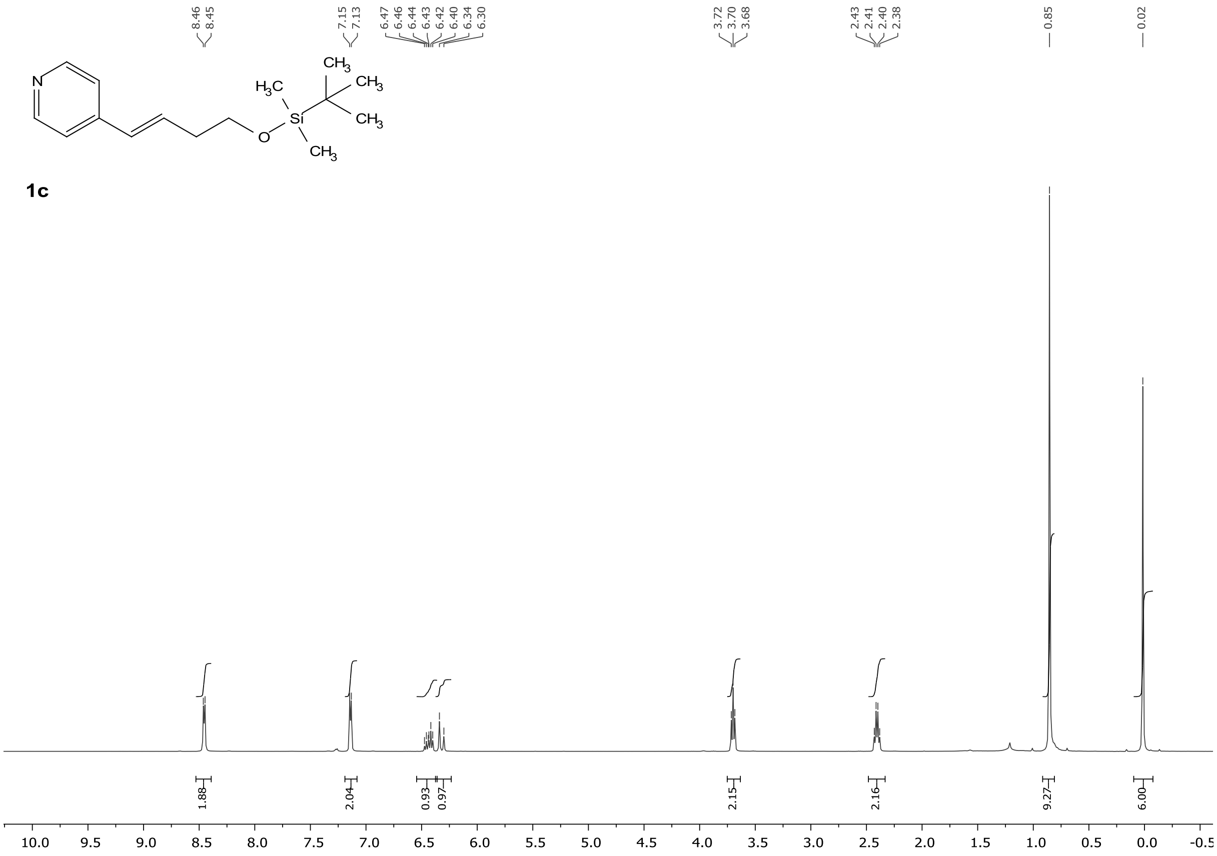


**Supplementary Figure 8** $^{1}$H NMR spectrum of **1c**

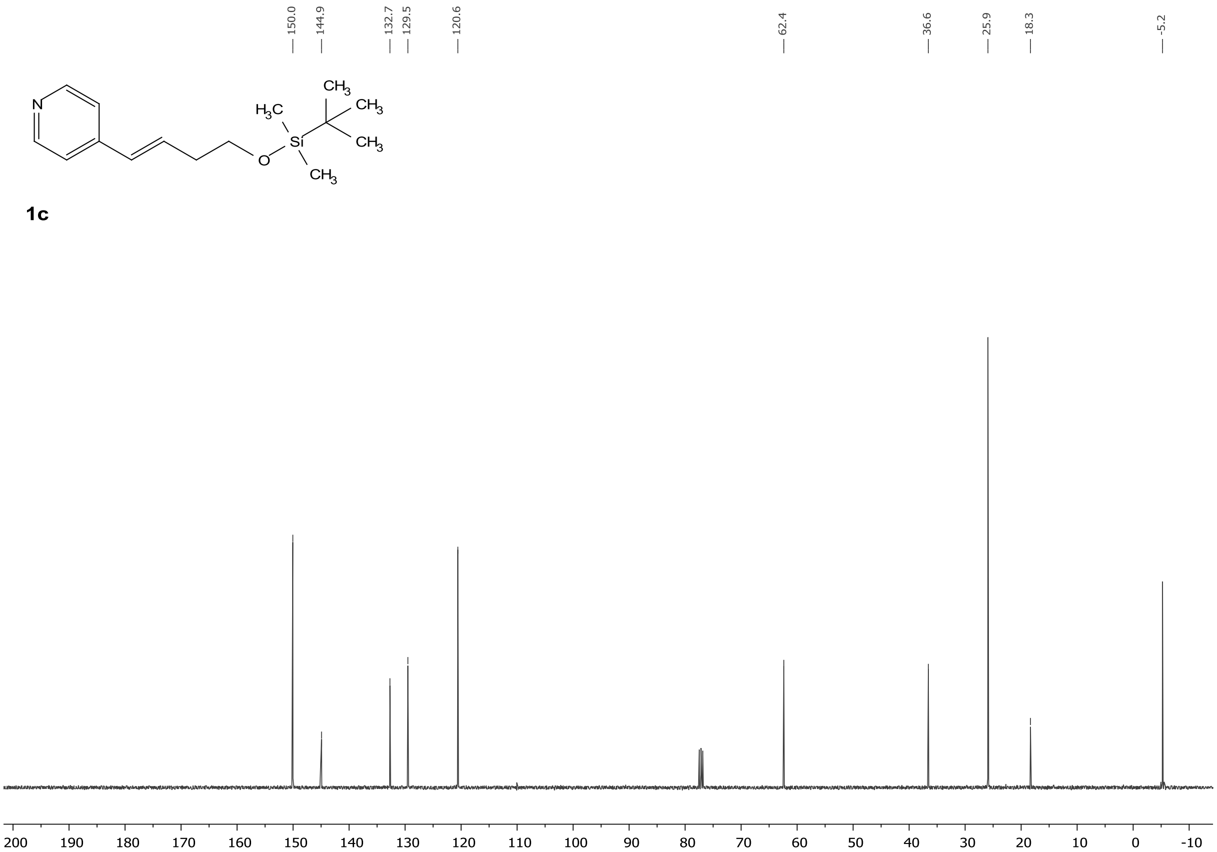


**Supplementary Figure 9** $^{13}$C NMR spectrum of **1c**

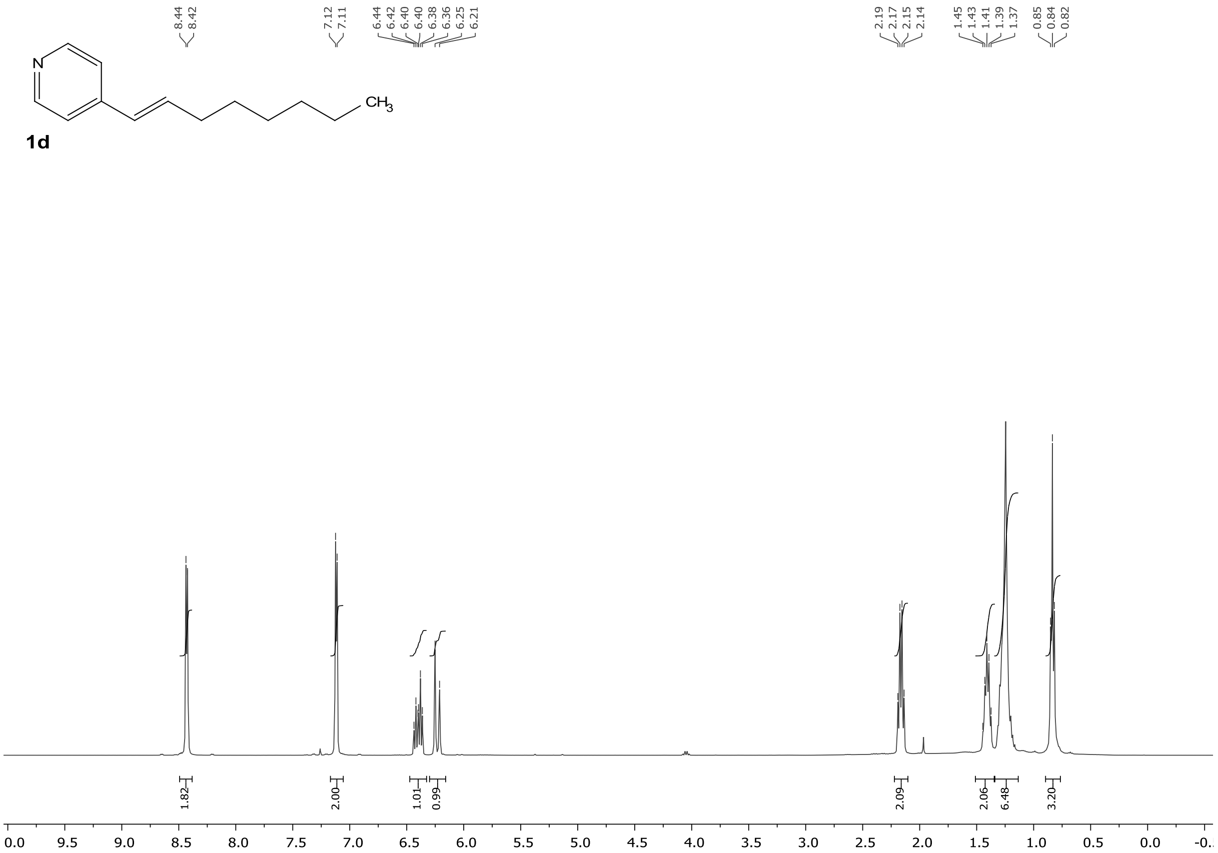


**Supplementary Figure 10** $^{1}$H NMR spectrum of **1d**

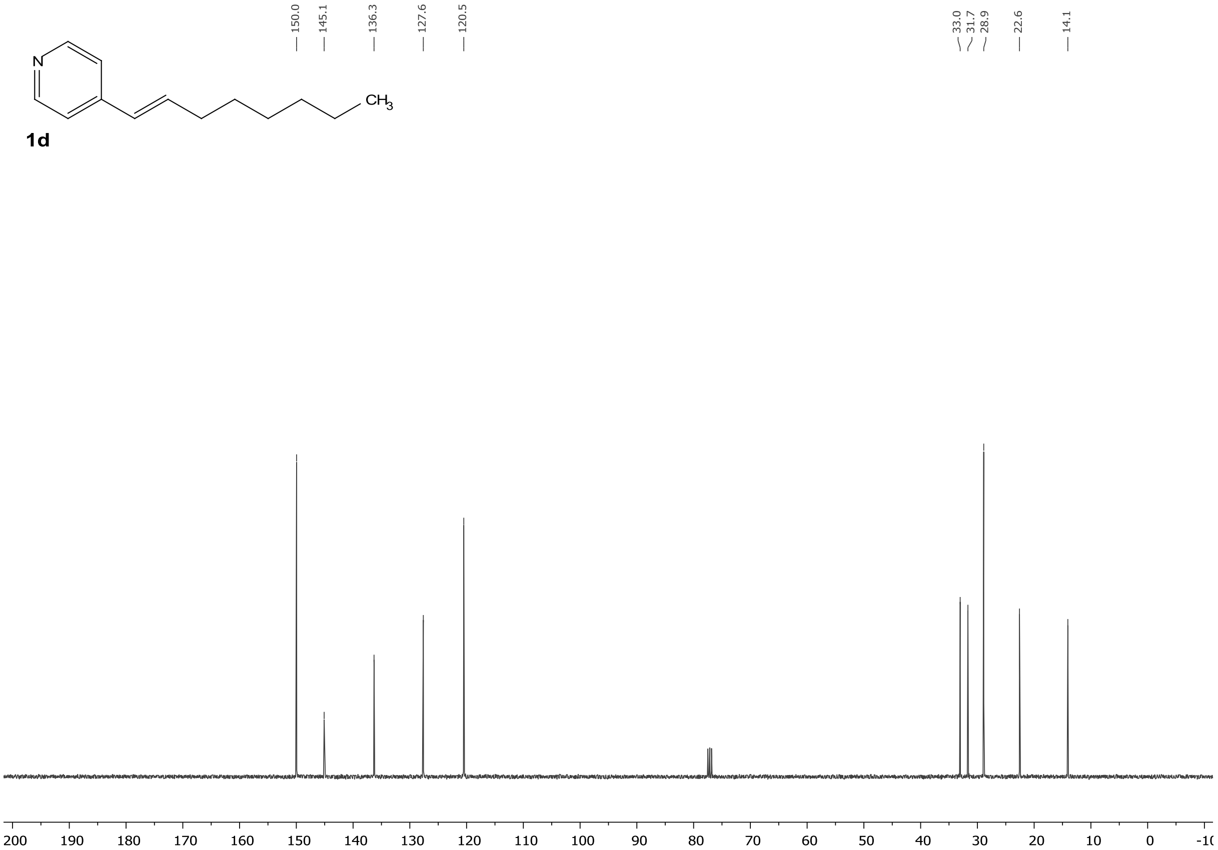


**Supplementary Figure 11** $^{13}$C NMR spectrum of **1d**

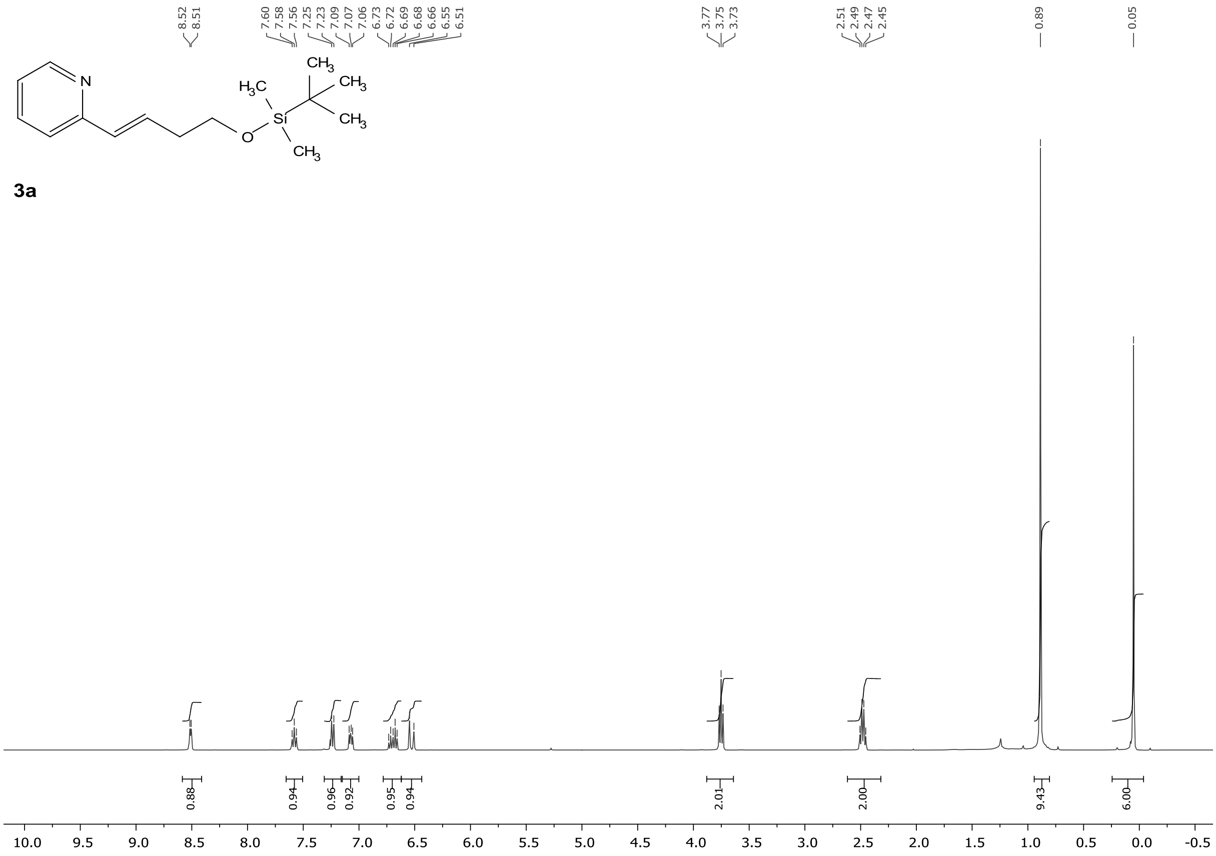


**Supplementary Figure 12** $^{1}$H NMR spectrum of **3a**

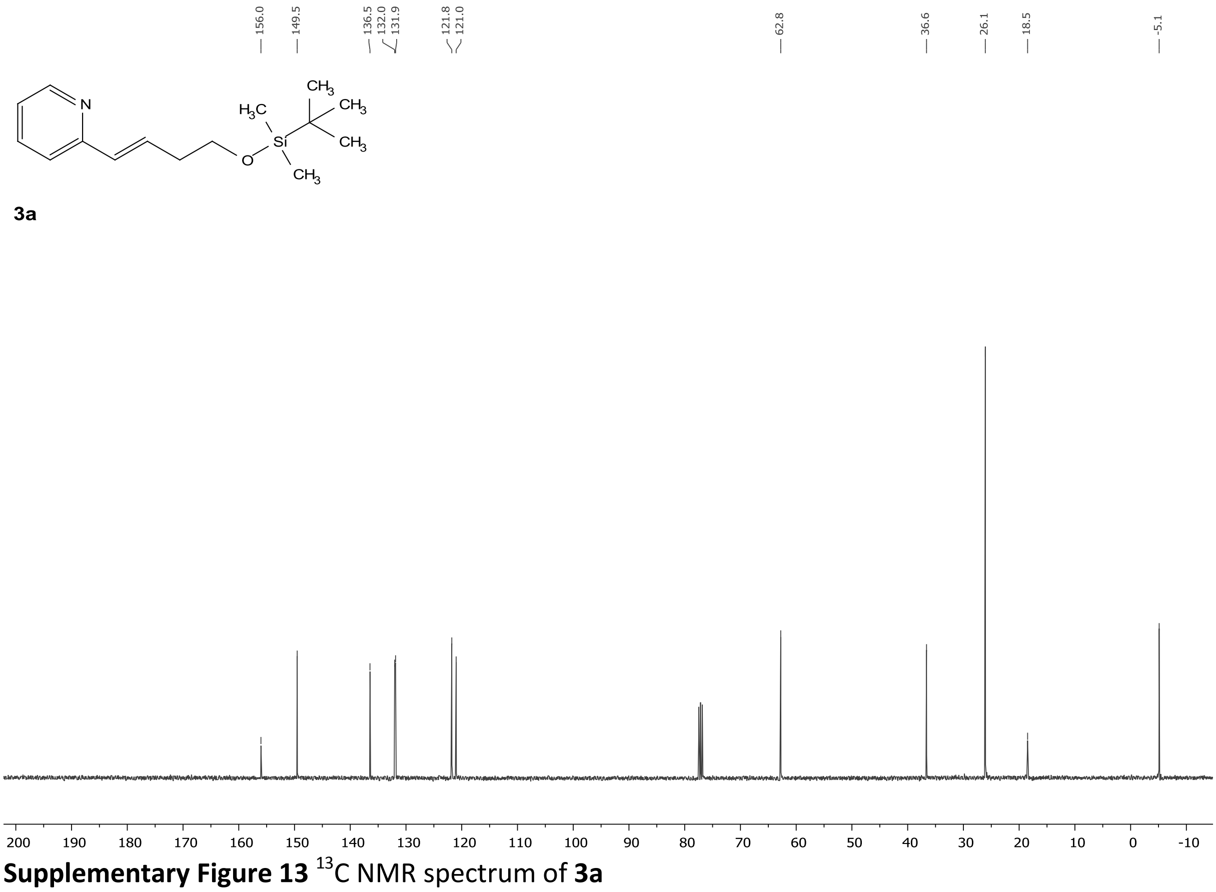


**Supplementary Figure 13** $^{13}$C NMR spectrum of **3a**

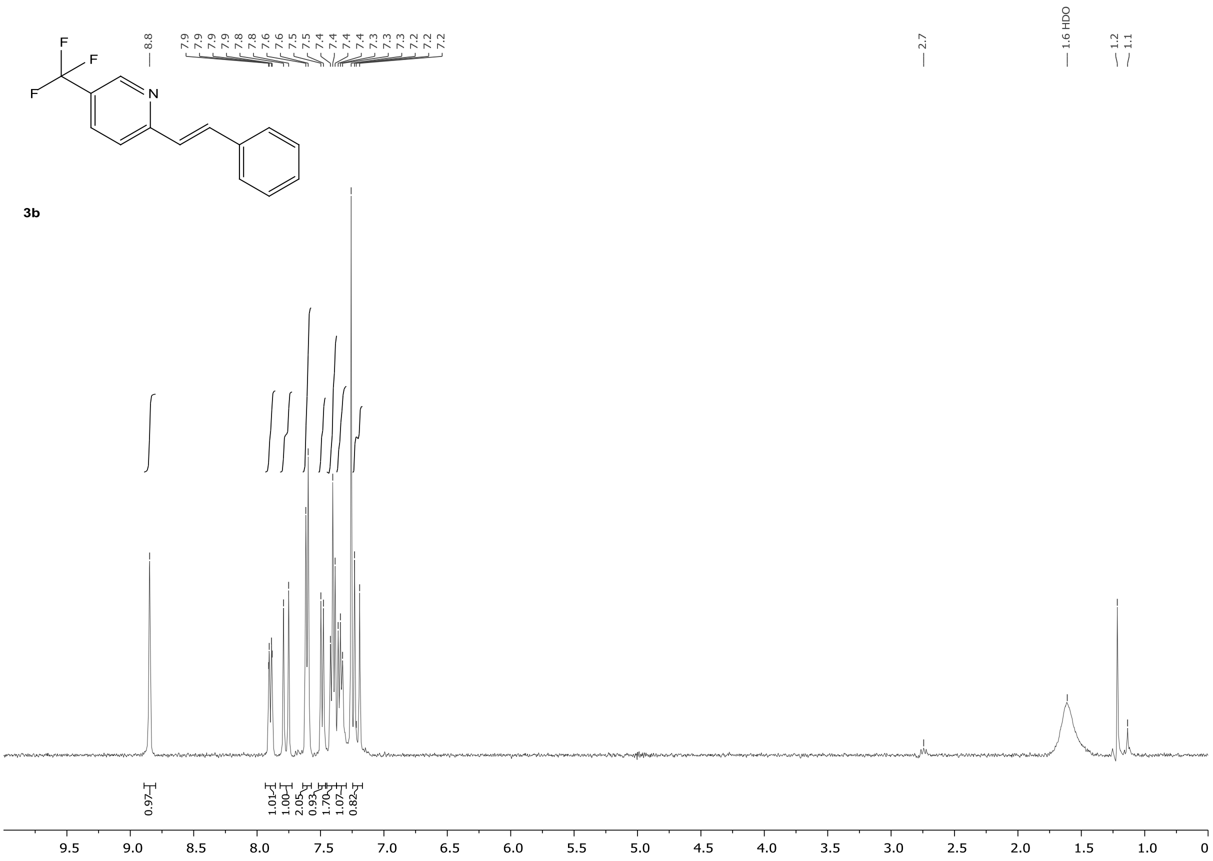

**Supplementary Figure 14** $^{1}$H NMR spectrum of **3b**

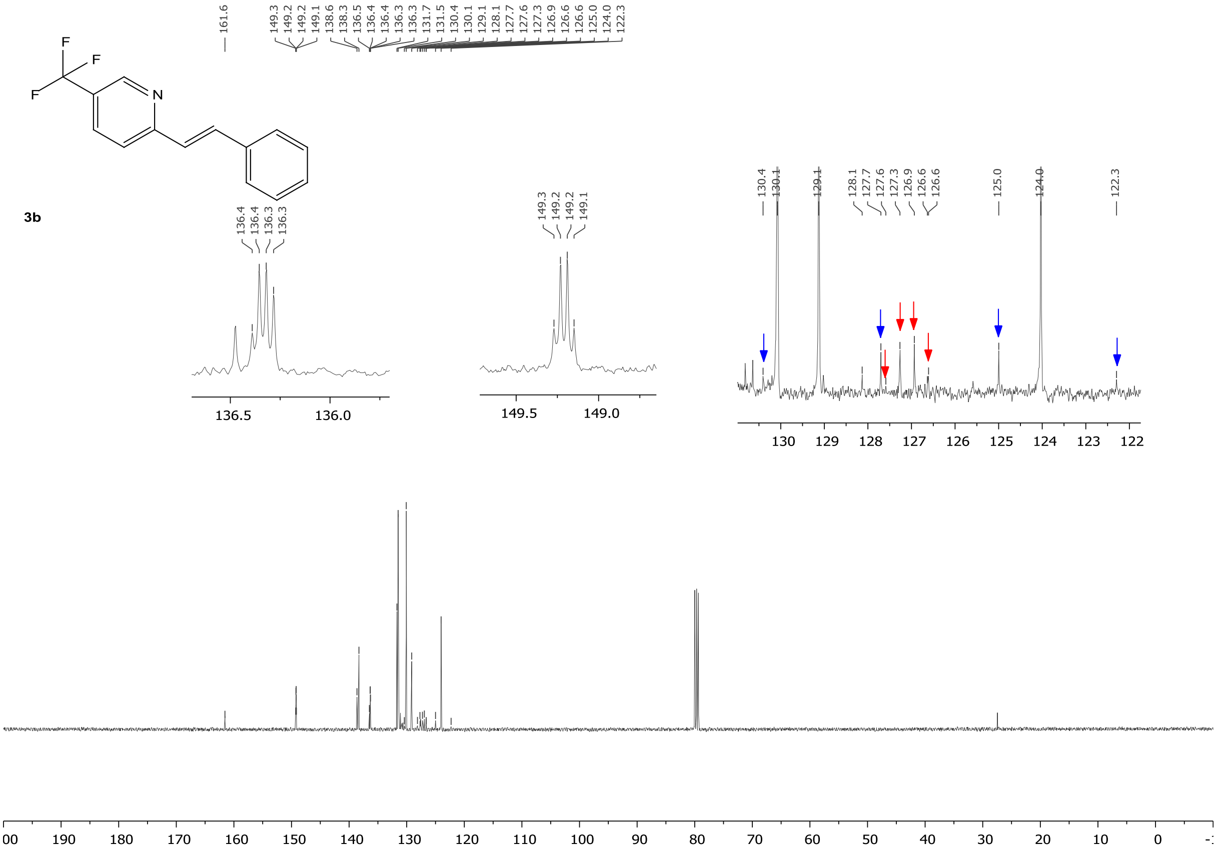

**Supplementary Figure 15** $^{13}$C NMR spectrum of **3b**

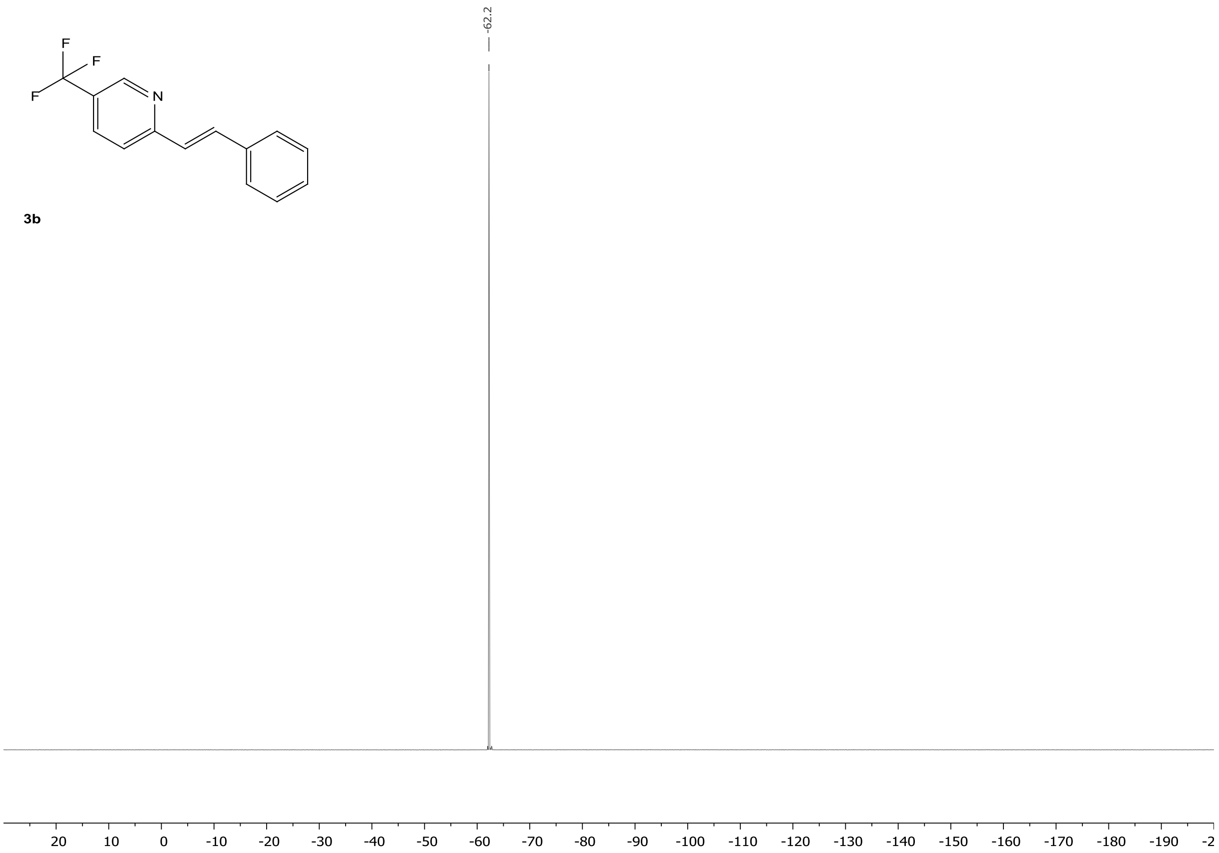

**Supplementary Figure 16** $^{19}$F NMR spectrum of **3b**

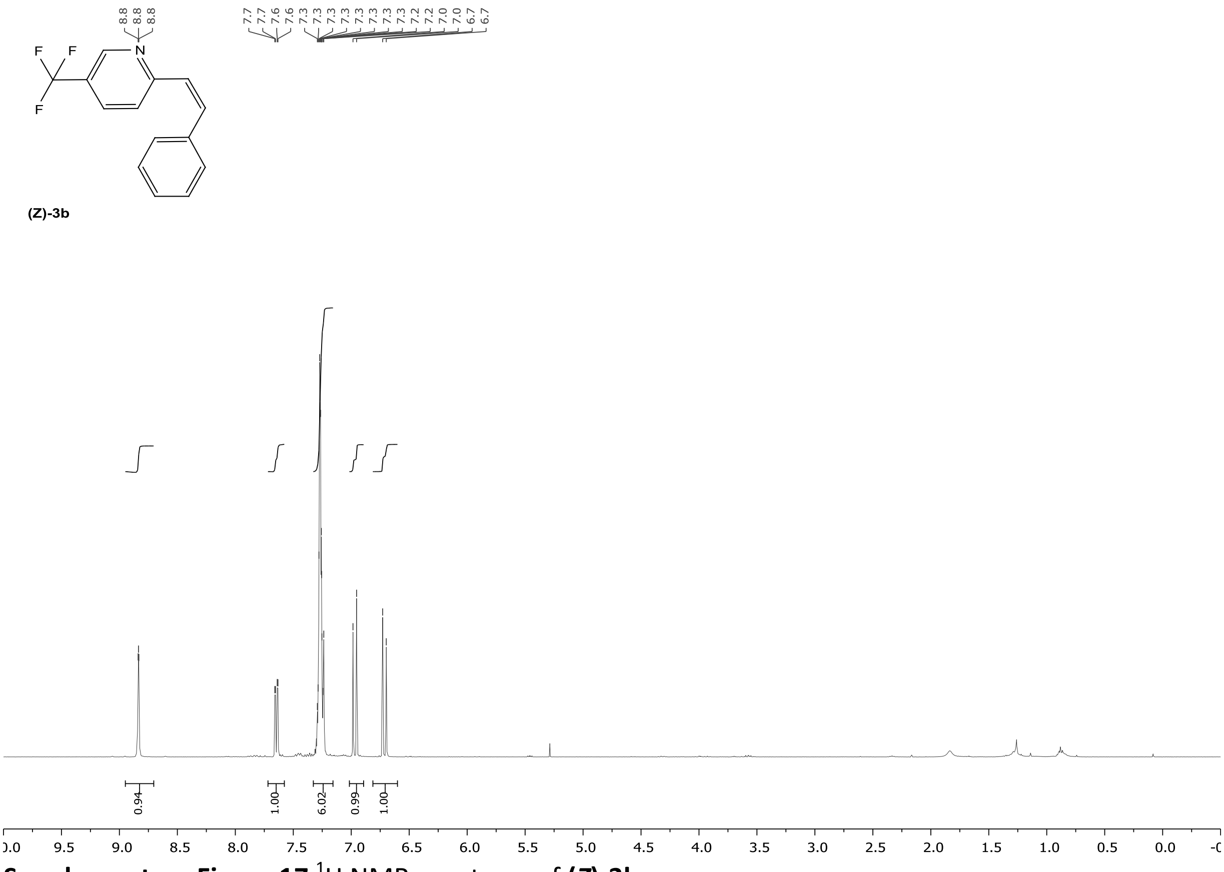

**Supplementary Figure 17** $^{1}$H NMR spectrum of **(*Z*)-3b**

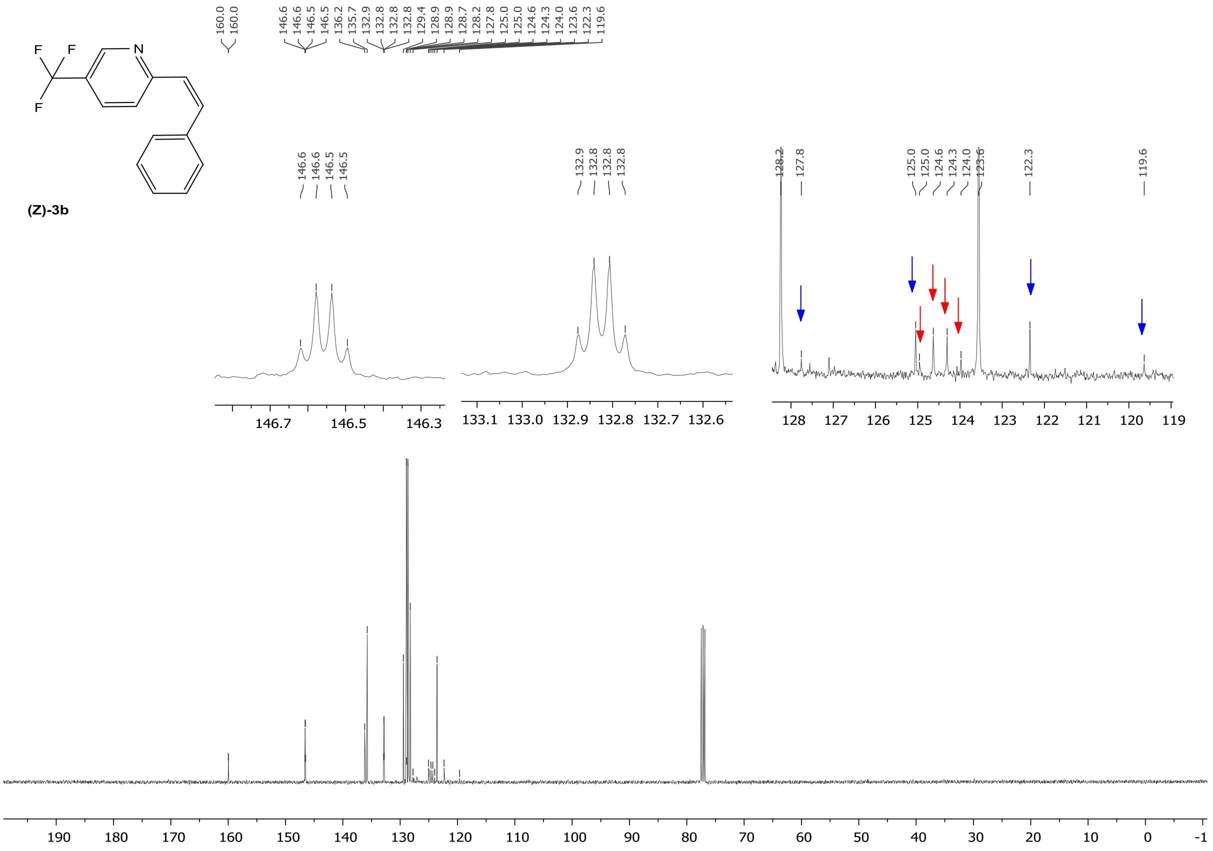


**Supplementary Figure 18** $^{13}$C NMR spectrum of ***(Z)*-3b**

:

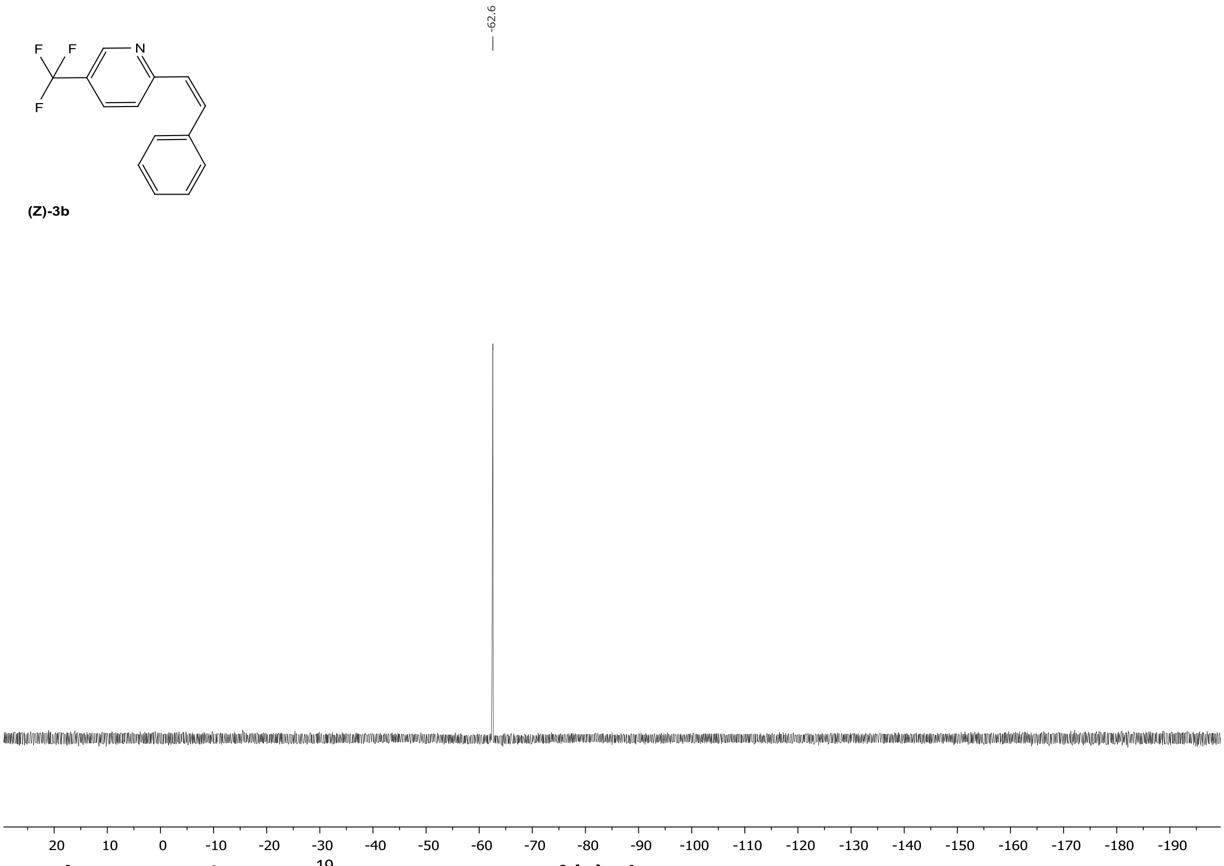


**Supplementary Figure 19** $^{19}$F NMR spectrum of ***(Z)*-3b**

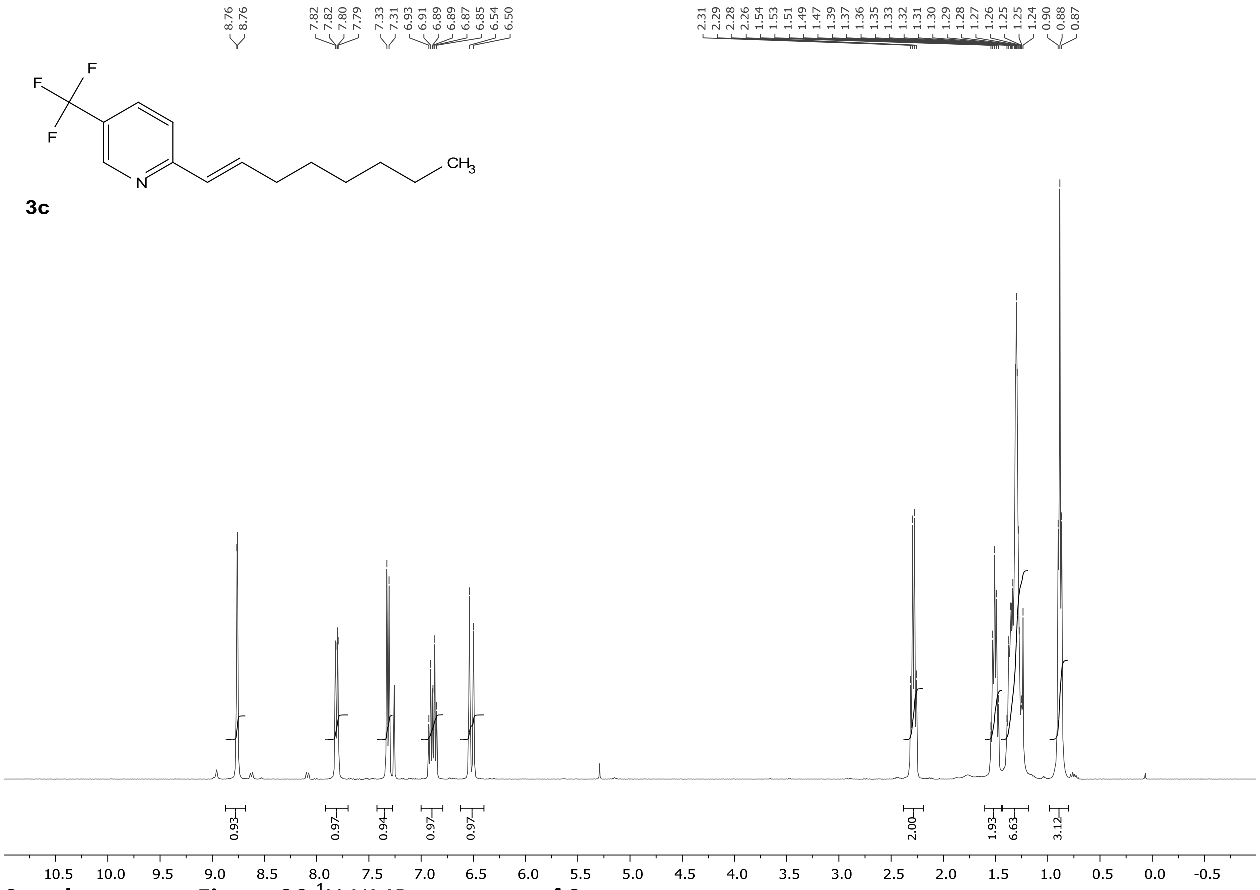

**Supplementary Figure 20** $^{1}$H NMR spectrum of **3c**

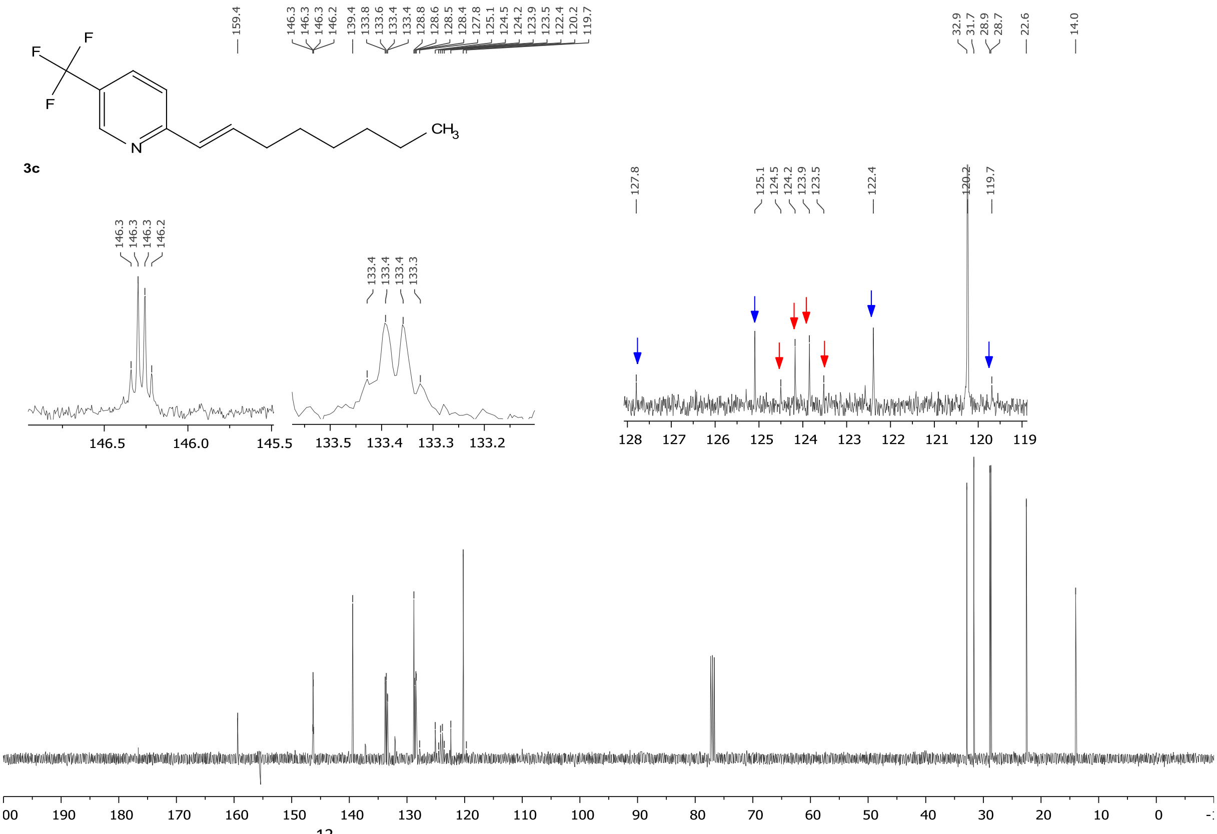

**Supplementary Figure 21** $^{13}$C NMR spectrum of **3c**

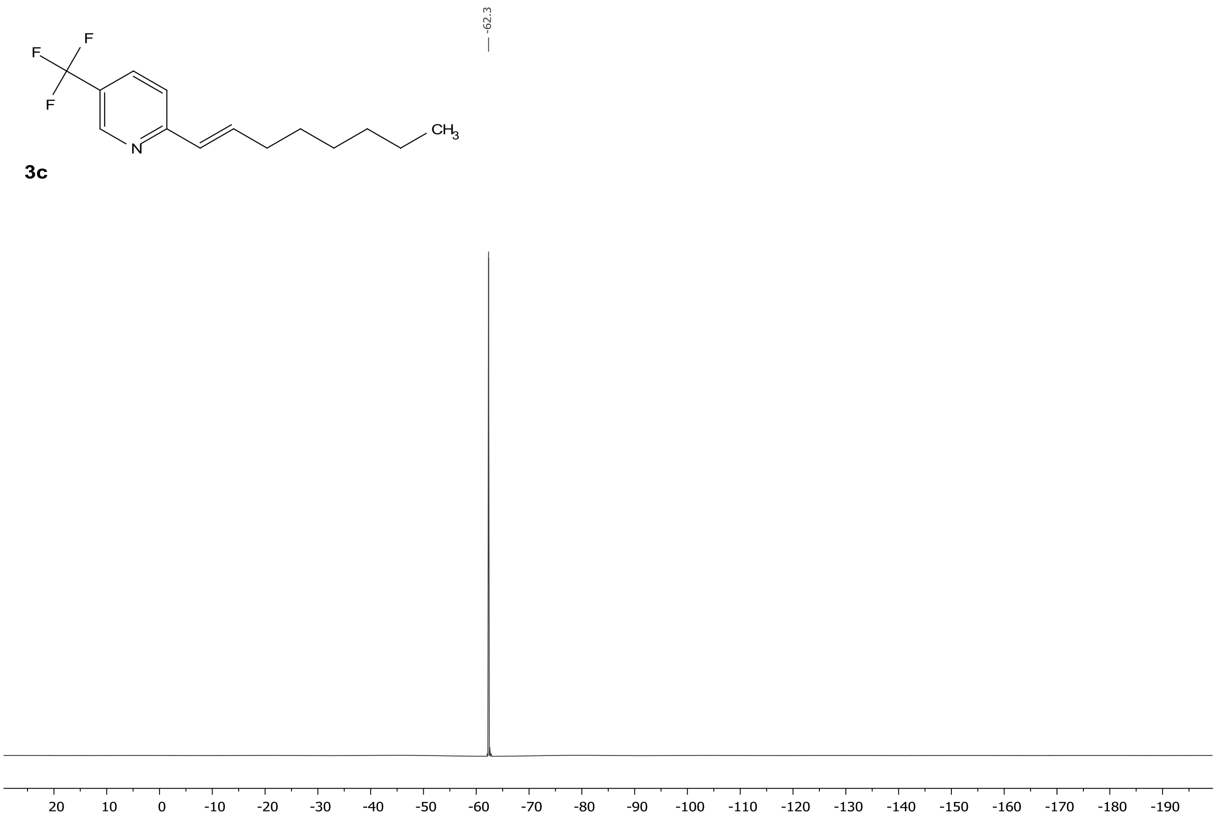

**Supplementary Figure 22** $^{19}F$ NMR spectrum of **3c**

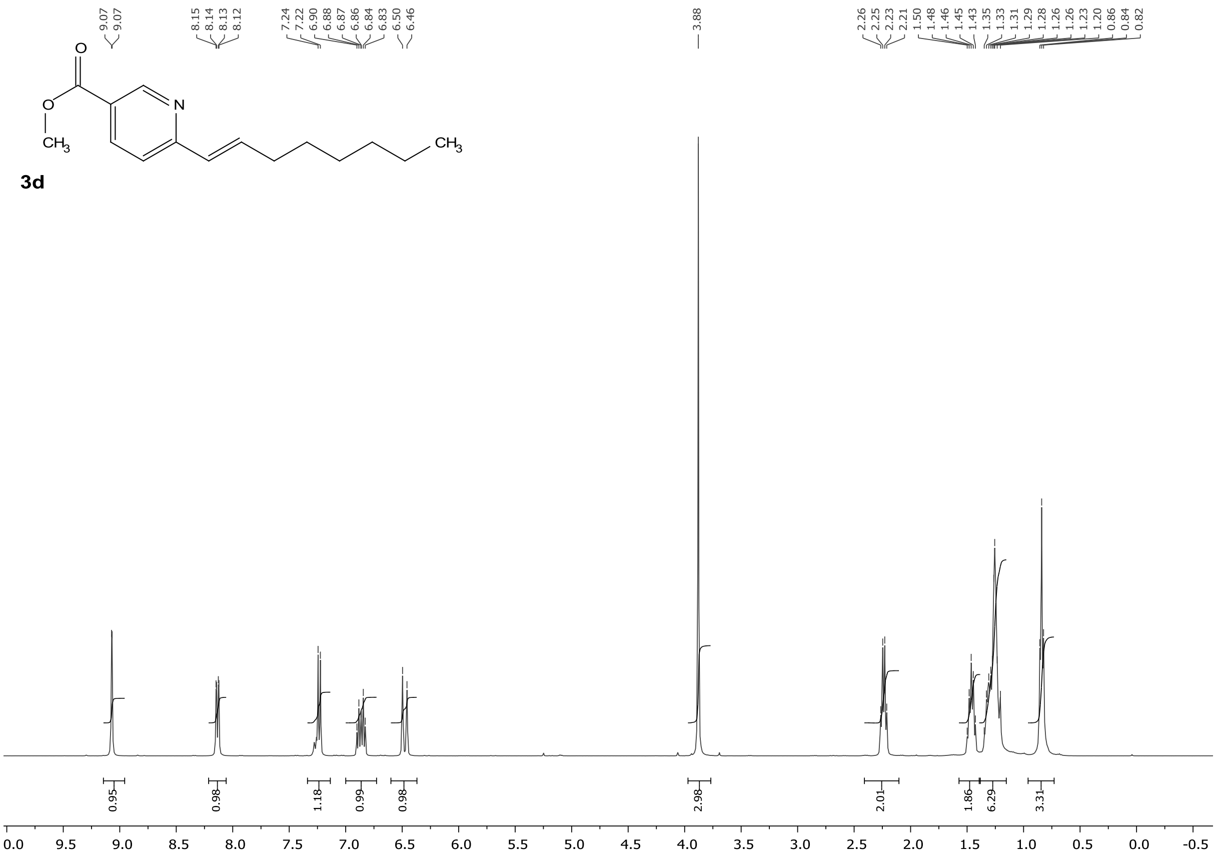

**Supplementary Figure 23** $^{1}H$ NMR spectrum of **3d**

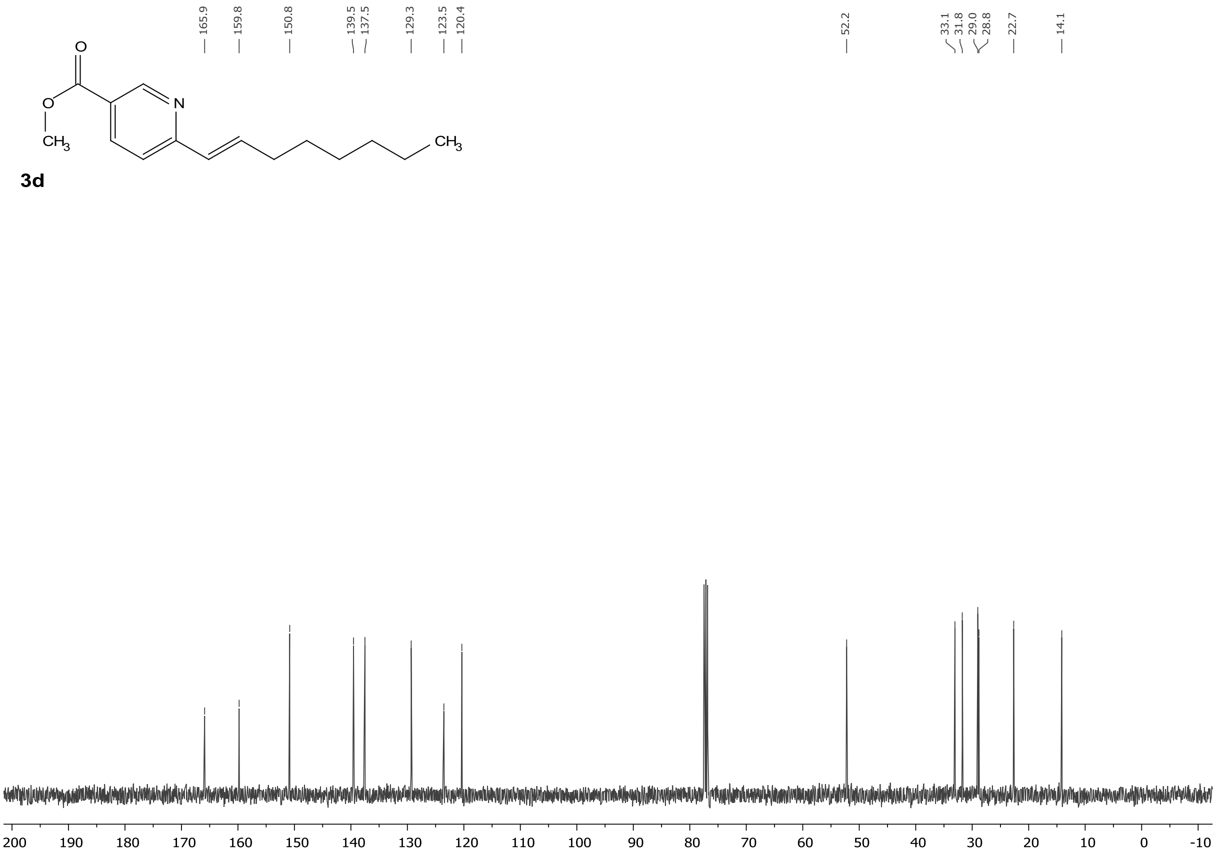


**Supplementary Figure 24** $^{13}$C NMR spectrum of **3d**

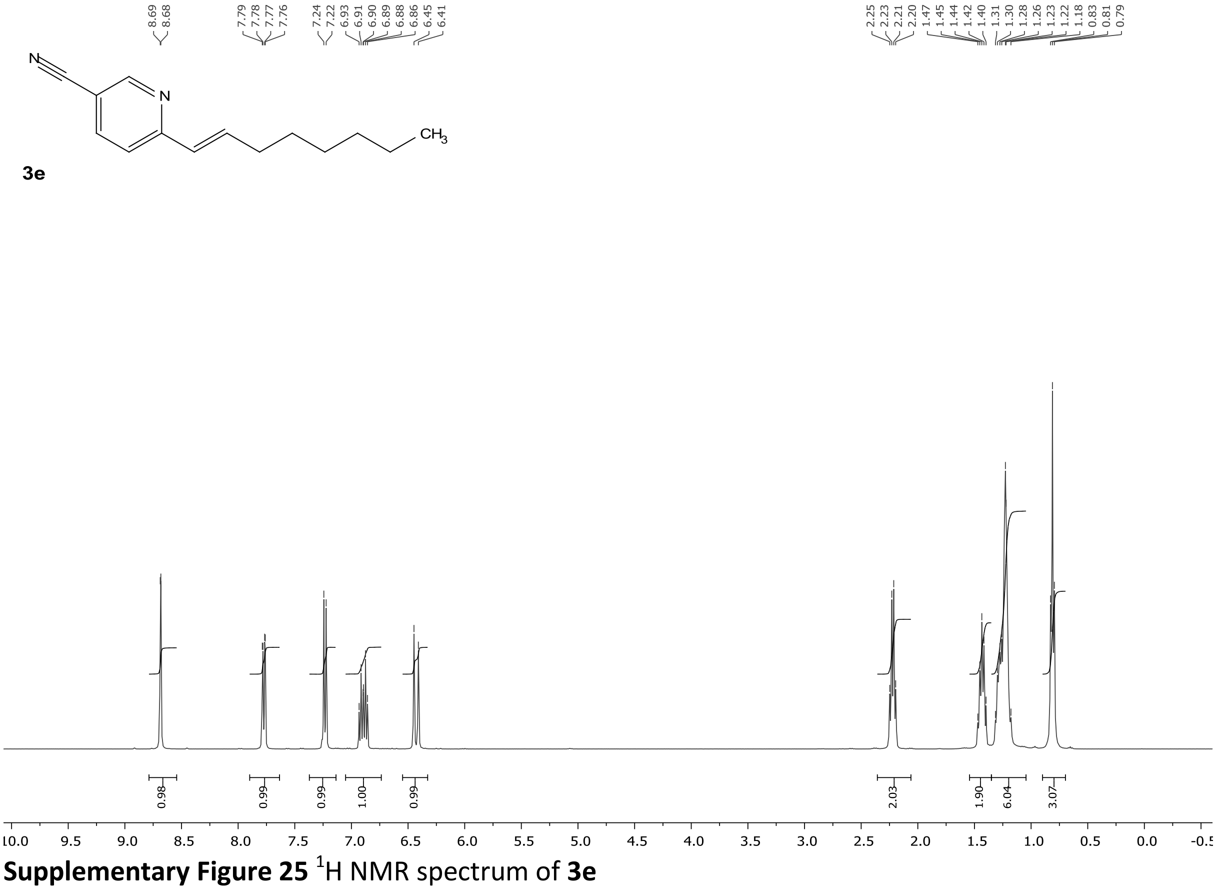


**Supplementary Figure 25** $^{1}$H NMR spectrum of **3e**

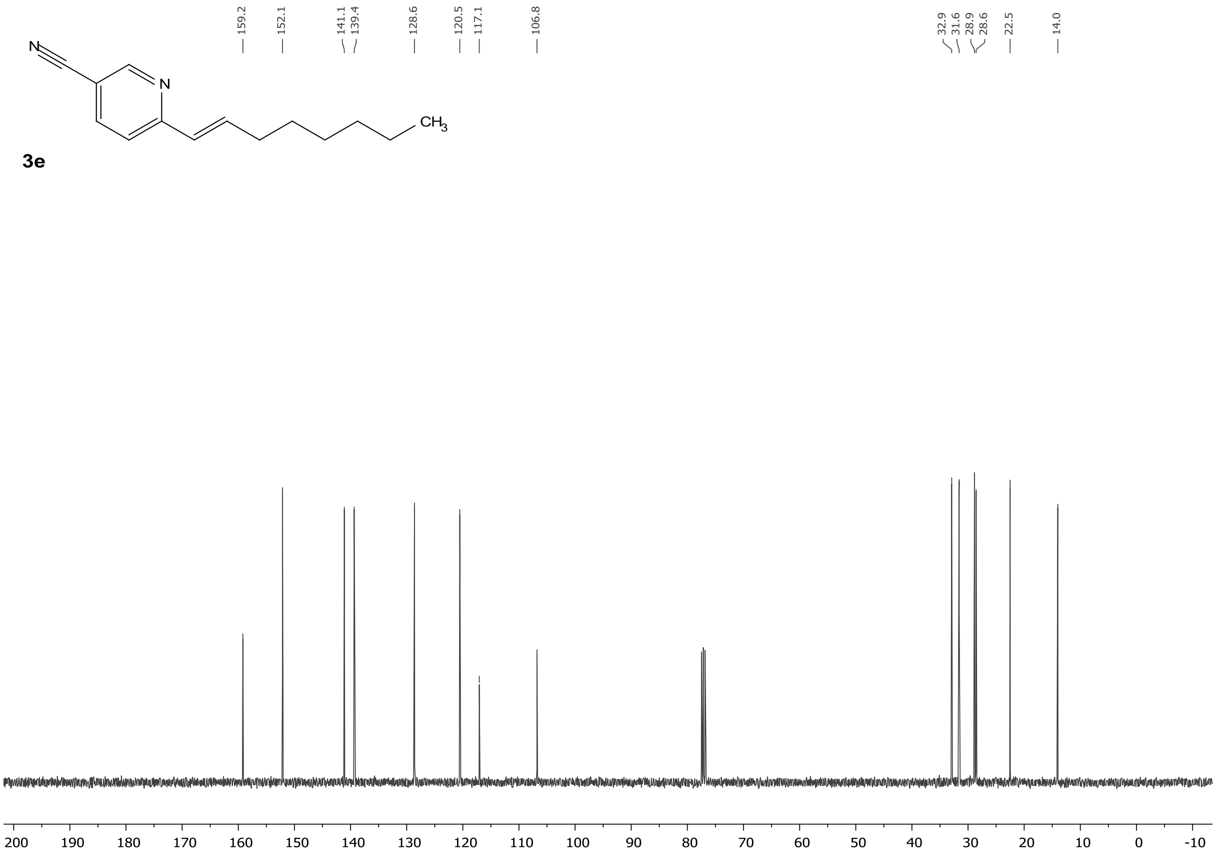


**Supplementary Figure 26** $^{13}$C NMR spectrum of **3e**

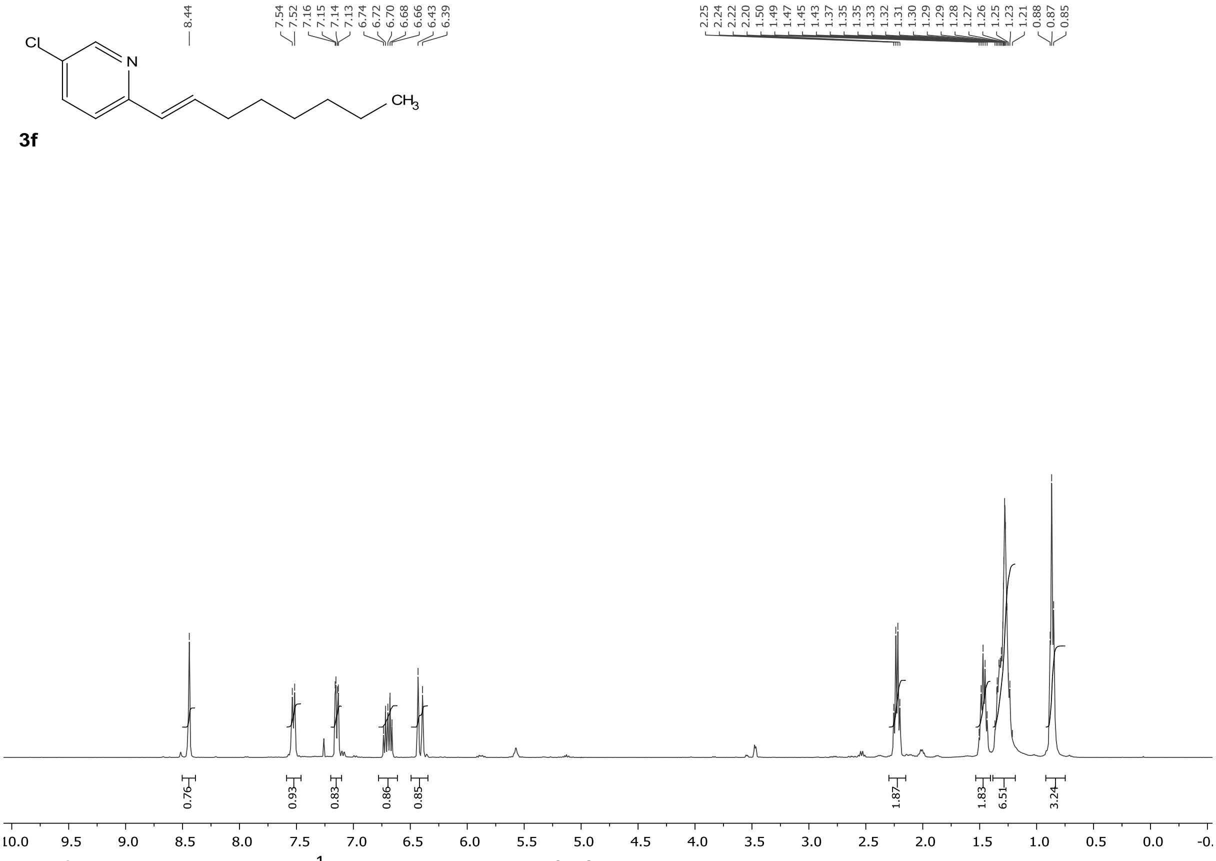


**Supplementary Figure 27** $^{1}$H NMR spectrum of **3f**

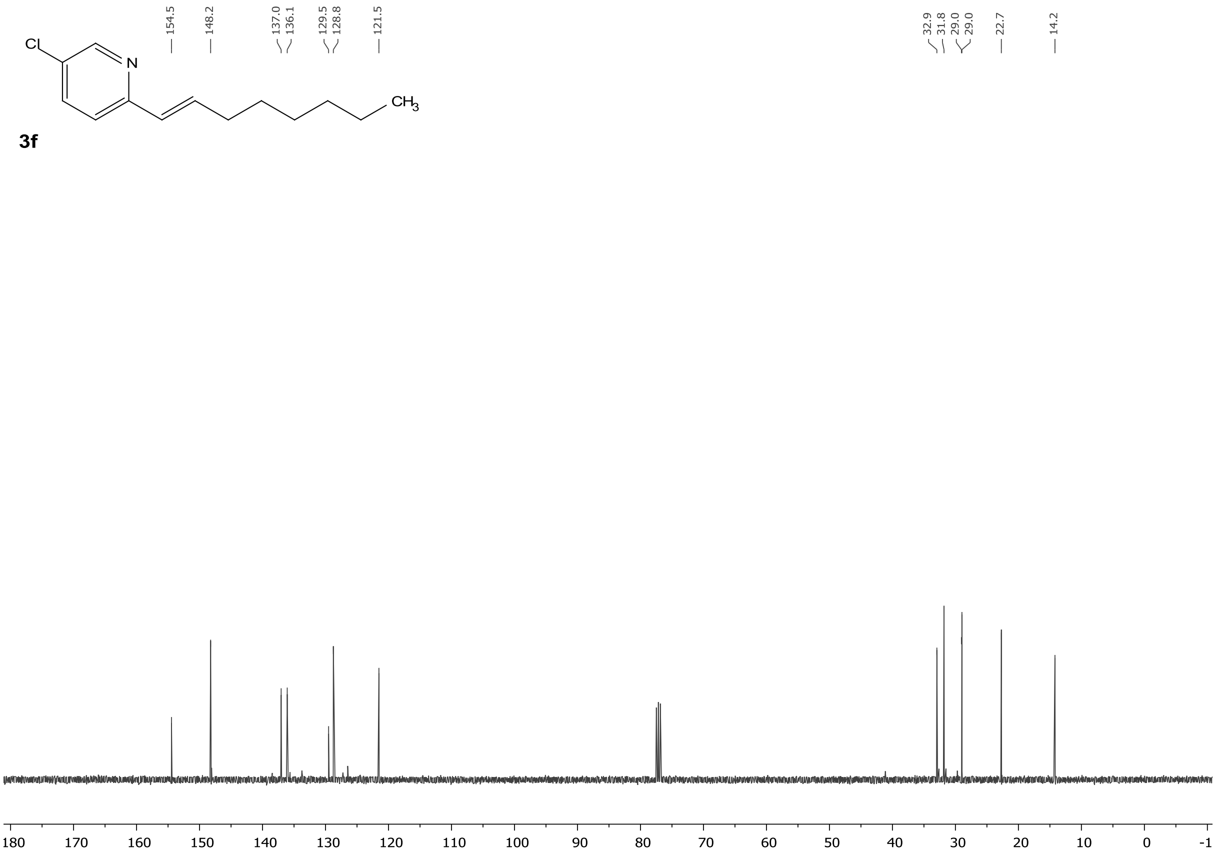


**Supplementary Figure 28** $^{13}$C NMR spectrum of **3f**

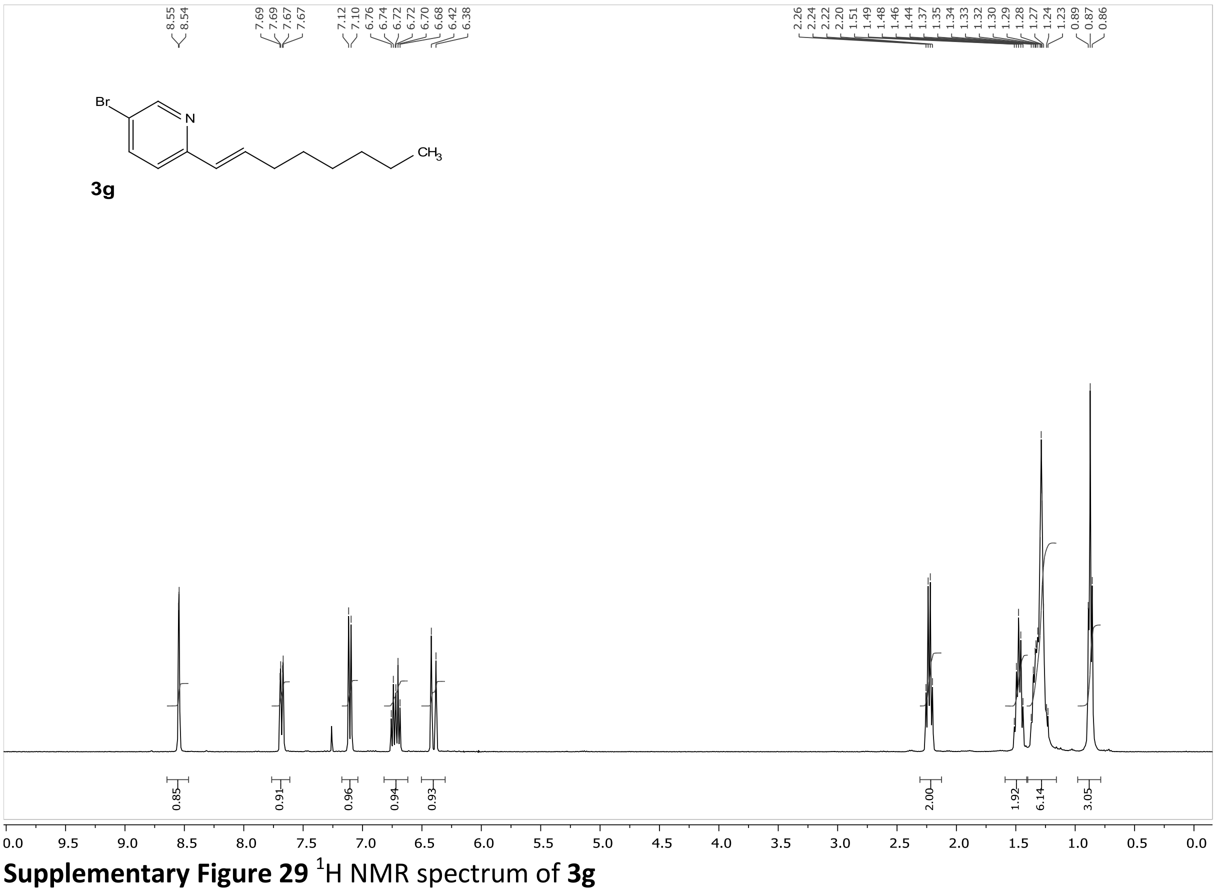


**Supplementary Figure 29** $^{1}$H NMR spectrum of **3g**

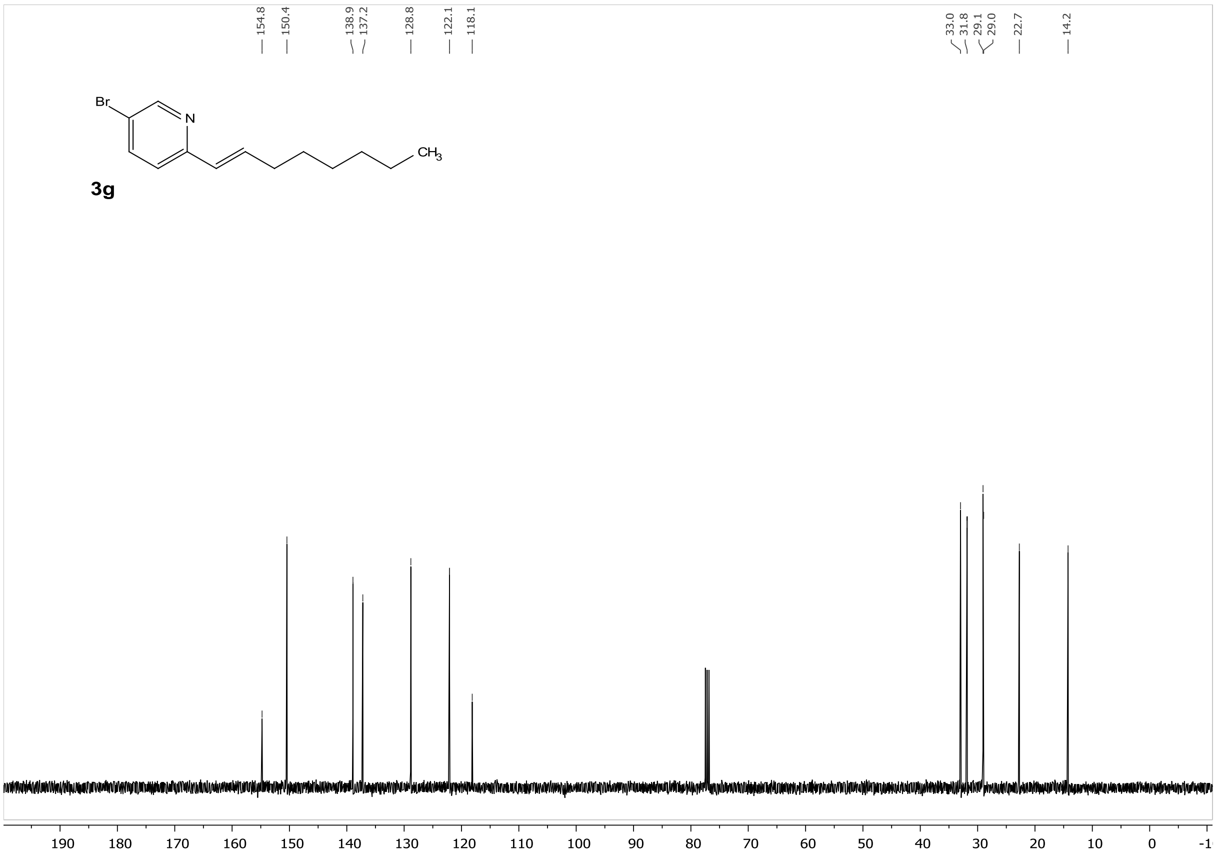

**Supplementary Figure 30** $^{13}$C NMR spectrum of **3g**

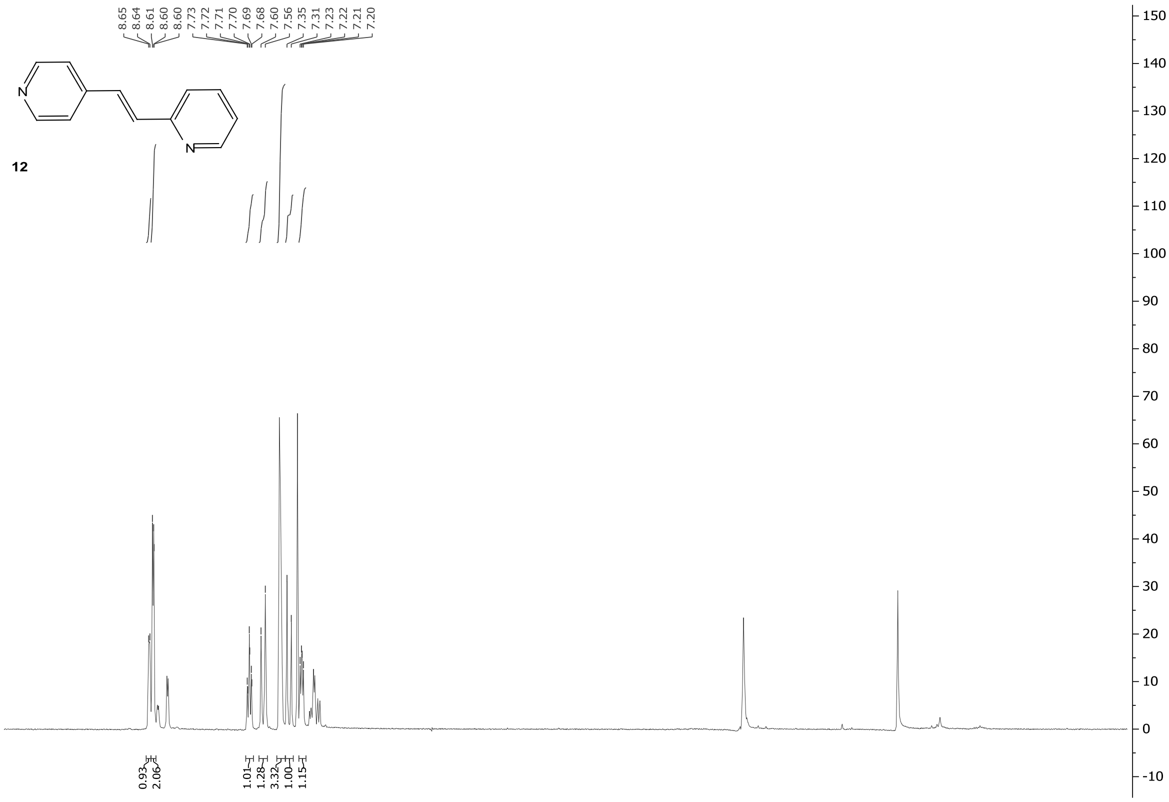

**Supplementary Figure 31** $^{1}$H NMR spectrum of **12**

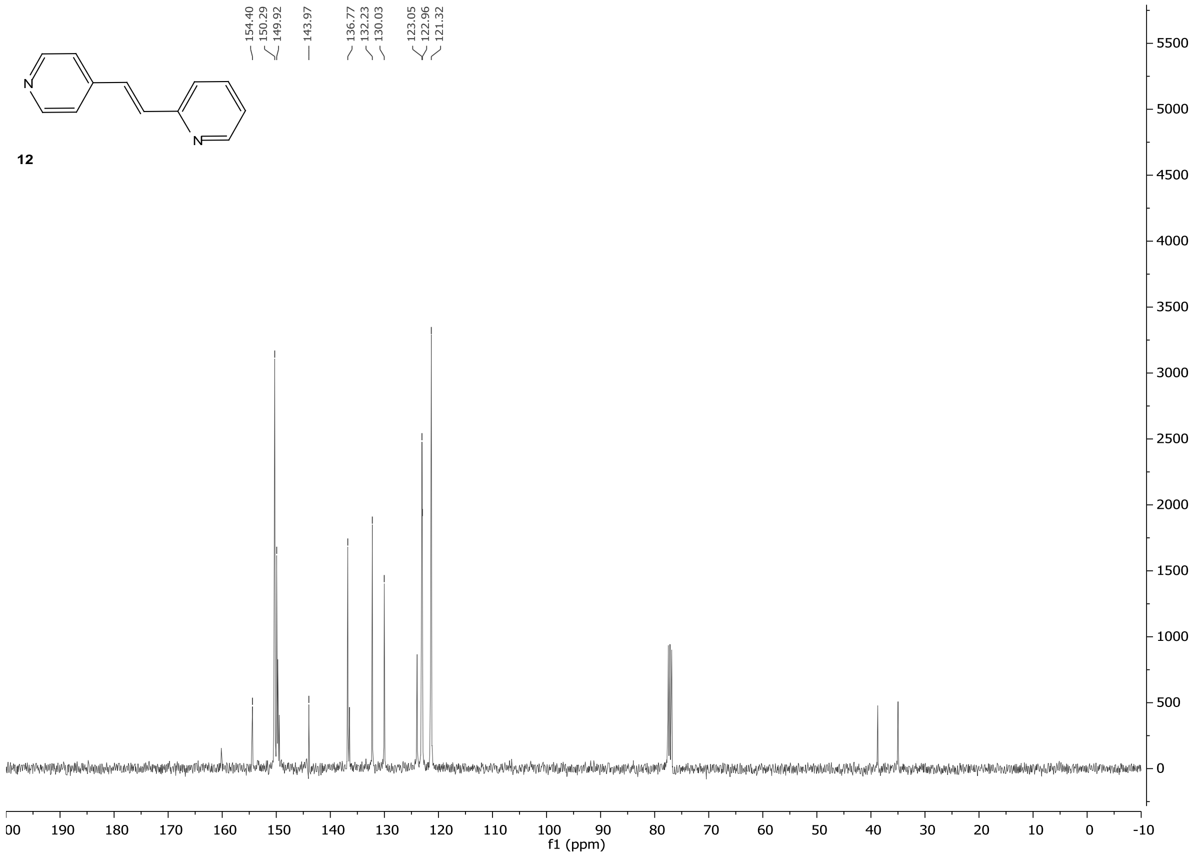

**Supplementary Figure 32** $^{13}$C NMR spectrum of **12**

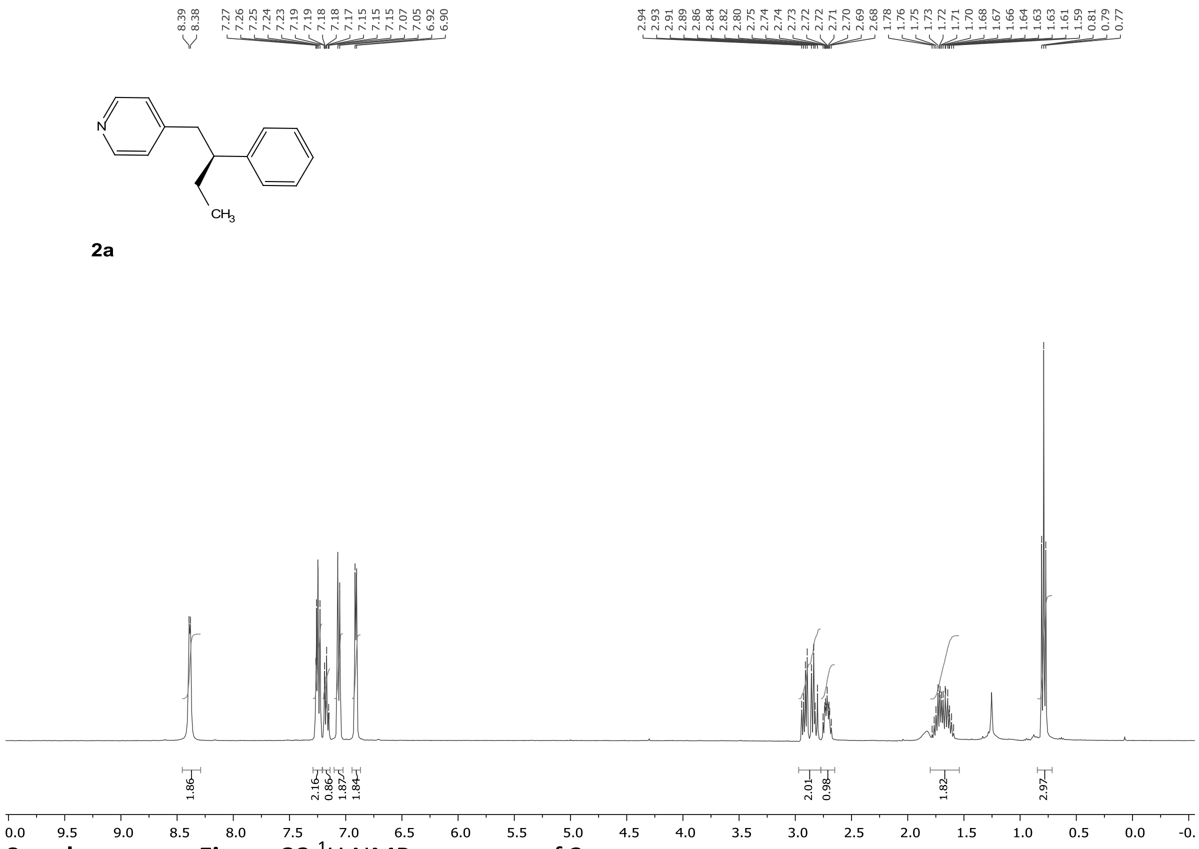

**Supplementary Figure 33** $^{1}$H NMR spectrum of **2a**

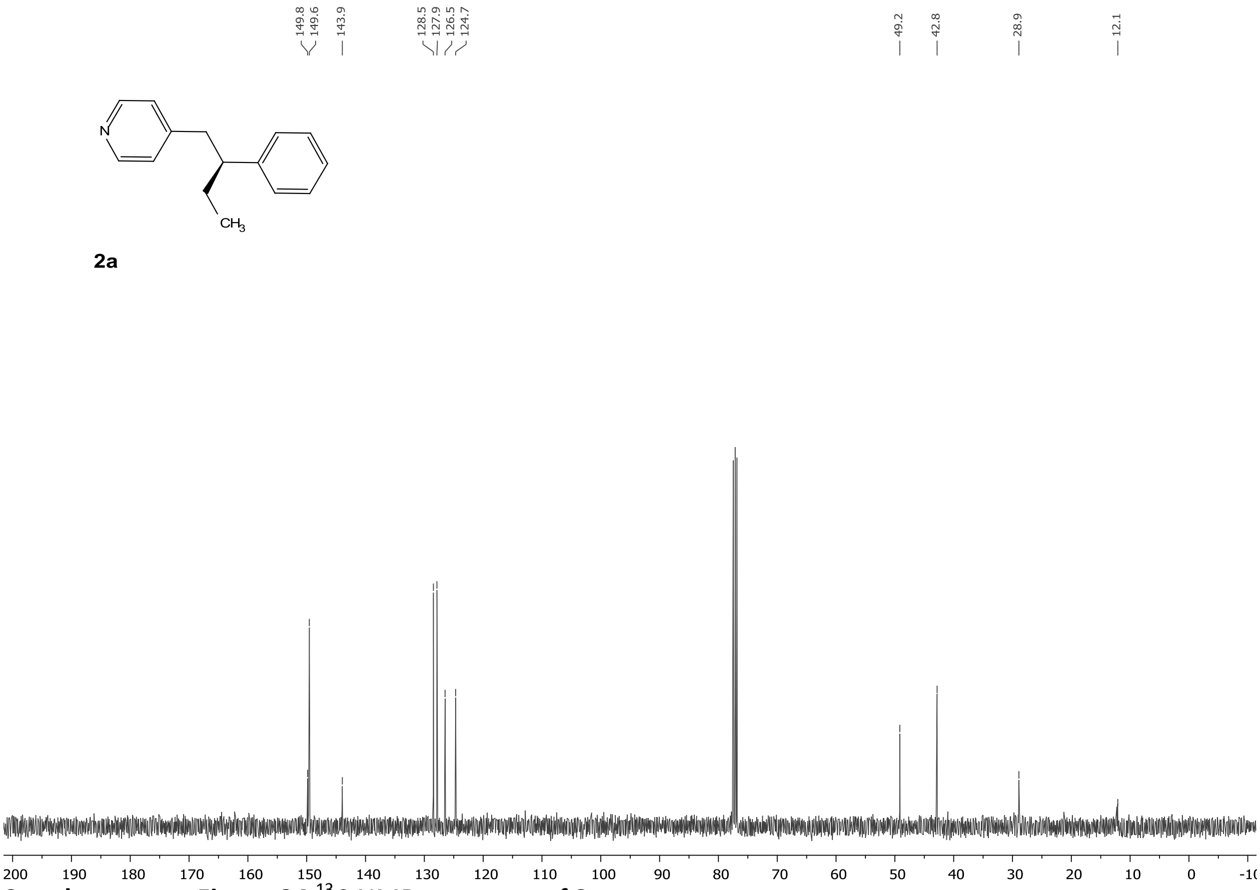


**2a**

**Supplementary Figure 34** $^{13}$C NMR spectrum of **2a**

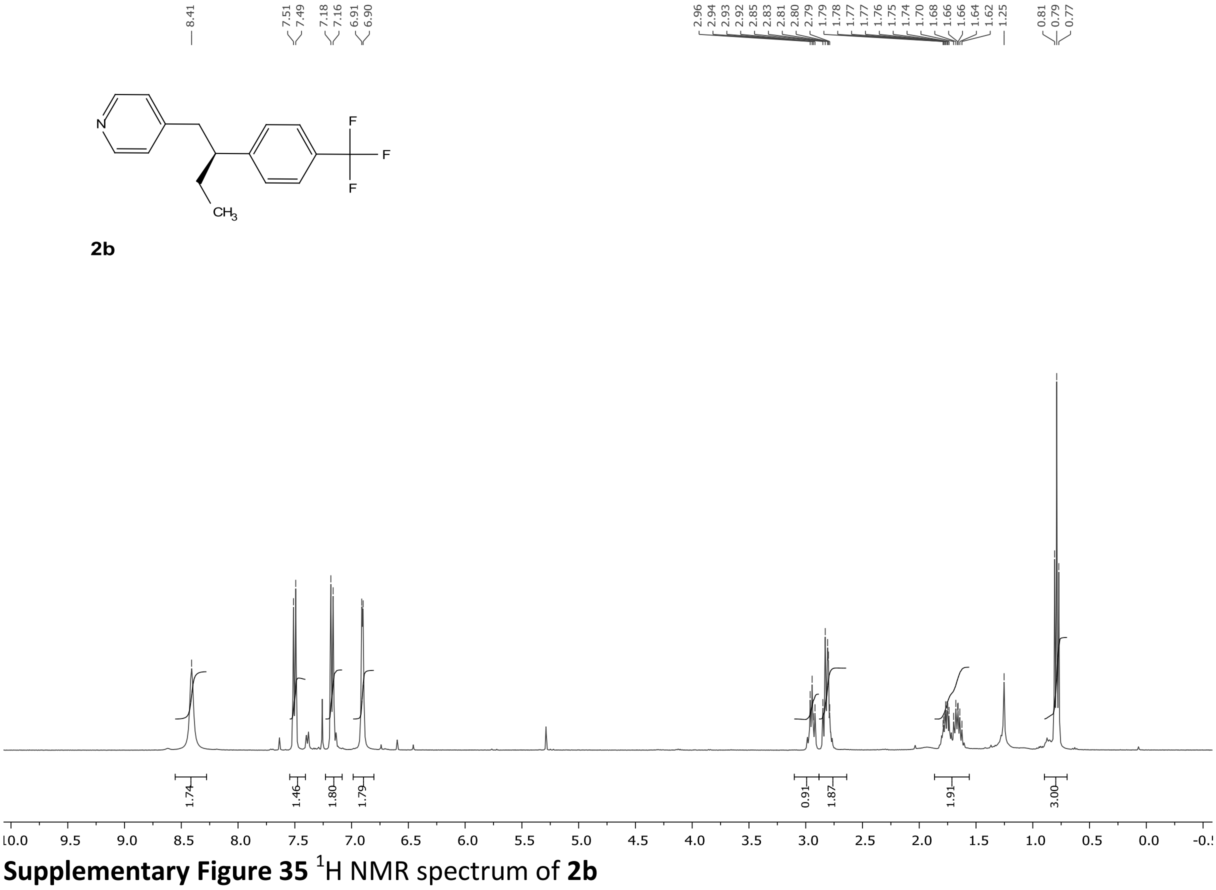


**2b**

**Supplementary Figure 35** $^{1}$H NMR spectrum of **2b**

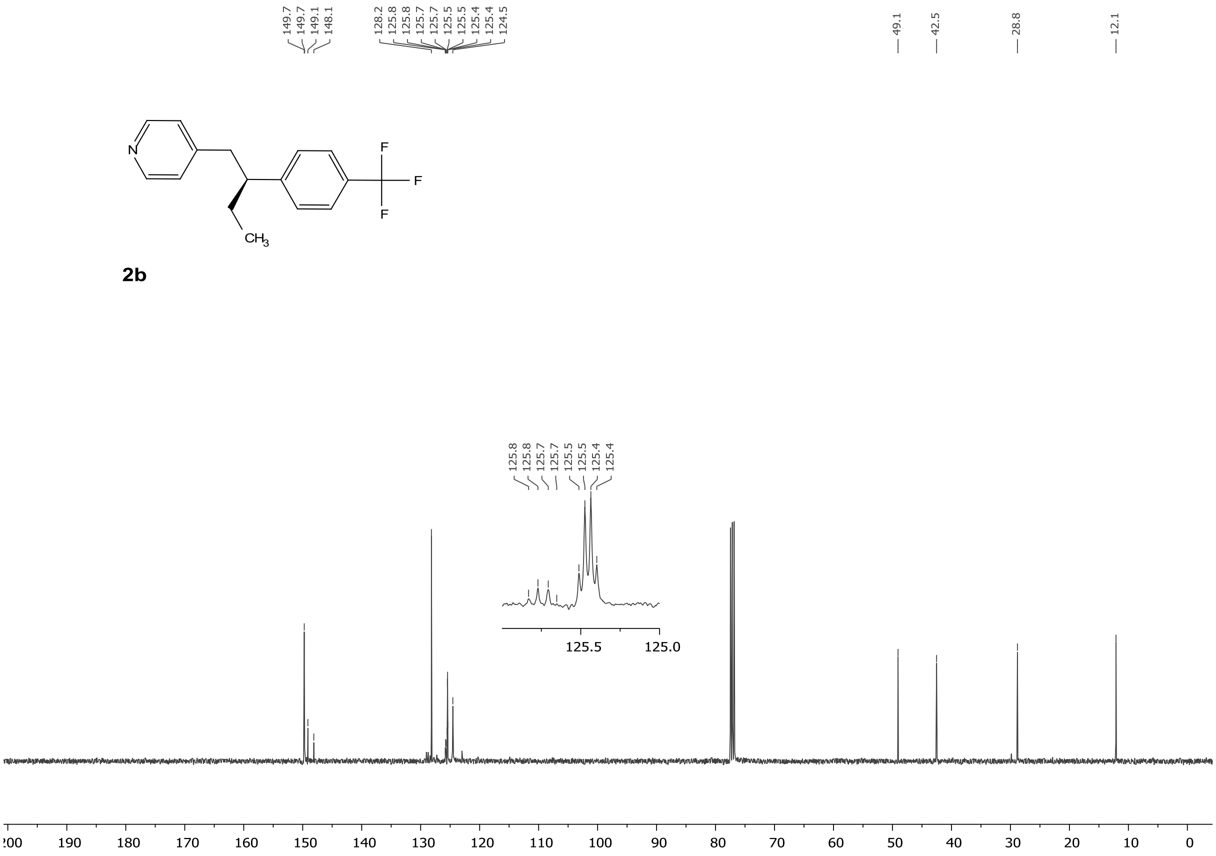


**Supplementary Figure 36** $^{13}$C NMR spectrum of **2b**

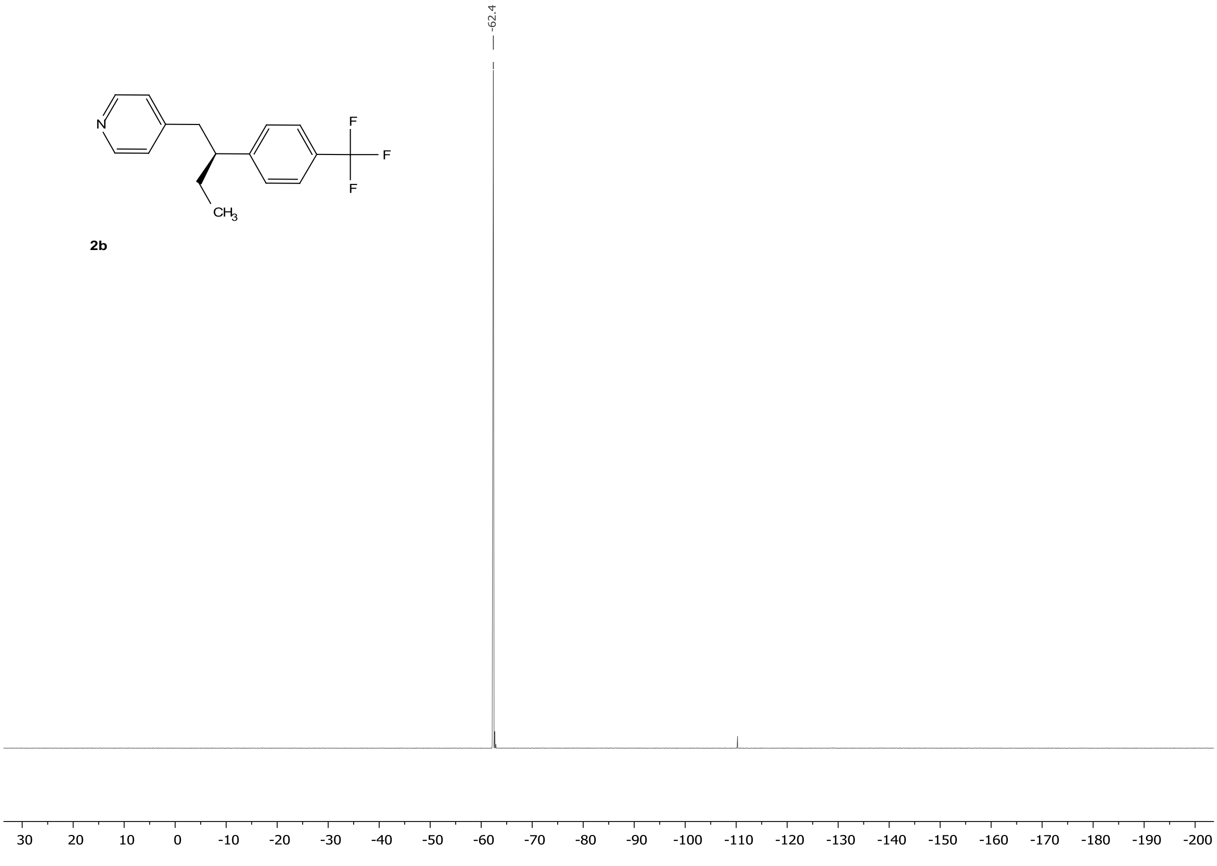


**Supplementary Figure 37** $^{19}$F NMR spectrum of **2b**

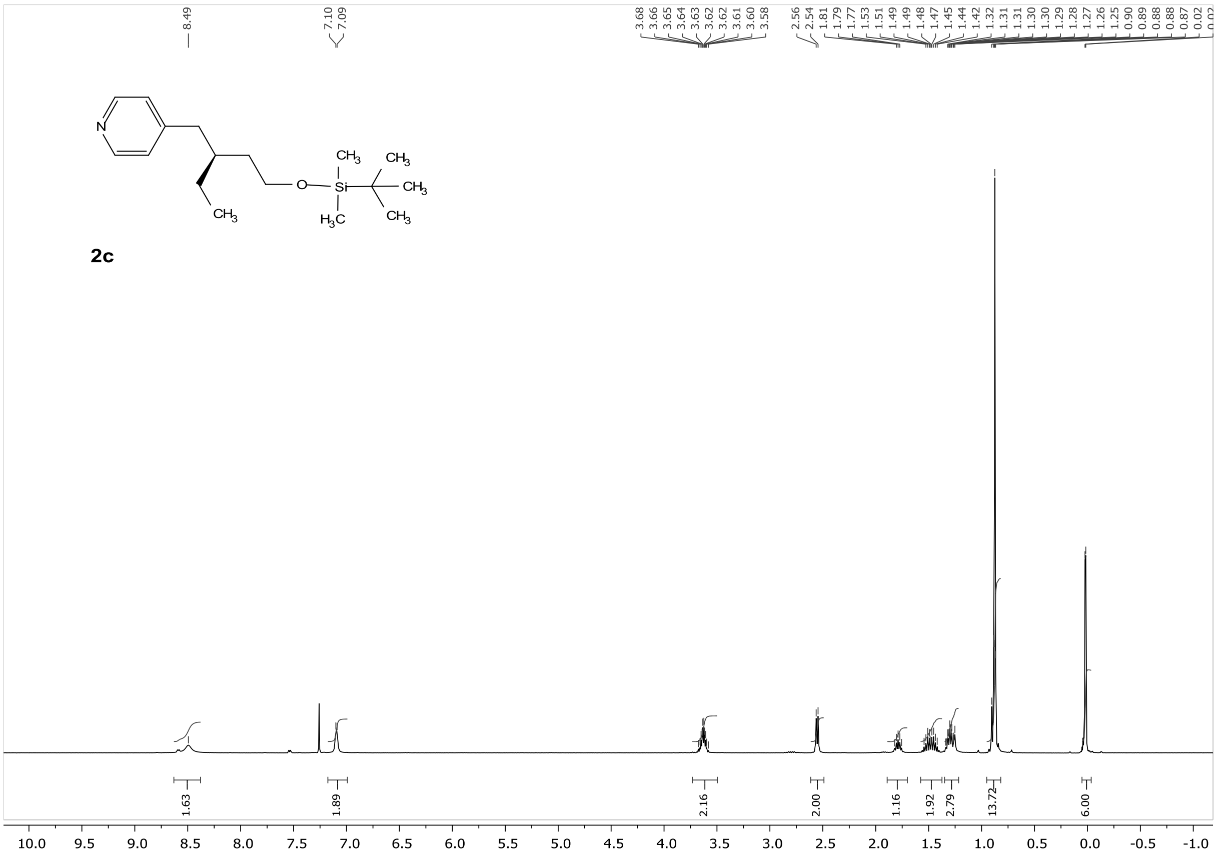

**Supplementary Figure 38** $^{1}$H NMR spectrum of **2c**

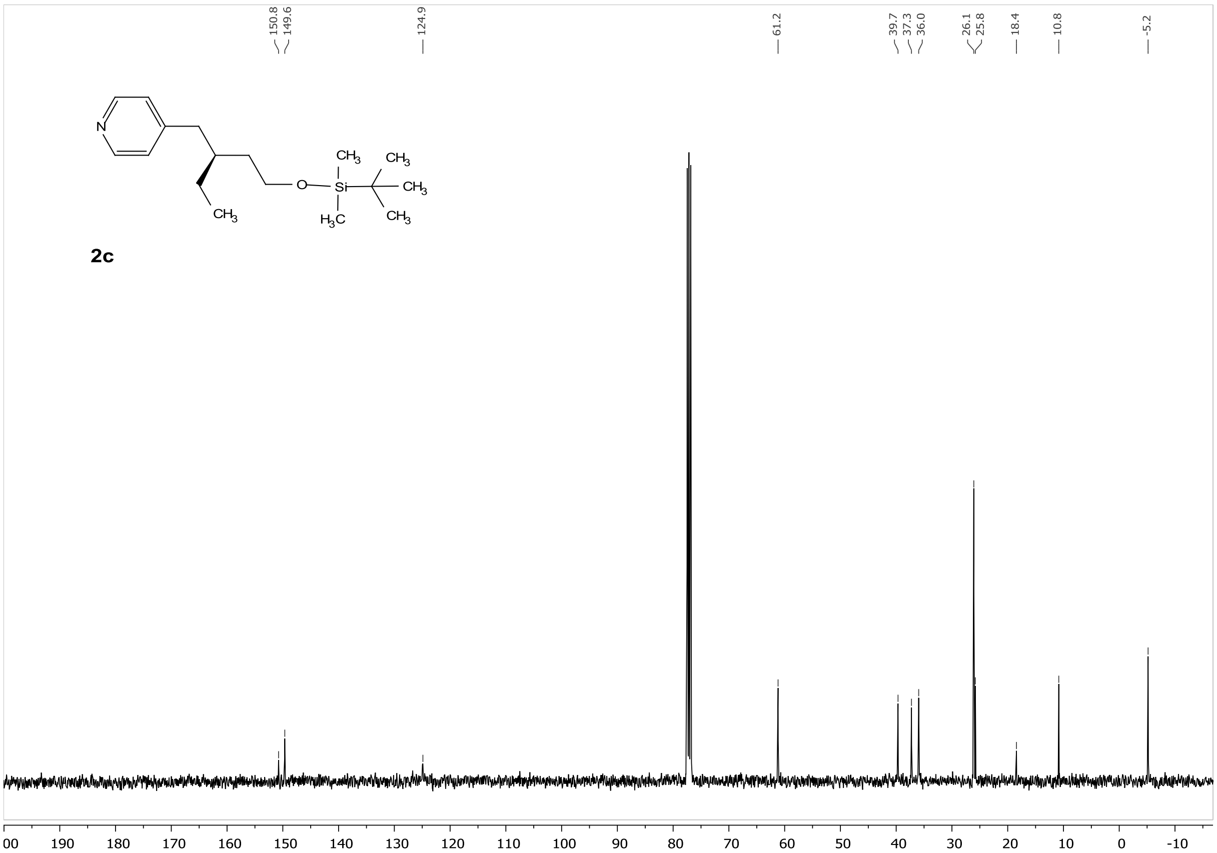

**Supplementary Figure 39** $^{13}$C NMR spectrum of **2c**

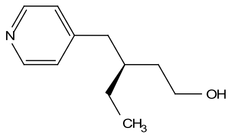


**2c'**

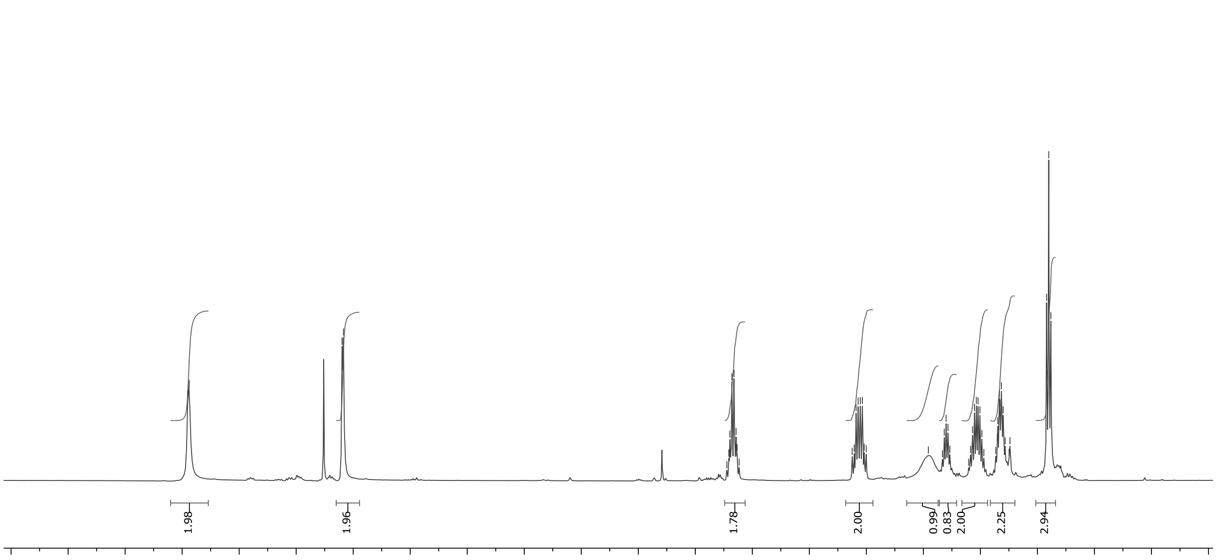


**Supplementary Figure 40** $^{1}$H NMR spectrum of **2c’**

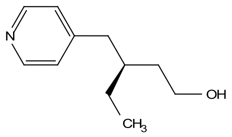


**2c'**

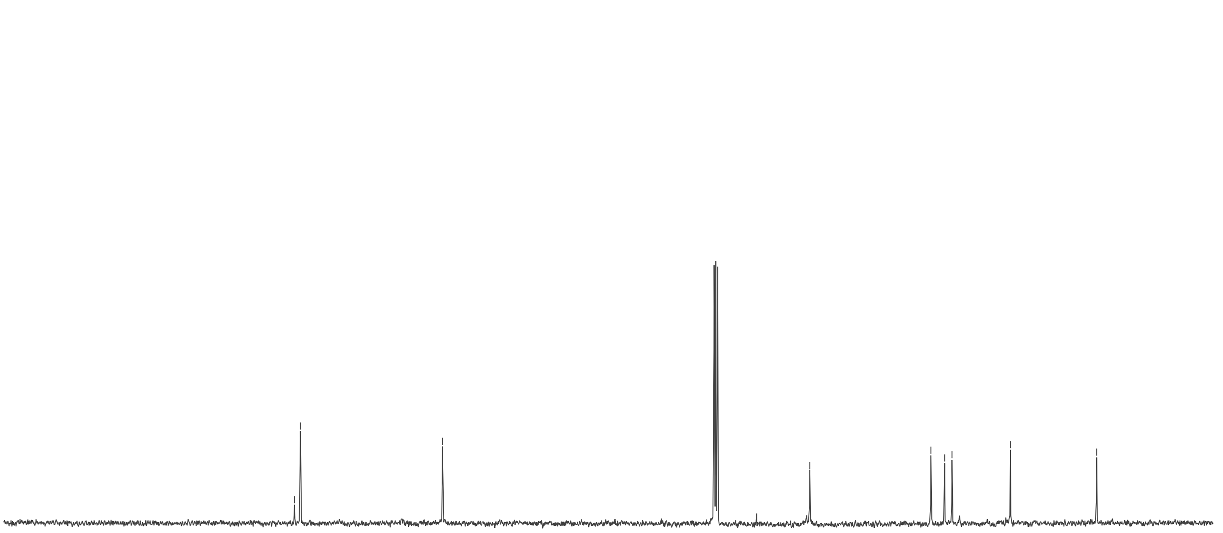


**Supplementary Figure 41** $^{13}$C NMR spectrum of **2c’**

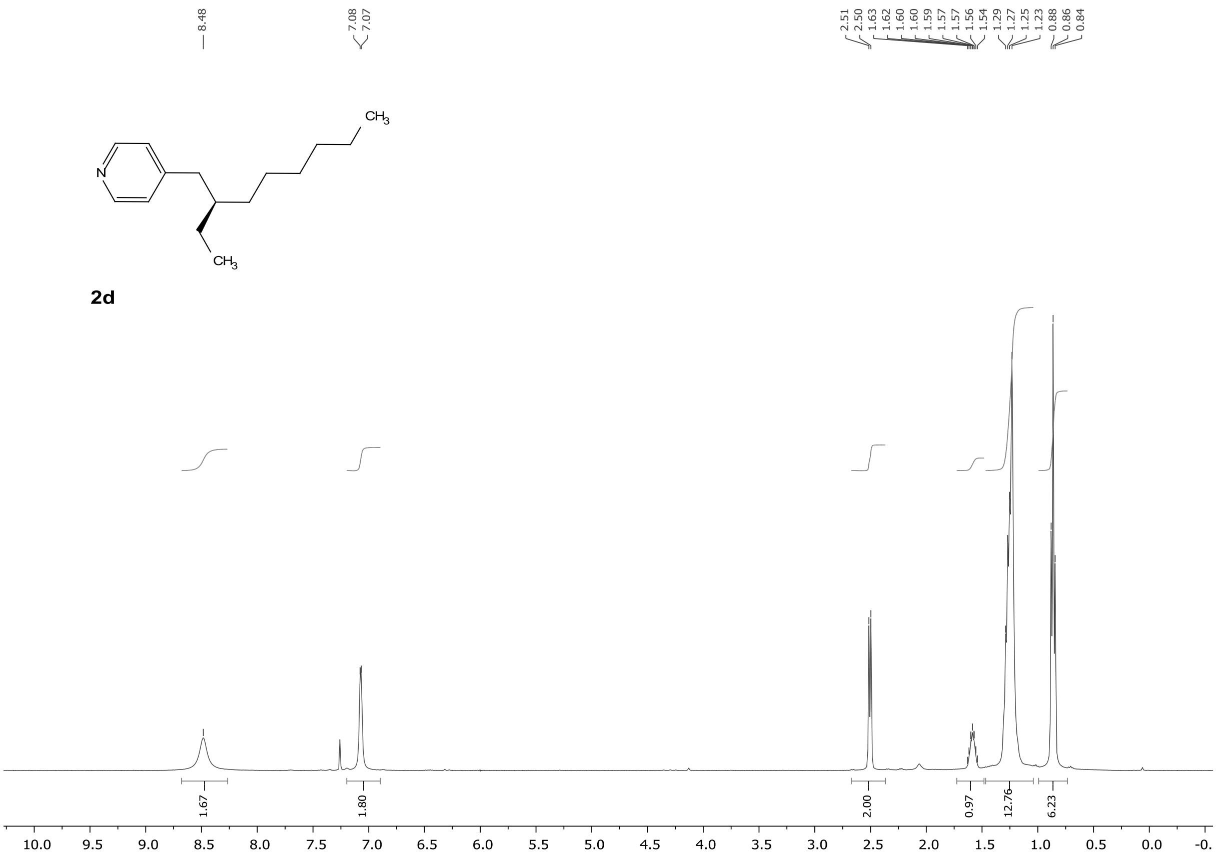


**2d**

**Supplementary Figure 42** $^{1}$H NMR spectrum of **2d**

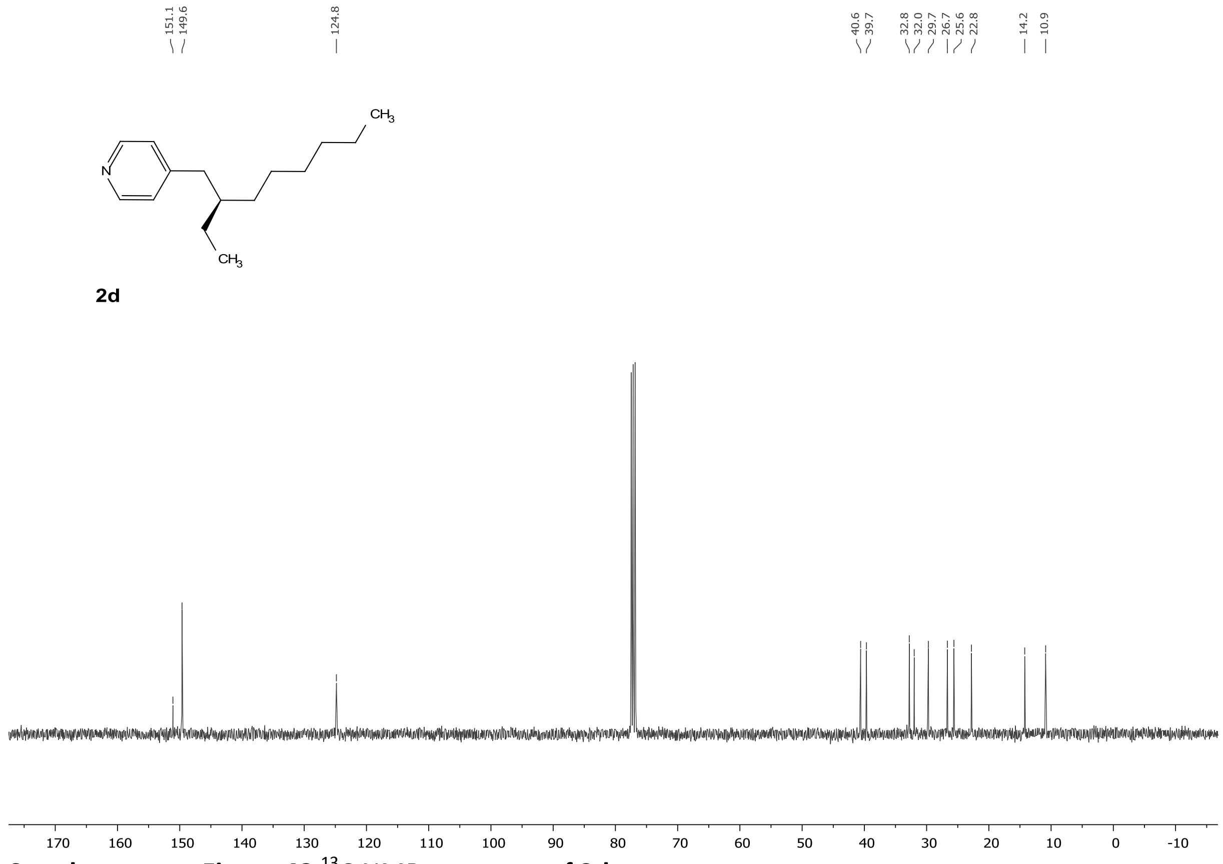


**Supplementary Figure 43** $^{13}$C NMR spectrum of **2d**

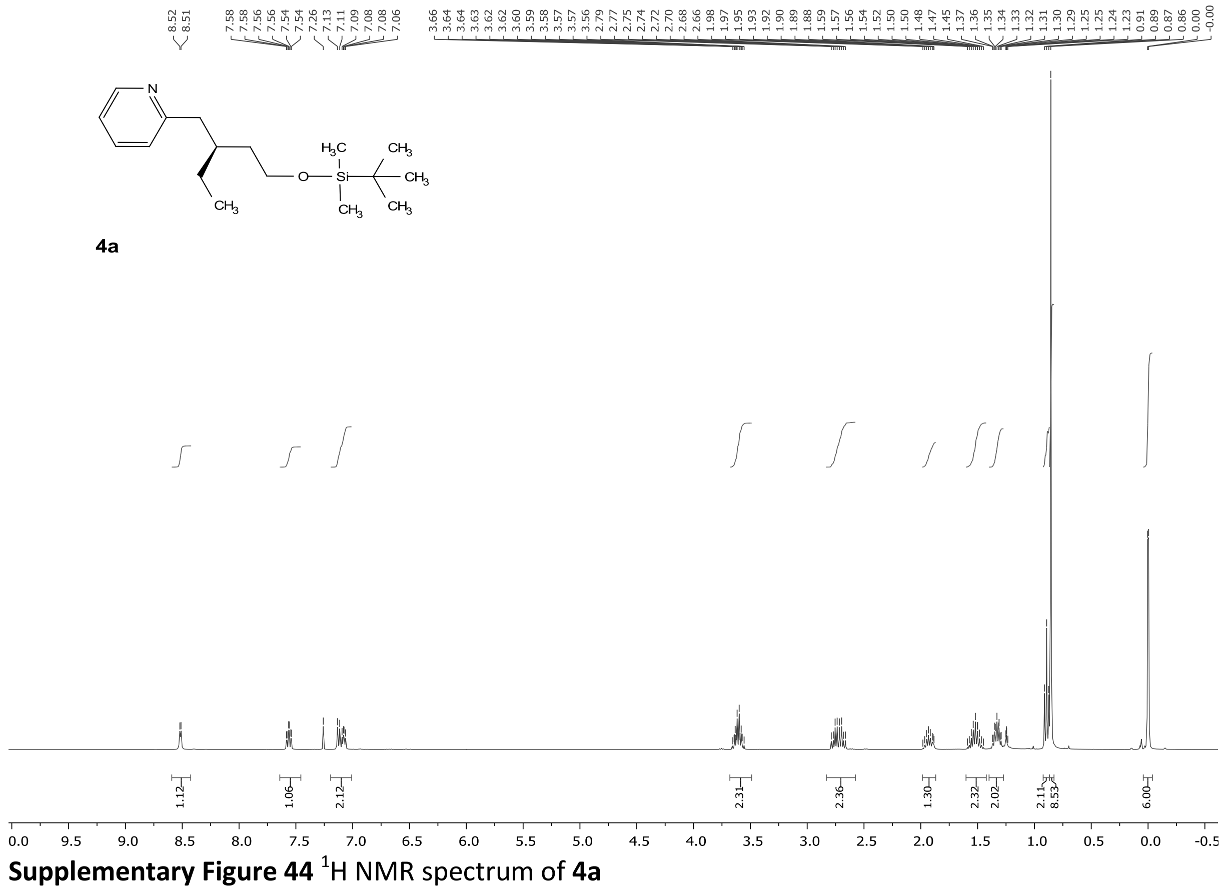


**Supplementary Figure 44** $^{1}$H NMR spectrum of **4a**

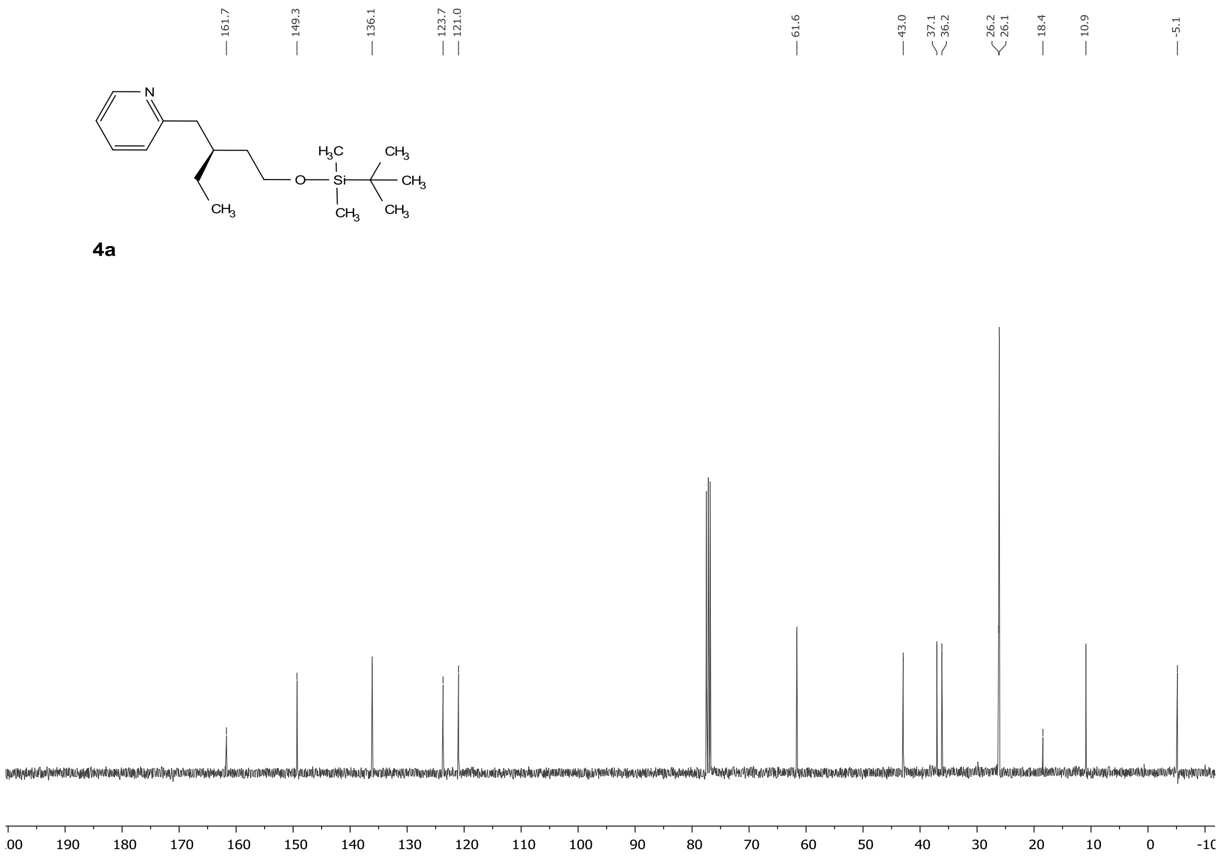

Supplementary Figure 45 $^{13}$C NMR spectrum of **4a**

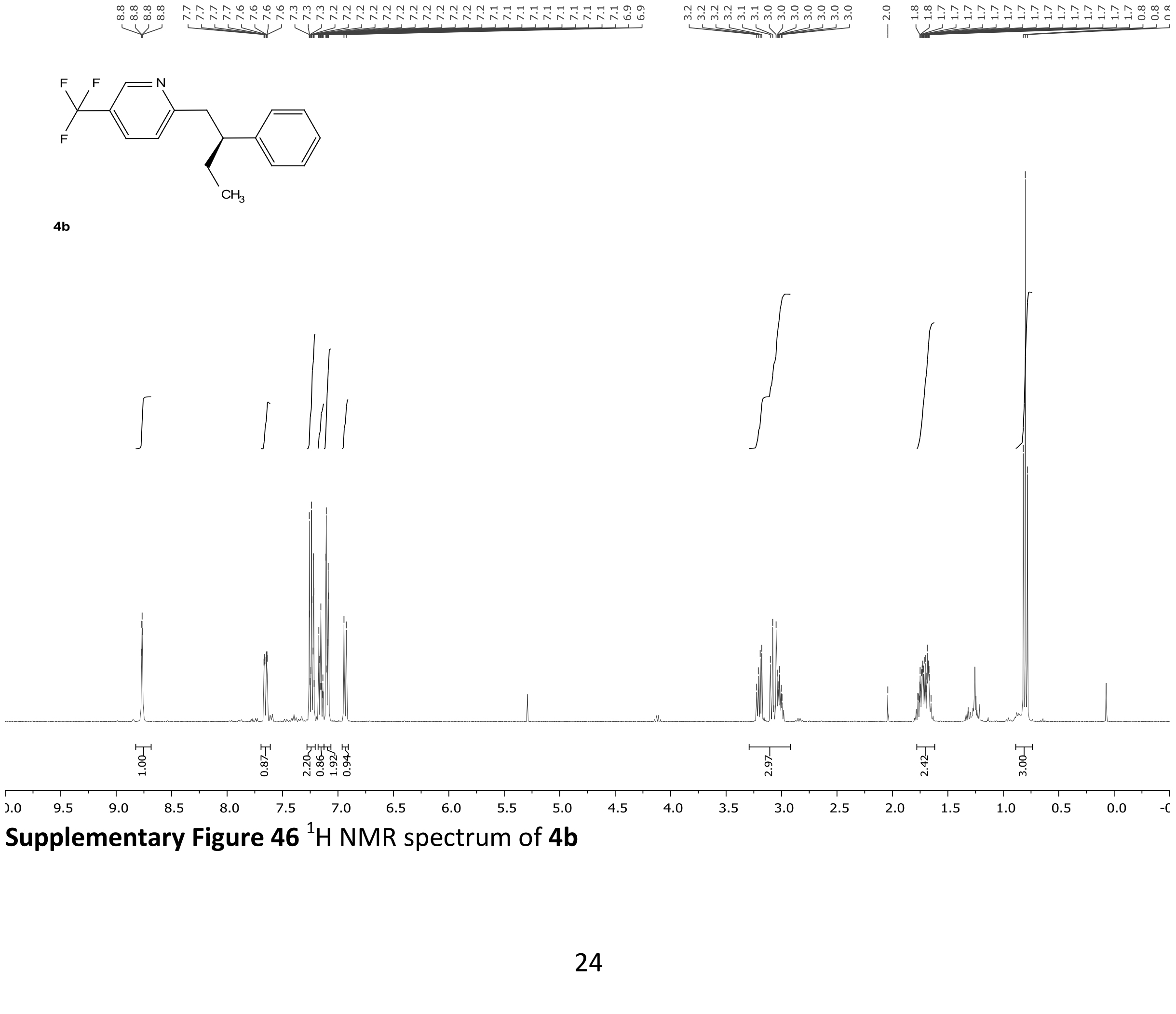

Supplementary Figure 46 $^{1}$H NMR spectrum of **4b**

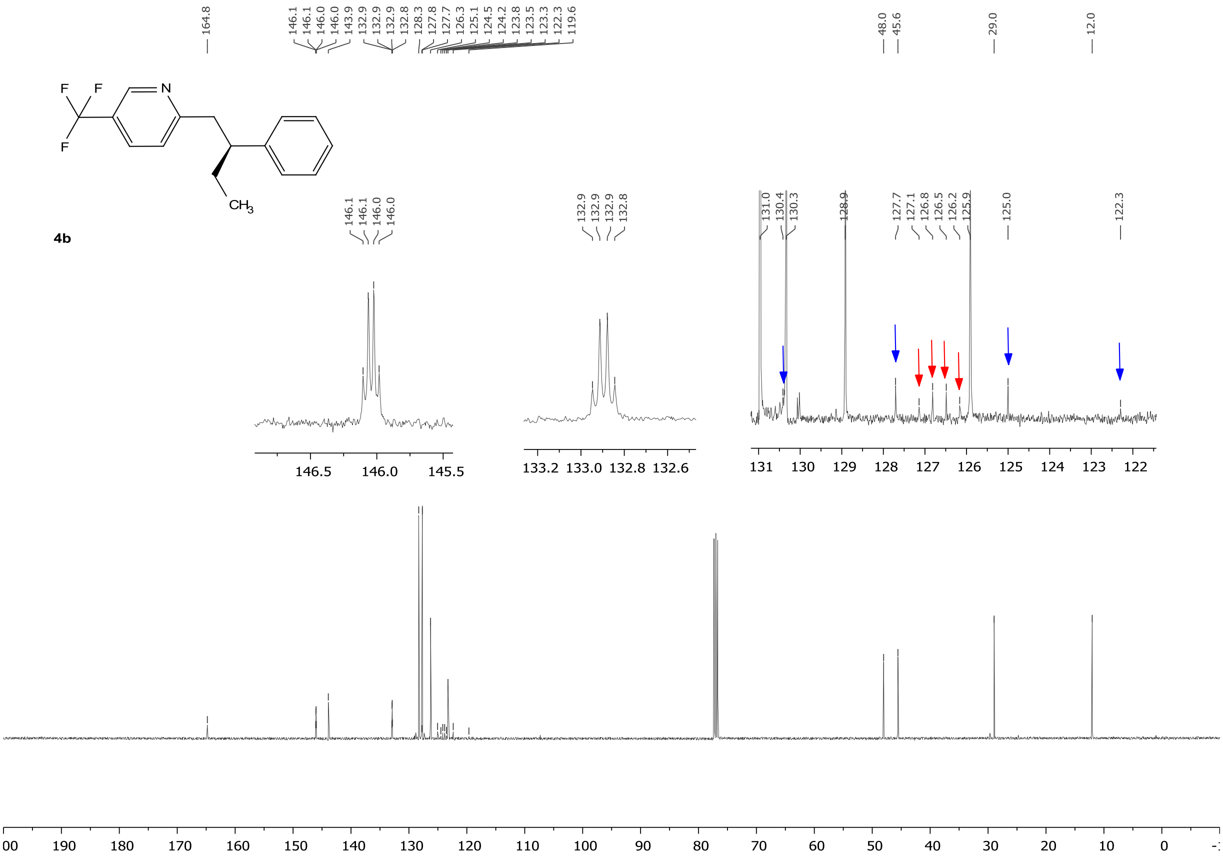

**Supplementary Figure 47** $^{13}$C NMR spectrum of **4b**

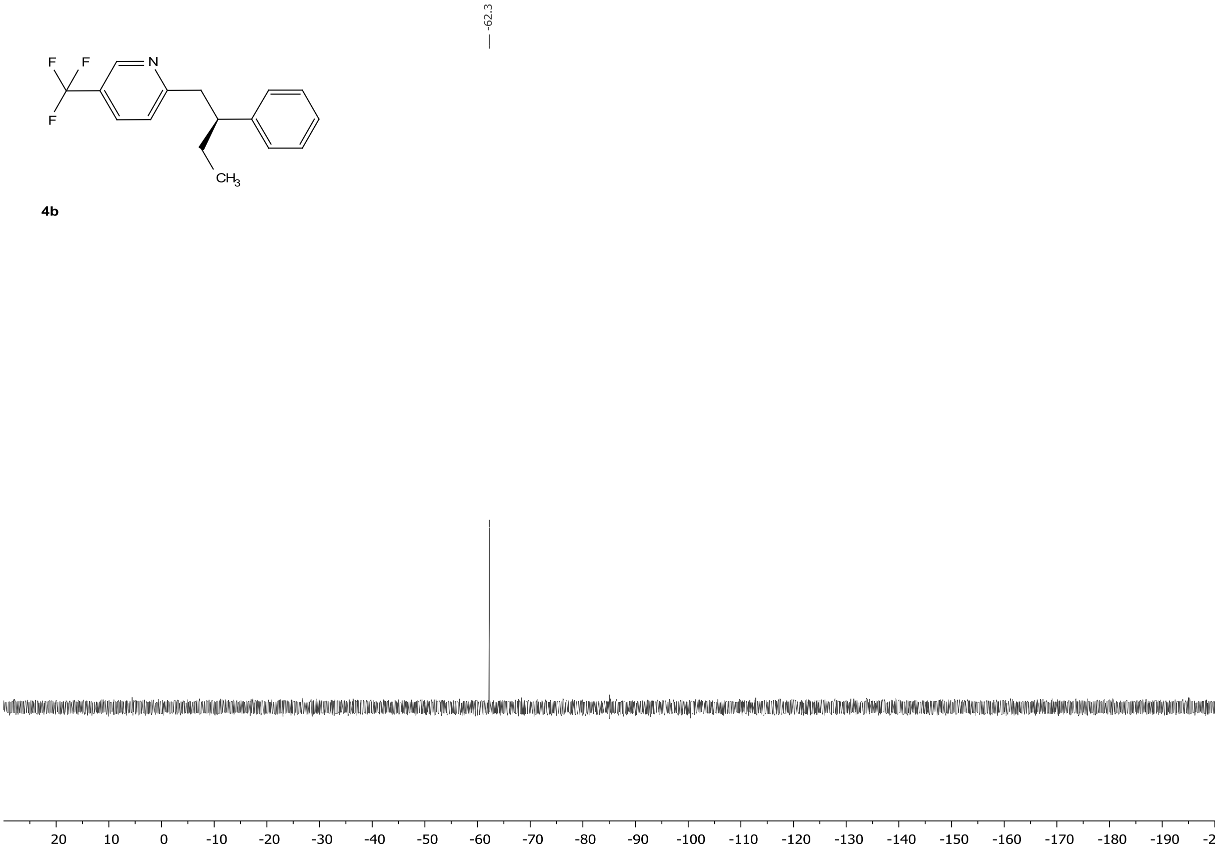

**Supplementary Figure 48** $^{19}$F NMR spectrum of **4b**

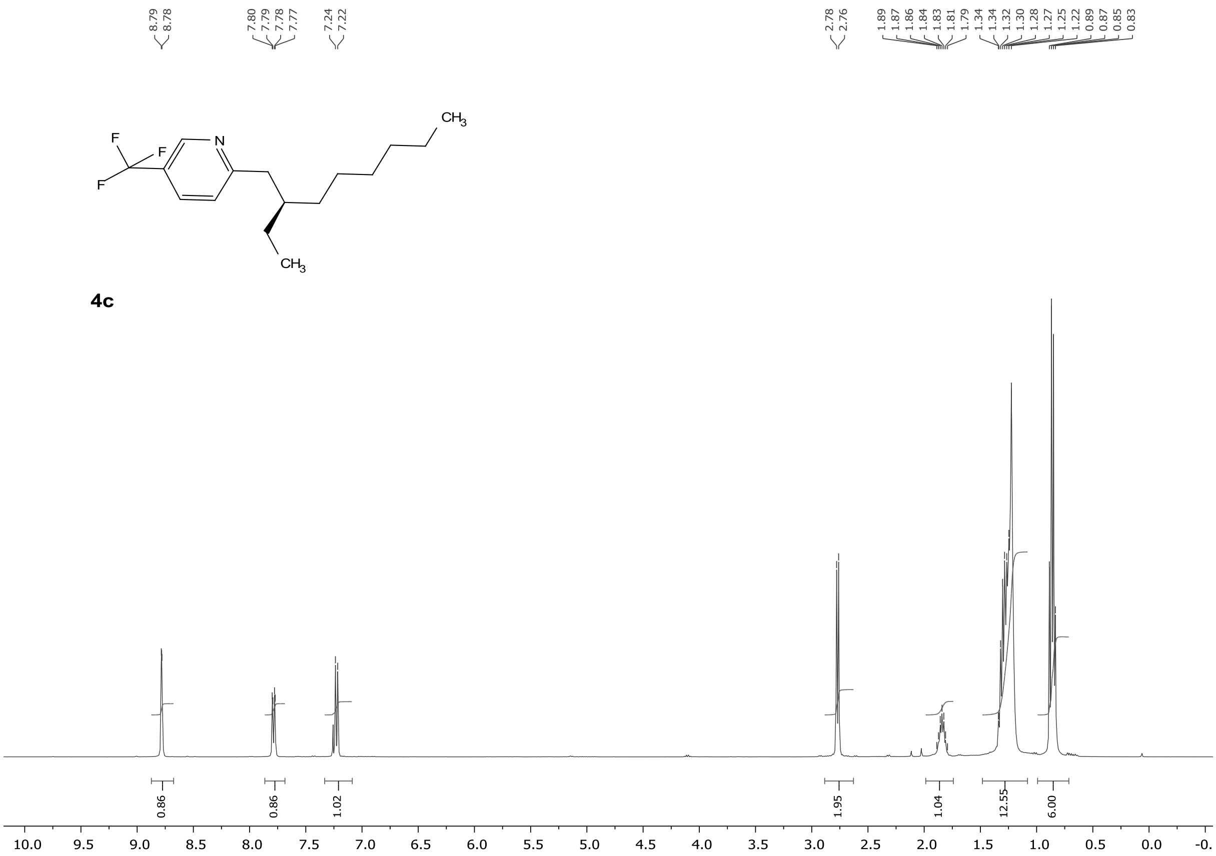

**Supplementary Figure 49** $^{1}$H NMR spectrum of **4c**

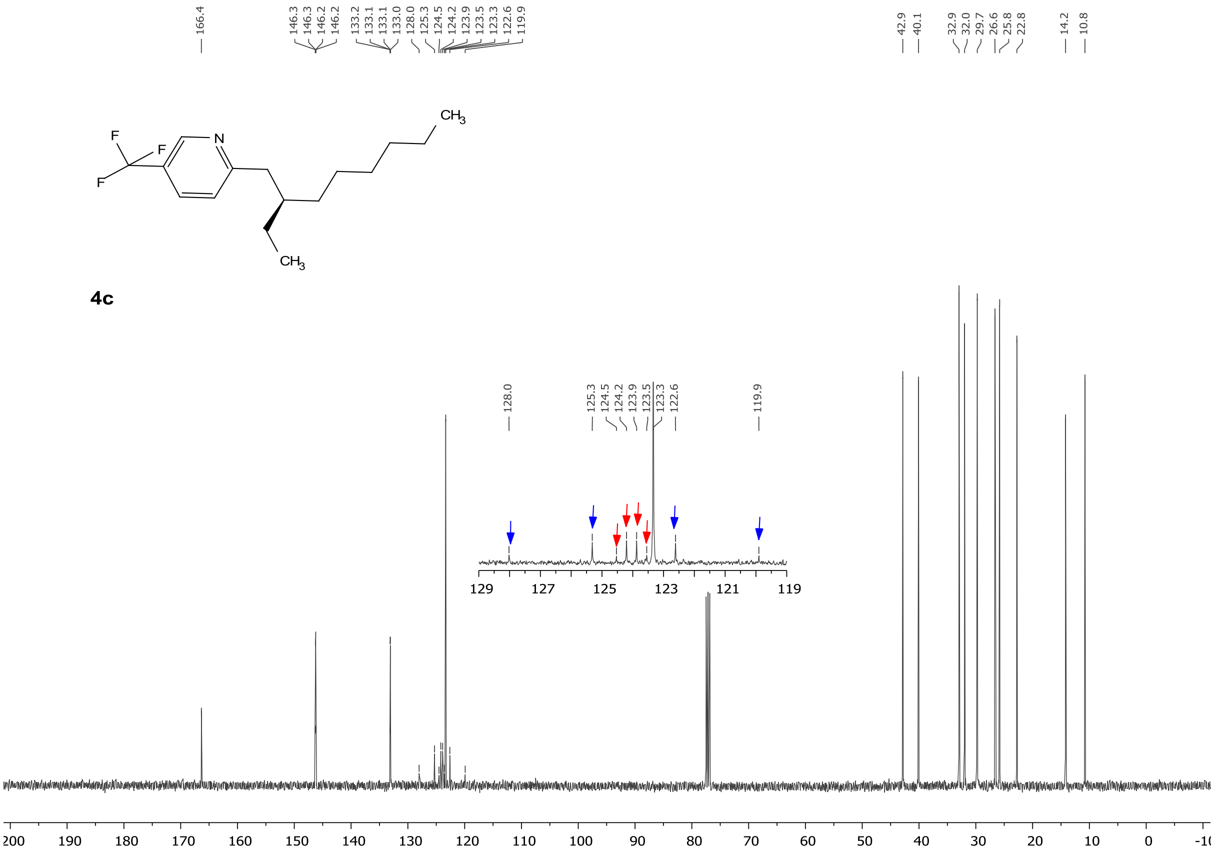

**Supplementary Figure 50** $^{13}$C NMR spectrum of **4c**

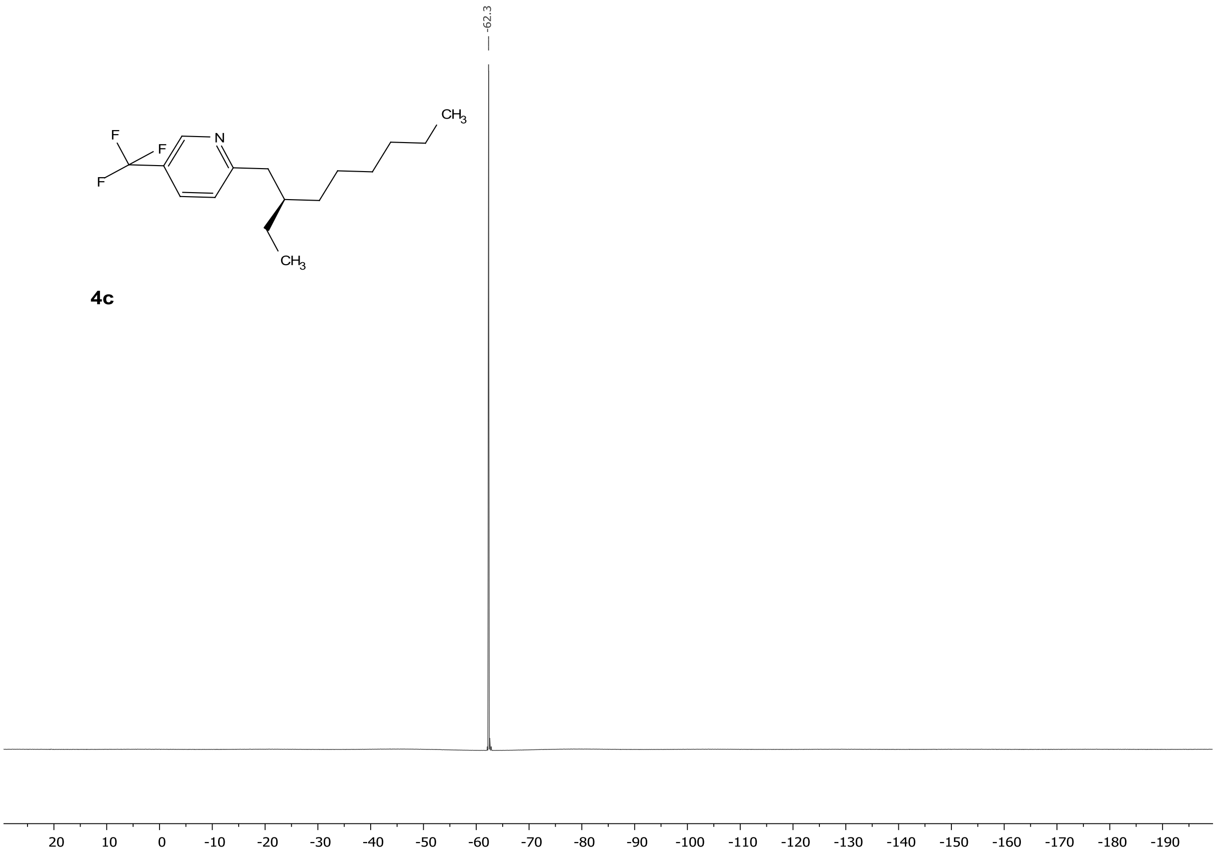

**Supplementary Figure 51** $^{19}$F NMR spectrum of **4c**

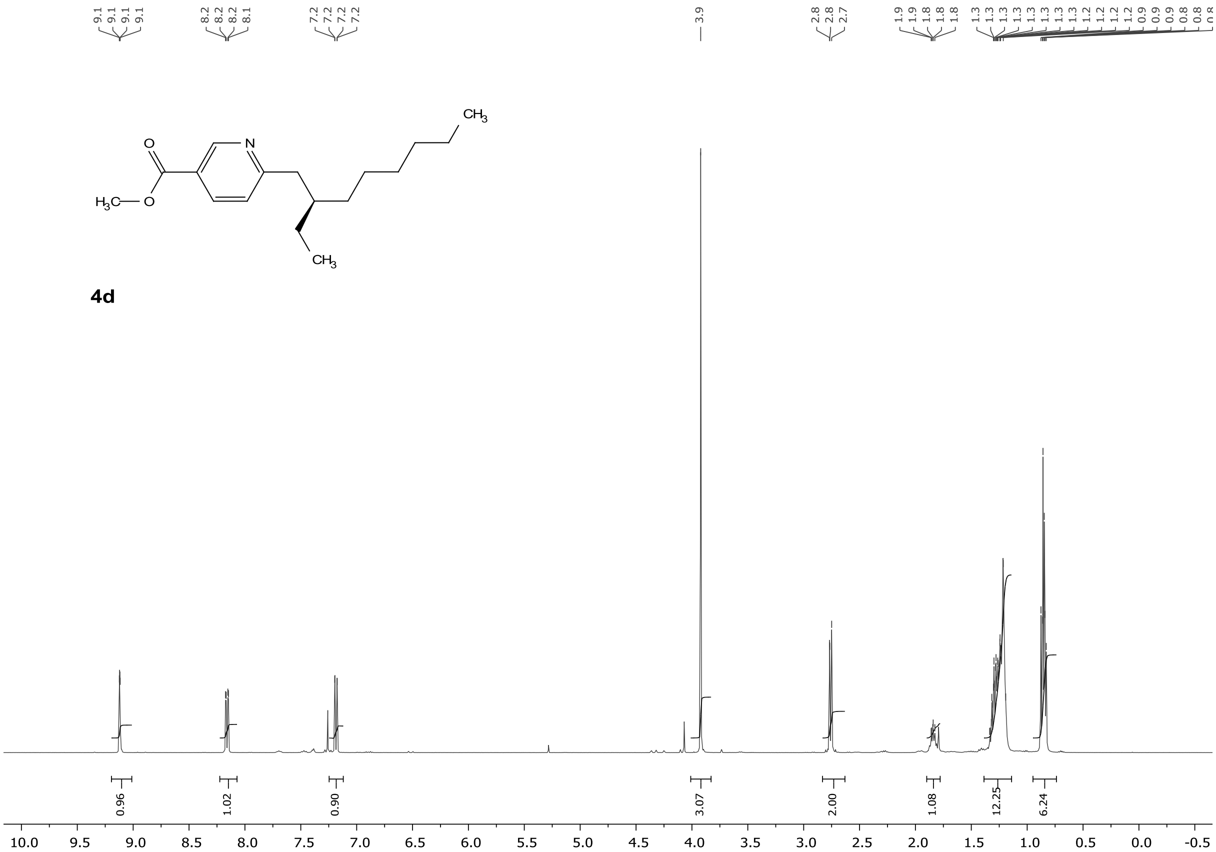

**Supplementary Figure 52** $^{1}$H NMR spectrum of **4d**

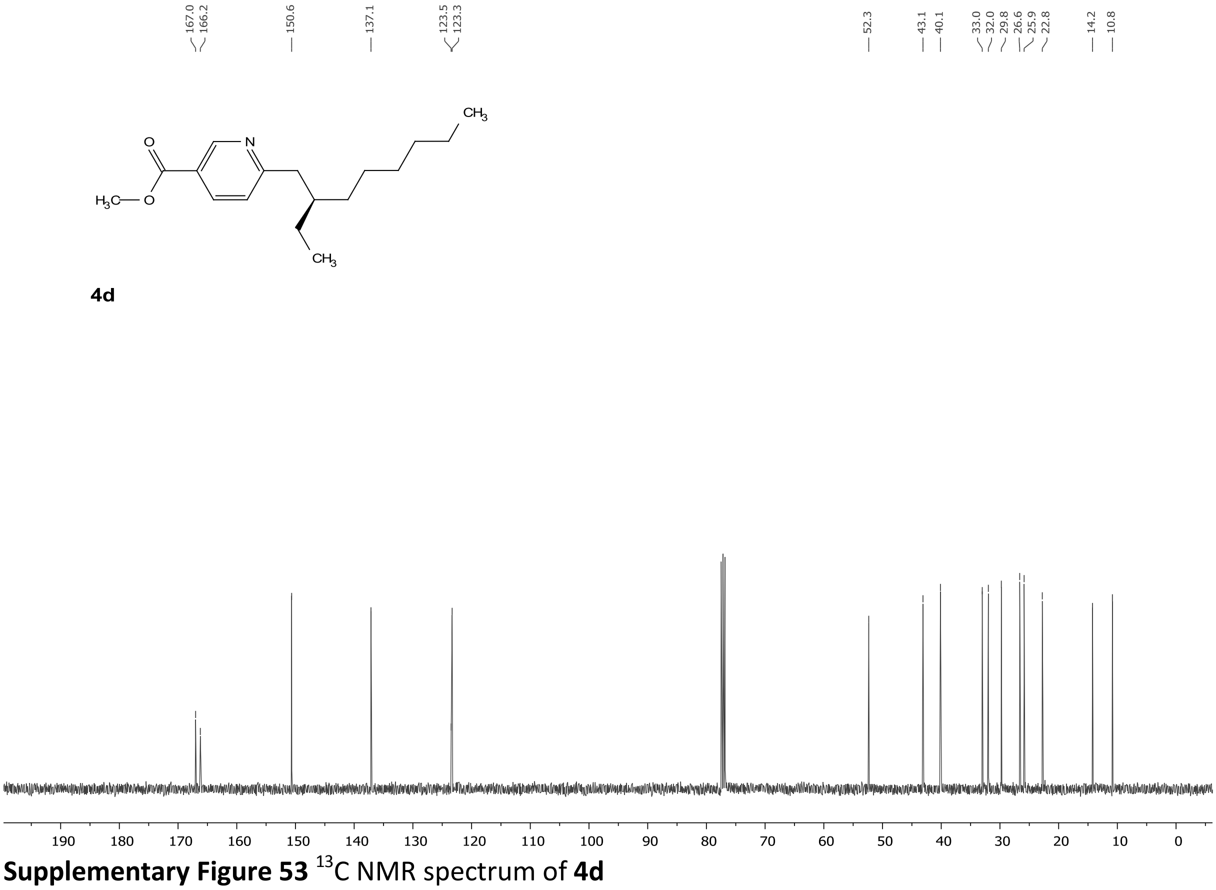

**Supplementary Figure 53** $^{13}$C NMR spectrum of **4d**

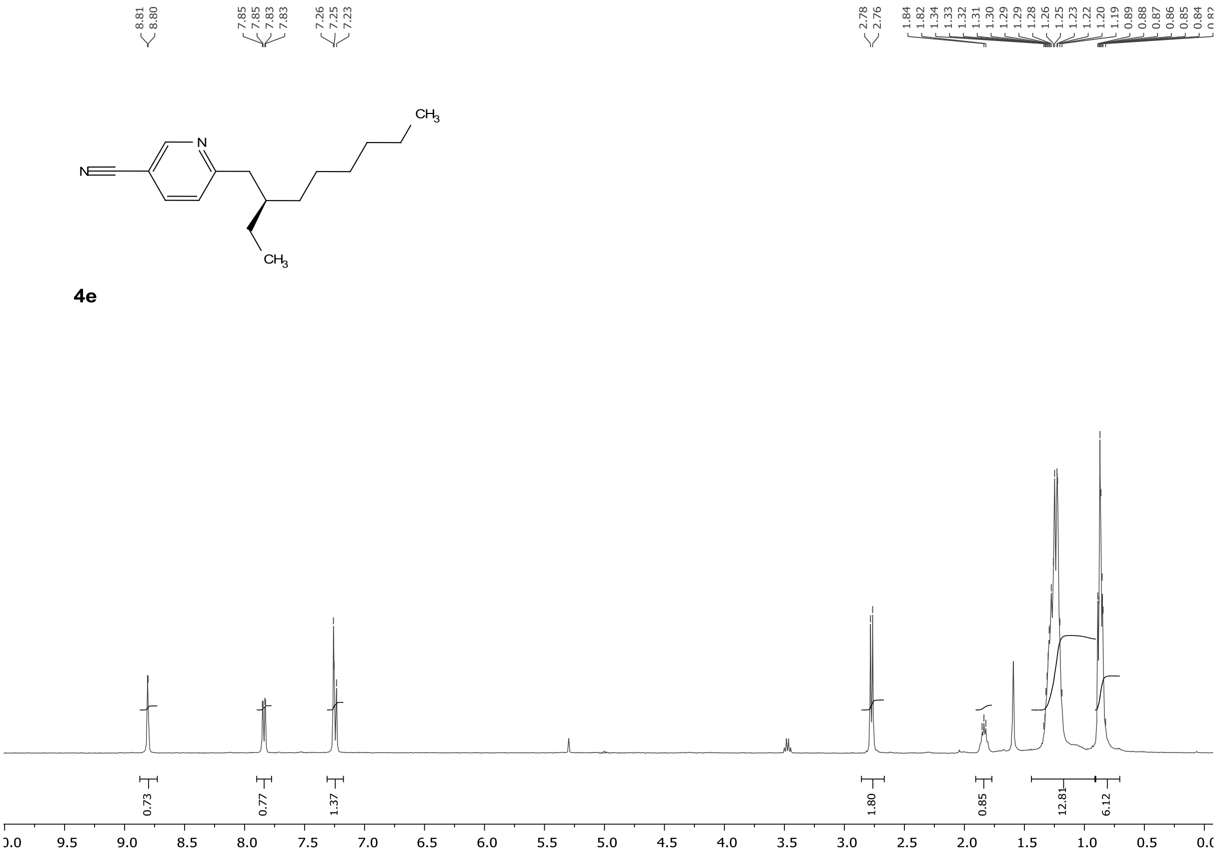


**Supplementary Figure 54** $^{1}H$ NMR spectrum of **4e**

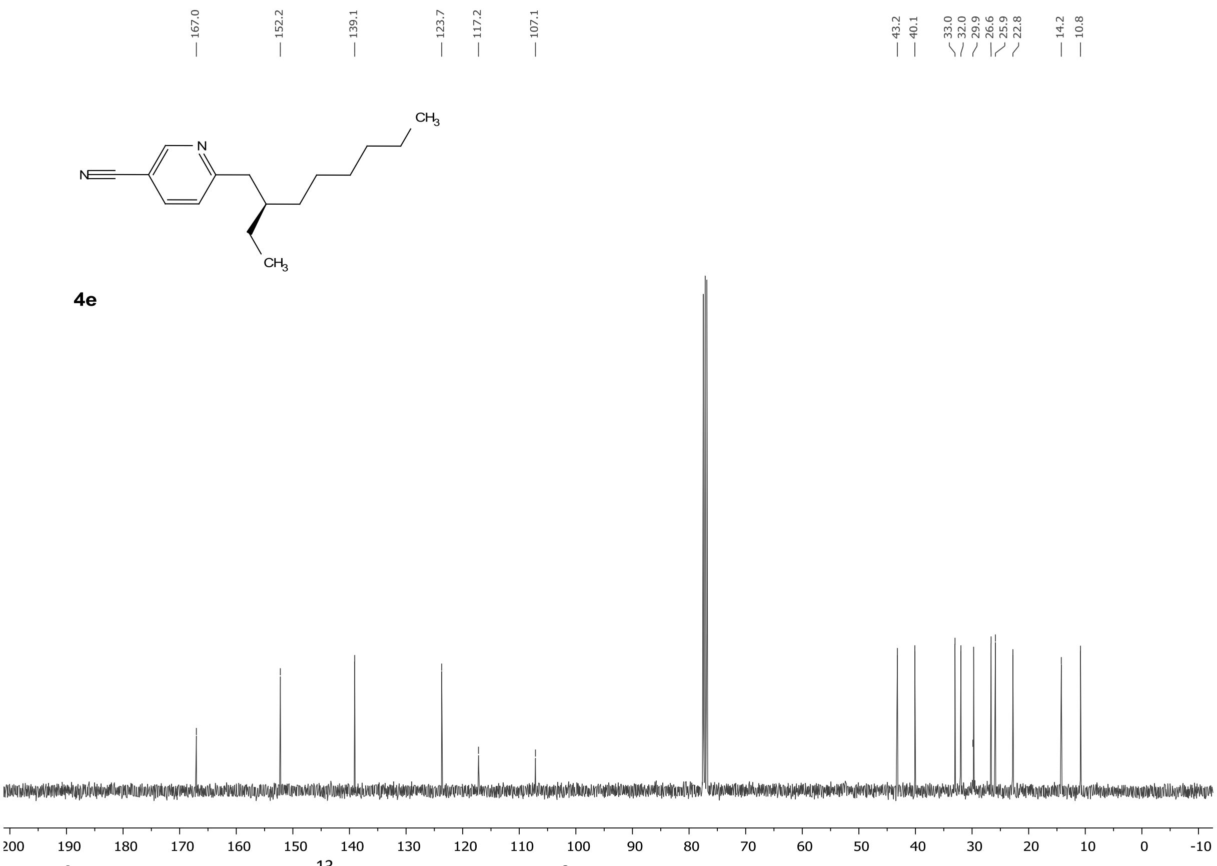


**Supplementary Figure 55** $^{13}C$ NMR spectrum of **4e**

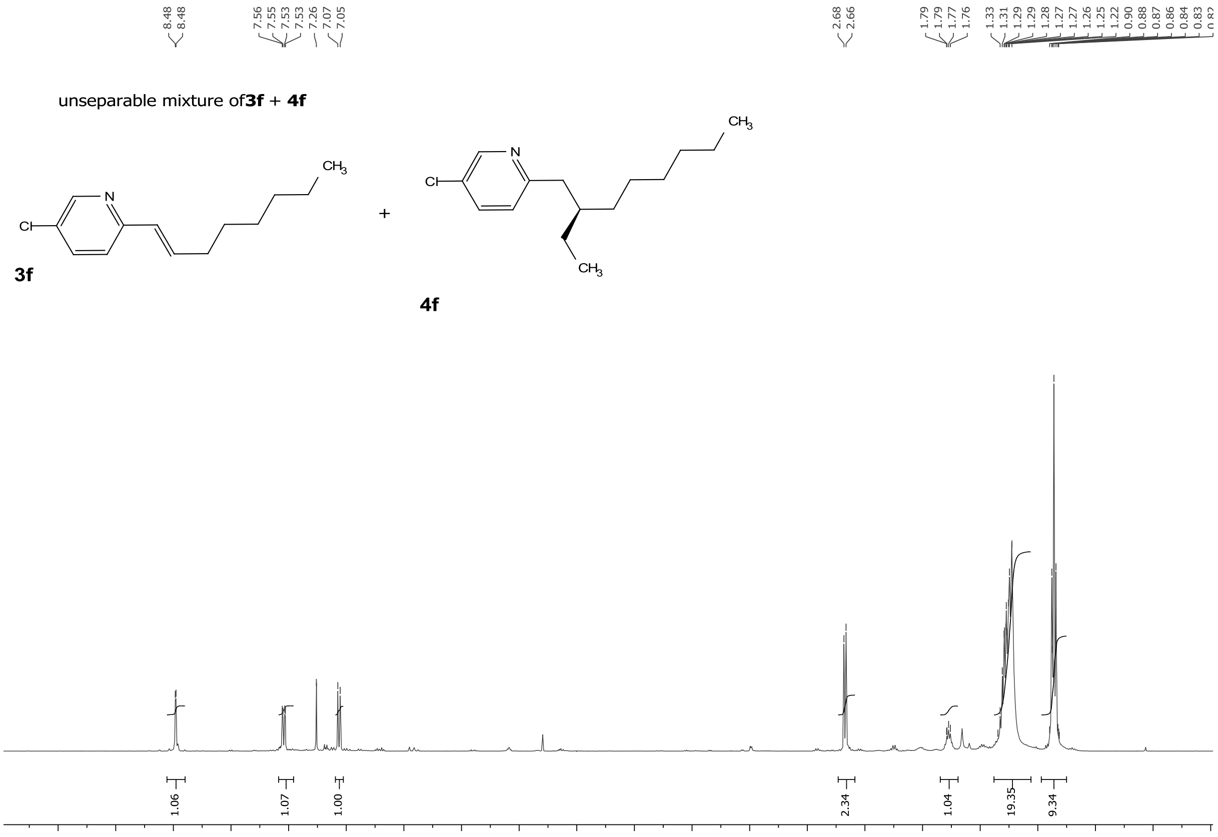


**Supplementary Figure 56** $^{1}$H NMR spectrum of **4f**

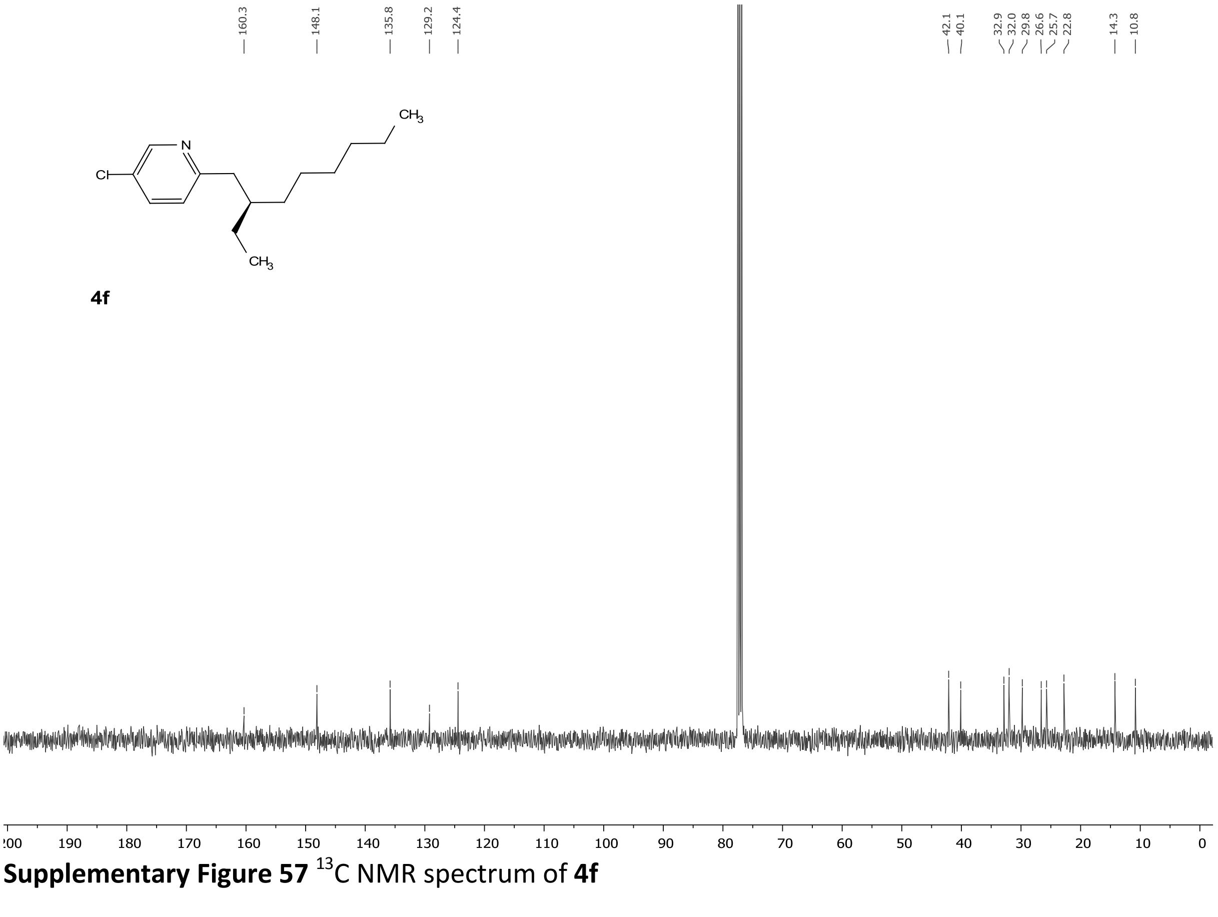


**Supplementary Figure 57** $^{13}$C NMR spectrum of **4f**

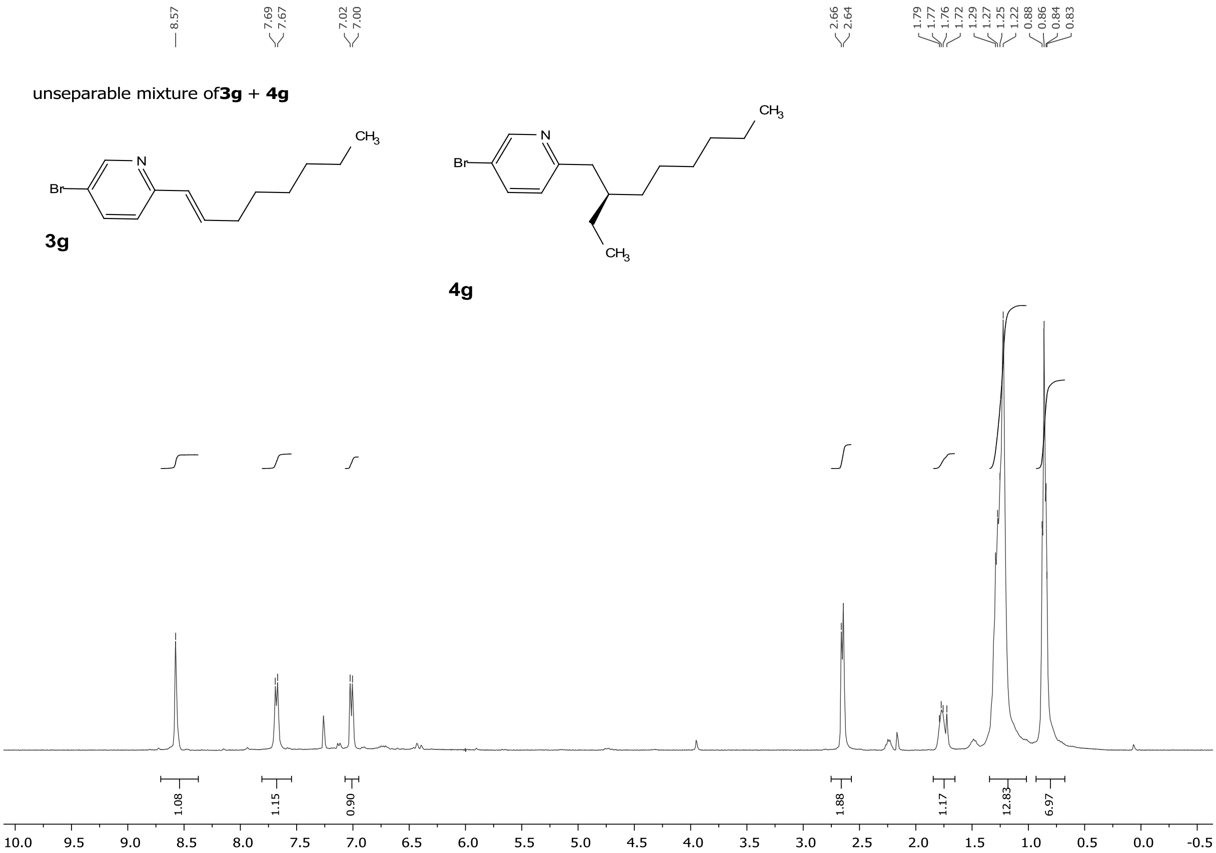


**Supplementary Figure 58** $^{1}$H NMR spectrum of **4g**

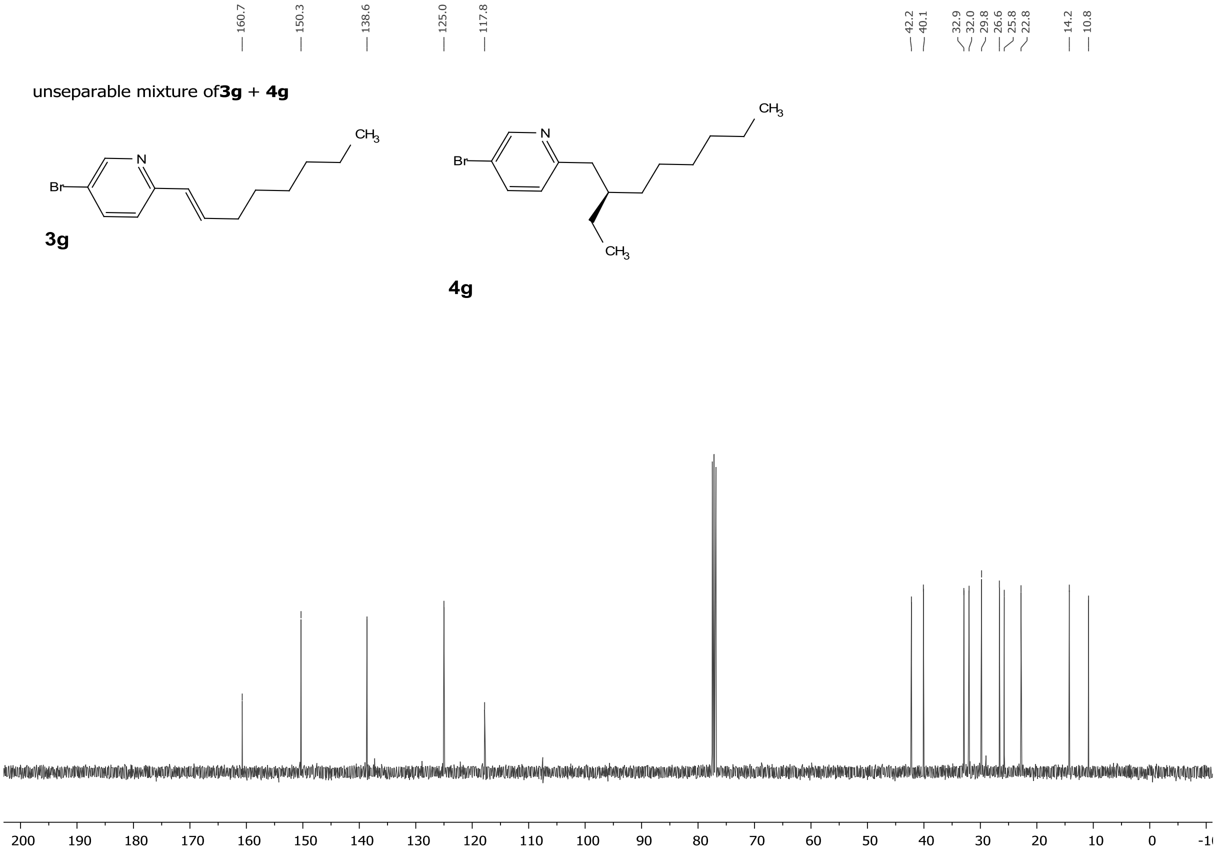


**Supplementary Figure 59** $^{13}$C NMR spectrum of **4g**

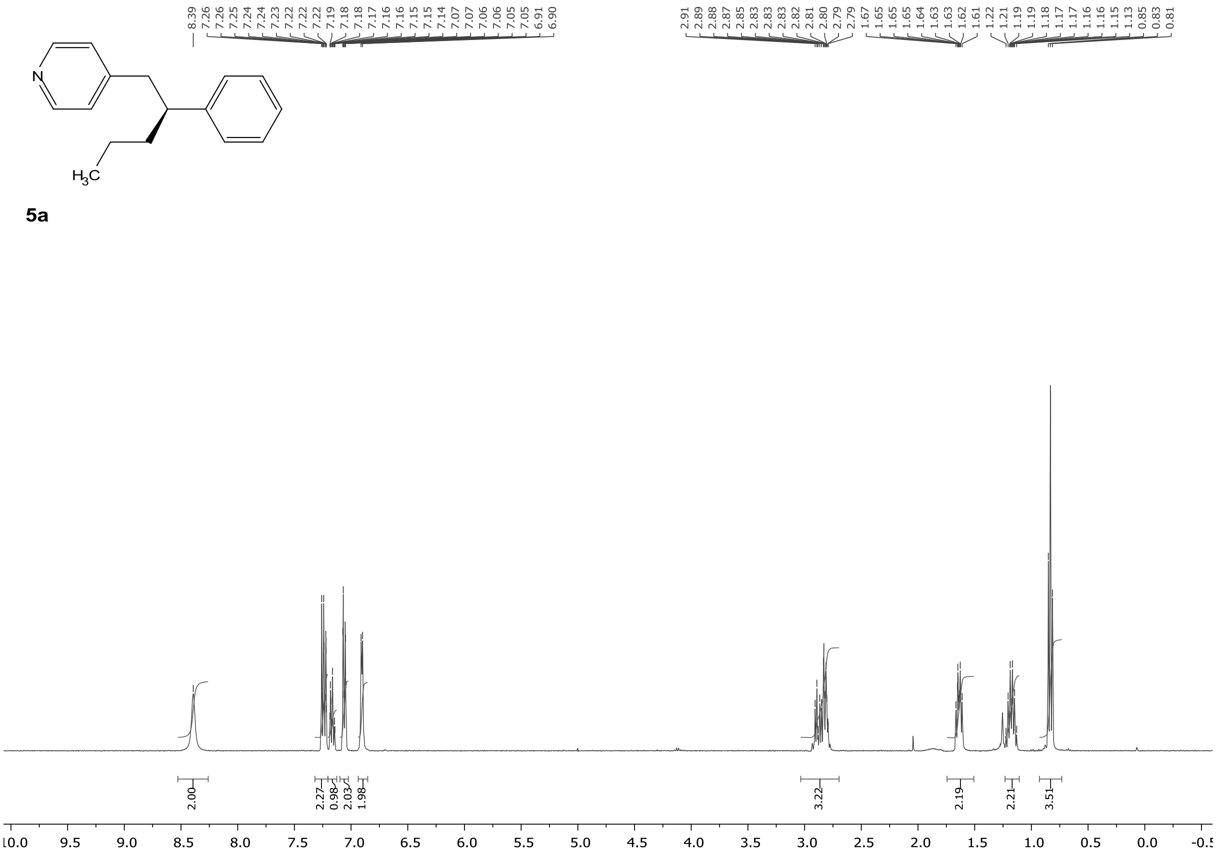

**Supplementary Figure 60** $^{1}$H NMR spectrum of **5a**

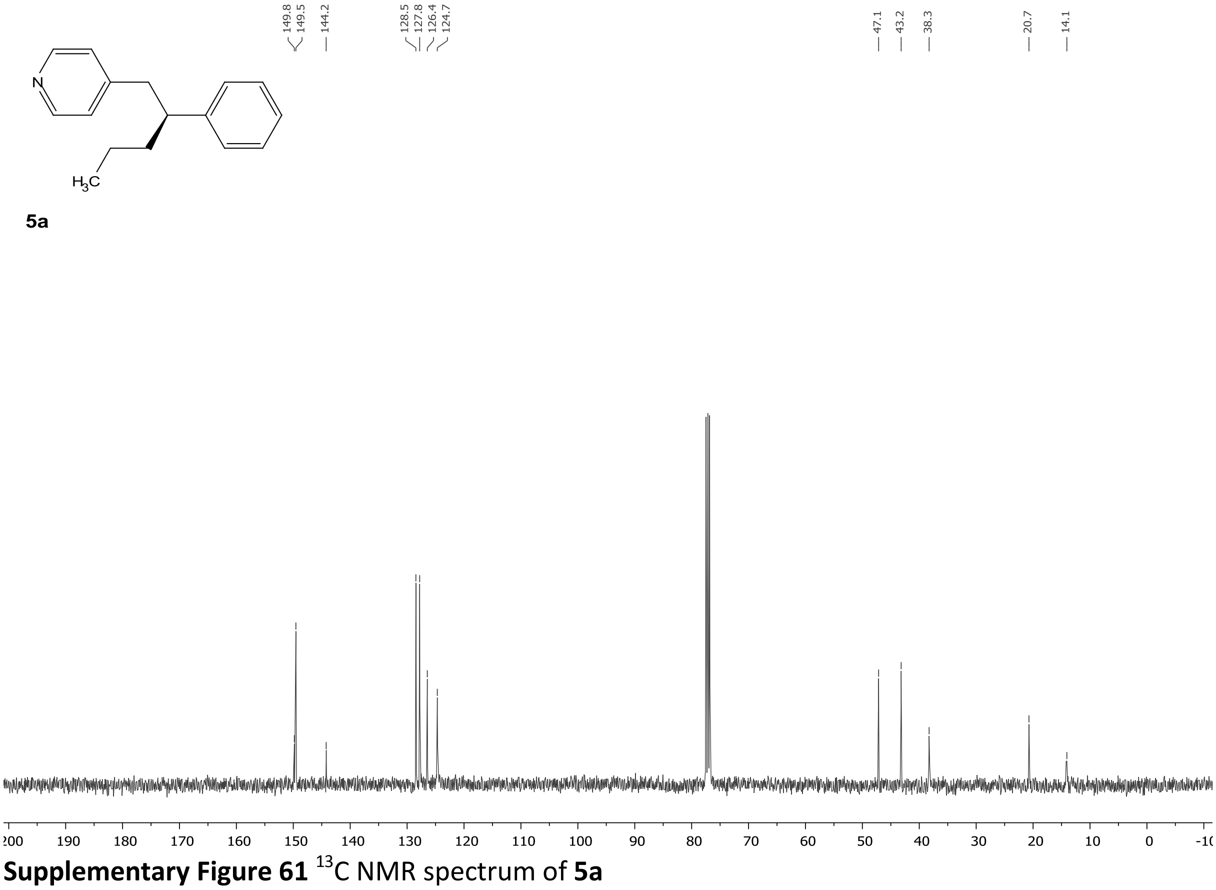

**Supplementary Figure 61** $^{13}$C NMR spectrum of **5a**

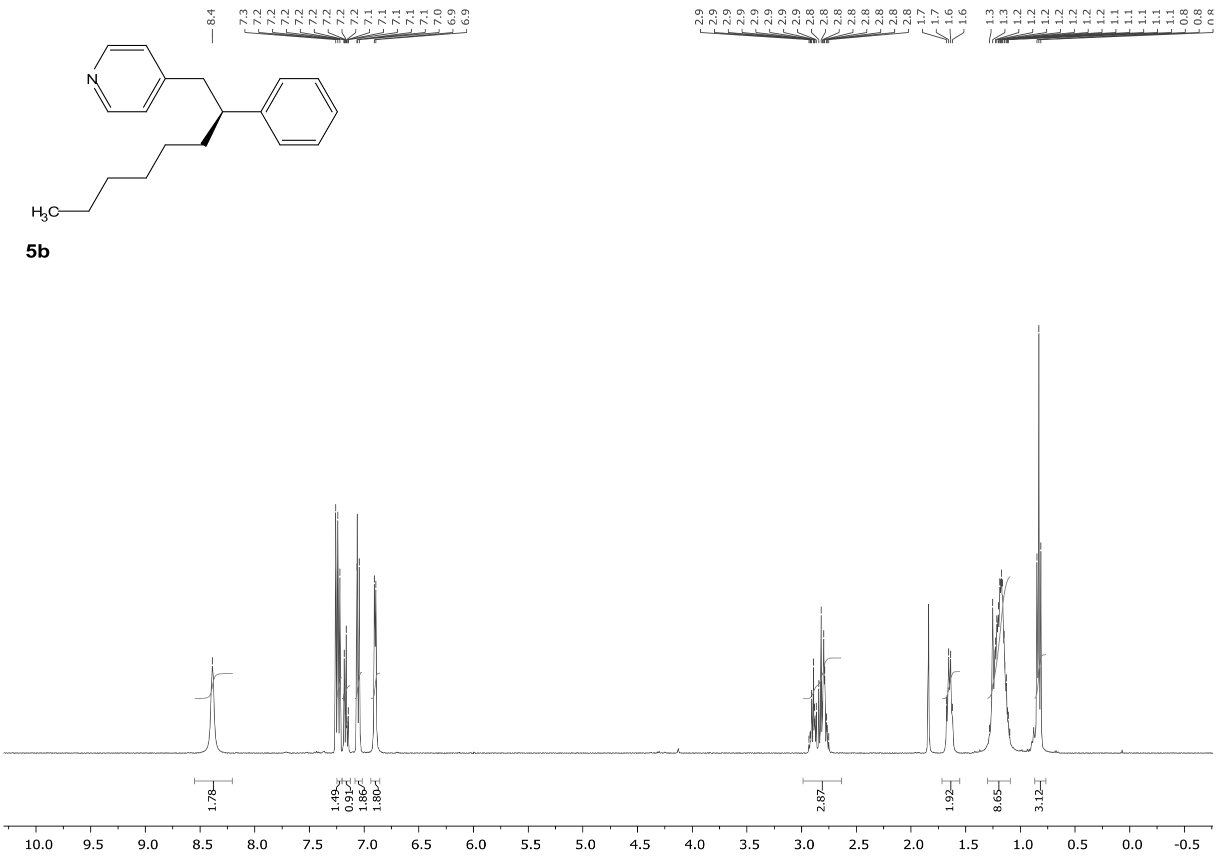


**Supplementary Figure 62** $^{1}$H NMR spectrum of 5b

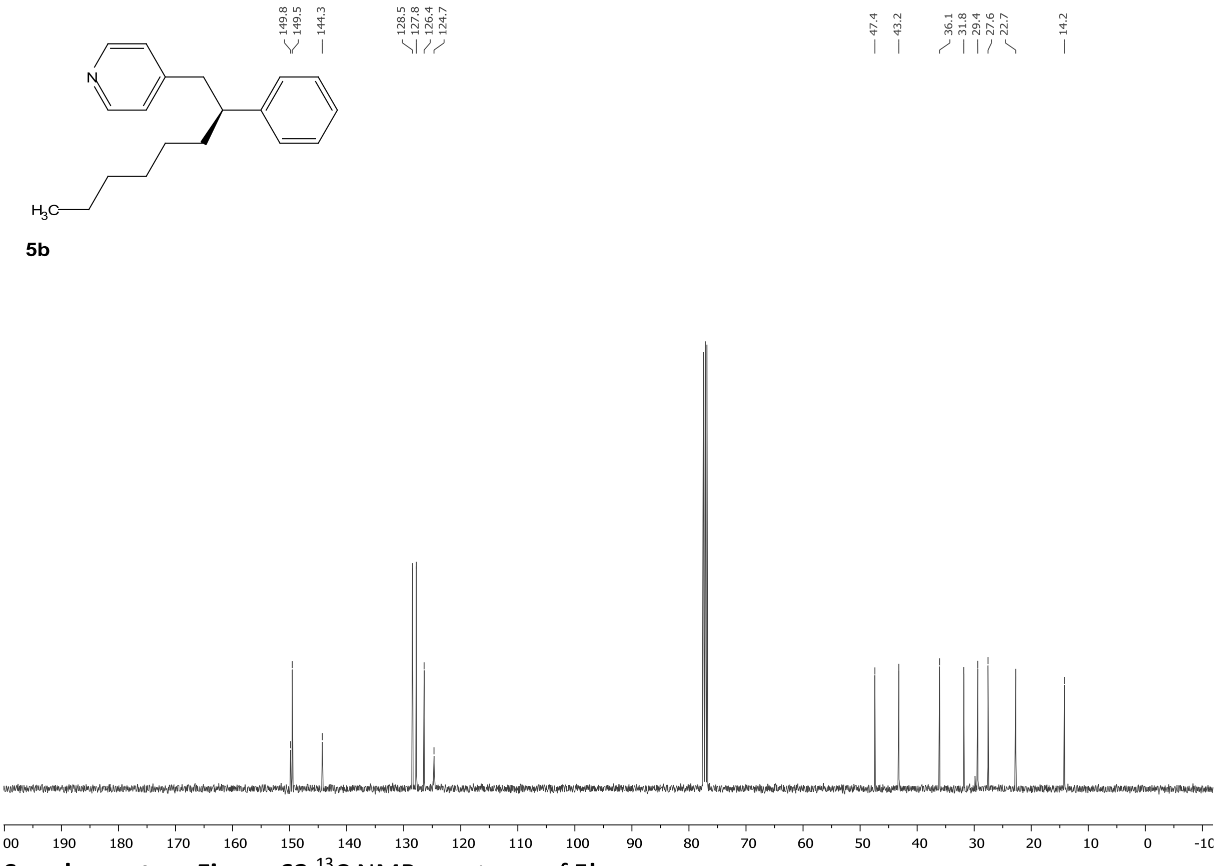


**Supplementary Figure 63** $^{13}$C NMR spectrum of **5b**

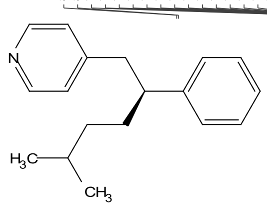

**5c**

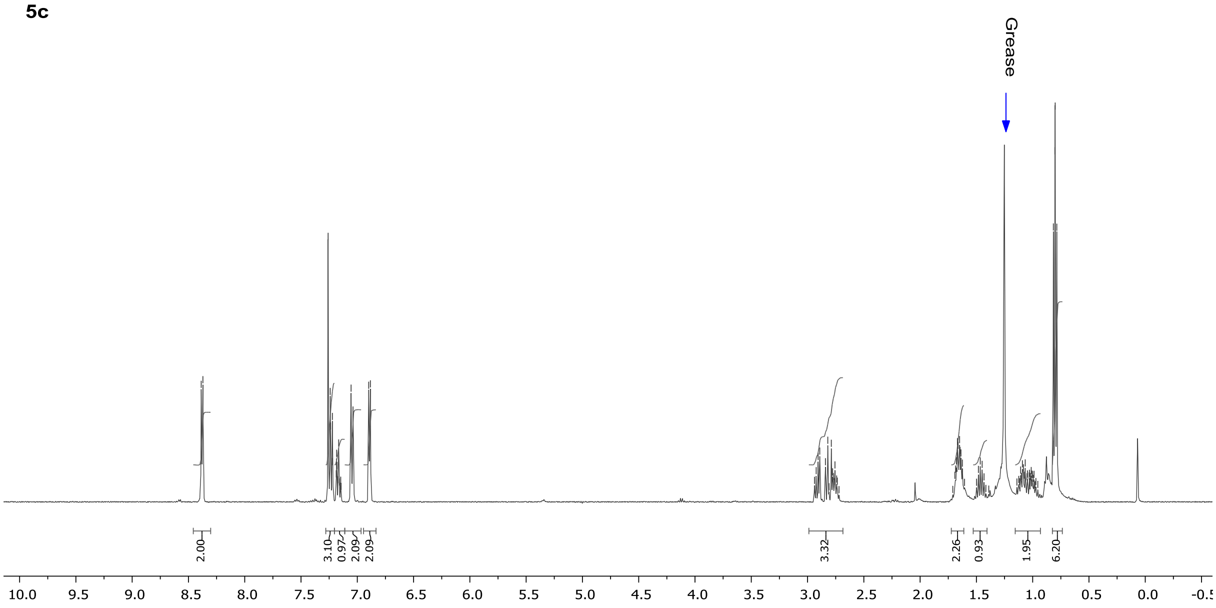


**Supplementary Figure 64** $^{1}$H NMR spectrum of **5c**

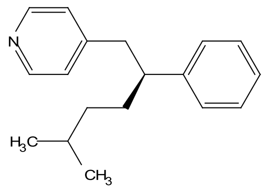

**5c**

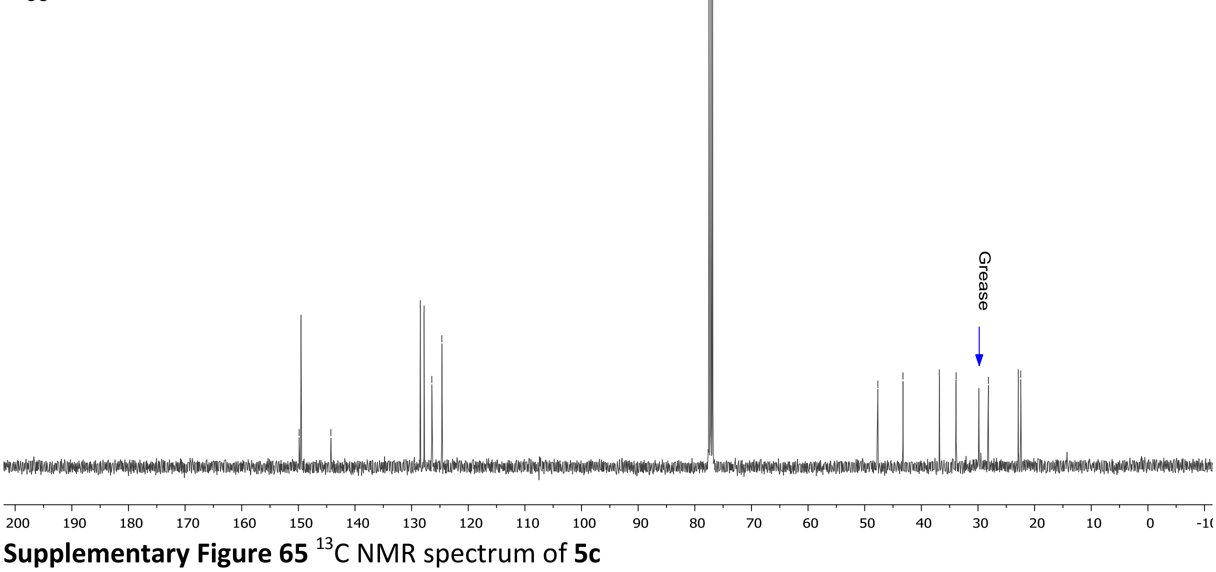


**Supplementary Figure 65** $^{13}$C NMR spectrum of **5c**

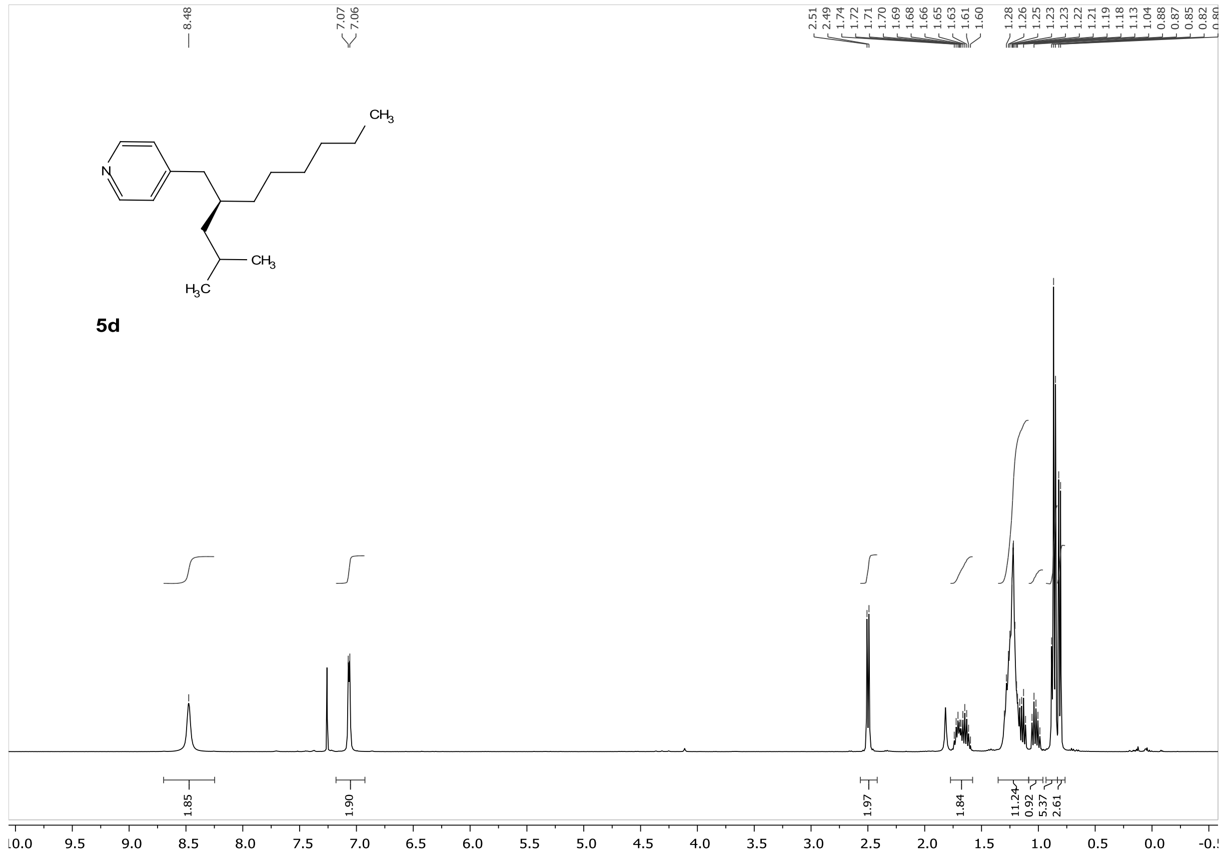

**Supplementary Figure 66** $^{1}$H NMR spectrum of **5d**

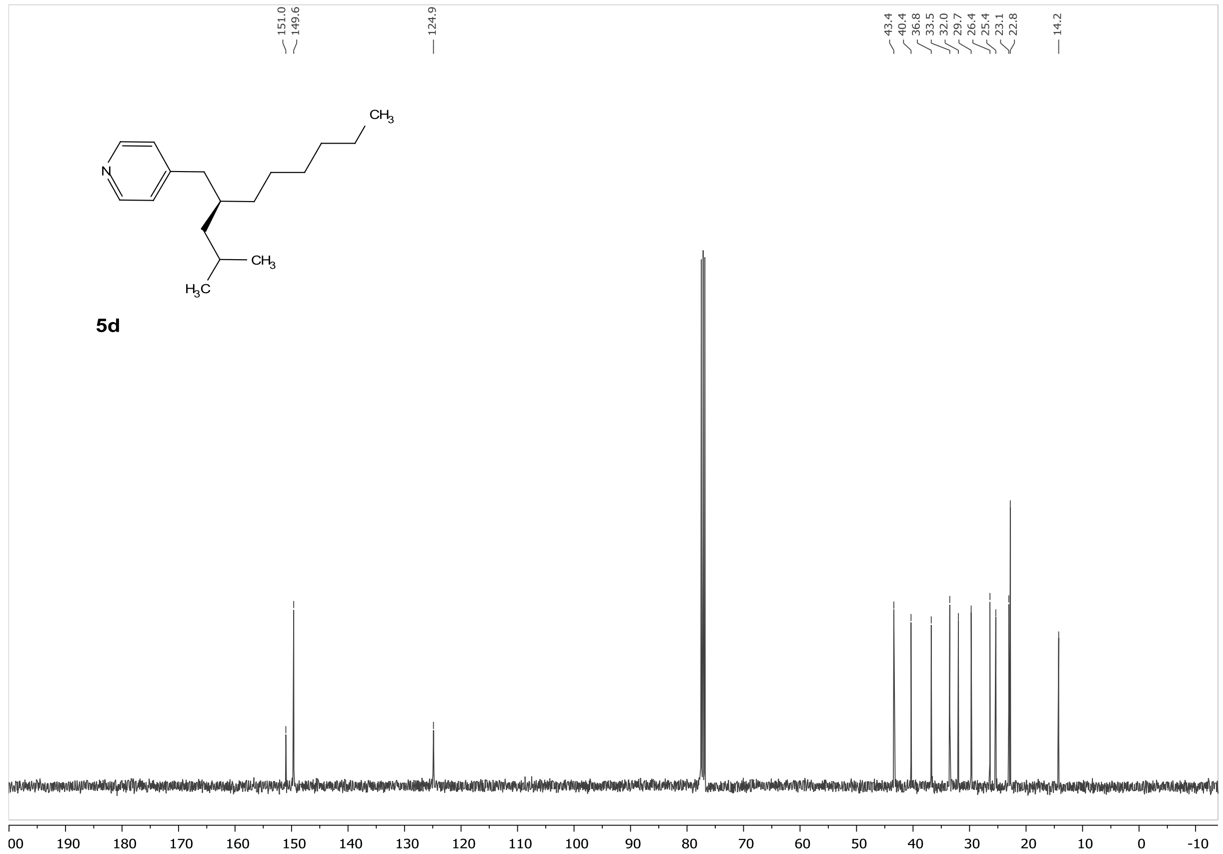

**Supplementary Figure 67** $^{13}$C NMR spectrum of **5d**

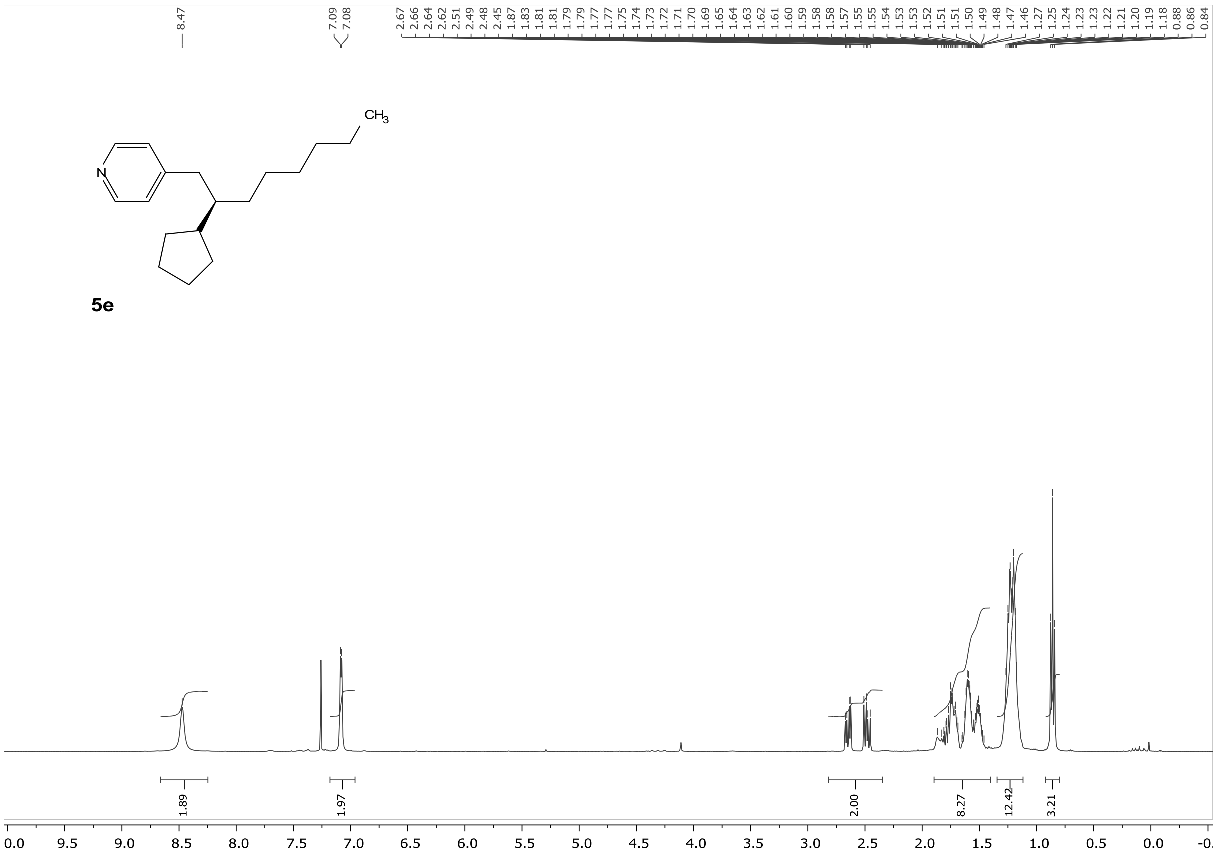

**Supplementary Figure 68** $^{1}$H NMR spectrum of **5e**

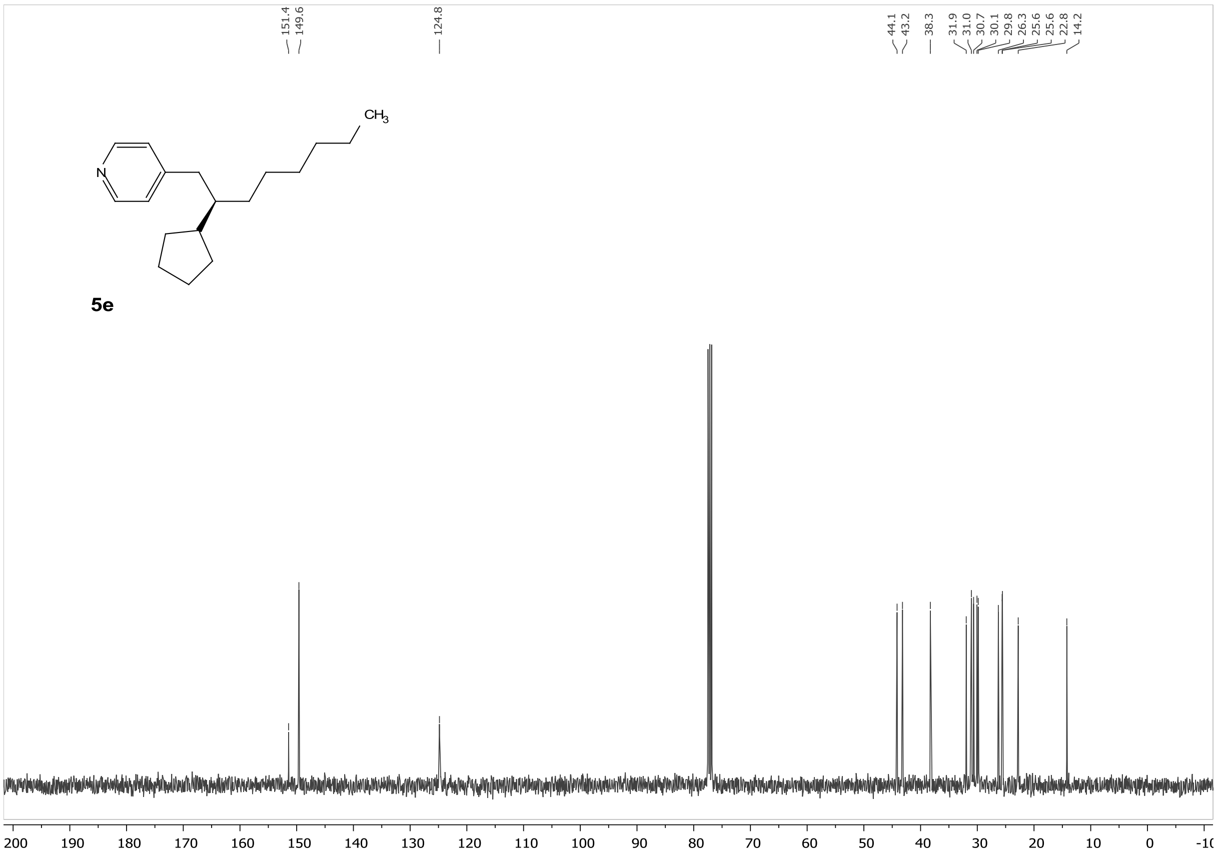

**Supplementary Figure 69** $^{13}$C NMR spectrum of **5e**

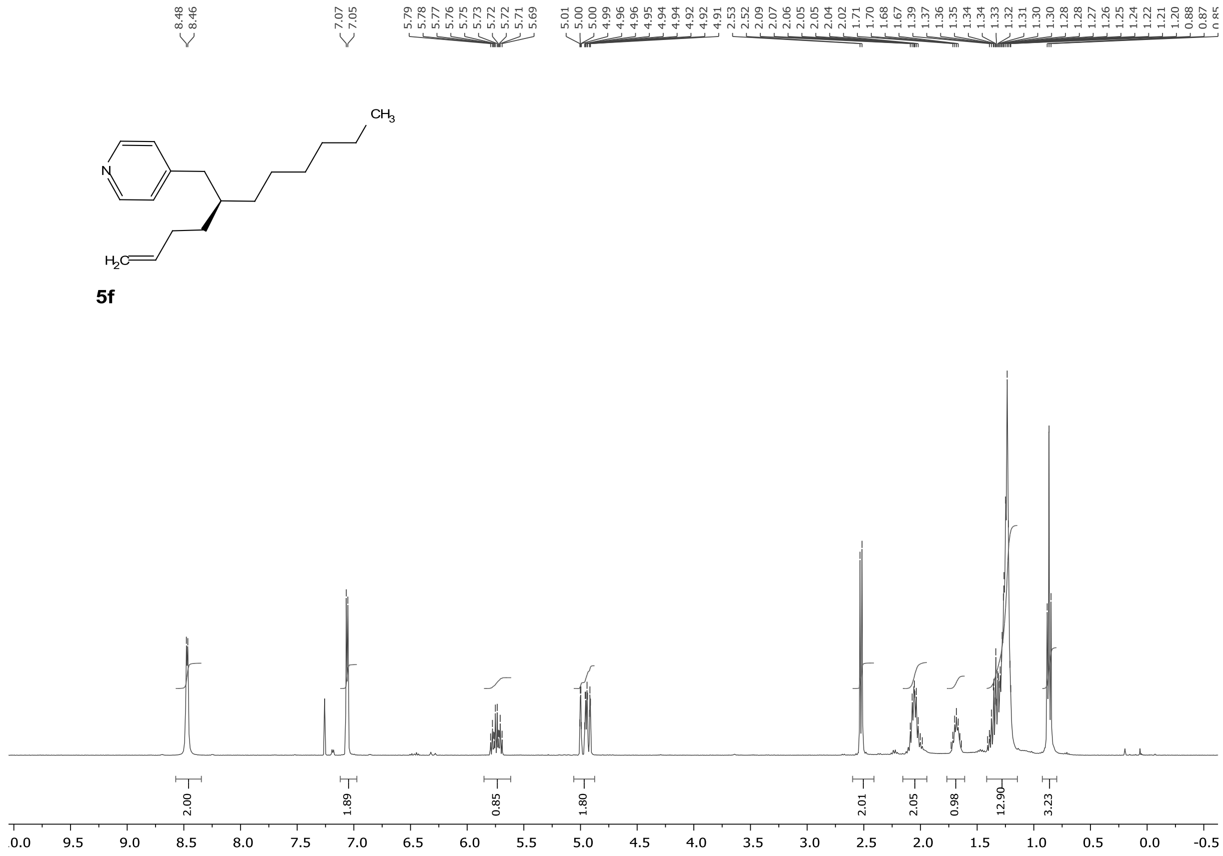


**Supplementary Figure 70** $^{1}$H NMR spectrum of **5f**

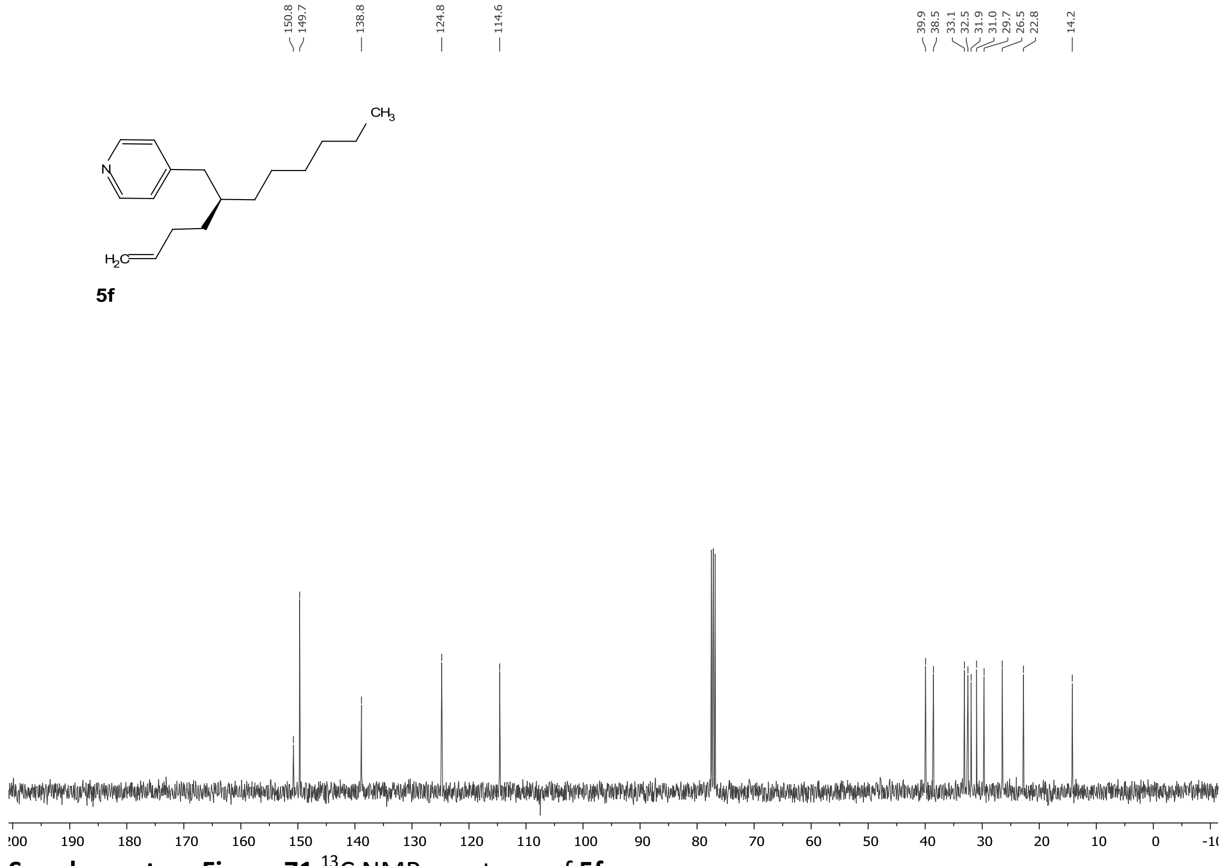


**Supplementary Figure 71** $^{13}$C NMR spectrum of **5f**

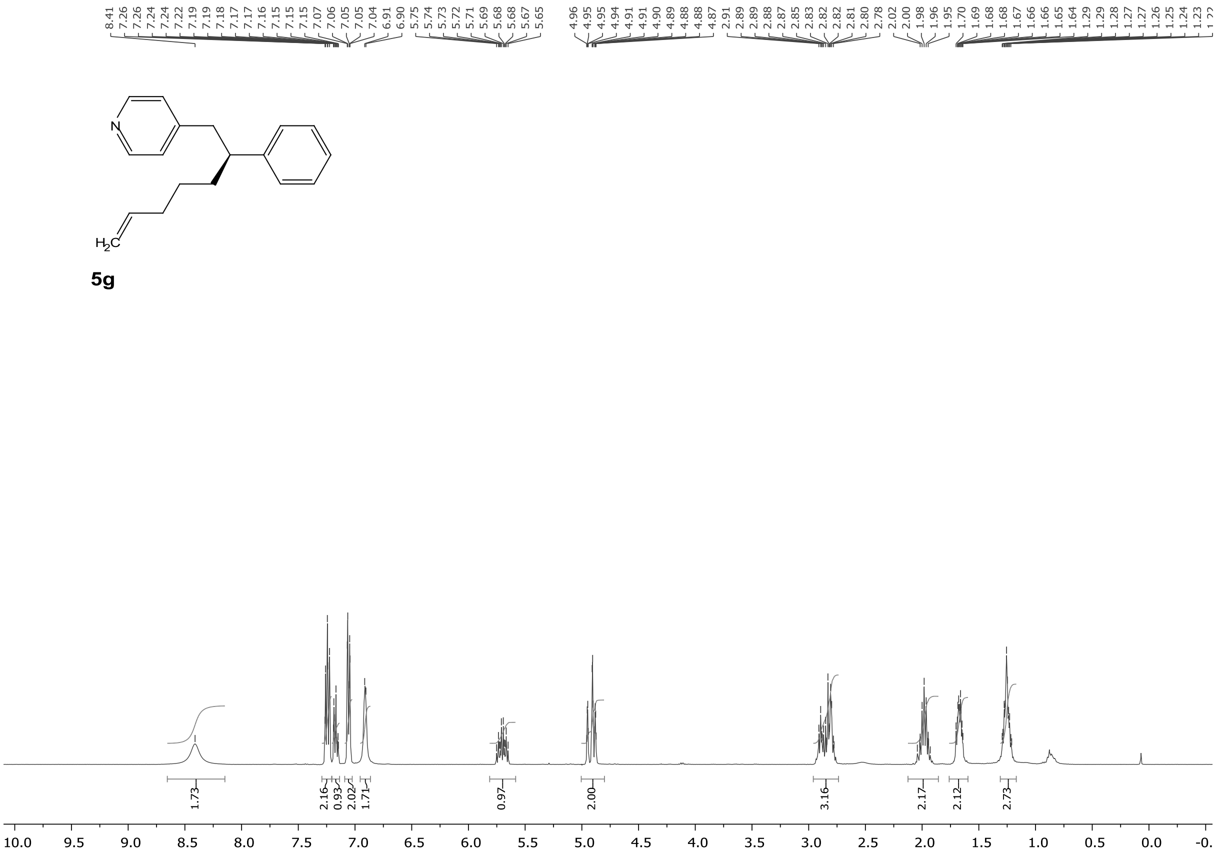


**Supplementary Figure 72** $^{1}$H NMR spectrum of **5g**

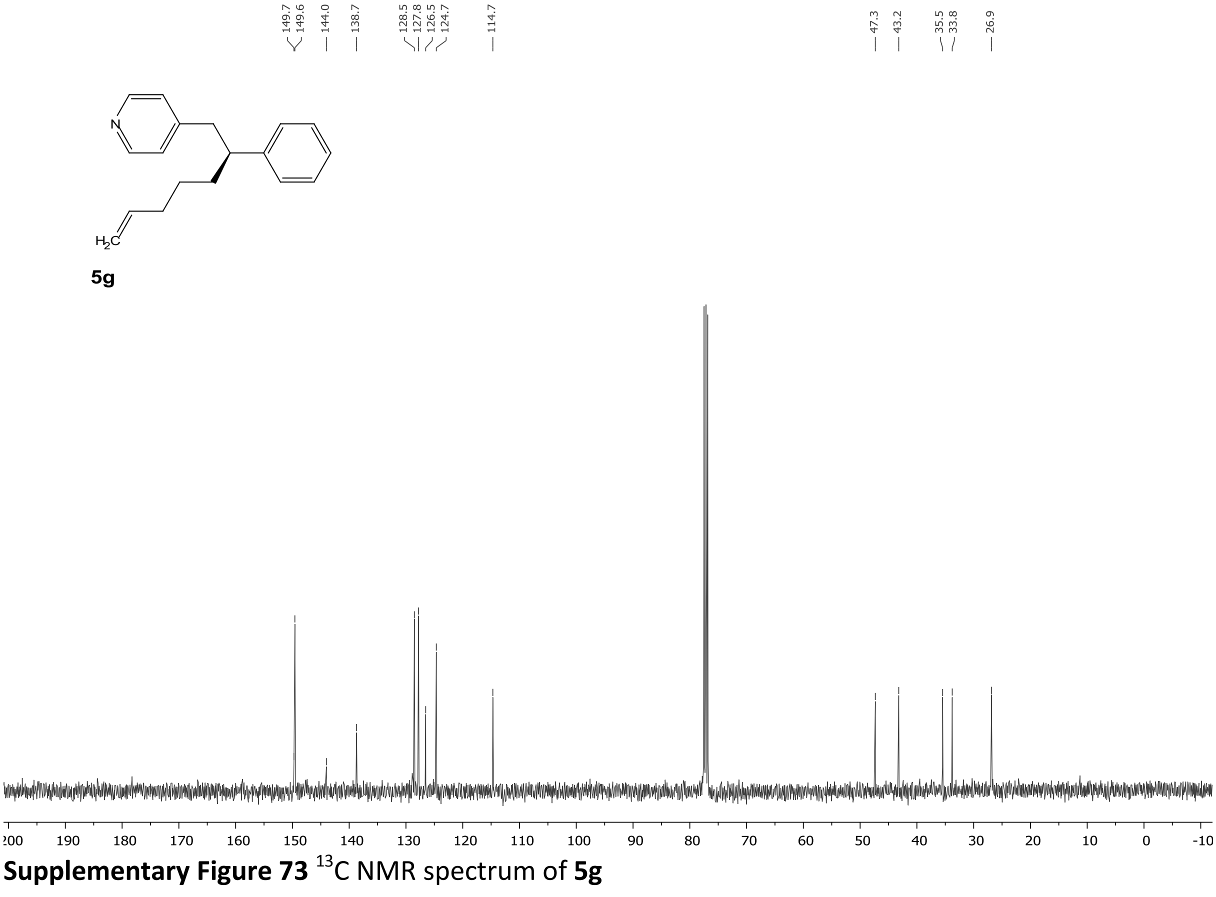


**Supplementary Figure 73** $^{13}$C NMR spectrum of **5g**

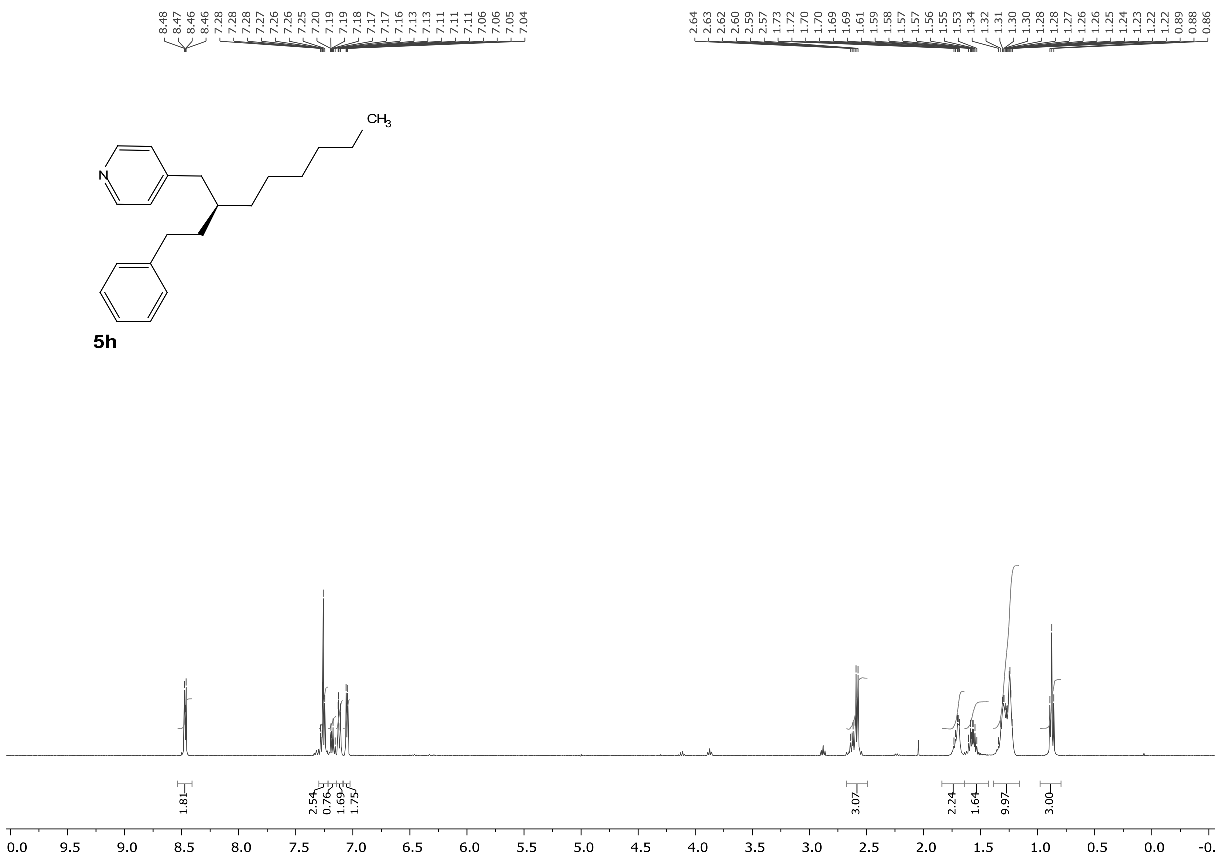

**Supplementary Figure 74** $^{1}$H NMR spectrum of **5h**

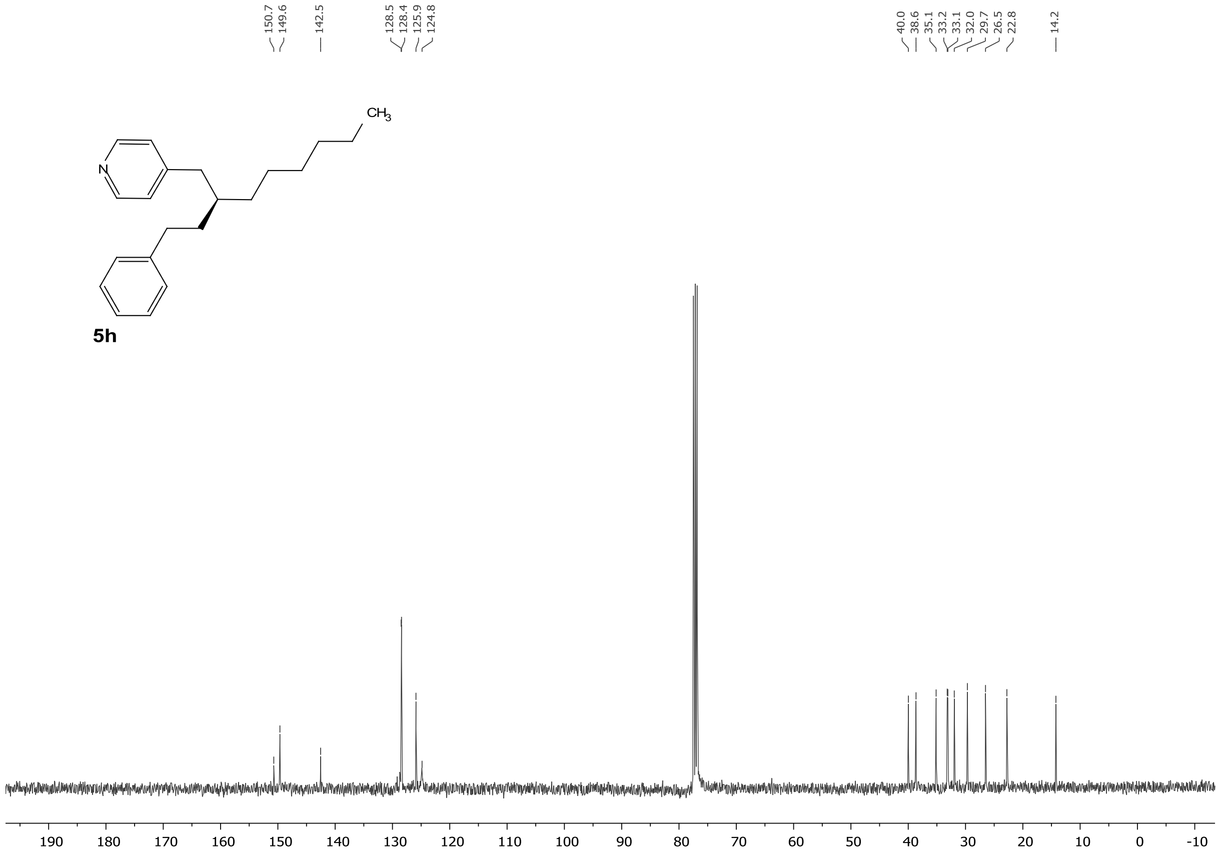

**Supplementary Figure 75** $^{13}$C NMR spectrum of **5h**

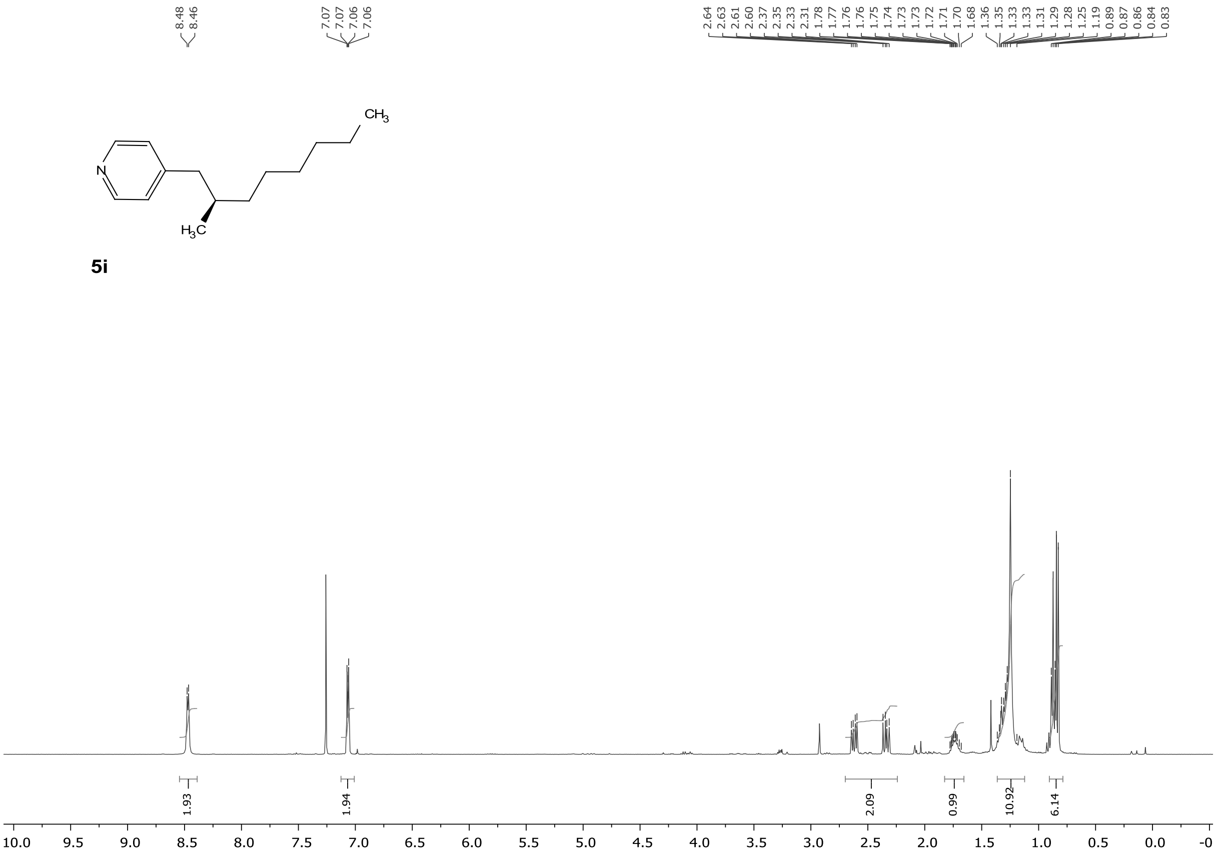


**Supplementary Figure 76** $^{1}$H NMR spectrum of **5i**

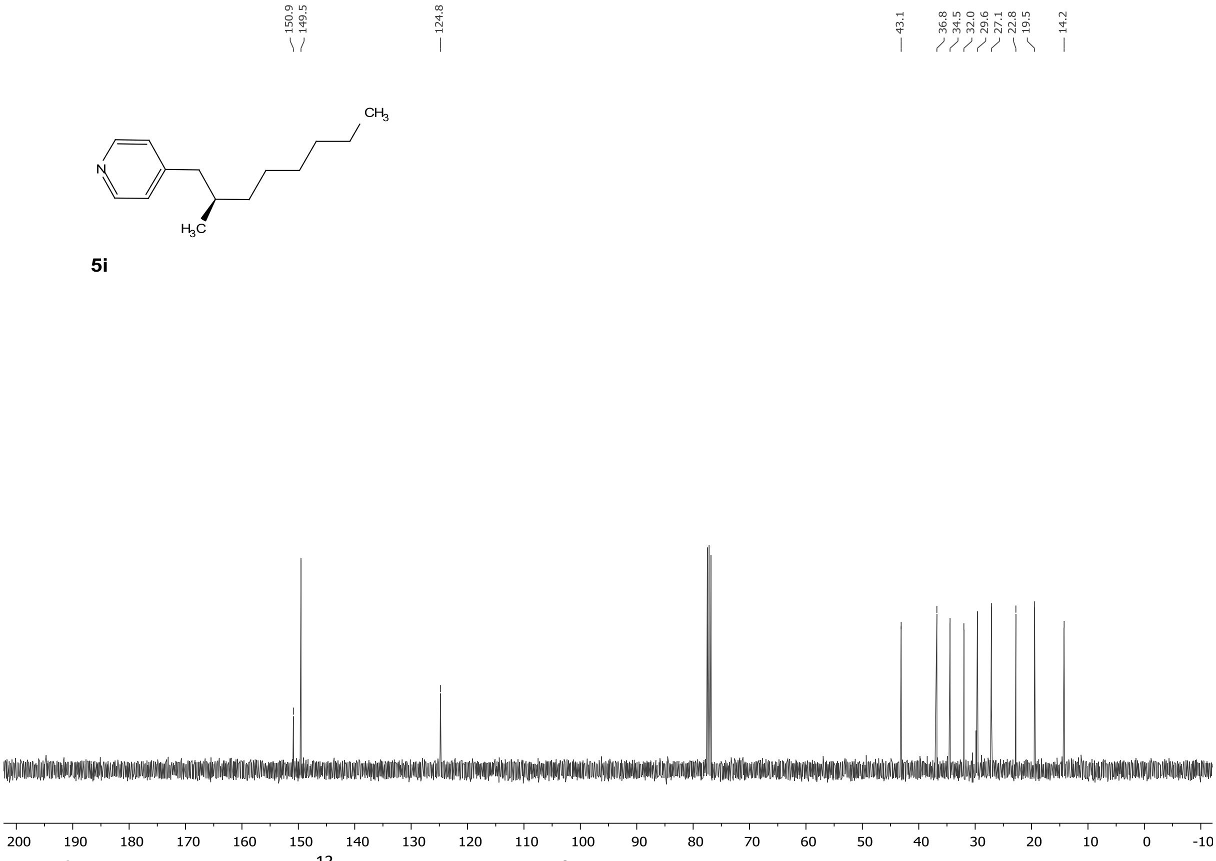


**Supplementary Figure 77** $^{13}$C NMR spectrum of **5i**

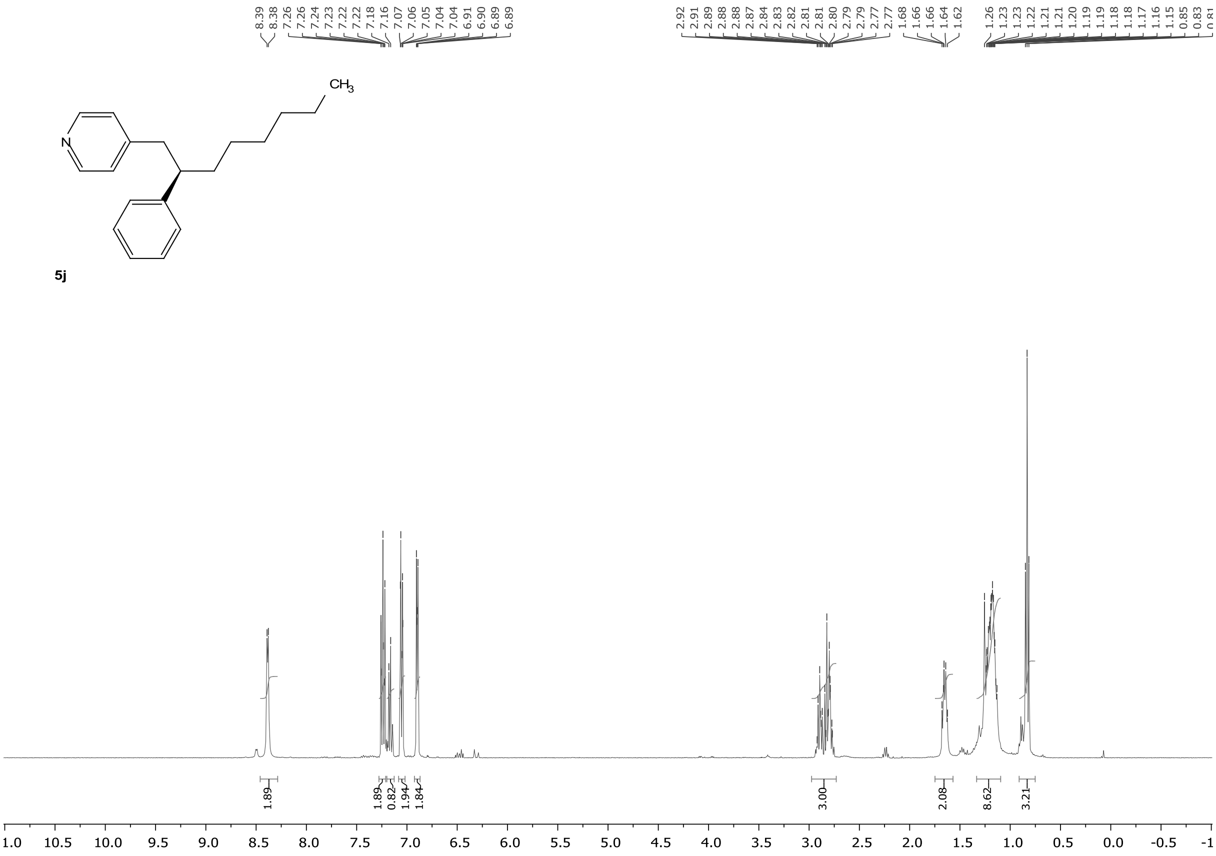

**Supplementary Figure 78** $^{1}$H NMR spectrum of **5j**

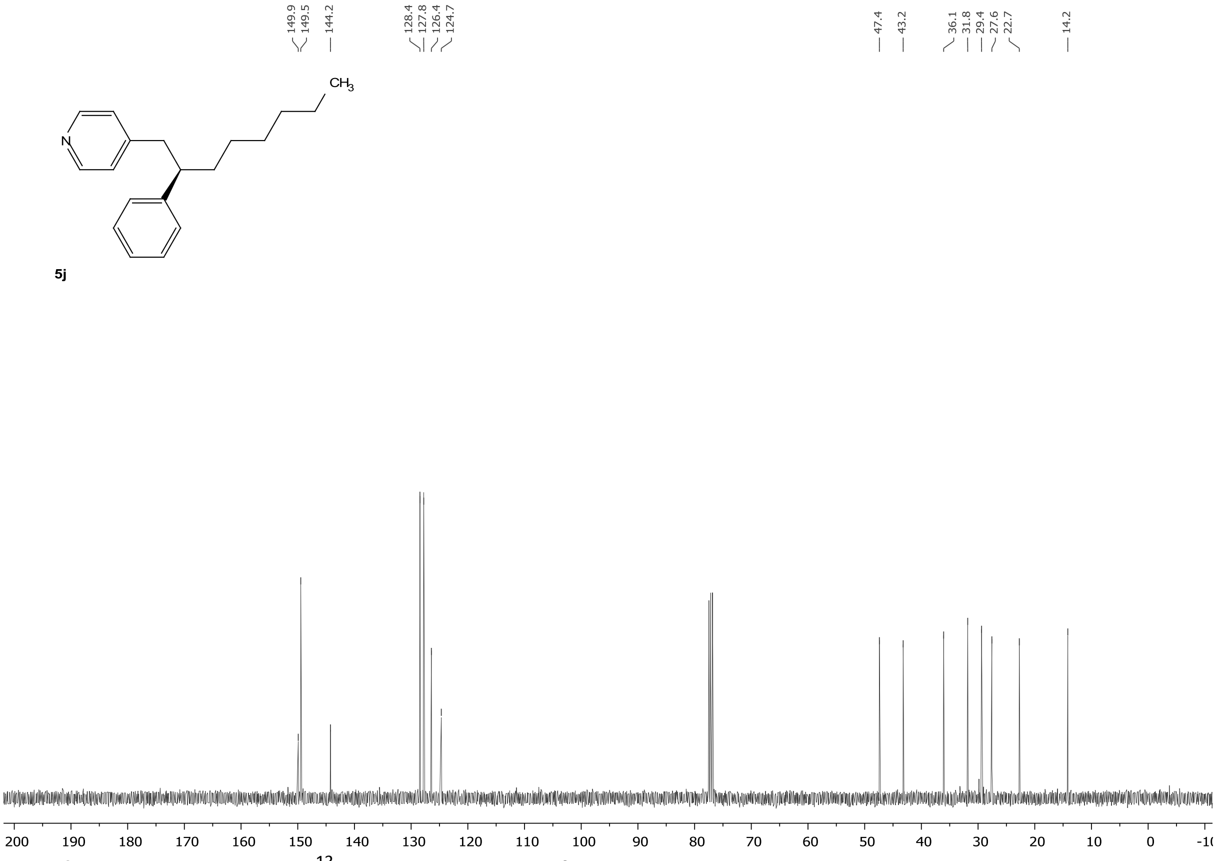

**Supplementary Figure 79** $^{13}$C NMR spectrum of **5j**

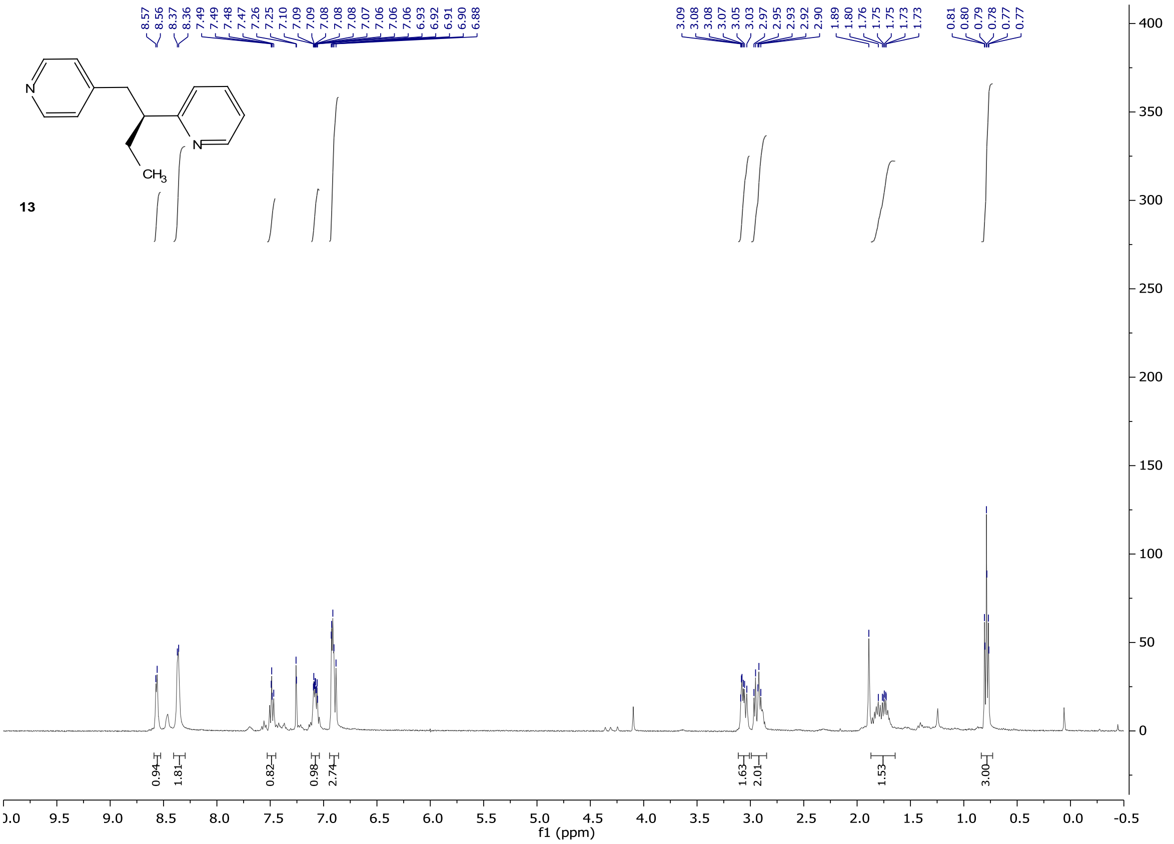

**Supplementary Figure 80** $^{1}$H NMR spectrum of **13**

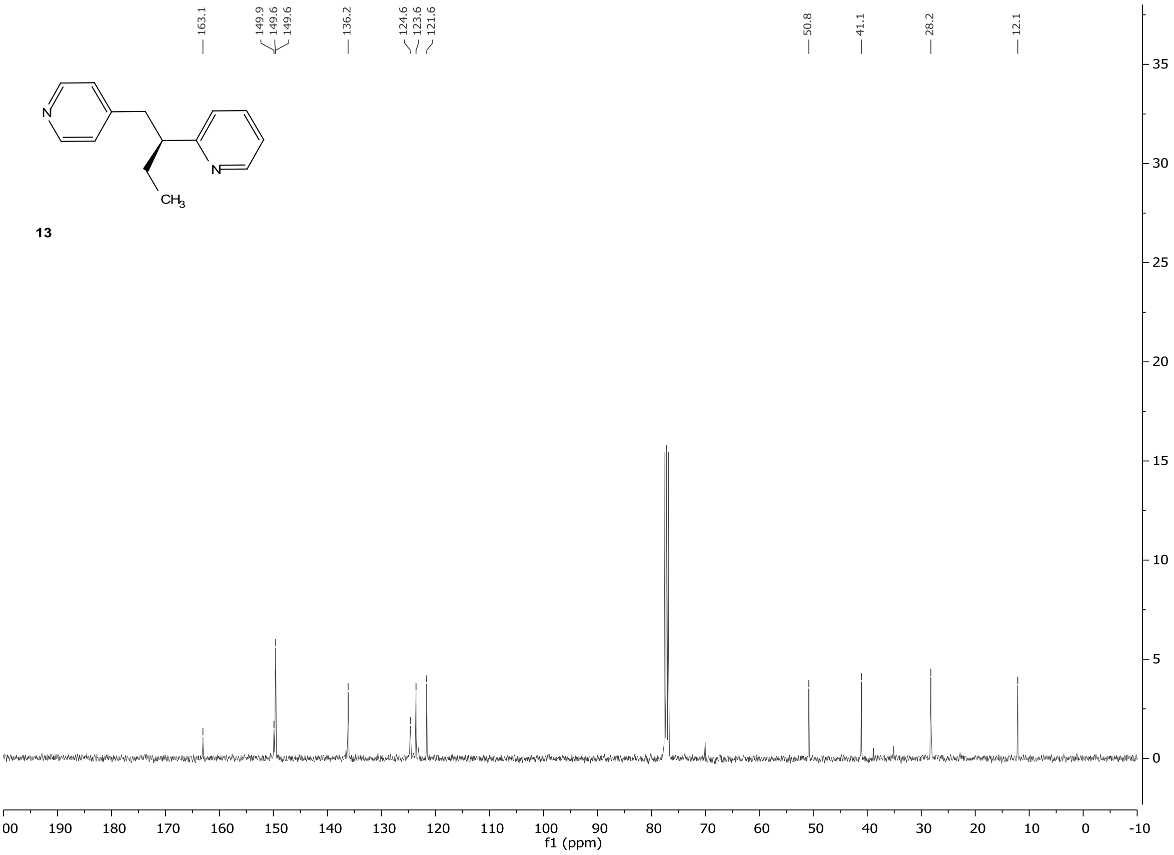

**Supplementary Figure 81** $^{13}$C NMR spectrum of **13**

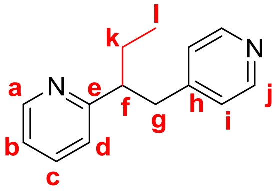


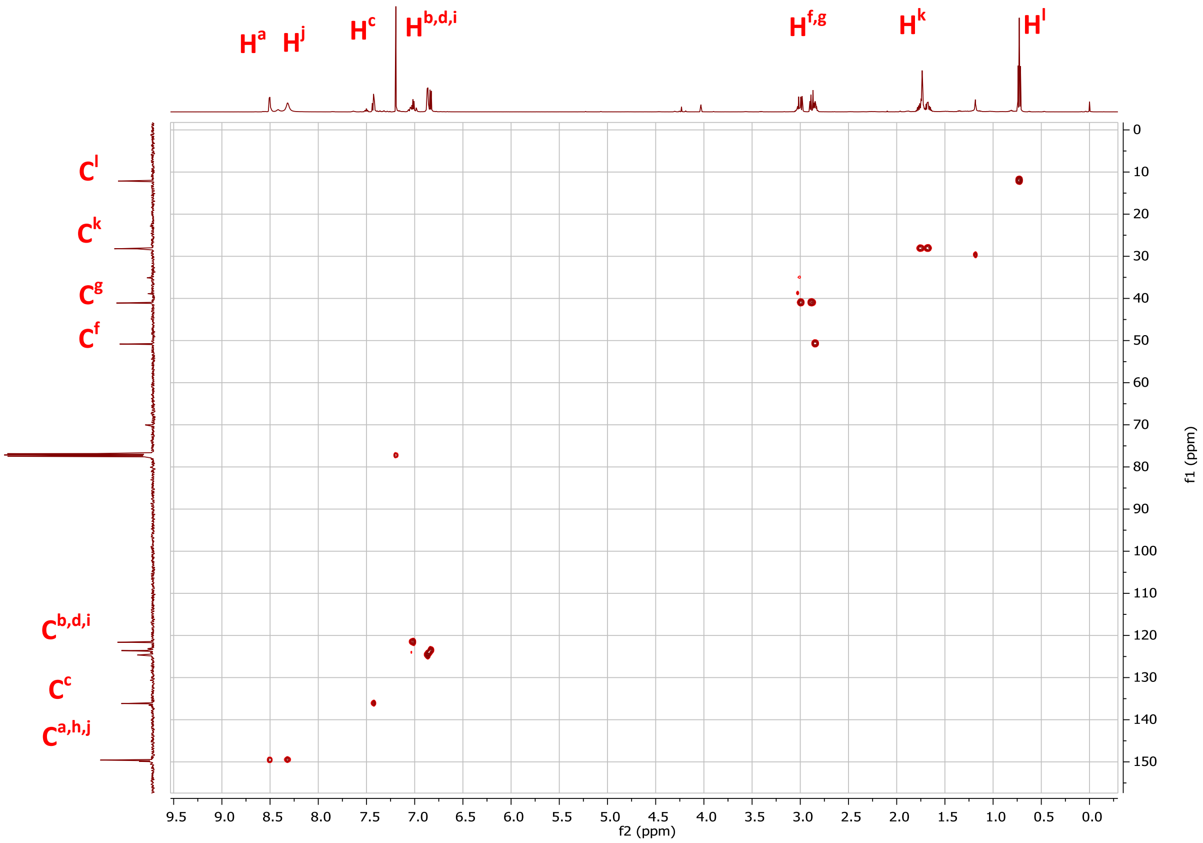


**Supplementary Figure 82** $^{1}H$-$^{13}C$-HSQC ($^{1}H$ at 600.0 MHz, $^{13}C$ at 150.75 MHz, $CDCl_3$) δ

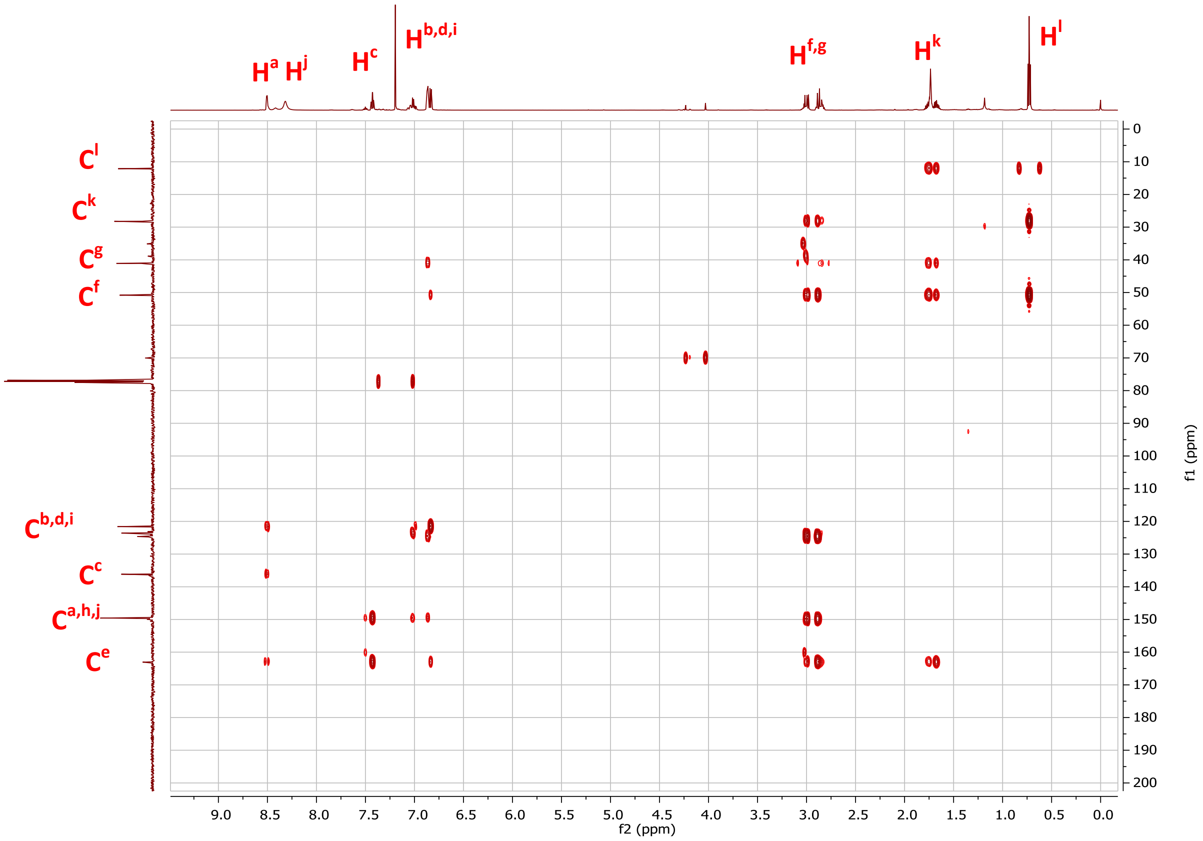


**Supplementary Figure 83** $^1$H-$^{13}$C-HMBC ($^1$H at 600.0 MHz, $^{13}$C at 150.75 MHz, $CDCl_3$) δ

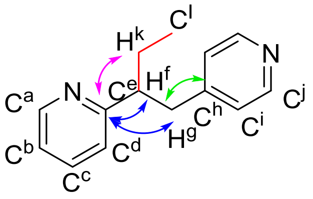


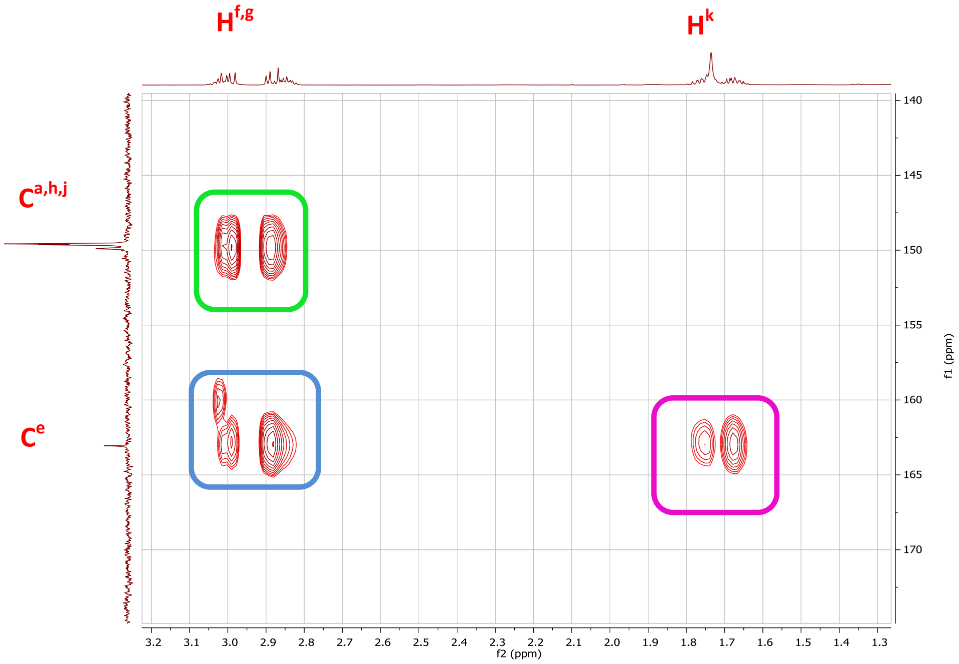


**Supplementary Figure 84** $^1$H-$^{13}$C-HMBC ($^1$H at 600.0 MHz, $^{13}$C at 150.75 MHz, $CDCl_3$) δ. Expansion of the signal corresponding to $^2J_{e\text{-}Hf}$ (blue), $^3J_{e\text{-}Hg}$ (blue), $^3J_{e\text{-}Hk}$ (purple) and $^2J_{h\text{-}Hg}$ (green).

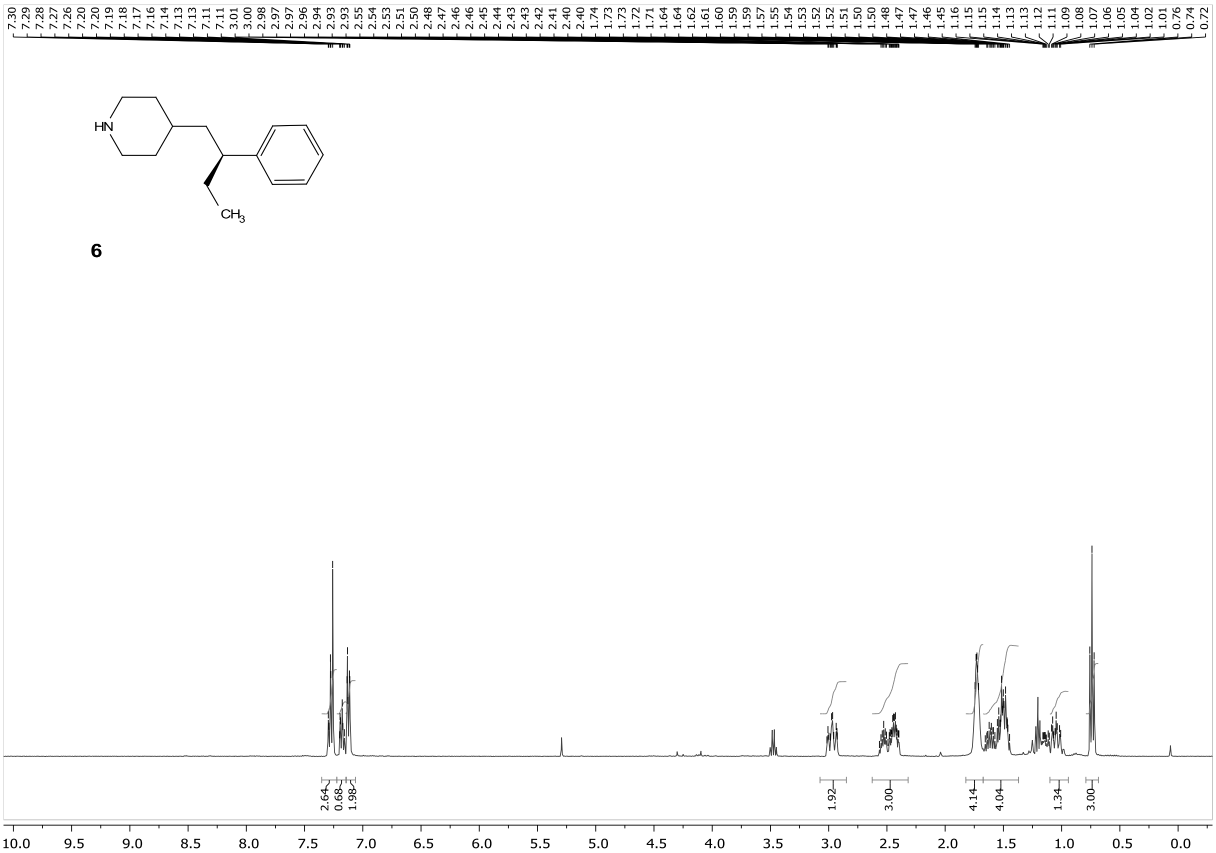


**Supplementary Figure 85** $^{1}$H NMR spectrum of **6**

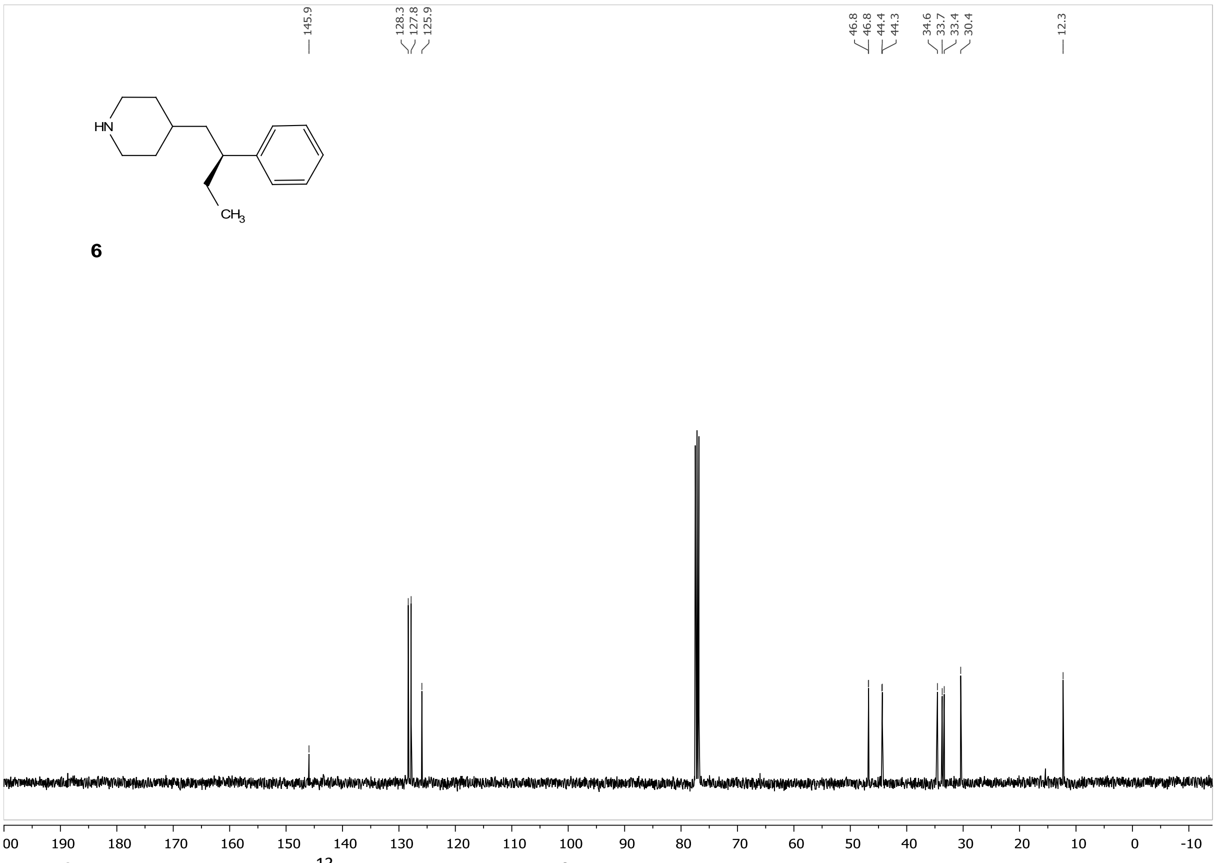


**Supplementary Figure 86** $^{13}$C NMR spectrum of **6**

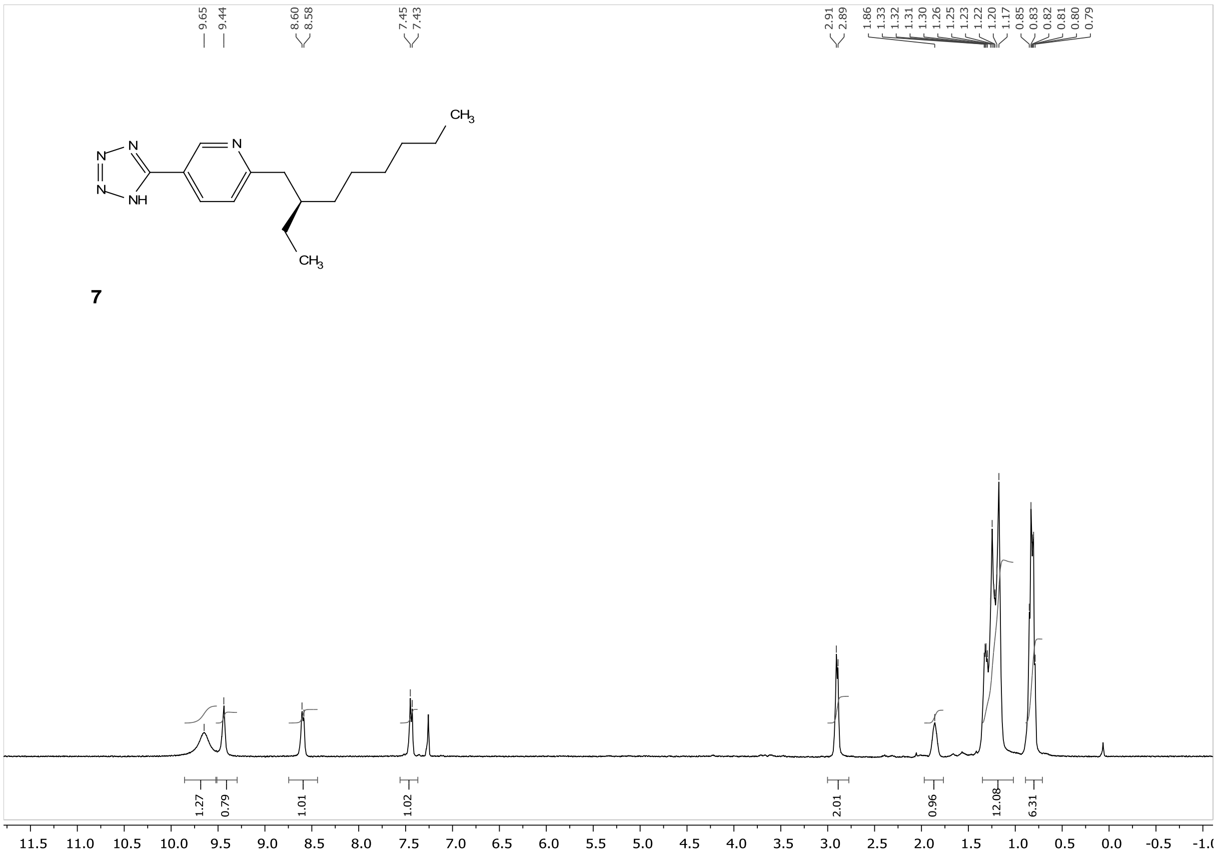

**Supplementary Figure 87** $^{1}$H NMR spectrum of **7**

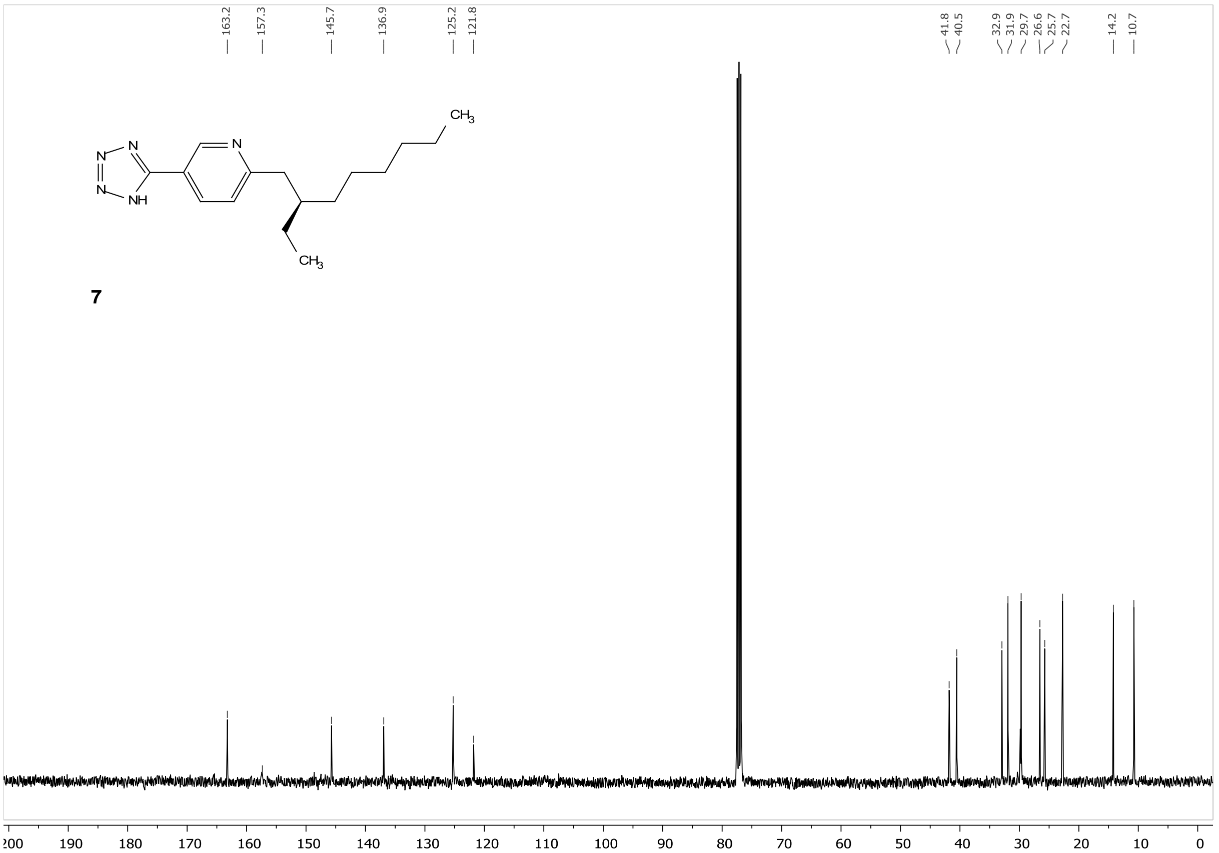

**Supplementary Figure 88** $^{13}$C NMR spectrum of **7**

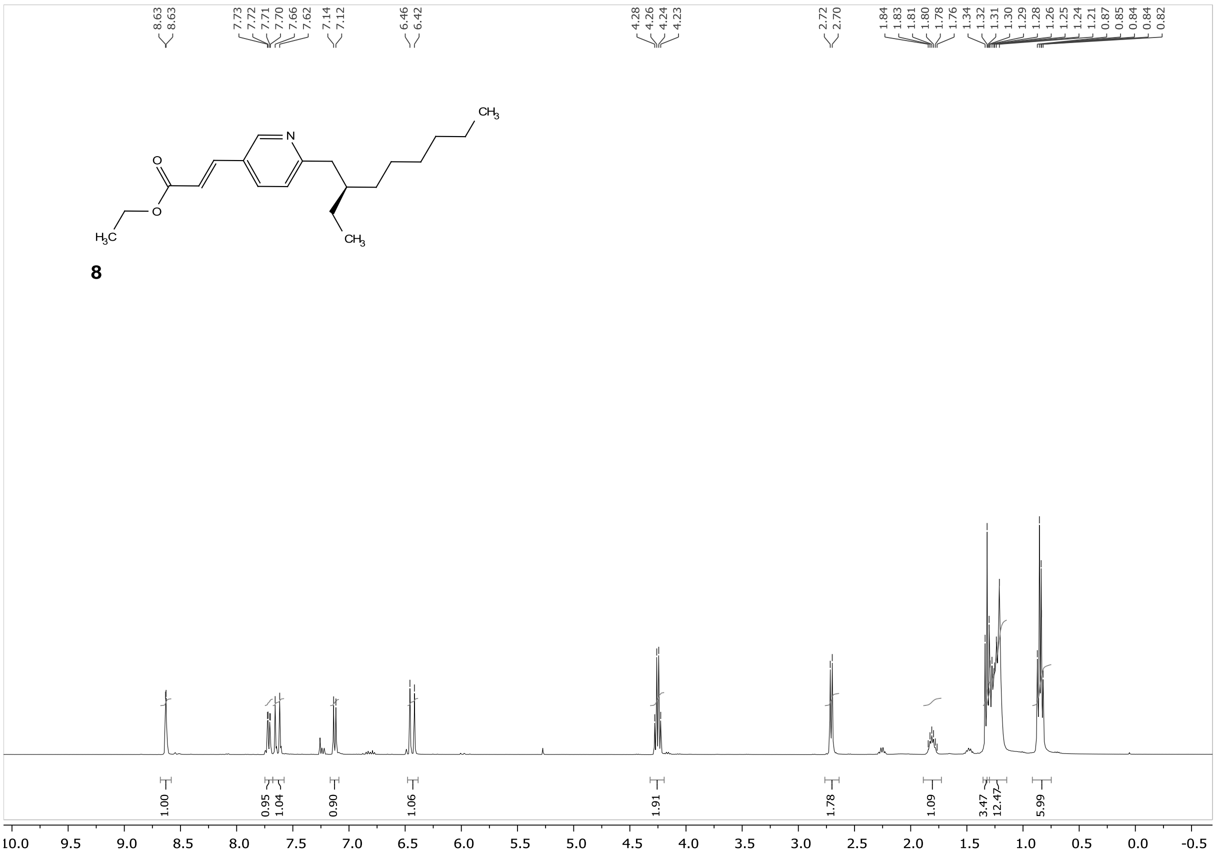


**Supplementary Figure 89** $^{1}$H NMR spectrum of **8**

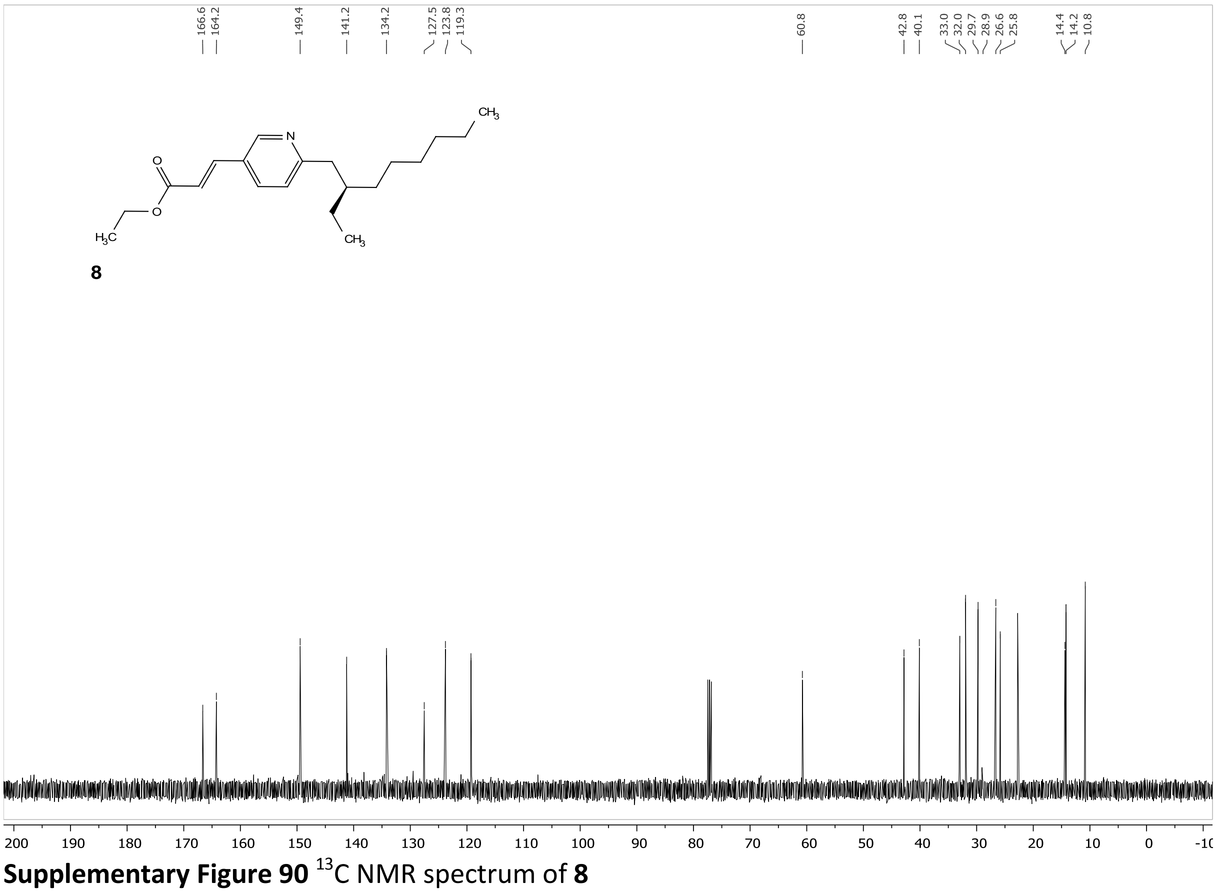


**Supplementary Figure 90** $^{13}$C NMR spectrum of **8**

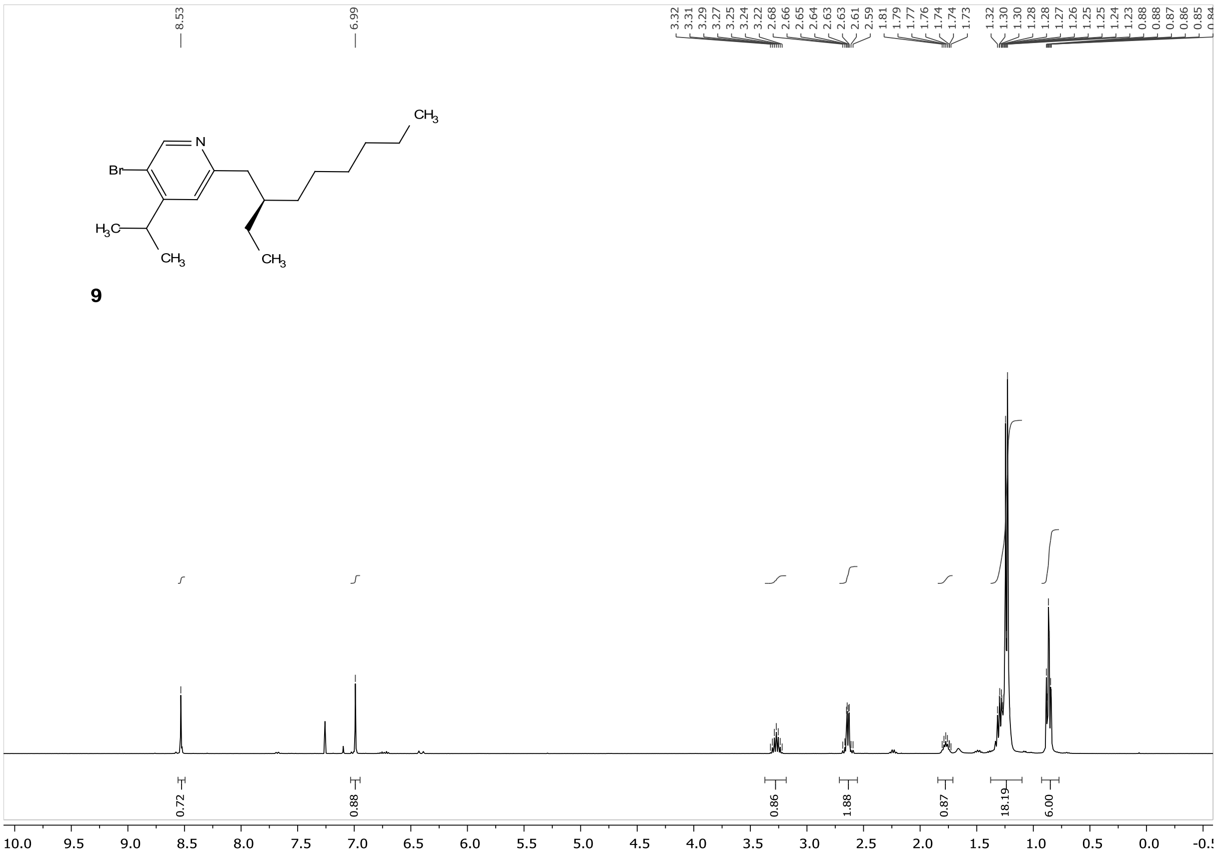


**Supplementary Figure 91** $^{1}$H NMR spectrum of **9**

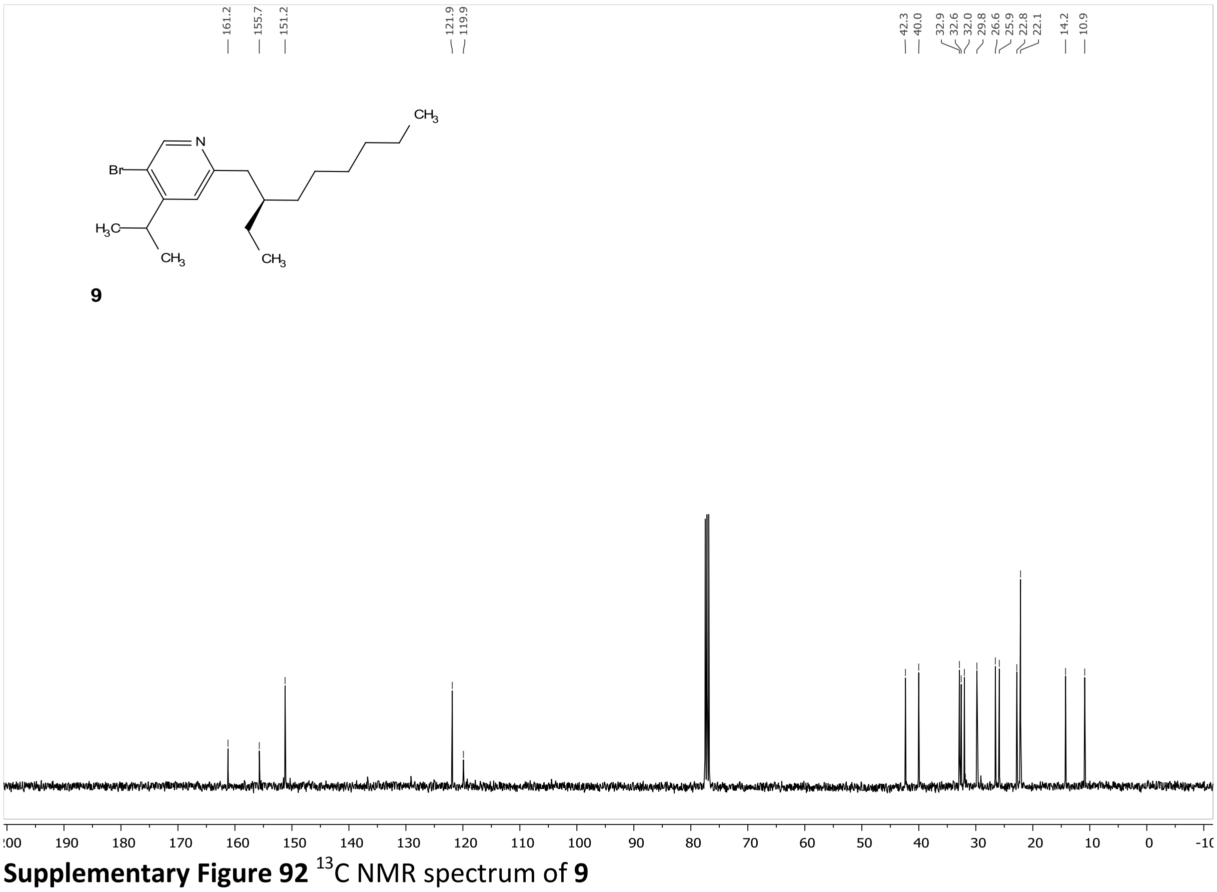


**Supplementary Figure 92** $^{13}$C NMR spectrum of **9**

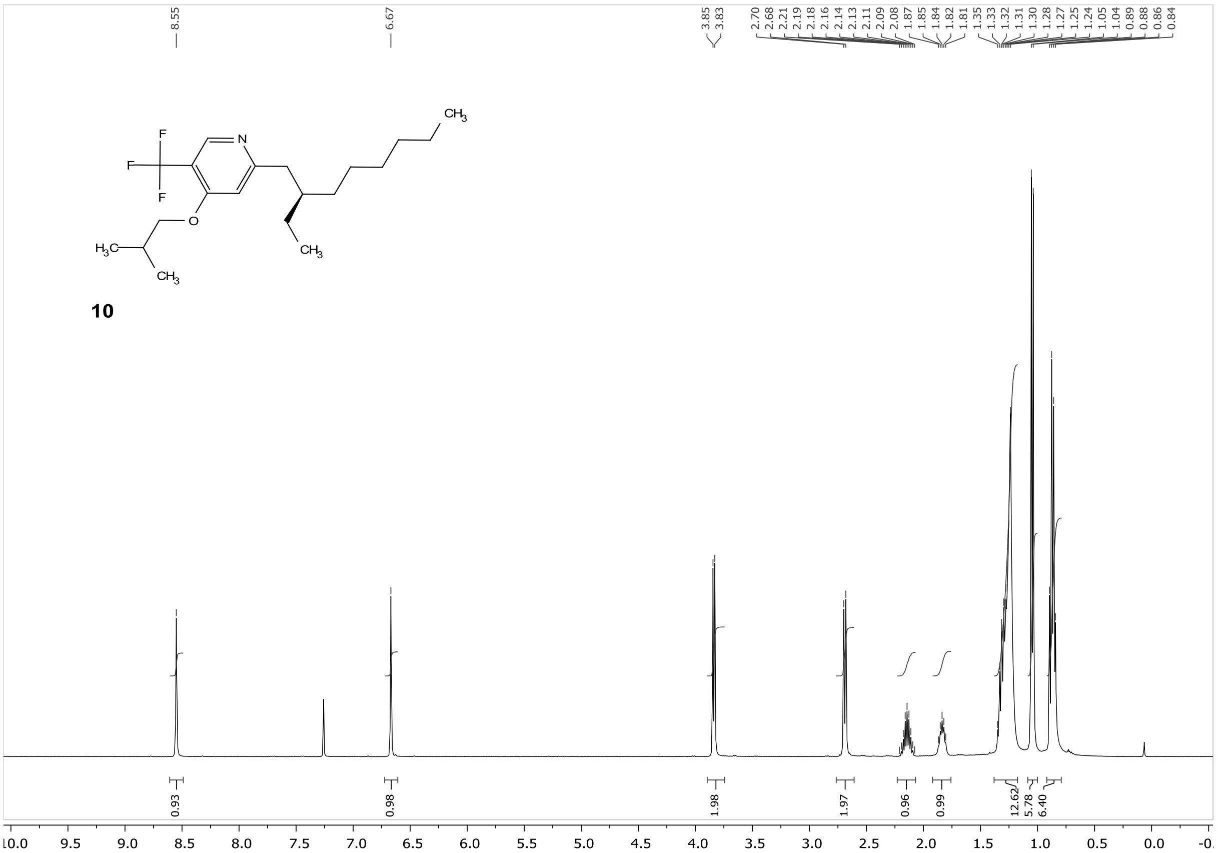

**Supplementary Figure 93** $^{1}$H NMR spectrum of **10**

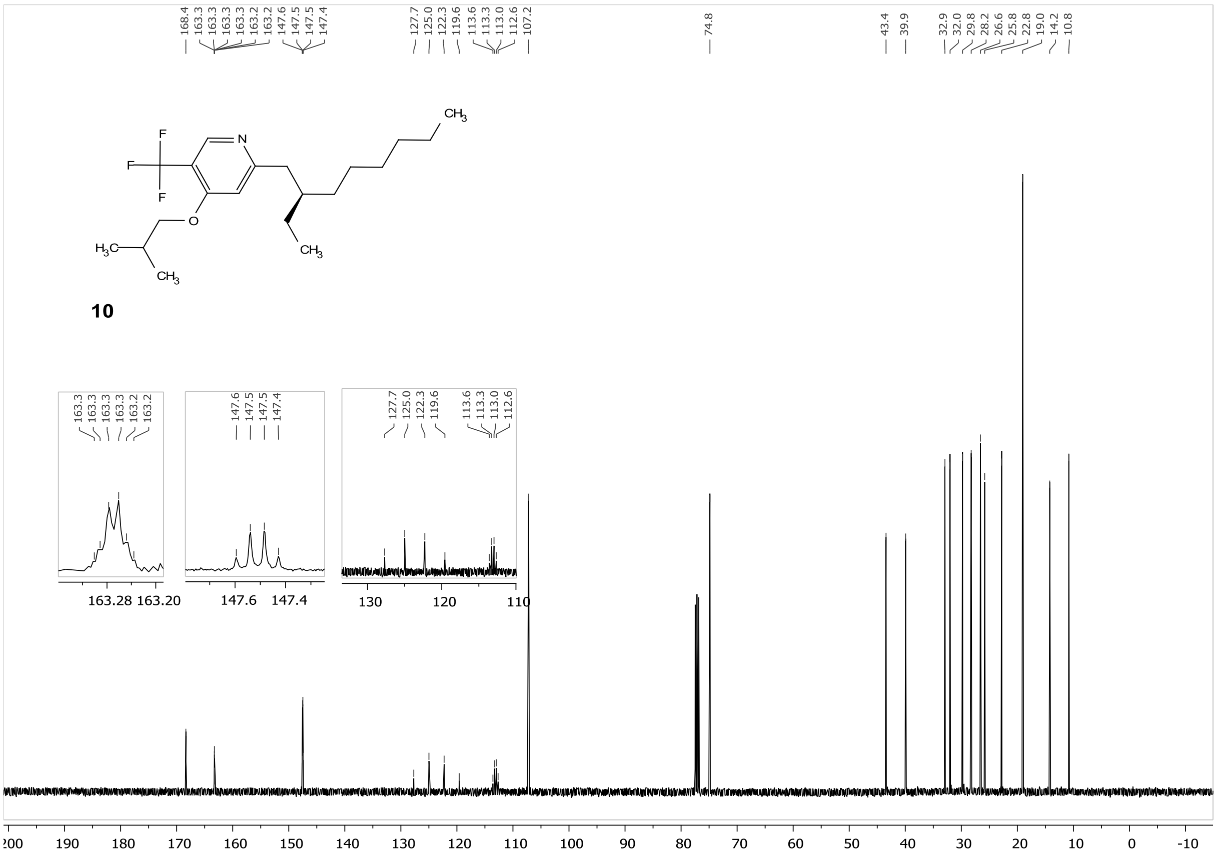

**Supplementary Figure 94** $^{13}$C NMR spectrum of **10**

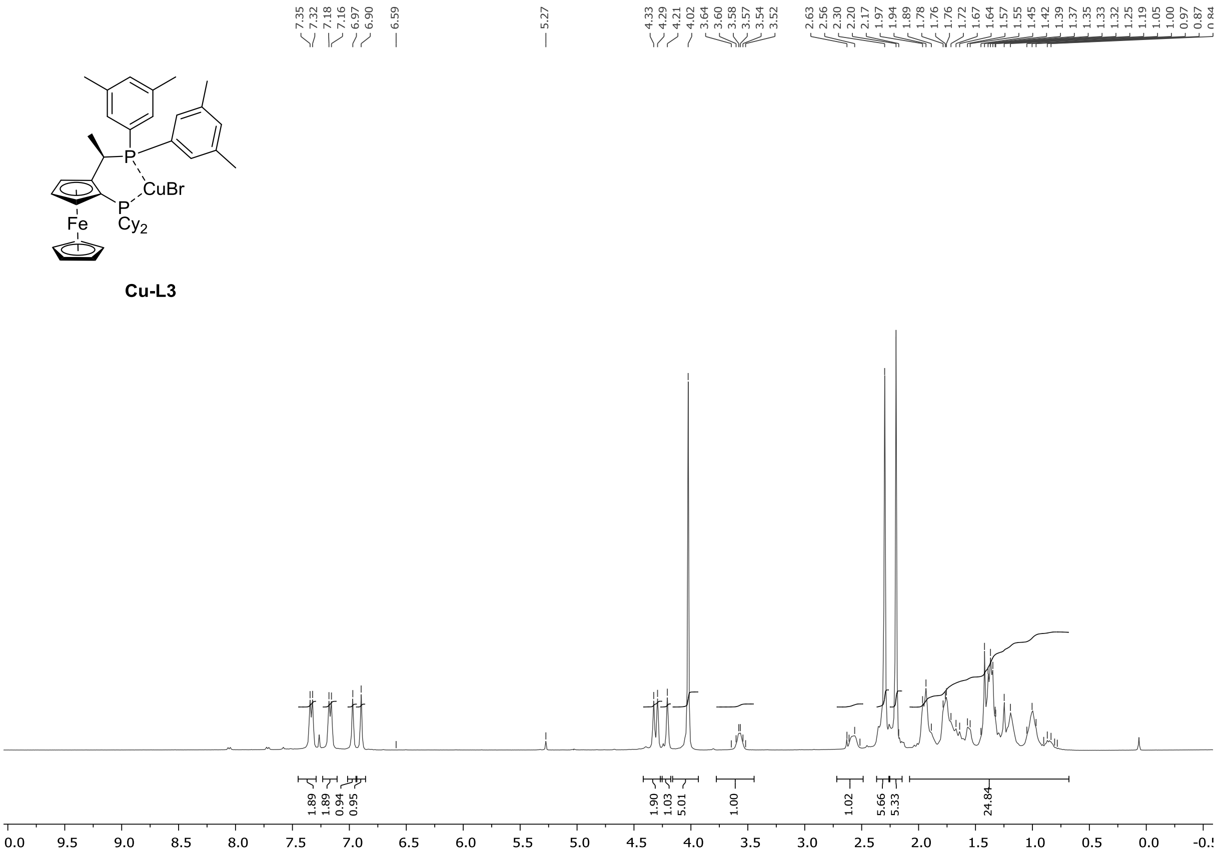

**Supplementary Figure 95** $^{1}$H NMR spectrum of **Cu-L3**

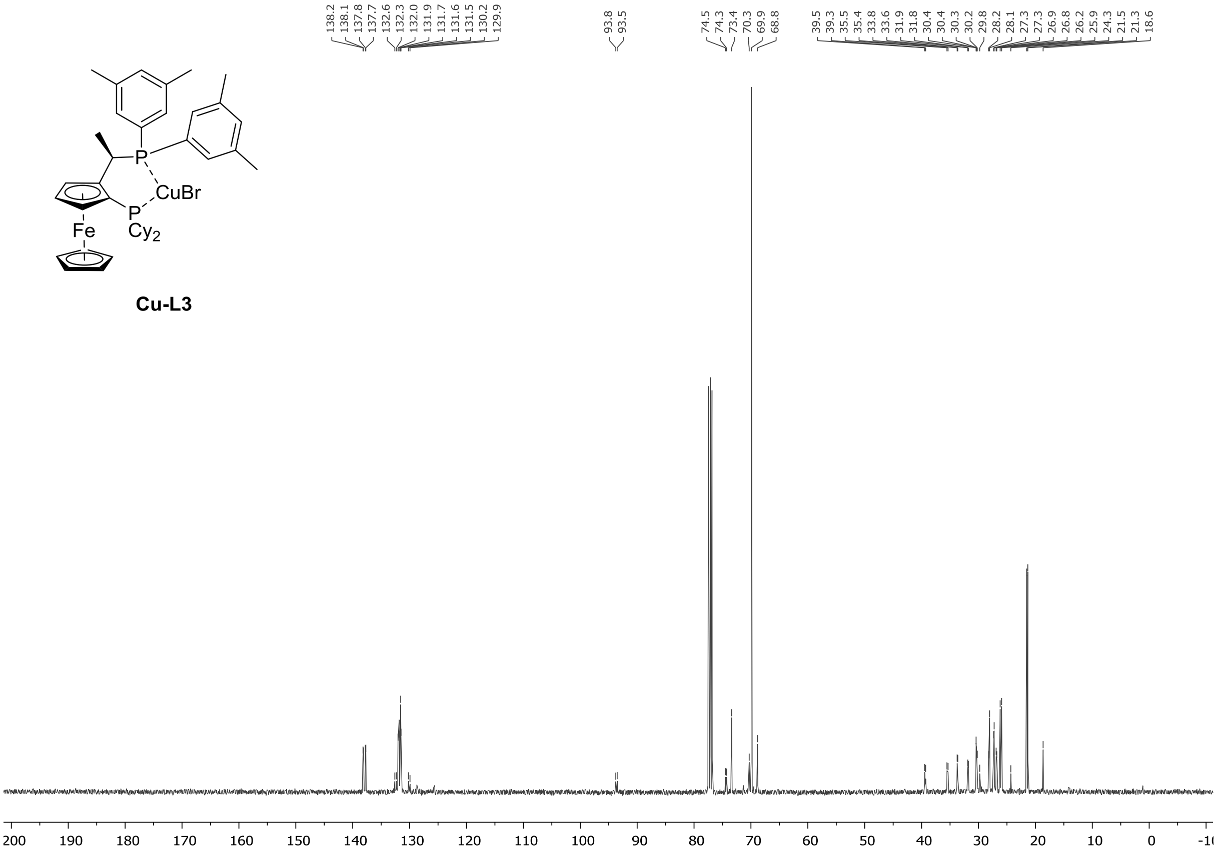

**Supplementary Figure 96** $^{13}$C NMR spectrum of **Cu-L3:**

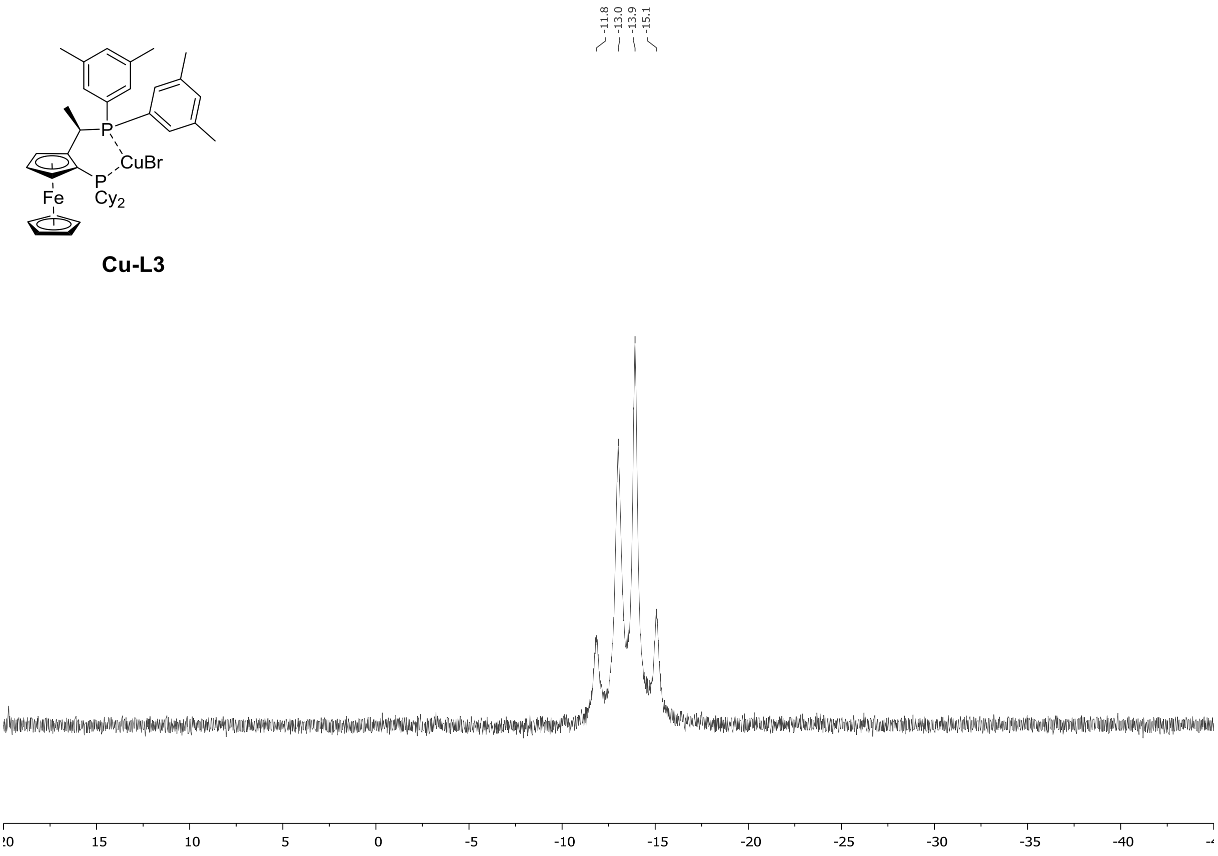


**Supplementary Figure 97** $^{32}$P NMR spectrum of **Cu-L3**

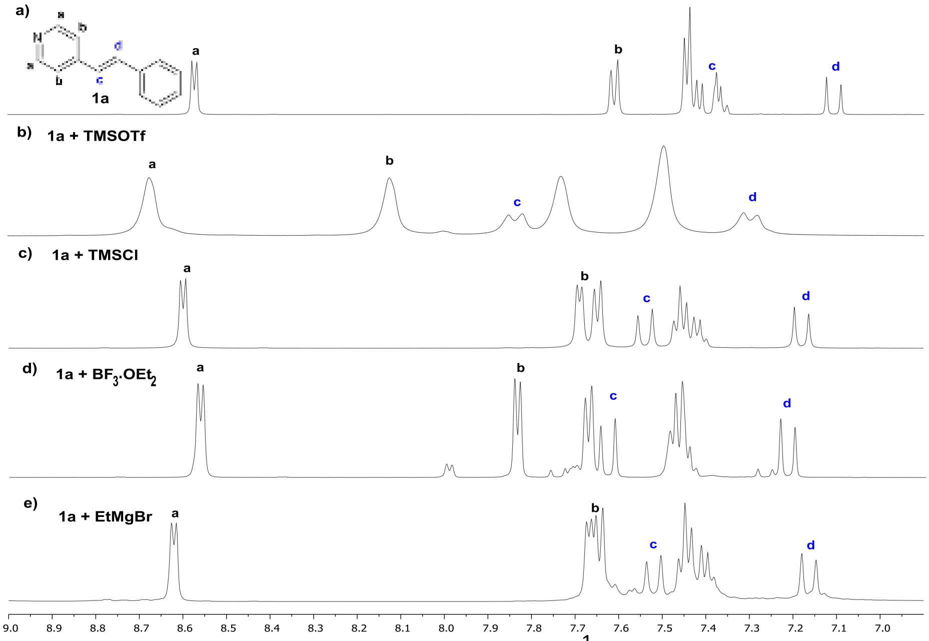


**Supplementary Figure 98 Effect of LA on substrates 1a,** $^{1}$H NMR experiments in $CD_2Cl_2$ at -60 °C: **a)** $^{1}$H NMR spectra of **1a**; **b)** $^{1}$H NMR spectra of **1a** and TMSOTf (2.0 equiv.); **c)** $^{1}$H NMR

spectra of **1a** and TMSCl (2.0 equiv.); **d)** $^{1}H$ NMR spectra of **1a** and $BF_3$·$OEt_2$ (2.0 equiv.); **e)** $^{1}H$ NMR spectra of **1a** and EtMgBr (2.0 equiv.).

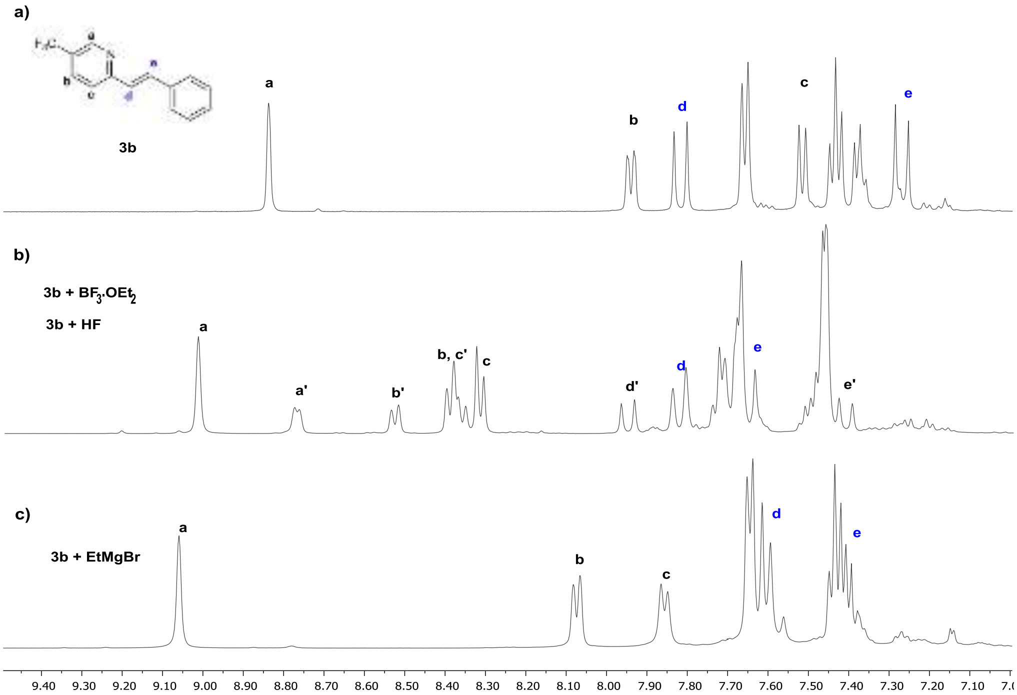


**Supplementary Figure 99 Effect of LA on substrates 3b**, $^{1}H$ NMR experiments in $CD_2Cl_2$ at -60 °C: **a)** $^{1}H$ NMR spectra of **3b**; **b)** $^{1}H$ NMR spectra of **3b** and $BF_3$•$Et_2O$ (1.5 equiv.), along with the complex of $BF_3$•$Et_2O$ with **3b**, a complex with residual HF with **3b** was observed; **c)** $^{1}H$ NMR spectra of **3b** and EtMgBr (2.0 equiv.).

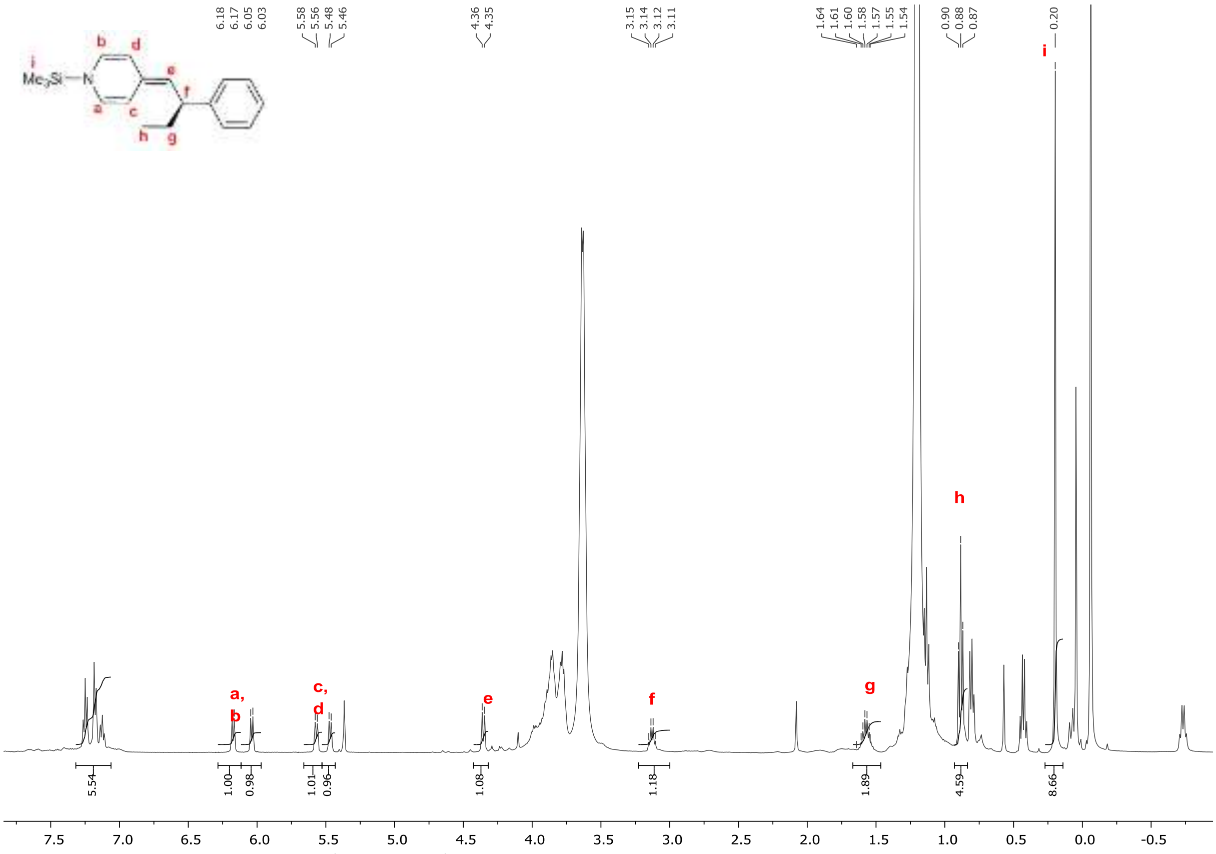

**Supplementary Figure 100** $^1H$ NMR ($^1H$ at 500 MHz, $CD_2Cl_2$)

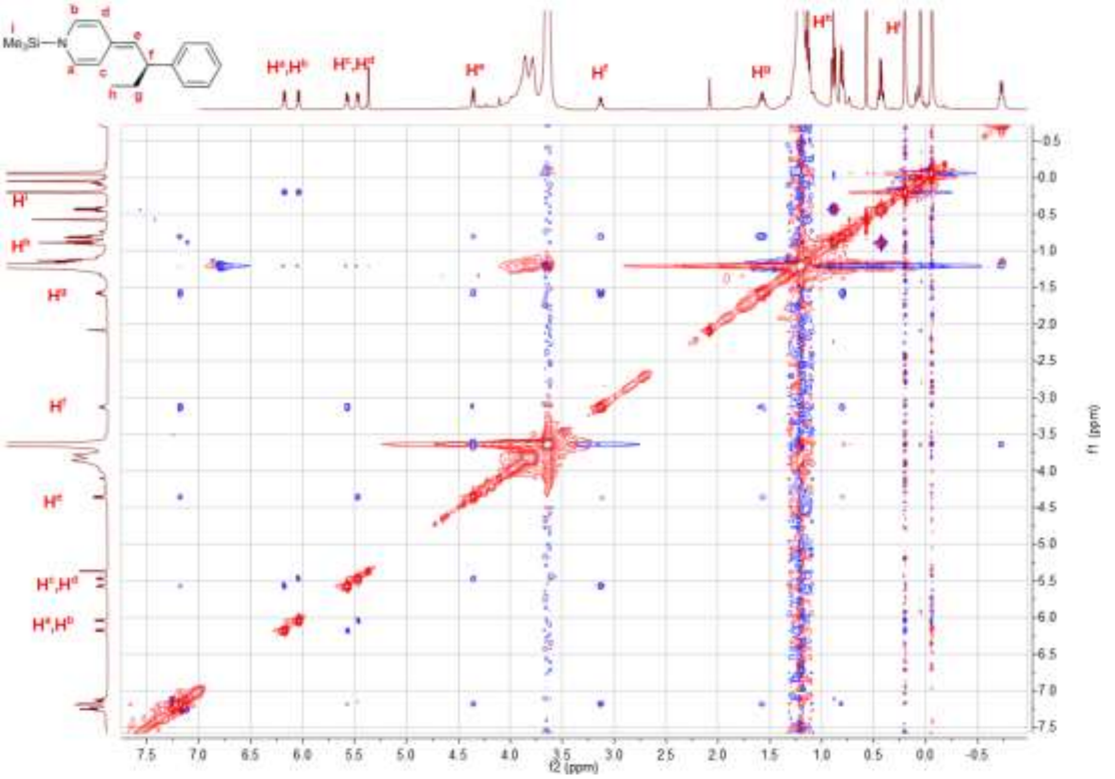

**Supplementary Figure 101** 2D NOESY ($^1H$ at 500 MHz, $CD_2Cl_2$)

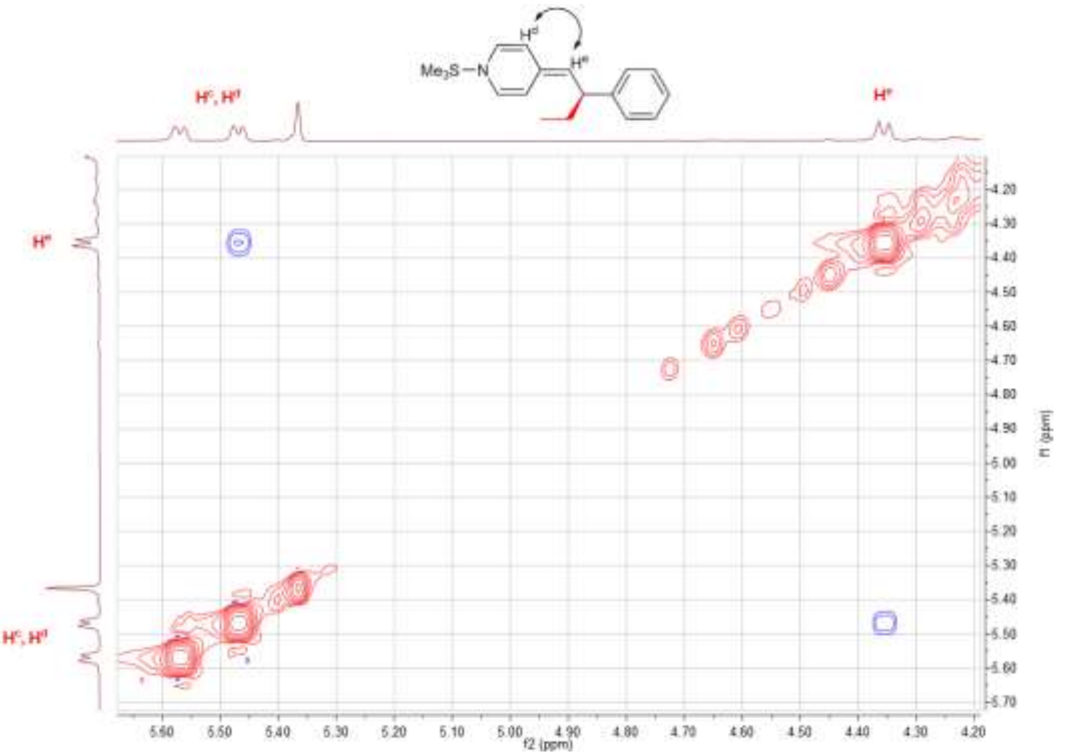


**Supplementary Figure 102** expansion of the spectrum 2D NOESY ($^1H$ at 500 MHz, $CD_2Cl_2$)

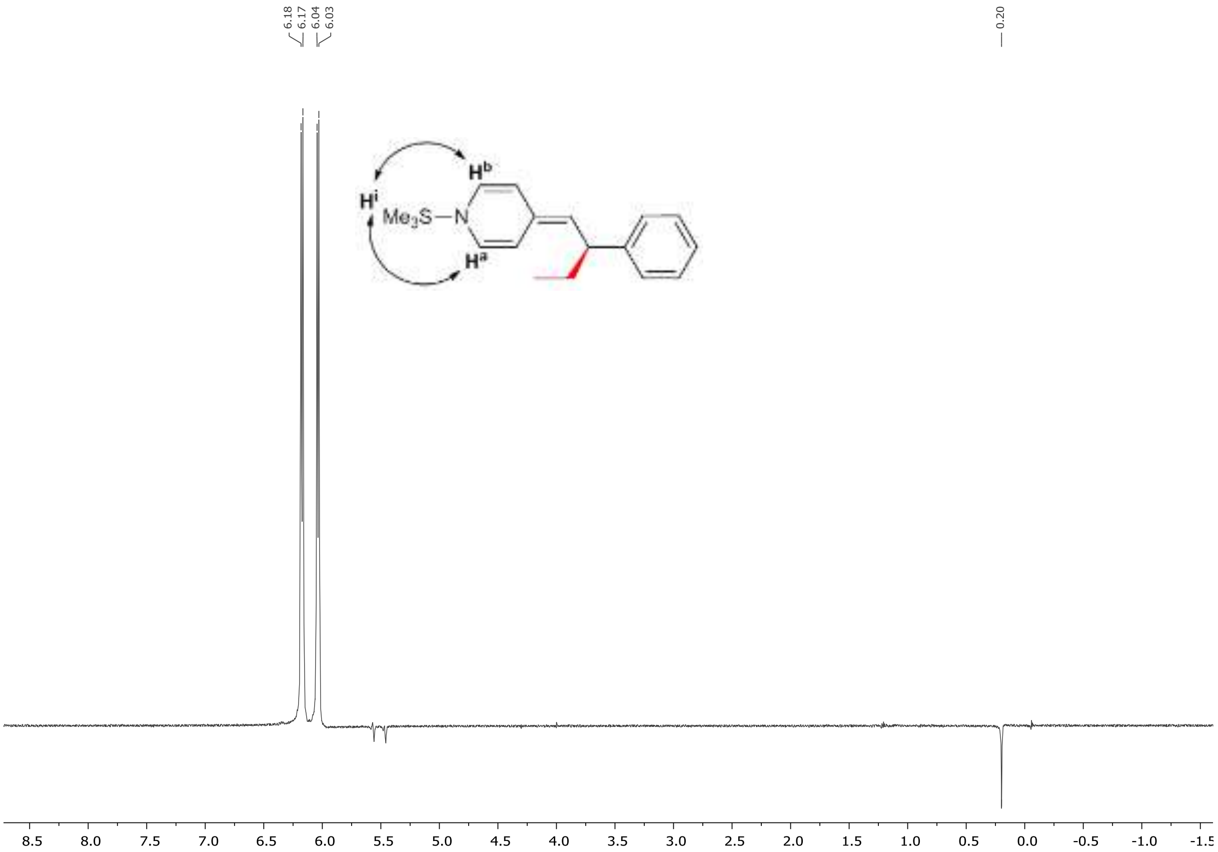


**Supplementary Figure 103** 1D NOESY ($^1H$ at 500 MHz, $CD_2Cl_2$)

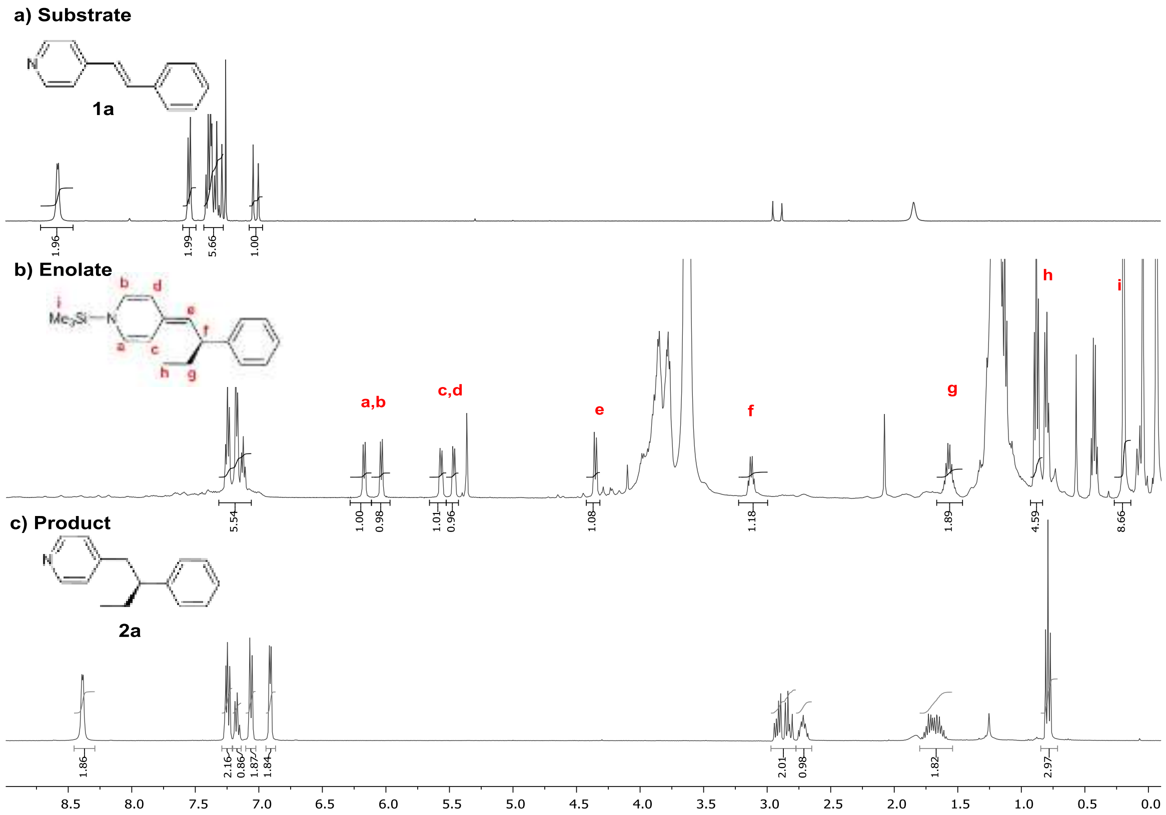


**Supplementary Figure 104 Comparison of 1H NMR spectra of the CA reaction mixture before quench with alkene substrate 1a and CA product 2a. a)** $^{1}$H NMR spectra of substrate **1a** in $CDCl_3$ at RT; **b)** $^{1}$H NMR spectra of enolate before quenching the reaction in $CD_2Cl_2$ at -60 °C; **c)** $^{1}$H NMR spectra of product **2a** in $CDCl_3$ at RT.

## CSP-HPLC and Chiral SFC

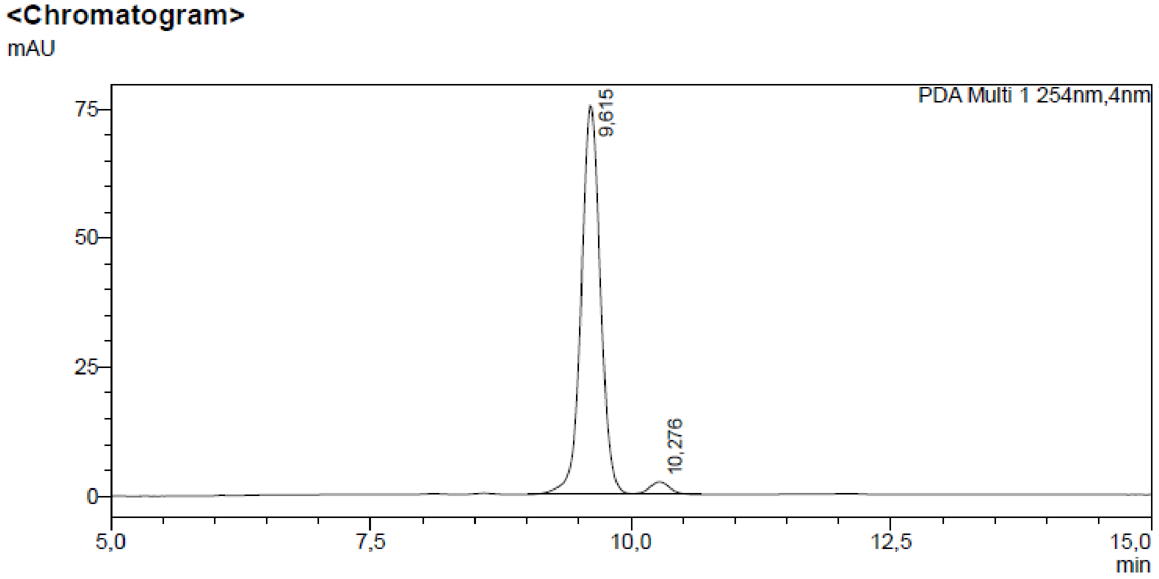


**<Peak Table>**

PDA Ch1 254nm

| Peak# | Ret. Time | Area | Height | Conc. | Unit | Mark | Name |
|---|---|---|---|---|---|---|---|
| 1 | 9,615 | 968729 | 75287 | 96,661 | | M | |
| 2 | 10,276 | 33460 | 2443 | 3,339 | | V M | |
| Total | | 1002189 | 77730 | | | | |

**Supplementary Figure 105** CSP-HPLC trace of **(*S*)-2a**

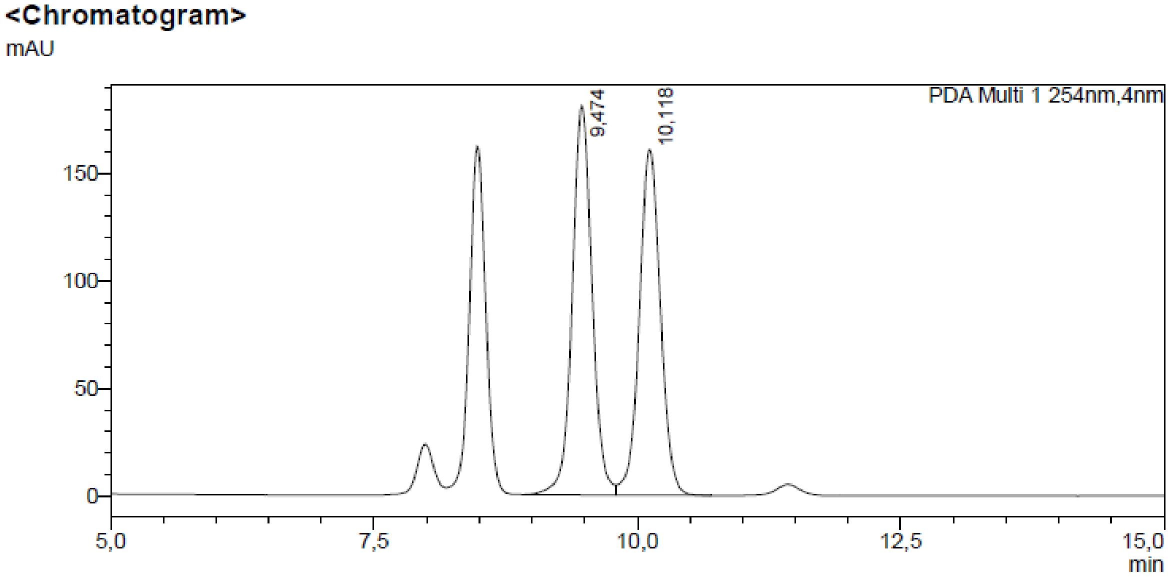


**<Peak Table>**

PDA Ch1 254nm

| Peak# | Ret. Time | Area | Height | Conc. | Unit | Mark | Name |
|---|---|---|---|---|---|---|---|
| 1 | 9,474 | 2378298 | 180990 | 51,450 | | | |
| 2 | 10,118 | 2244221 | 160770 | 48,550 | | V | |
| Total | | 4622519 | 341760 | | | | |

**Supplementary Figure 106** CSP-HPLC trace of **(*rac*)-2a**

<Chromatogram>

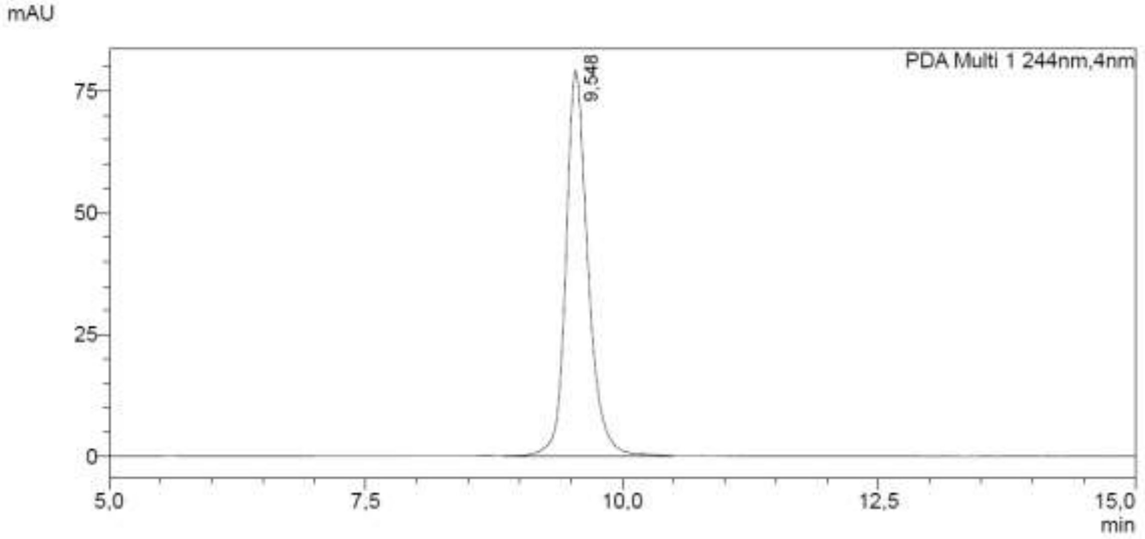


<Peak Table>

PDA Ch1 244nm

| Peak# | Ret. Time | Area | Height | Area% |
|---|---|---|---|---|
| 1 | 9,548 | 1182797 | 79065 | 100,000 |
| Total | | 1182797 | 79065 | 100,000 |

**Supplementary Figure 107** CSP-HPLC trace of **(*S*)-2a** using TMSBr

<Chromatogram>

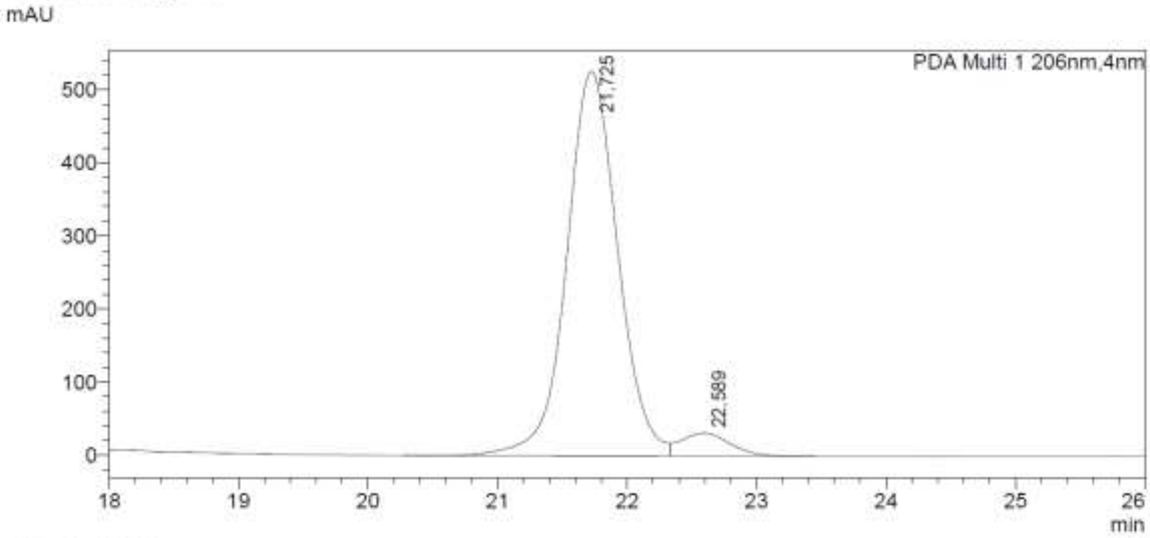


<Peak Table>

PDA Ch1 206nm

| Peak# | Ret. Time | Area | Height | Conc. | Unit | Mark | Name |
|---|---|---|---|---|---|---|---|
| 1 | 21,725 | 14437985 | 526063 | 94,575 | | M | |
| 2 | 22,589 | 828156 | 31406 | 5,425 | | V M | |
| Total | | 15266141 | 557469 | | | | |

**Supplementary Figure 108** CSP-HPLC trace of **(*S*)-2b**

<Chromatogram>

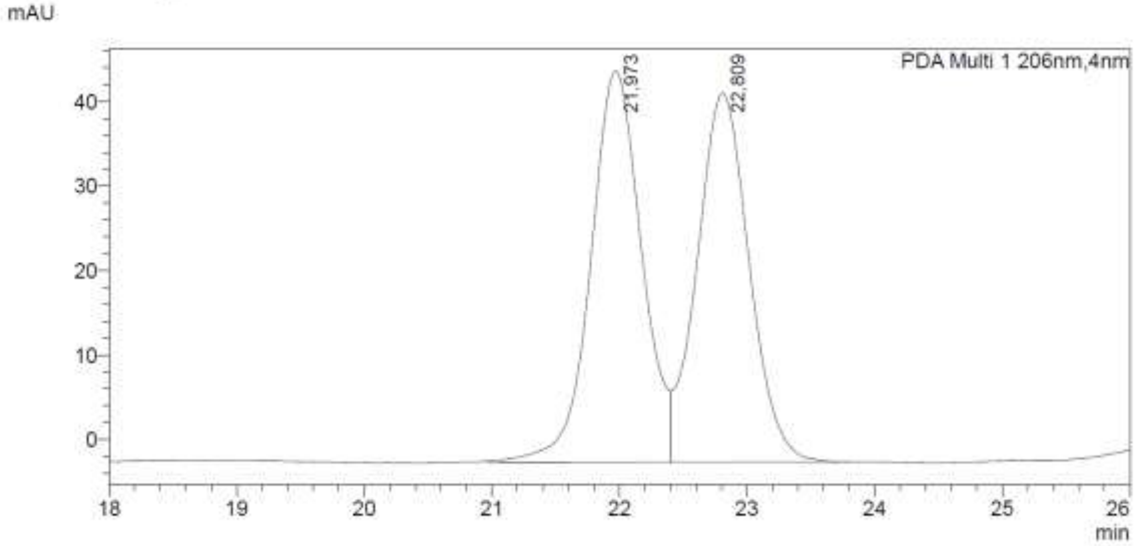


<Peak Table>

PDA Ch1 206nm

| Peak# | Ret. Time | Area | Height | Conc. | Unit | Mark | Name |
|---|---|---|---|---|---|---|---|
| 1 | 21,973 | 1289717 | 46342 | 50,940 | | V | |
| 2 | 22,809 | 1242098 | 43654 | 49,060 | | V | |
| Total | | 2531816 | 89995 | | | | |

**Supplementary Figure 109** CSP-HPLC trace of **(*rac*)-2b**

<Chromatogram>

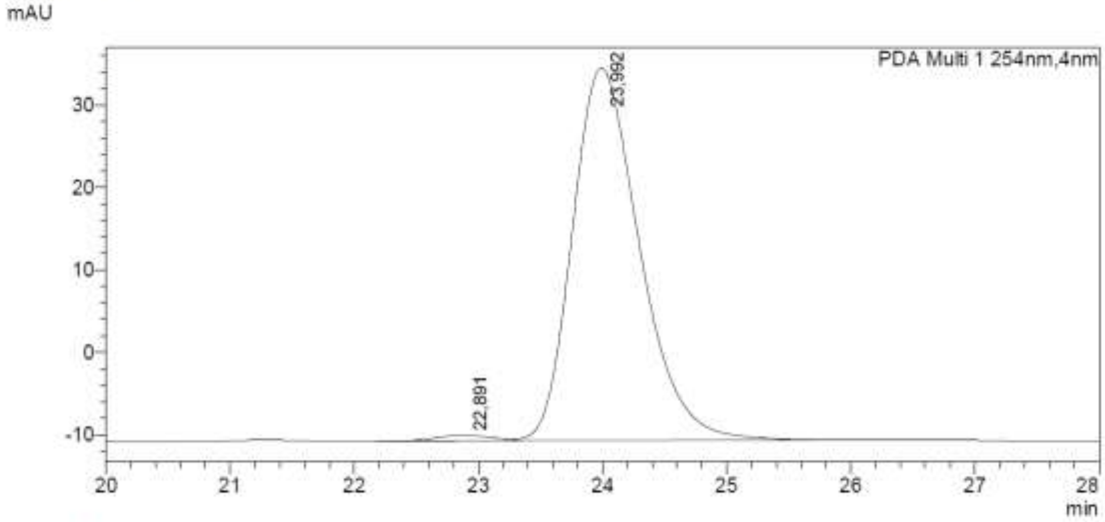


<Peak Table>

PDA Ch1 254nm

| Peak# | Ret. Time | Height | Area | Area% | Conc. | Unit | Mark |
|---|---|---|---|---|---|---|---|
| 1 | 22,891 | 689 | 21000 | 1,217 | 1,217 | | M |
| 2 | 23,992 | 45211 | 1704232 | 98,783 | 98,783 | | V M |
| Total | | 45900 | 1725232 | 100,000 | | | |

**Supplementary Figure 110** CSP-HPLC trace of **(*S*)-2c’**

<Chromatogram>

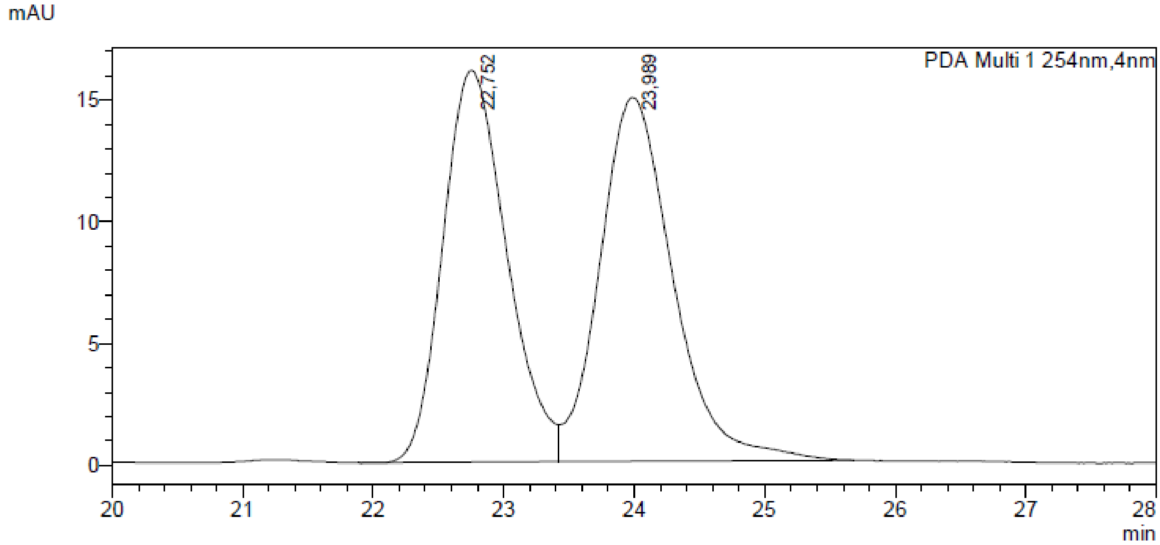


<Peak Table>

PDA Ch1 254nm

| Peak# | Ret. Time | Height | Area | Area% | Conc. | Unit | Mark |
|---|---|---|---|---|---|---|---|
| 1 | 22,752 | 16085 | 547925 | 48,452 | 48,452 | | M |
| 2 | 23,989 | 14944 | 582926 | 51,548 | 51,548 | | V M |
| Total | | 31030 | 1130851 | 100,000 | | | |

**Supplementary Figure 111** CSP-HPLC trace of **(*rac*)-2c’**

## <Chromatogram>

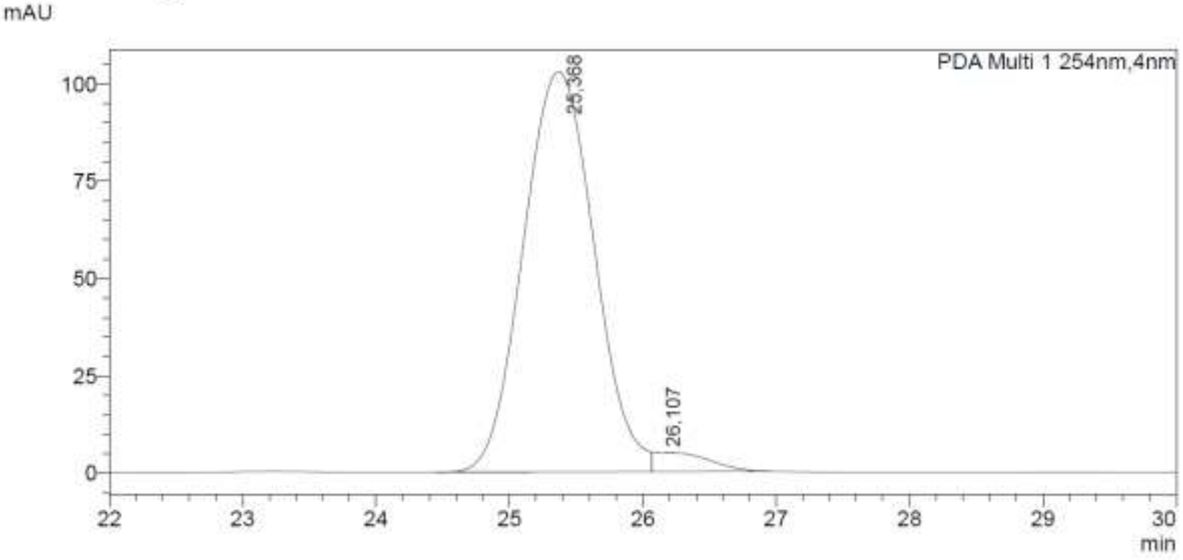


## <Peak Table>

PDA Ch1 254nm

| Peak# | Ret. Time | Area | Height | Conc. | Area% | Unit | Mark |
|---|---|---|---|---|---|---|---|
| 1 | 25,368 | 3797201 | 102757 | 0,000 | 96,505 | | M |
| 2 | 26,107 | 137525 | 4855 | 0,000 | 3,495 | | V M |
| Total | | 3934726 | 107612 | | 100,000 | | |

**Supplementary Figure 112** CSP-HPLC trace of ***(R)*-2d**

## <Chromatogram>

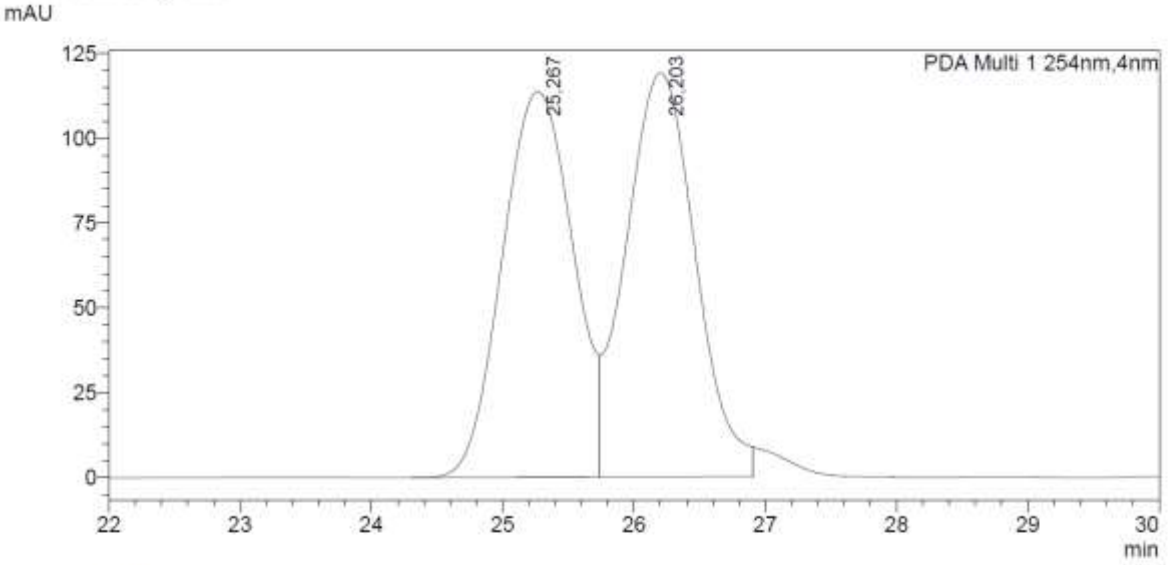


## <Peak Table>

PDA Ch1 254nm

| Peak# | Ret. Time | Area | Height | Conc. | Area% | Unit | Mark |
|---|---|---|---|---|---|---|---|
| 1 | 25,267 | 4260233 | 113645 | 49,268 | 49,268 | | M |
| 2 | 26,203 | 4386850 | 119067 | 50,732 | 50,732 | | V M |
| Total | | 8647083 | 232712 | | 100,000 | | |

**Supplementary Figure 113** CSP-HPLC trace of **(*rac*)-2d**

<Chromatogram>

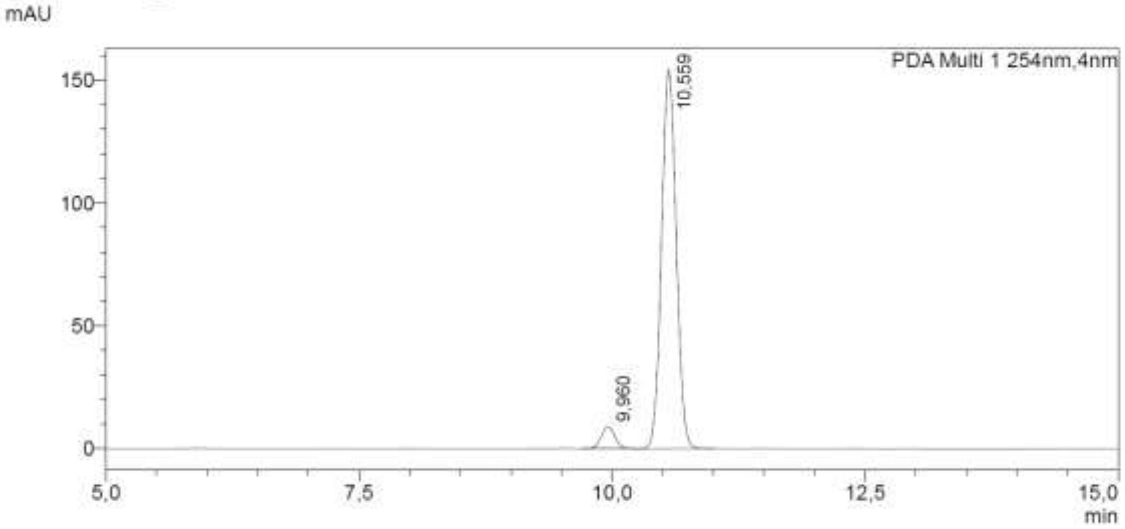


<Peak Table>

PDA Ch1 254nm

| Peak# | Ret. Time | Area | Height | Conc. | Area% | Unit | Mark |
|---|---|---|---|---|---|---|---|
| 1 | 9,960 | 84279 | 8838 | 5,140 | 5,140 | | M |
| 2 | 10,559 | 1555326 | 154202 | 94,860 | 94,860 | | M |
| Total | | 1639605 | 163041 | | 100,000 | | |

**Supplementary Figure 114** CSP-HPLC trace of **(*S*)-4a**

<Chromatogram>

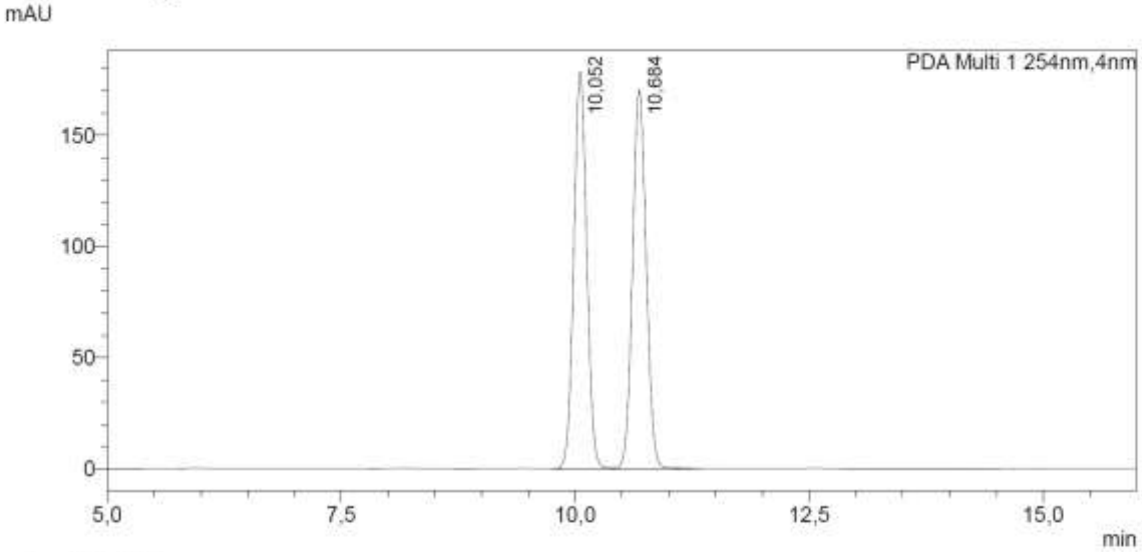


<Peak Table>

PDA Ch1 254nm

| Peak# | Ret. Time | Area | Height | Conc. | Area% | Unit | Mark |
|---|---|---|---|---|---|---|---|
| 1 | 10,052 | 1719689 | 178333 | 49,942 | 49,942 | | |
| 2 | 10,684 | 1723674 | 170484 | 50,058 | 50,058 | | V |
| Total | | 3443363 | 348817 | | 100,000 | | |

**Supplementary Figure 115** CSP-HPLC trace of **(*rac*)-4a**

<Chromatogram>

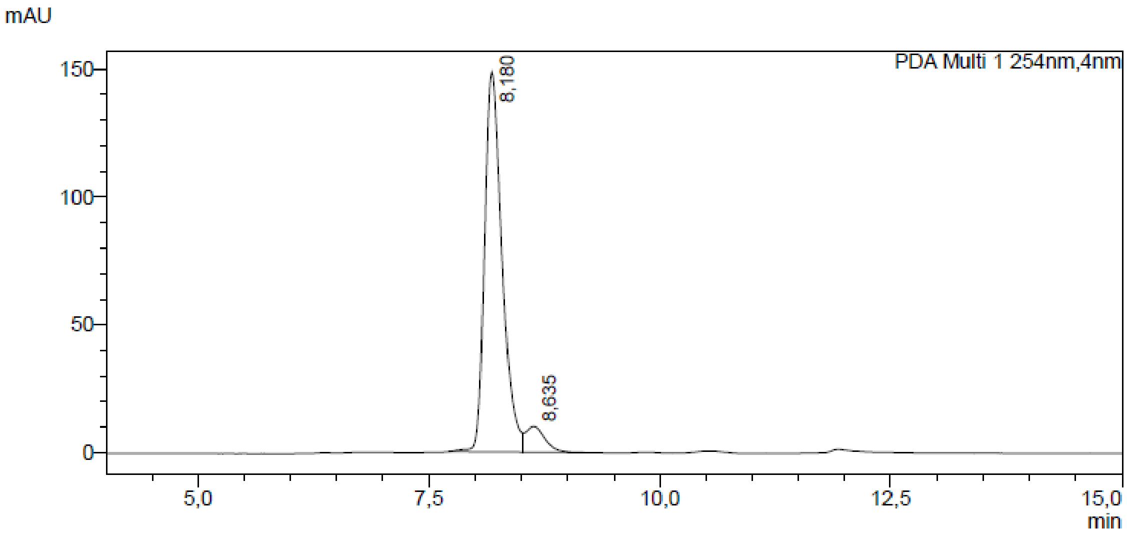


**<Peak Table>**

PDA Ch1 254nm

| Peak# | Ret. Time | Area | Height | Area% |
|---|---|---|---|---|
| 1 | 8,180 | 1977051 | 148493 | 93,131 |
| 2 | 8,635 | 145813 | 10095 | 6,869 |
| Total | | 2122864 | 158588 | 100,000 |

**Supplementary Figure 116** CSP-HPLC trace of **(*S*)-4b**

<Chromatogram>

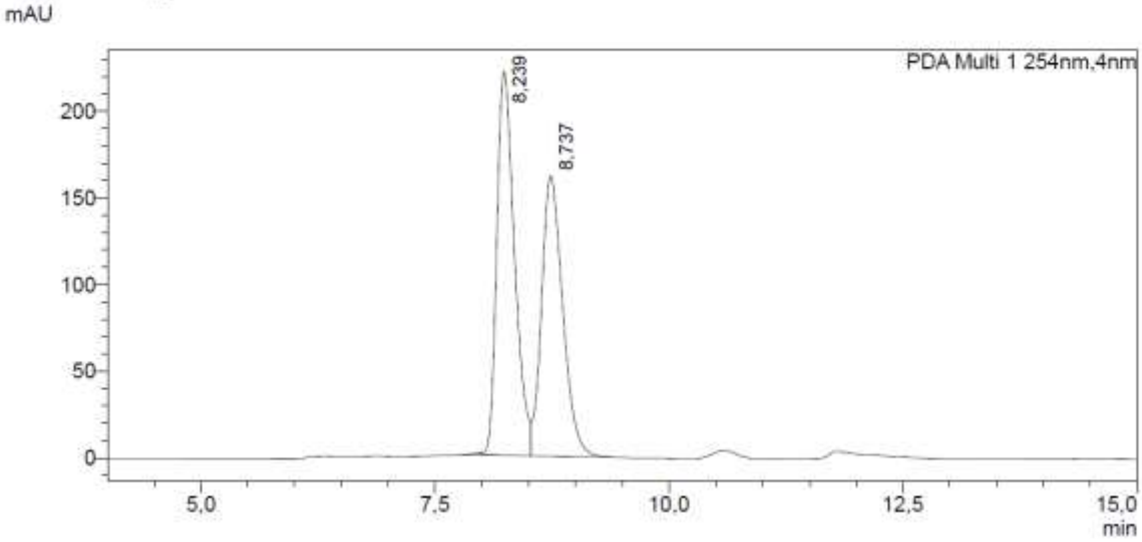


Peak Table

PDA Ch1 254nm

| Peak# | Ret. Time | Area | Height | Area% |
|---|---|---|---|---|
| 1 | 8,239 | 2912684 | 221165 | 53,414 |
| 2 | 8,737 | 2540393 | 161556 | 46,586 |
| Total | | 5453077 | 382722 | 100,000 |

**Supplementary Figure 117** CSP-HPLC trace of a scalemic mixture of **(*S*)-4b** and **(*R*)-4b**

<Chromatogram>

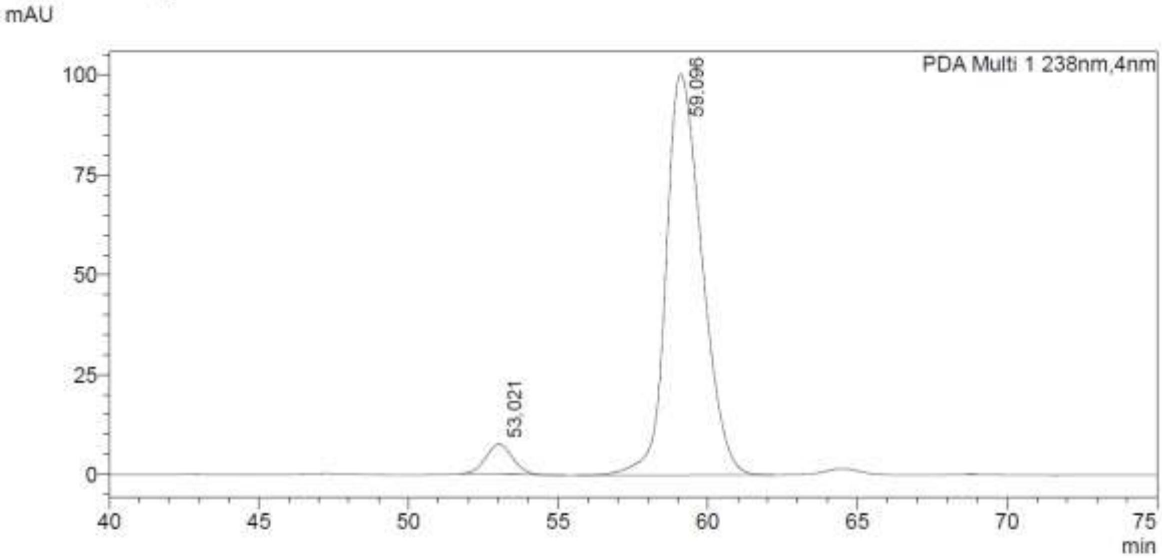


<Peak Table>

PDA Ch1 238nm

| Peak# | Ret. Time | Area | Height | Conc. | Area% | Unit | Mark |
|---|---|---|---|---|---|---|---|
| 1 | 53,021 | 468219 | 7496 | 0,000 | 5,294 | | M |
| 2 | 59,096 | 8376670 | 100410 | 0,000 | 94,706 | | M |
| Total | | 8844890 | 107906 | | 100,000 | | |

**Supplementary Figure 118** CSP-HPLC trace of ***(R)*-4d**

<Chromatogram>

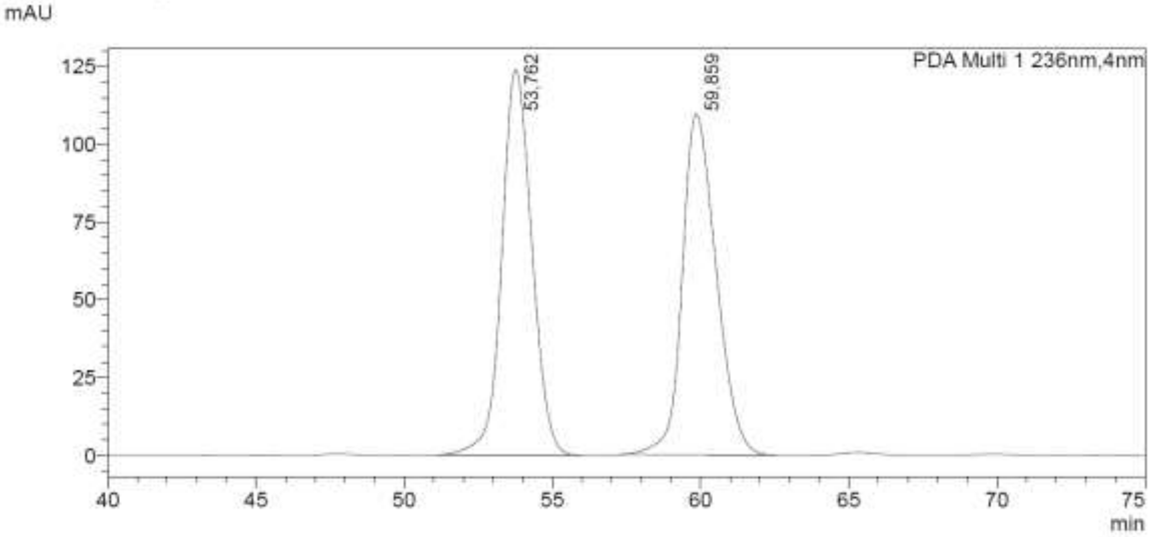


<Peak Table>

PDA Ch1 236nm

| Peak# | Ret. Time | Area | Height | Conc. | Area% | Unit | Mark |
|---|---|---|---|---|---|---|---|
| 1 | 53,762 | 8743721 | 123697 | 49,996 | 49,996 | | |
| 2 | 59,859 | 8745195 | 109418 | 50,004 | 50,004 | | |
| Total | | 17488916 | 233115 | | 100,000 | | |

**Supplementary Figure 119** CSP-HPLC trace of **(*rac*)-4d**

**<Chromatogram>**

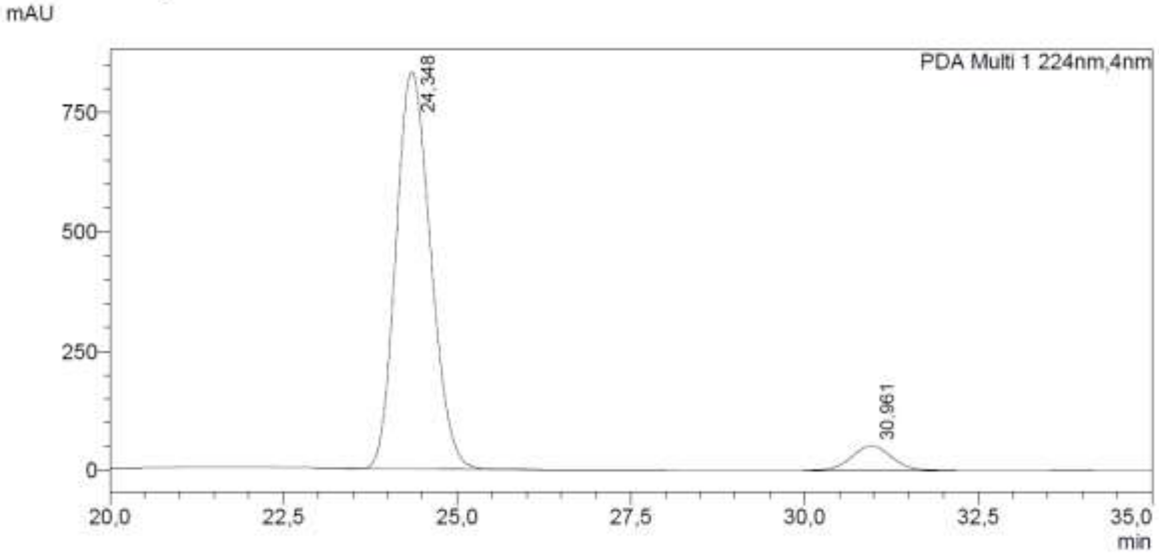


**<Peak Table>**

PDA Ch1 224nm

| Peak# | Ret. Time | Height | Conc. | Area | Area% | Unit | Mark |
|---|---|---|---|---|---|---|---|
| 1 | 24,348 | 830842 | 0,000 | 28917743 | 93,445 | | M |
| 2 | 30,961 | 51181 | 0,000 | 2028677 | 6,555 | | |
| Total | | 882023 | | 30946420 | 100,000 | | |

**Supplementary Figure 120** CSP-HPLC trace of **(*R*)-4e**

**<Chromatogram>**

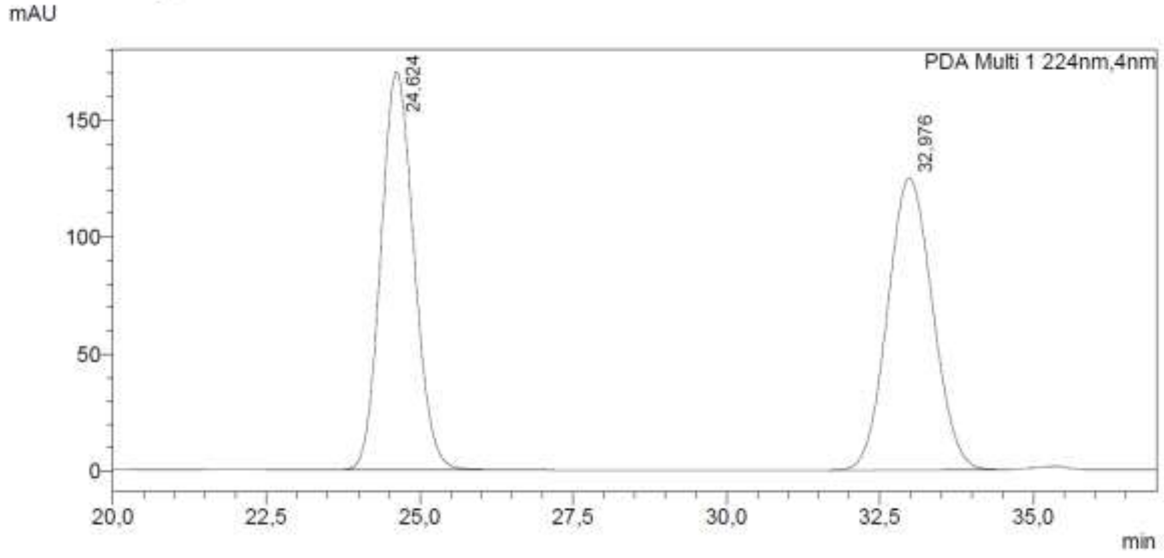


**<Peak Table>**

PDA Ch1 224nm

| Peak# | Ret. Time | Height | Conc. | Area | Area% | Unit | Mark |
|---|---|---|---|---|---|---|---|
| 1 | 24,624 | 170116 | 50,061 | 6380954 | 50,061 | | |
| 2 | 32,976 | 124704 | 49,939 | 6365512 | 49,939 | | |
| Total | | 294820 | | 12746466 | 100,000 | | |

**Supplementary Figure 121** CSP-HPLC trace of **(*rac*)-4e**

## <Chromatogram>

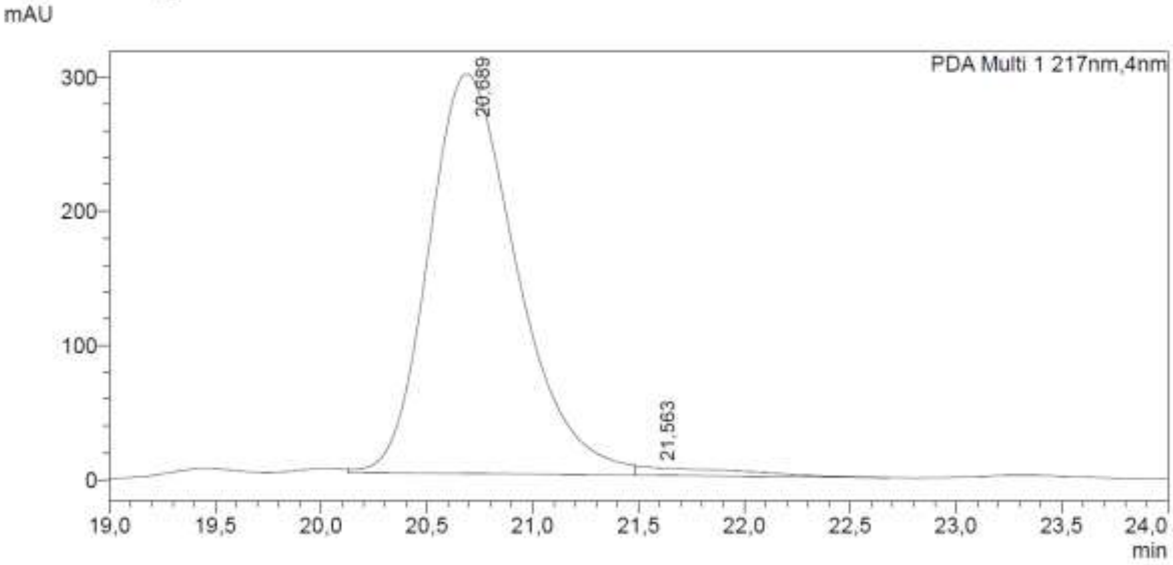


## <Peak Table>

PDA Ch1 217nm

| Peak# | Ret. Time | Height | Conc. | Area | Area% | Unit | Mark |
|---|---|---|---|---|---|---|---|
| 1 | 20,689 | 297584 | 0,000 | 8620585 | 97,695 | | M |
| 2 | 21,563 | 5983 | 0,000 | 203373 | 2,305 | | V M |
| Total | | 303568 | | 8823958 | 100,000 | | |

**Supplementary Figure 122** CSP-HPLC trace of **(*R*)-4f**

## <Chromatogram>

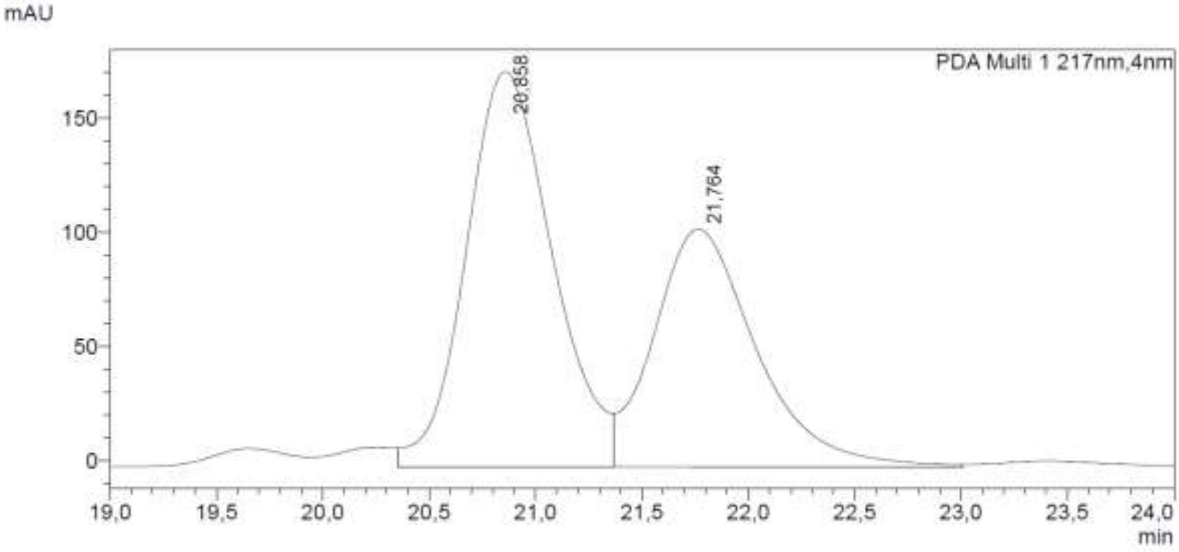


## <Peak Table>

PDA Ch1 217nm

| Peak# | Ret. Time | Height | Conc. | Area | Area% | Unit | Mark |
|---|---|---|---|---|---|---|---|
| 1 | 20,858 | 172941 | 58,425 | 4886447 | 58,425 | | V |
| 2 | 21,764 | 103988 | 41,575 | 3477181 | 41,575 | | V |
| Total | | 276930 | | 8363629 | 100,000 | | |

**Supplementary Figure 123** CSP-HPLC trace of **(*S*)-4f** and **(*R*)-4f**

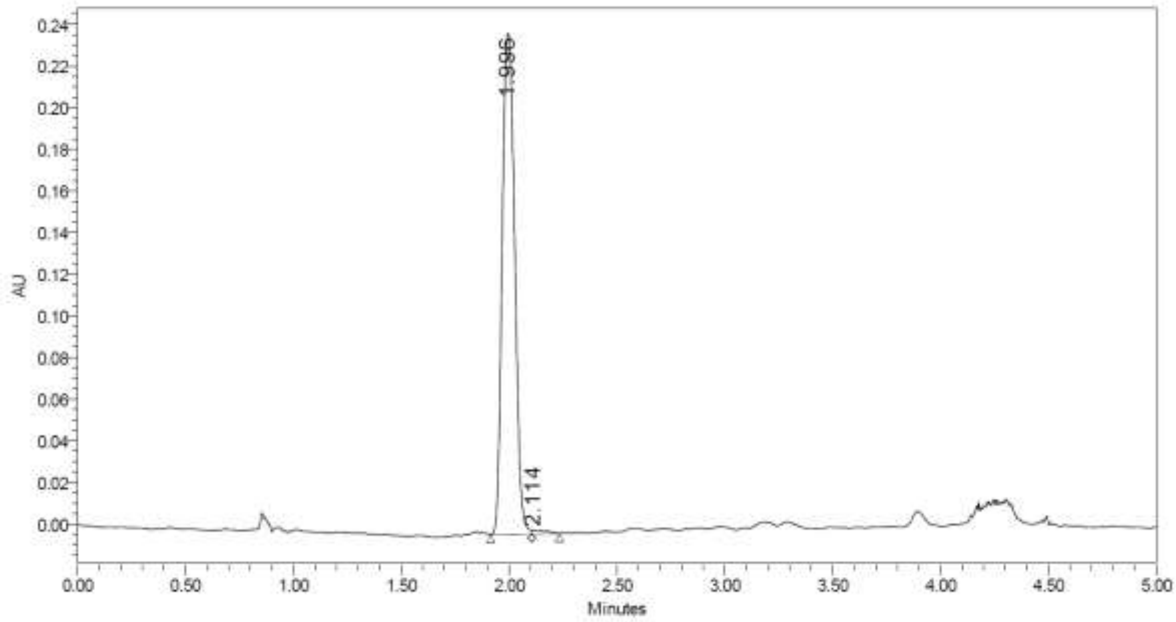


| | RT | Area | Height | % Area |
|---|---|---|---|---|
| 1 | 1.996 | 965300 | 240789 | 99.12 |
| 2 | 2.114 | 8612 | 1837 | 0.88 |

**Supplementary Figure 124** Chiral SFC trace of **(*R*)-4g**

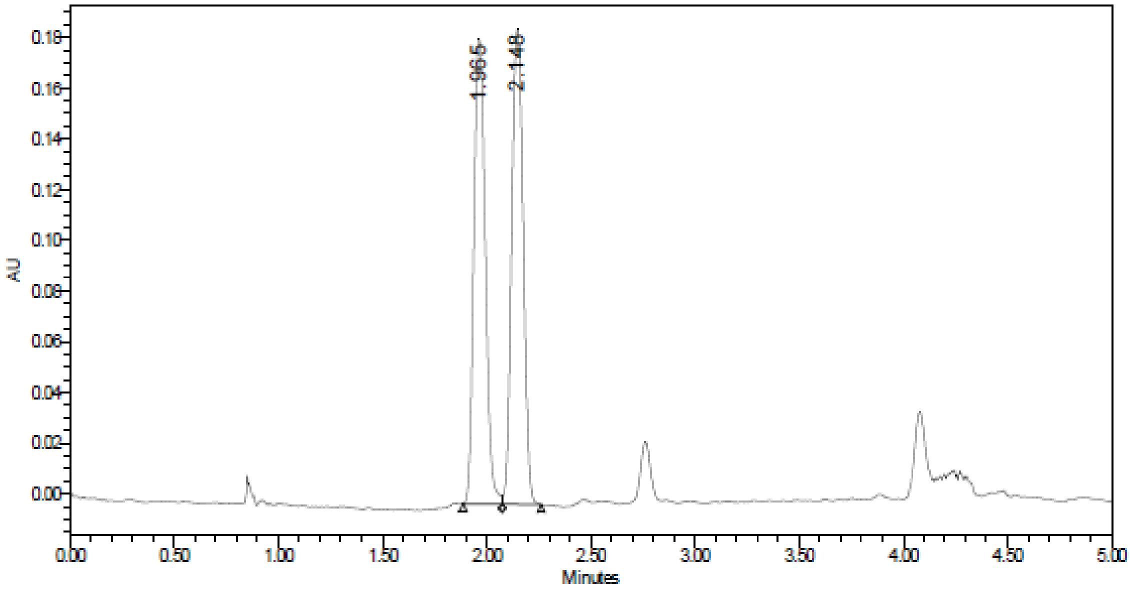


| | RT | Area | Height | % Area |
|---|---|---|---|---|
| 1 | 1.965 | 697099 | 183026 | 49.36 |
| 2 | 2.148 | 715107 | 187337 | 50.64 |

**Supplementary Figure 125** Chiral SFC trace of **(*rac*)-4g**

**<Chromatogram>**

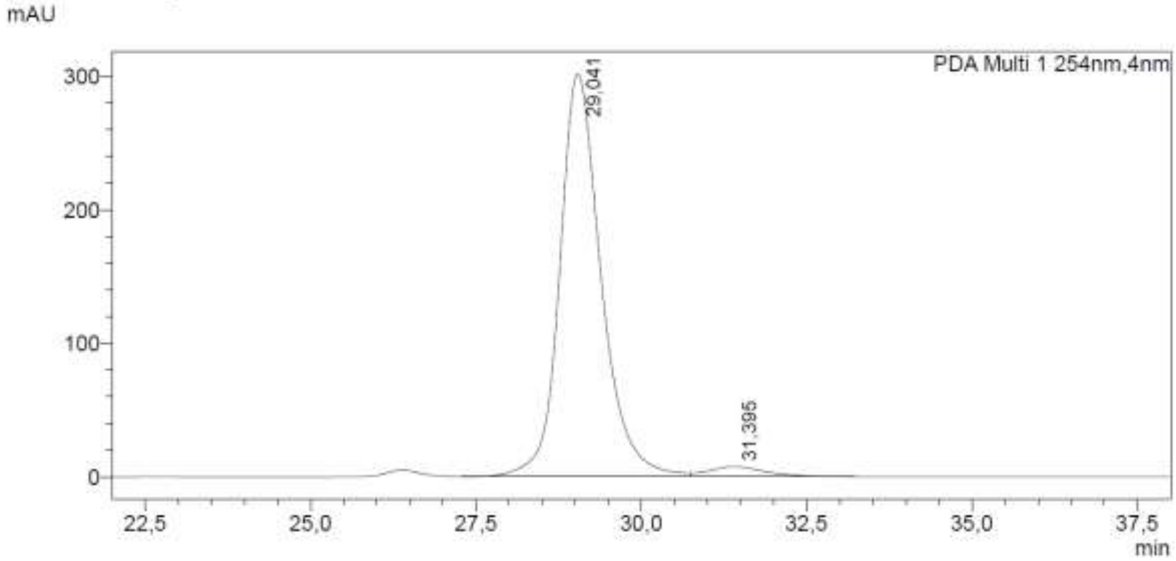


**<Peak Table>**

PDA Ch1 254nm

| Peak# | Ret. Time | Area | Height | Conc. | Area% | Unit | Mark |
|---|---|---|---|---|---|---|---|
| 1 | 29,041 | 13107240 | 301653 | 0,000 | 96,795 | | M |
| 2 | 31,395 | 434050 | 7450 | 0,000 | 3,205 | | V M |
| Total | | 13541290 | 309103 | | 100,000 | | |

**Supplementary Figure 126** CSP-HPLC trace of **(*S*)-5a**

**<Chromatogram>**

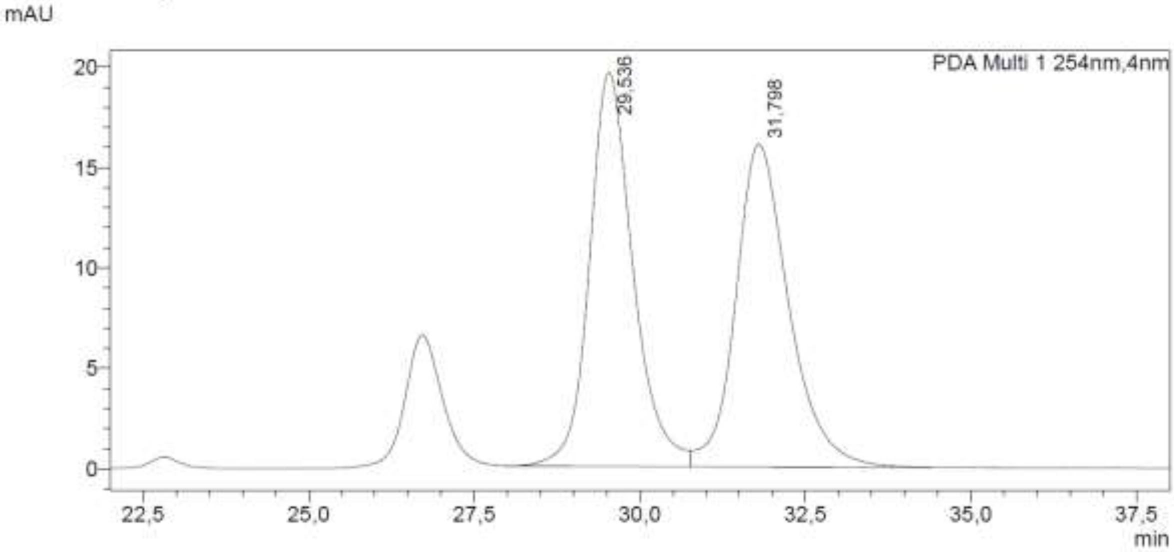


**<Peak Table>**

PDA Ch1 254nm

| Peak# | Ret. Time | Area | Height | Conc. | Area% | Unit | Mark |
|---|---|---|---|---|---|---|---|
| 1 | 29,536 | 898891 | 19624 | 0,000 | 49,976 | | M |
| 2 | 31,798 | 899737 | 16086 | 0,000 | 50,024 | | V M |
| Total | | 1798628 | 35709 | | 100,000 | | |

**Supplementary Figure 127** CSP-HPLC trace of **(*rac*)-5a**

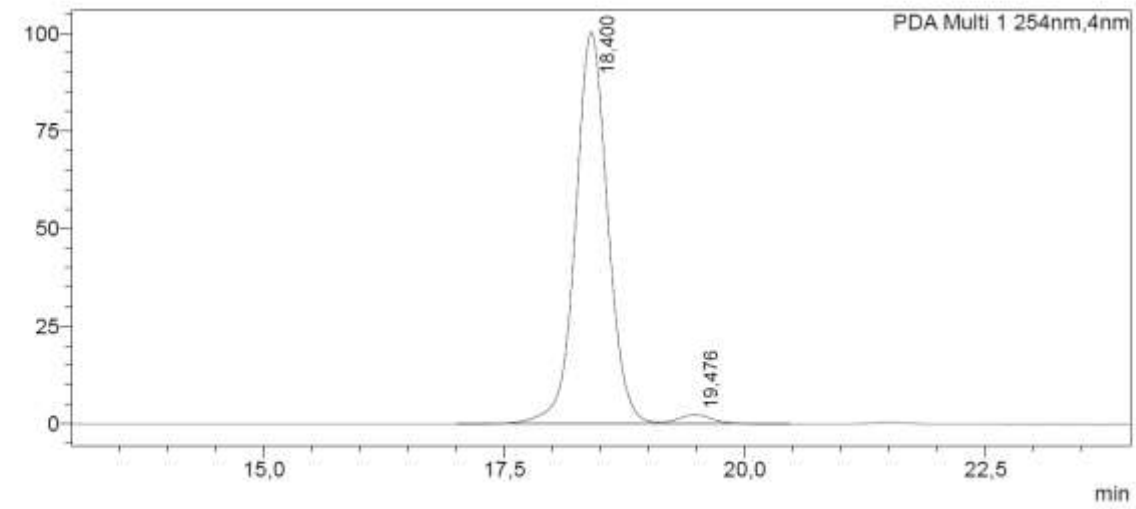


<Peak Table>

PDA Ch1 254nm

| Peak# | Ret. Time | Area | Height | Conc. | Area% | Unit | Mark |
|---|---|---|---|---|---|---|---|
| 1 | 18,400 | 2340591 | 100412 | 97,505 | 97,505 | | M |
| 2 | 19,476 | 59886 | 2391 | 2,495 | 2,495 | | V M |
| Total | | 2400478 | 102802 | | 100,000 | | |

**Supplementary Figure 128** CSP-HPLC trace of **(*S*)-5b**

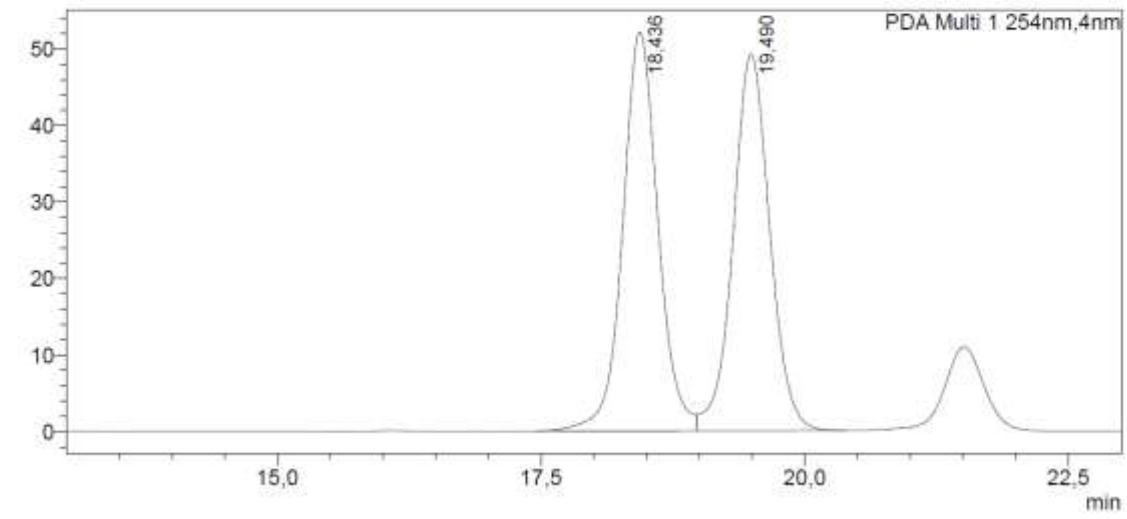


<Peak Table>

PDA Ch1 254nm

| Peak# | Ret. Time | Area | Height | Conc. | Area% | Mark | Unit |
|---|---|---|---|---|---|---|---|
| 1 | 18,436 | 1215123 | 52120 | 50,509 | 50,509 | | |
| 2 | 19,490 | 1190632 | 49261 | 49,491 | 49,491 | V | |
| Total | | 2405755 | 101381 | | 100,000 | | |

**Supplementary Figure 129** CSP-HPLC trace of **(*rac*)-5b**

<Chromatogram>

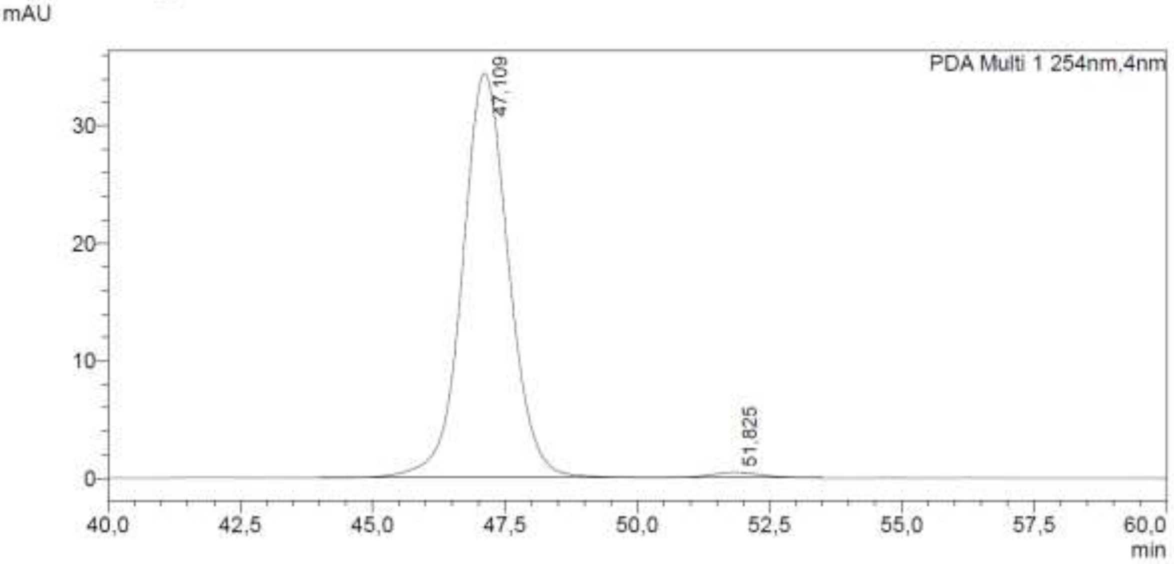


<Peak Table>

PDA Ch1 254nm

| Peak# | Ret. Time | Area | Height | Conc. | Area% | Unit | Mark |
|---|---|---|---|---|---|---|---|
| 1 | 47,109 | 2099120 | 34433 | 0,000 | 98,520 | | M |
| 2 | 51,825 | 31529 | 470 | 0,000 | 1,480 | | V M |
| Total | | 2130649 | 34902 | | 100,000 | | |

**Supplementary Figure 130** CSP-HPLC trace of **(*S*)-5c**

<Chromatogram>

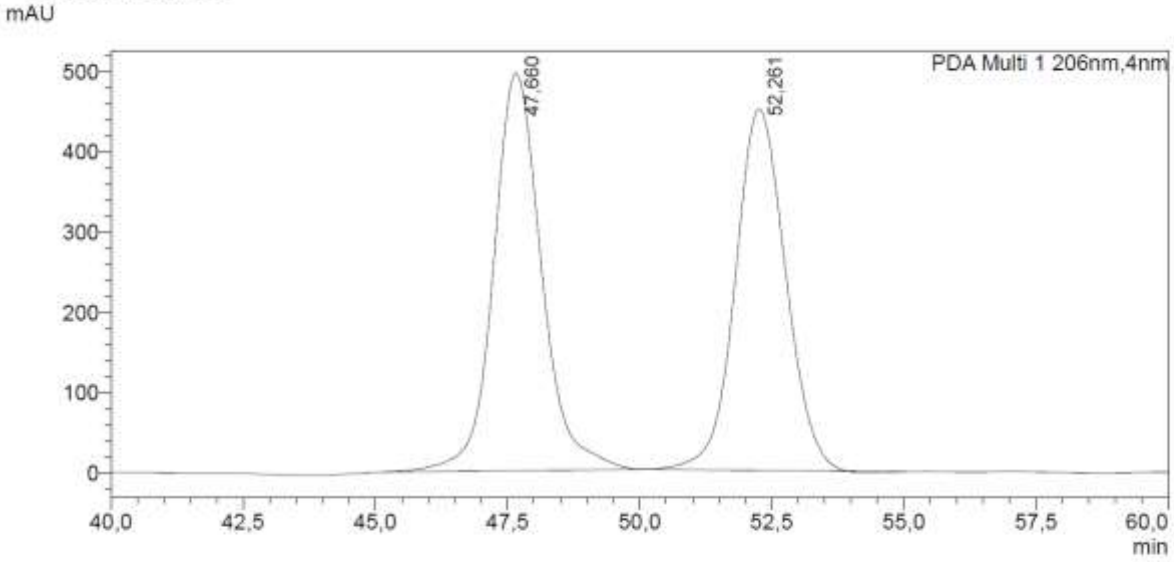


<Peak Table>

PDA Ch1 206nm

| Peak# | Ret. Time | Area | Height | Conc. | Area% | Unit | Mark |
|---|---|---|---|---|---|---|---|
| 1 | 47,660 | 31382080 | 495723 | 51,244 | 51,244 | | M |
| 2 | 52,261 | 29857928 | 450003 | 48,756 | 48,756 | | M |
| Total | | 61240007 | 945726 | | 100,000 | | |

**Supplementary Figure 131** CSP-HPLC trace of **(*rac*)-5c**

## <Chromatogram>

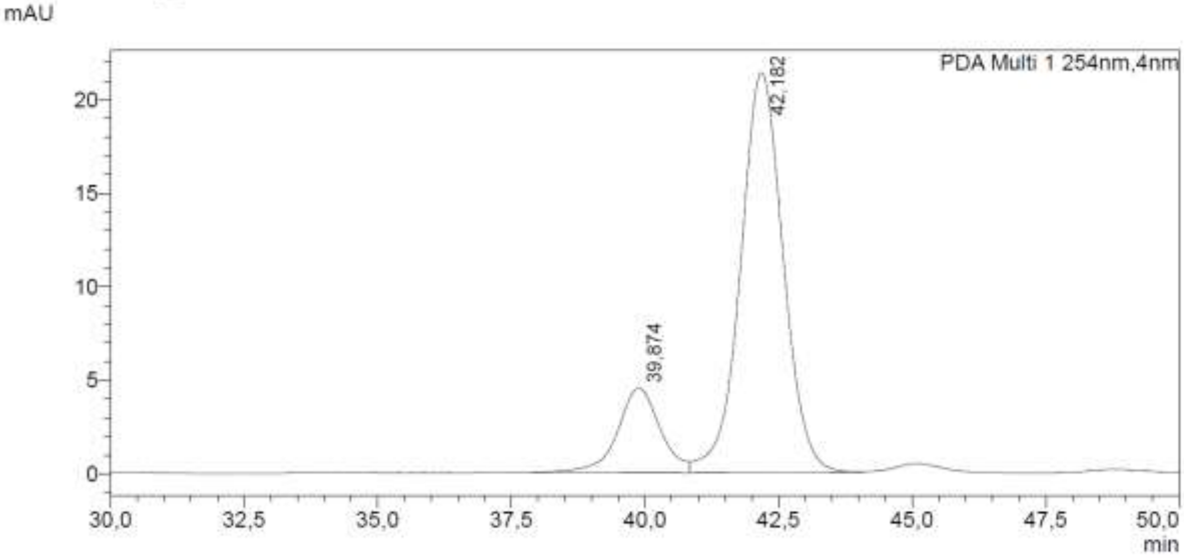


## <Peak Table>

PDA Ch1 254nm

| Peak# | Ret. Time | Area | Height | Conc. | Area% | Unit | Mark |
|---|---|---|---|---|---|---|---|
| 1 | 39,874 | 251499 | 4525 | 0,000 | 17,466 | | M |
| 2 | 42,182 | 1188444 | 21377 | 0,000 | 82,534 | | M |
| Total | | 1439943 | 25902 | | 100,000 | | |

**Supplementary Figure 132** CSP-HPLC trace of **(*S*)-5d**

## <Chromatogram>

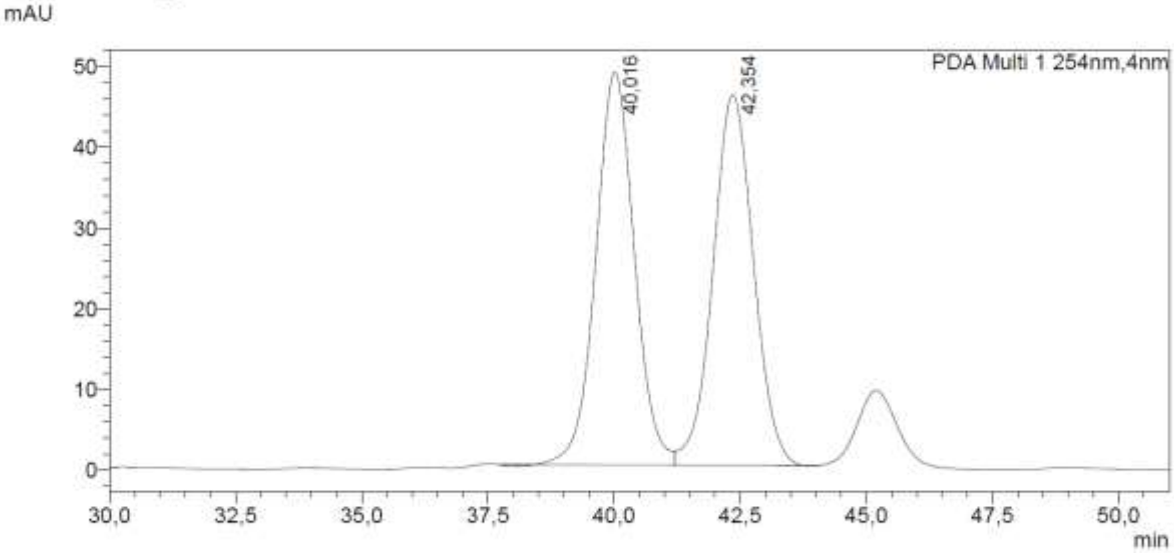


## <Peak Table>

PDA Ch1 254nm

| Peak# | Ret. Time | Area | Height | Conc. | Area% | Unit | Mark |
|---|---|---|---|---|---|---|---|
| 1 | 40,016 | 2589721 | 48611 | 50,238 | 50,238 | | M |
| 2 | 42,354 | 2565186 | 45880 | 49,762 | 49,762 | | V M |
| Total | | 5154907 | 94491 | | 100,000 | | |

**Supplementary Figure 133** CSP-HPLC trace of **(*rac*)-5d**

<Chromatogram>

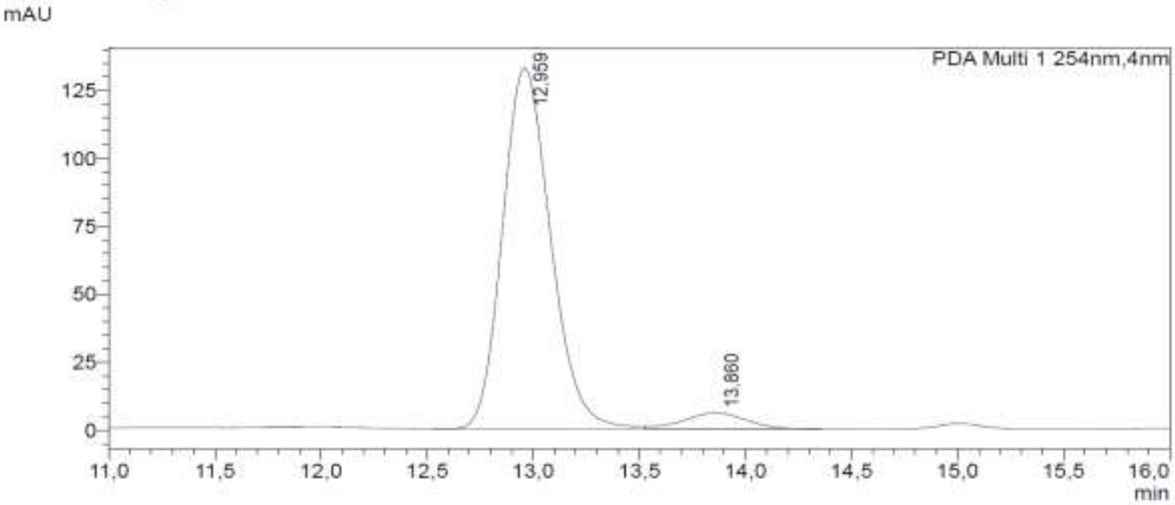


<Peak Table>

PDA Ch1 254nm

| Peak# | Ret. Time | Area | Height | Conc. | Area% | Unit | Mark |
|---|---|---|---|---|---|---|---|
| 1 | 12,959 | 2098970 | 132926 | 94,471 | 94,471 | | M |
| 2 | 13,860 | 122850 | 5928 | 5,529 | 5,529 | | V M |
| Total | | 2221820 | 138854 | | 100,000 | | |

**Supplementary Figure 134** CSP-HPLC trace of **(*R*)-5e**

<Chromatogram>

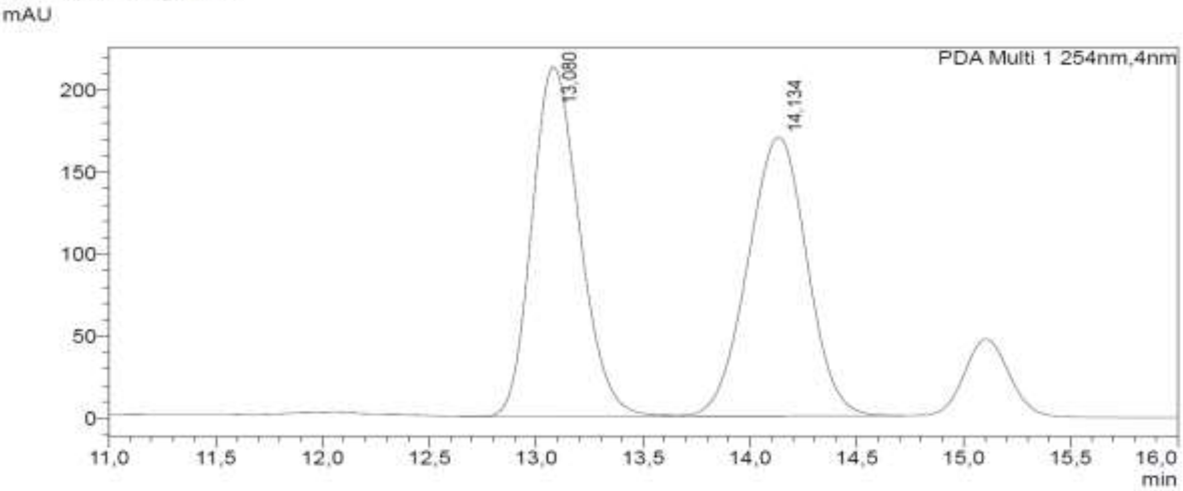


<Peak Table>

PDA Ch1 254nm

| Peak# | Ret. Time | Area | Height | Conc. | Area% | Unit | Mark |
|---|---|---|---|---|---|---|---|
| 1 | 13,080 | 3299519 | 213200 | 50,223 | 50,223 | | M |
| 2 | 14,134 | 3270221 | 170088 | 49,777 | 49,777 | | V M |
| Total | | 6569740 | 383289 | | 100,000 | | |

**Supplementary Figure 135** CSP-HPLC trace of **(*rac*)-5e**

<Chromatogram>

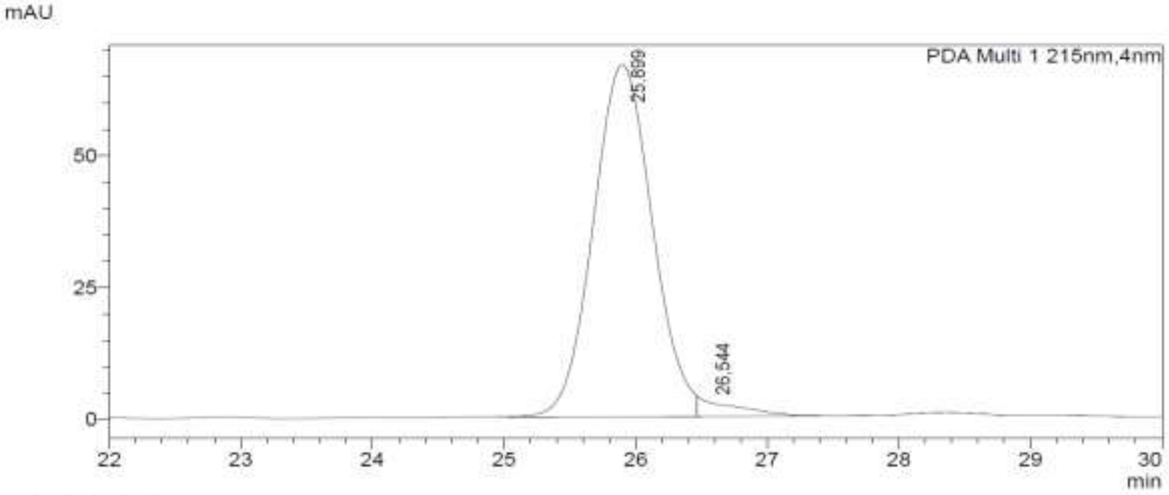


<Peak Table>

PDA Ch1 215nm

| Peak# | Ret. Time | Area | Height | Conc. | Area% | Unit | Mark |
|---|---|---|---|---|---|---|---|
| 1 | 25,899 | 2087359 | 66777 | 96,819 | 96,819 | | V M |
| 2 | 26,544 | 68577 | 2827 | 3,181 | 3,181 | | V M |
| Total | | 2155935 | 69604 | | 100,000 | | |

**Supplementary Figure 136** CSP-HPLC trace of **(*S*)-5f**

<Chromatogram>

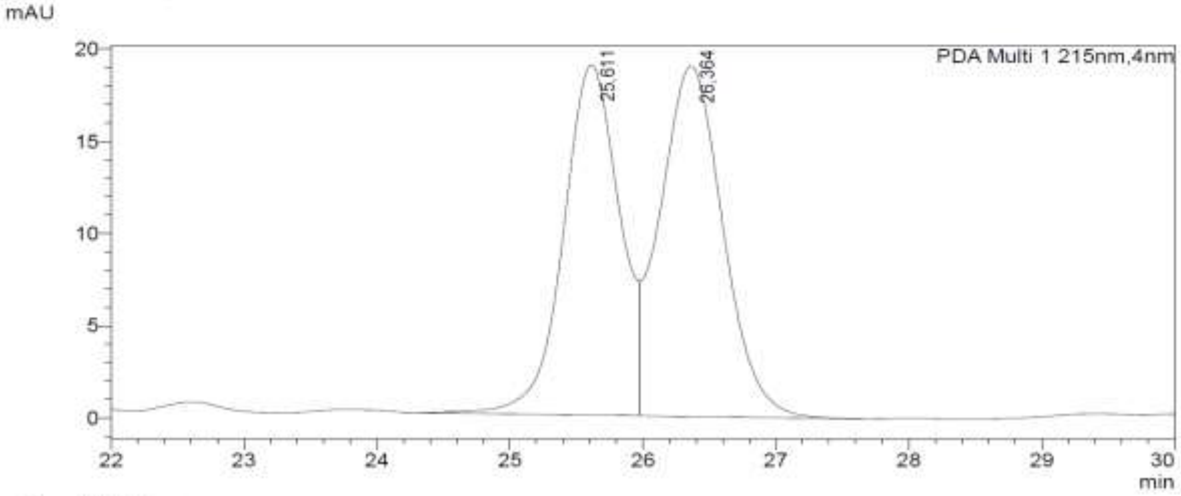


<Peak Table>

PDA Ch1 215nm

| Peak# | Ret. Time | Area | Height | Conc. | Area% | Unit | Mark |
|---|---|---|---|---|---|---|---|
| 1 | 25,611 | 559312 | 18977 | 47,213 | 47,213 | | M |
| 2 | 26,364 | 625338 | 18992 | 52,787 | 52,787 | | V M |
| Total | | 1184650 | 37969 | | 100,000 | | |

**Supplementary Figure 137** CSP-HPLC trace of **(*rac*)-5f**

<Chromatogram>

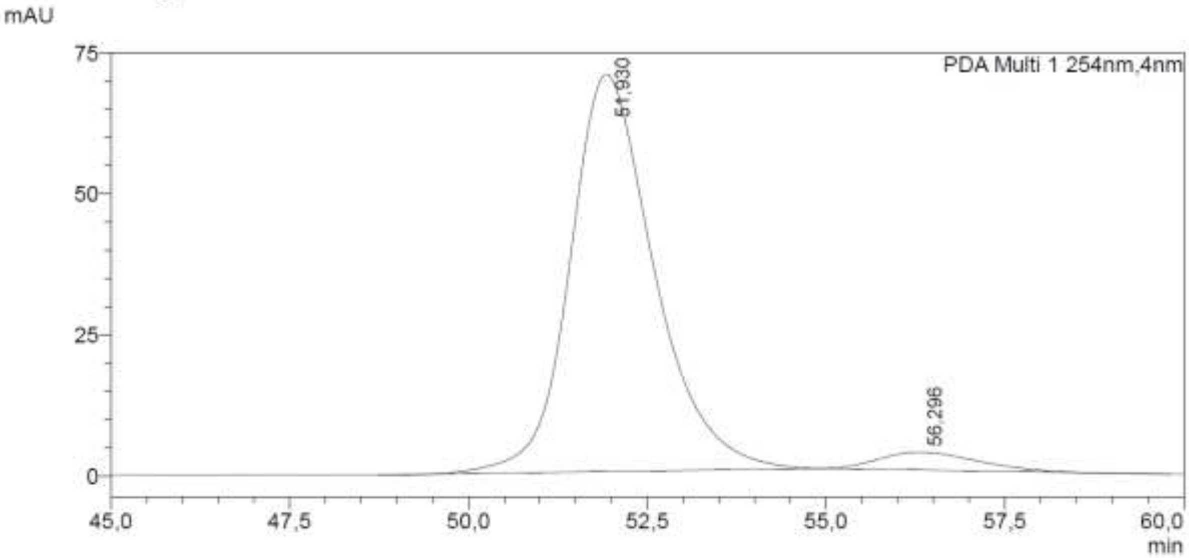


<Peak Table>

PDA Ch1 254nm

| Peak# | Ret. Time | Area | Height | Conc. | Area% | Unit | Mark |
|---|---|---|---|---|---|---|---|
| 1 | 51,930 | 5780379 | 70357 | 0,000 | 95,057 | | M |
| 2 | 56,296 | 300598 | 3156 | 0,000 | 4,943 | | M |
| Total | | 6080977 | 73513 | | 100,000 | | |

**Supplementary Figure 138** CSP-HPLC trace of **(*R*)-5g**

<Chromatogram>

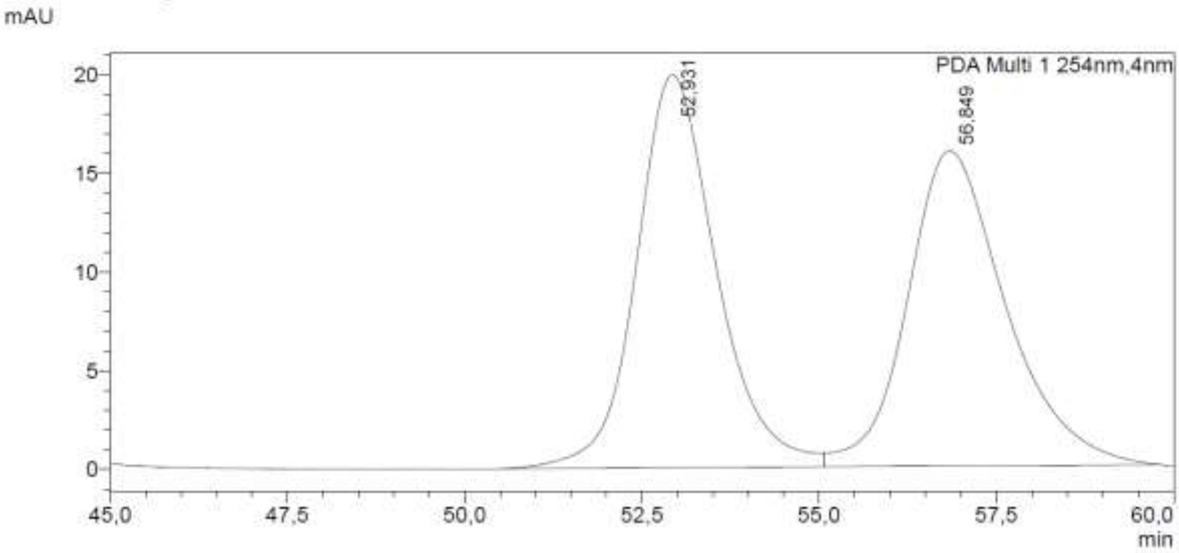


<Peak Table>

PDA Ch1 254nm

| Peak# | Ret. Time | Area | Height | Conc. | Area% | Unit | Mark |
|---|---|---|---|---|---|---|---|
| 1 | 52,931 | 1559903 | 19894 | 0,000 | 50,346 | | M |
| 2 | 56,849 | 1538477 | 15954 | 0,000 | 49,654 | | V M |
| Total | | 3098380 | 35848 | | 100,000 | | |

**Supplementary Figure 139** CSP-HPLC trace of **(*rac*)-5g**

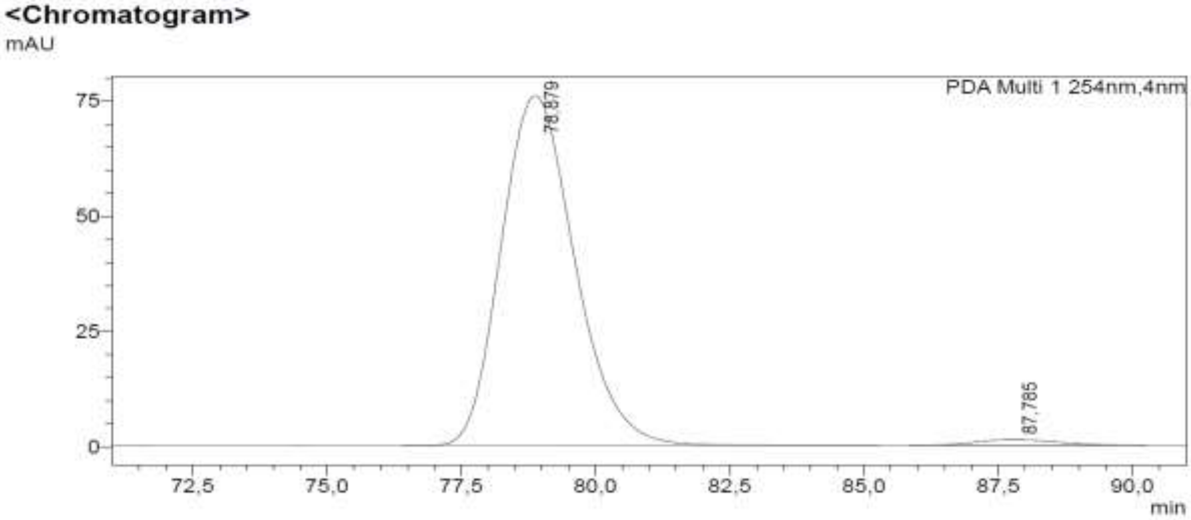


<Peak Table>

PDA Ch1 254nm

| Peak# | Ret. Time | Area | Height | Conc. | Area% | Unit | Mark |
|---|---|---|---|---|---|---|---|
| 1 | 78,879 | 7234022 | 75786 | 0,000 | 98,219 | | M |
| 2 | 87,785 | 131168 | 1258 | 0,000 | 1,781 | | M |
| Total | | 7365190 | 77043 | | 100,000 | | |

**Supplementary Figure 140** CSP-HPLC trace of **(*S*)-5h**

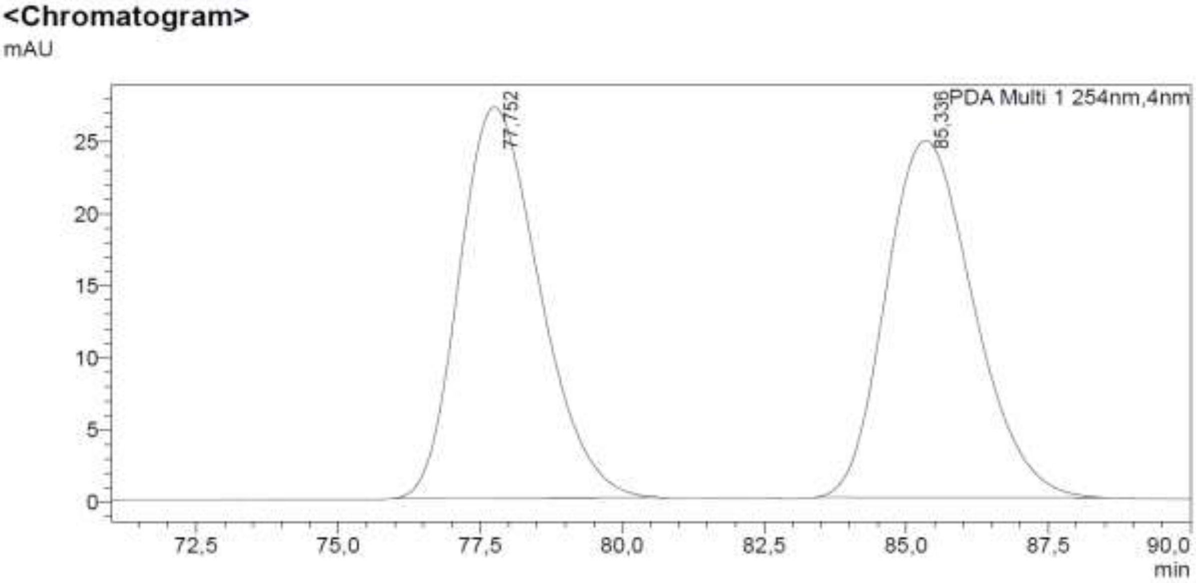


<Peak Table>

PDA Ch1 254nm

| Peak# | Ret. Time | Area | Height | Conc. | Area% | Unit | Mark |
|---|---|---|---|---|---|---|---|
| 1 | 77,752 | 2659436 | 27082 | 0,000 | 50,018 | | |
| 2 | 85,336 | 2657536 | 24710 | 0,000 | 49,982 | | |
| Total | | 5316971 | 51791 | | 100,000 | | |

**Supplementary Figure 141** CSP-HPLC trace of **(*rac*)-5h**

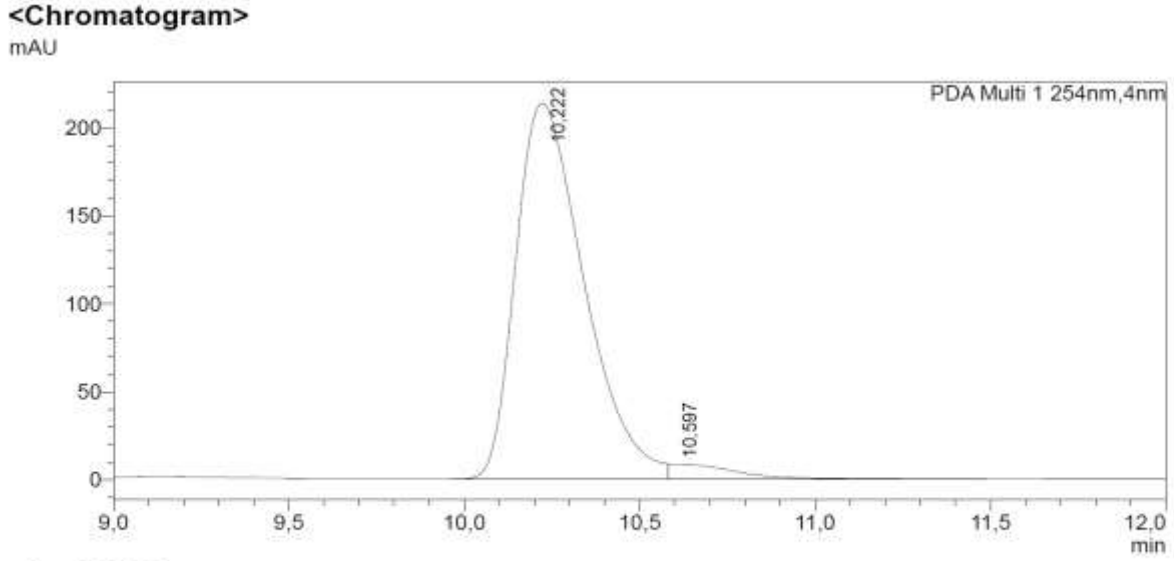


**<Peak Table>**

PDA Ch1 254nm

| Peak# | Ret. Time | Area | Height | Conc. | Area% | Unit | Mark |
|---|---|---|---|---|---|---|---|
| 1 | 10,222 | 2894073 | 213589 | 96,560 | 96,560 | | M |
| 2 | 10,597 | 103095 | 8218 | 3,440 | 3,440 | | V M |
| Total | | 2997168 | 221807 | | 100,000 | | |

**Supplementary Figure 142** CSP-HPLC trace of **(*R*)-5i**

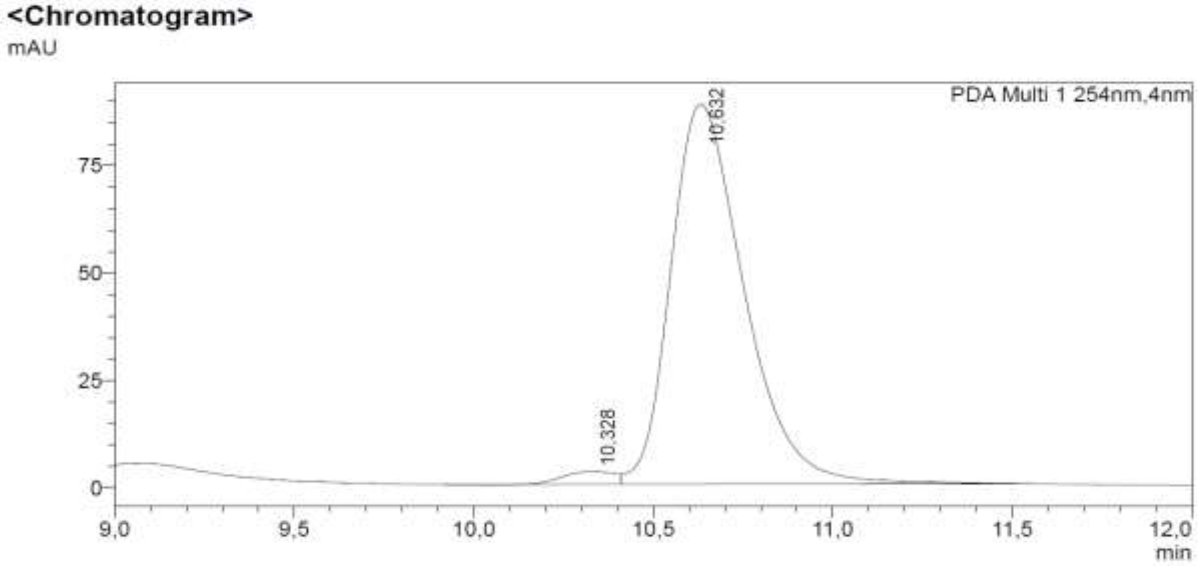


**<Peak Table>**

PDA Ch1 254nm

| Peak# | Ret. Time | Area | Height | Conc. | Area% | Unit | Mark |
|---|---|---|---|---|---|---|---|
| 1 | 10,328 | 30371 | 3050 | 2,357 | 2,357 | | M |
| 2 | 10,632 | 1257957 | 88420 | 97,643 | 97,643 | | V M |
| Total | | 1288328 | 91470 | | 100,000 | | |

**Supplementary Figure 143** CSP-HPLC trace of **(*S*)-5i**

## <Chromatogram>

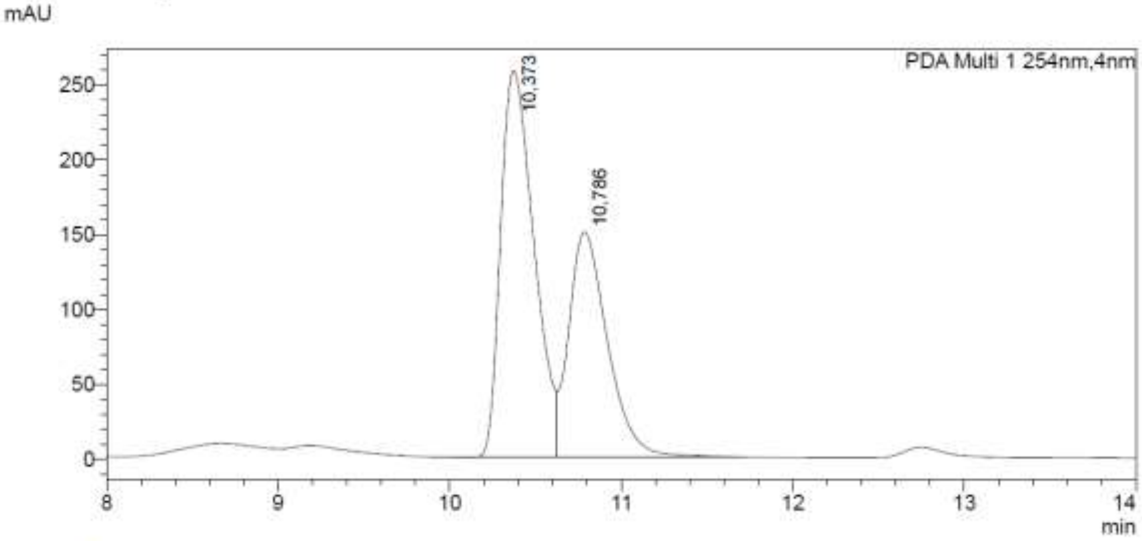


## <Peak Table>

PDA Ch1 254nm

| Peak# | Ret. Time | Area | Height | Conc. | Area% | Unit | Mark |
|---|---|---|---|---|---|---|---|
| 1 | 10,373 | 3486297 | 257884 | 59,691 | 59,691 | | M |
| 2 | 10,786 | 2354324 | 150467 | 40,309 | 40,309 | | V M |
| Total | | 5840621 | 408350 | | 100,000 | | |

**Supplementary Figure 144** CSP-HPLC trace of a scalemic mixture of ***(R)*-5i** and ***(S)*-5i**

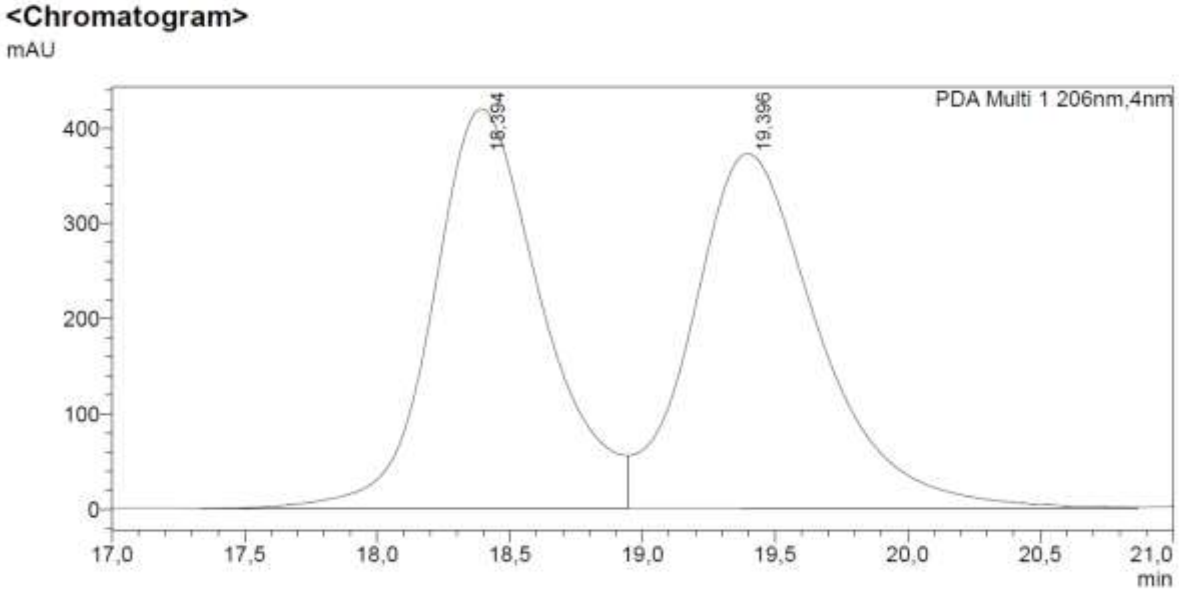


<Peak Table>

PDA Ch1 206nm

| Peak# | Ret. Time | Area | Height | Area% | Mark |
|---|---|---|---|---|---|
| 1 | 18,394 | 11980732 | 419891 | 49,070 | V |
| 2 | 19,396 | 12434841 | 373055 | 50,930 | V |
| Total | | 24415573 | 792947 | 100,000 | |

**Supplementary Figure 145** CSP-HPLC trace of **(*R*)-5j**

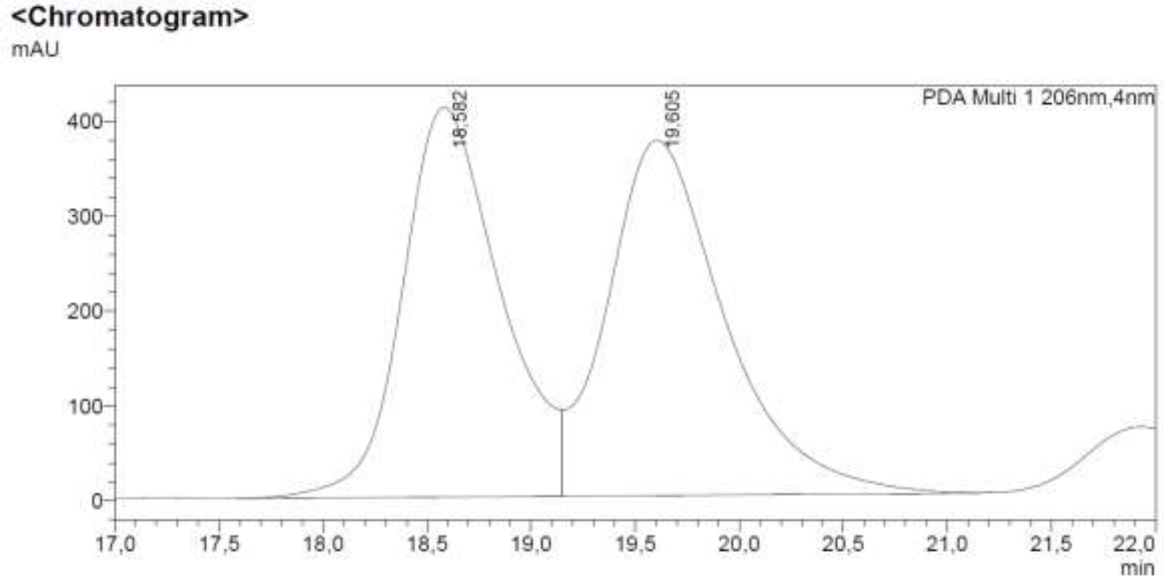


<Peak Table>

PDA Ch1 206nm

| Peak# | Ret. Time | Area | Height | Area% | Mark |
|---|---|---|---|---|---|
| 1 | 18,582 | 13502290 | 411463 | 47,809 | |
| 2 | 19,605 | 14739621 | 374680 | 52,191 | V |
| Total | | 28241910 | 786143 | 100,000 | |

**Supplementary Figure 146** CSP-HPLC trace of **(*rac*)-5j**

<Chromatogram>

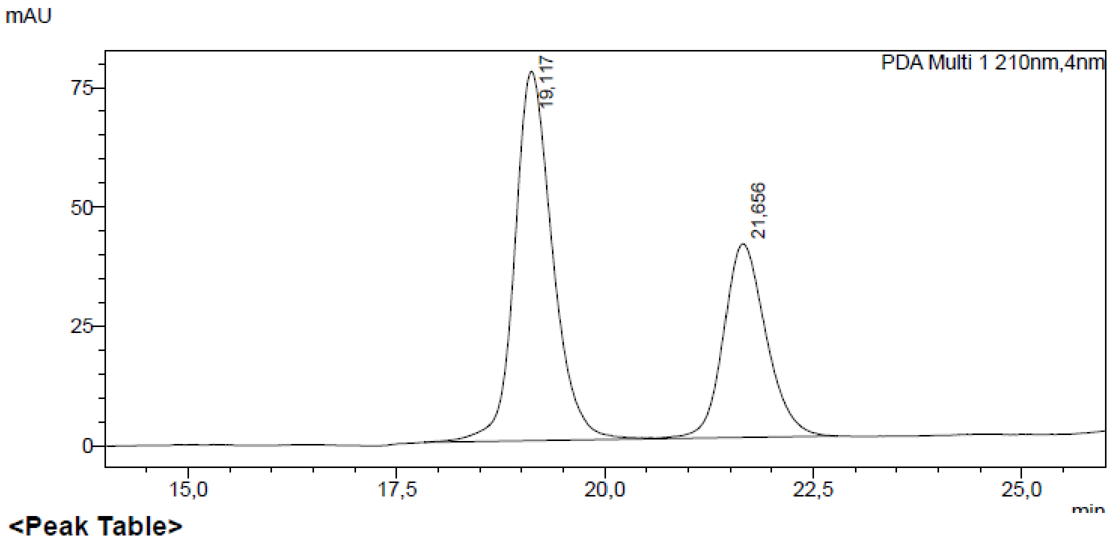


<Peak Table>

PDA Ch1 210nm

| Peak# | Ret. Time | Area | Height | Conc. | Unit | Mark | Name |
|---|---|---|---|---|---|---|---|
| 1 | 19,117 | 2455474 | 77276 | 63,074 | | M | |
| 2 | 21,656 | 1437536 | 40546 | 36,926 | | V M | |
| Total | | 3893010 | 117822 | | | | |

**Supplementary Figure 147** CSP-HPLC trace of the **(*S*)-13** obtained with **TMSOTf**

<Chromatogram>

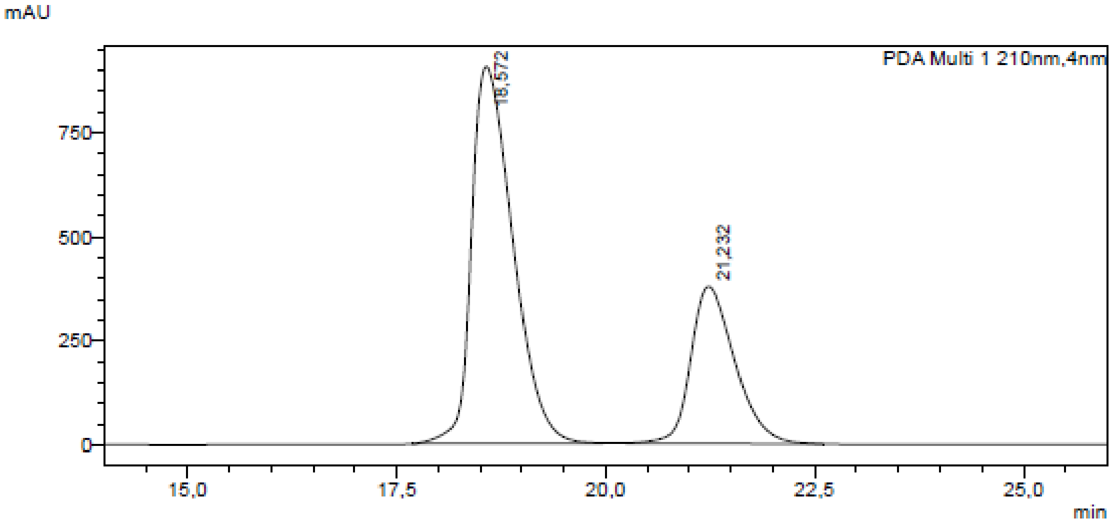


<Peak Table>

PDA Ch1 210nm

| Peak# | Ret. Time | Area | Height | Conc. | Unit | Mark | Name |
|---|---|---|---|---|---|---|---|
| 1 | 18,572 | 31408072 | 906297 | 69,298 | | M | |
| 2 | 21,232 | 13915291 | 377535 | 30,702 | | M | |
| Total | | 45323363 | 1283832 | | | | |

**Supplementary Figure 148** CSP-HPLC trace of **(S)-13** obtained with **$BF_3{\cdot}OEt_2$**

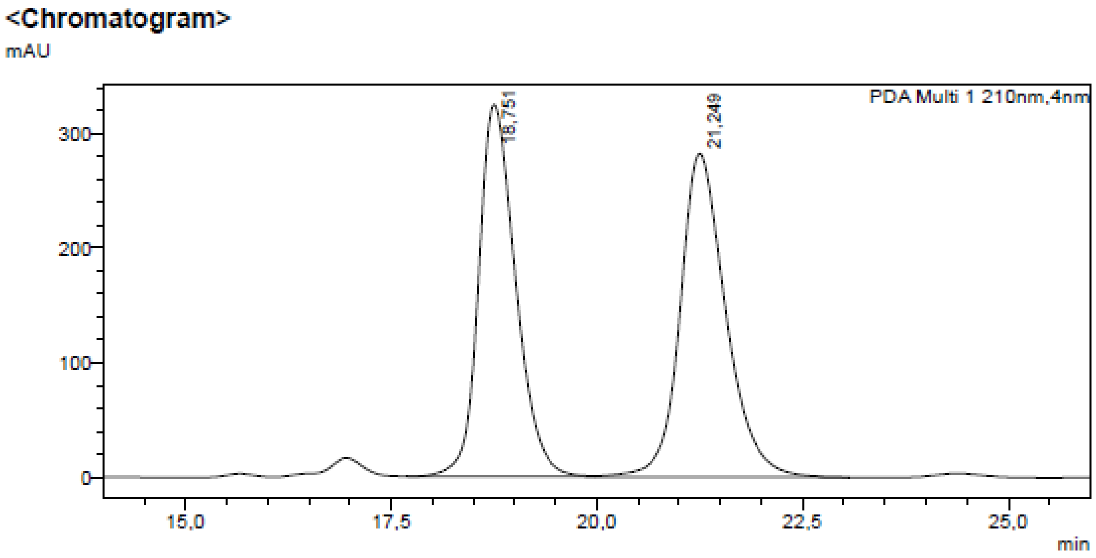


<Peak Table>

PDA Ch1 210nm

| Peak# | Ret. Time | Area | Height | Conc. | Unit | Mark | Name |
|---|---|---|---|---|---|---|---|
| 1 | 18,751 | 10126768 | 324545 | 48,195 | | M | |
| 2 | 21,249 | 10885462 | 282241 | 51,805 | | V M | |
| Total | | 21012230 | 606785 | | | | |

**Supplementary Figure 149** CSP-HPLC trace of **(*rac*)-13**

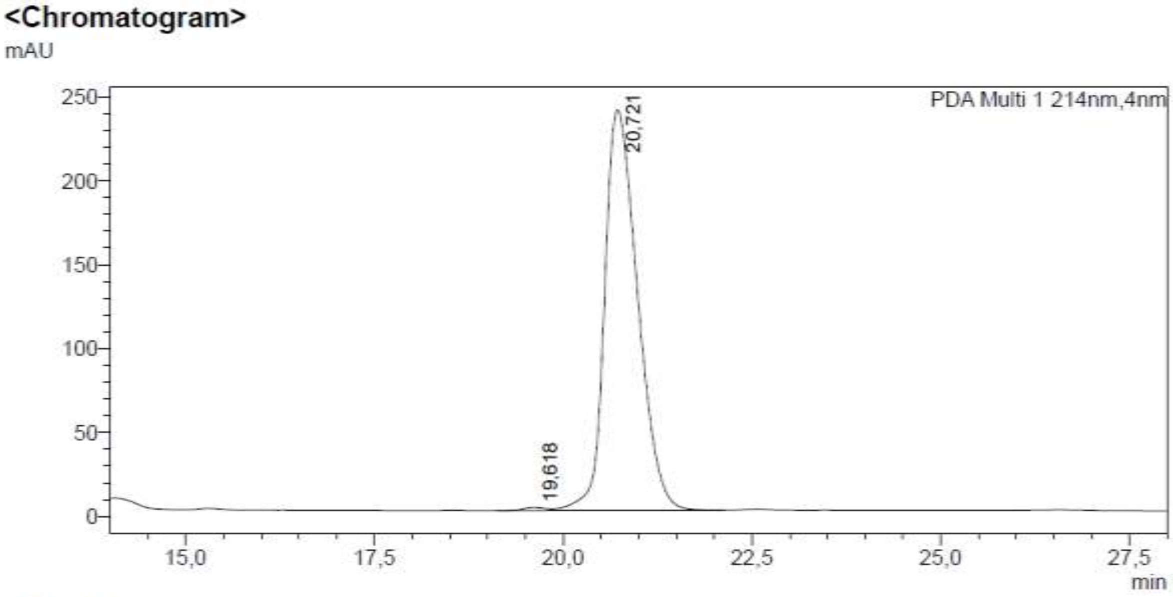


<Peak Table>

PDA Ch1 214nm

| Peak# | Ret. Time | Height | Area | Area% | Conc. | Unit | Mark |
|---|---|---|---|---|---|---|---|
| 1 | 19,618 | 1851 | 39904 | 0,546 | 0,546 | | M |
| 2 | 20,721 | 238847 | 7264048 | 99,454 | 99,454 | | V M |
| Total | | 240698 | 7303952 | 100,000 | | | |

**Supplementary Figure 150** CSP-HPLC trace of ***(R)*-10**

<Chromatogram>

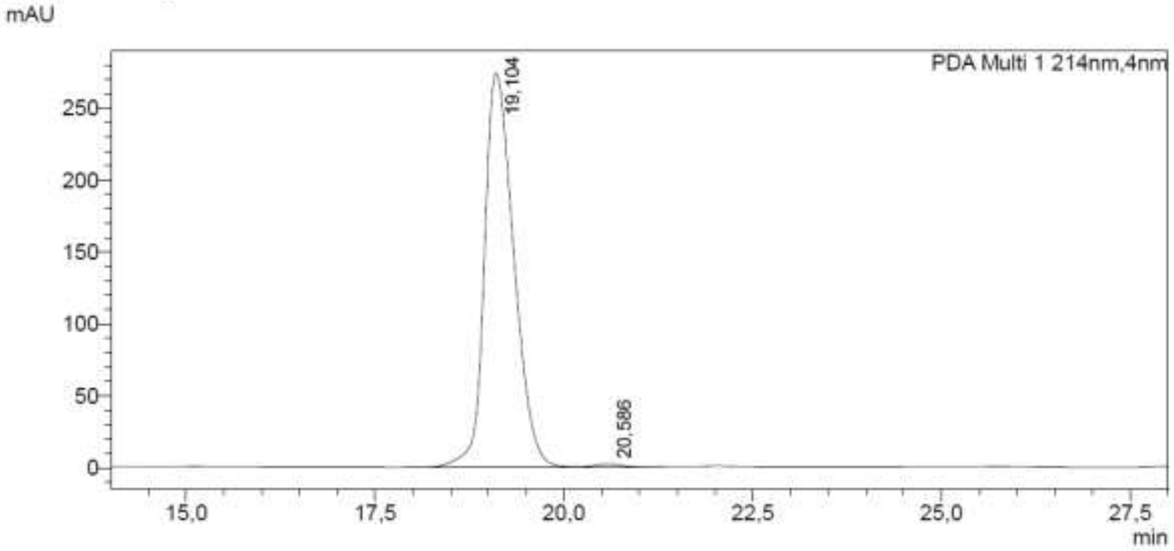


<Peak Table>

PDA Ch1 214nm

| Peak# | Ret. Time | Height | Area | Area% | Conc. | Unit | Mark |
|---|---|---|---|---|---|---|---|
| 1 | 19,104 | 274014 | 7427418 | 99,186 | 99,186 | | M |
| 2 | 20,586 | 2301 | 60939 | 0,814 | 0,814 | | V M |
| Total | | 276315 | 7488357 | 100,000 | | | |

**Supplementary Figure 151** CSP-HPLC trace of ***(S)*-10**

<Chromatogram>

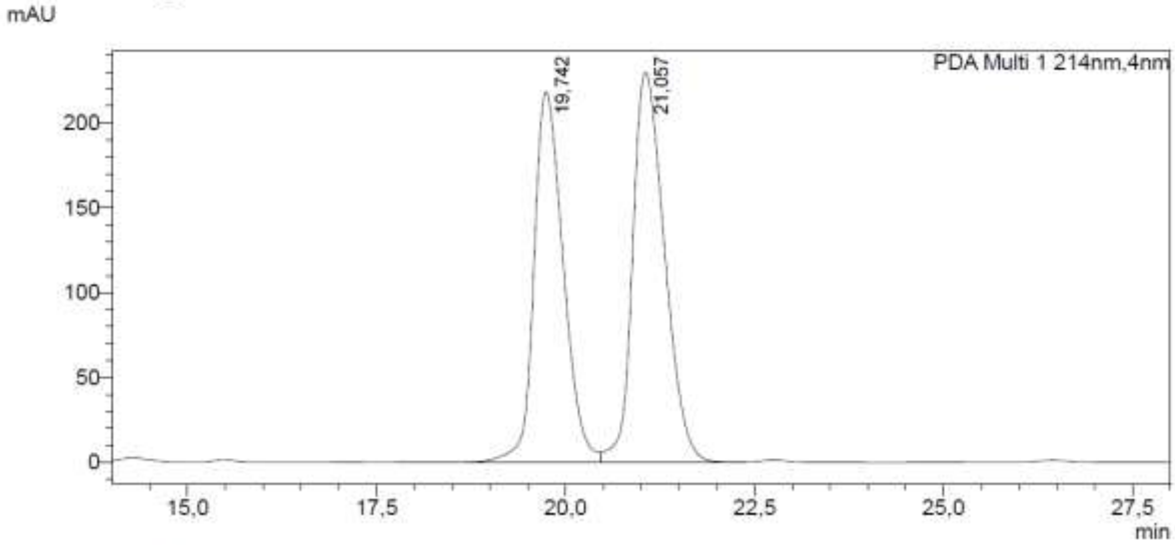


<Peak Table>

PDA Ch1 214nm

| Peak# | Ret. Time | Height | Area | Area% | Conc. | Unit | Mark |
|---|---|---|---|---|---|---|---|
| 1 | 19,742 | 218270 | 5894534 | 46,584 | 46,584 | | M |
| 2 | 21,057 | 229682 | 6759018 | 53,416 | 53,416 | | V M |
| Total | | 447951 | 12653552 | 100,000 | | | |

**Supplementary Figure 152** CSP-HPLC trace of a scalemic mixture of **(*R*)-10** and **(*S*)-10**

<Chromatogram>

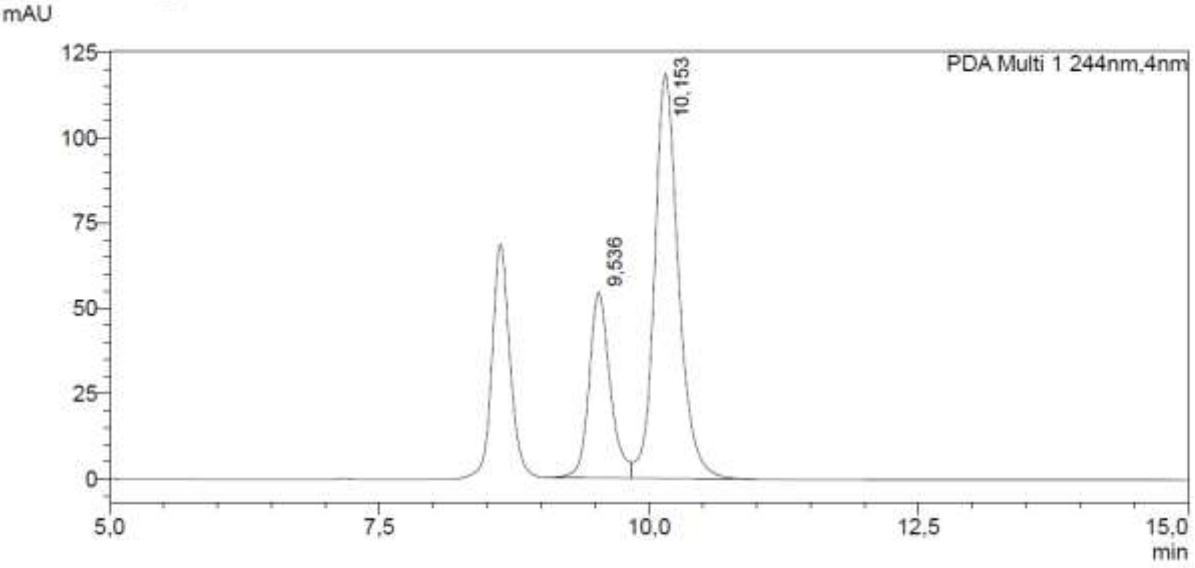


<Peak Table>

PDA Ch1 244nm

| Peak# | Ret. Time | Area | Height | Area% |
|---|---|---|---|---|
| 1 | 9,536 | 740203 | 54387 | 28,685 |
| 2 | 10,153 | 1840279 | 118659 | 71,315 |
| Total | | 2580482 | 173046 | 100,000 |

**Supplementary Figure 153** CSP-HPLC trace of **(*R*)-2a** obtained from **(*Z*)-1a**

<Chromatogram>

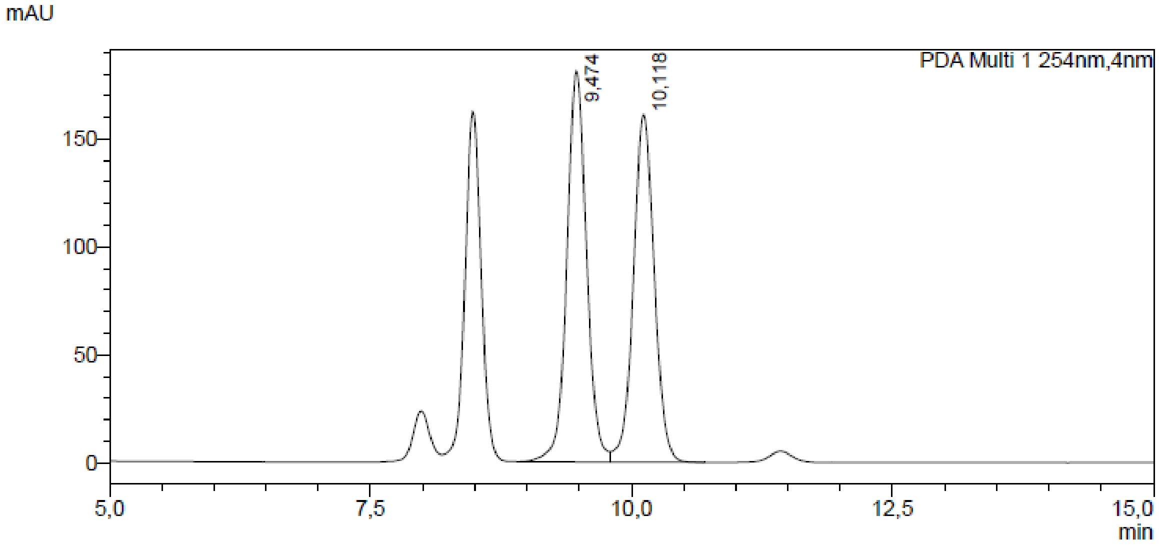


<Peak Table>

PDA Ch1 254nm

| Peak# | Ret. Time | Area | Height | Conc. | Unit | Mark | Name |
|---|---|---|---|---|---|---|---|
| 1 | 9,474 | 2378298 | 180990 | 51,450 | | | |
| 2 | 10,118 | 2244221 | 160770 | 48,550 | | V | |
| Total | | 4622519 | 341760 | | | | |

**Supplementary Figure 154** CSP-HPLC trace of **(*rac*)-2a**

## <Chromatogram>

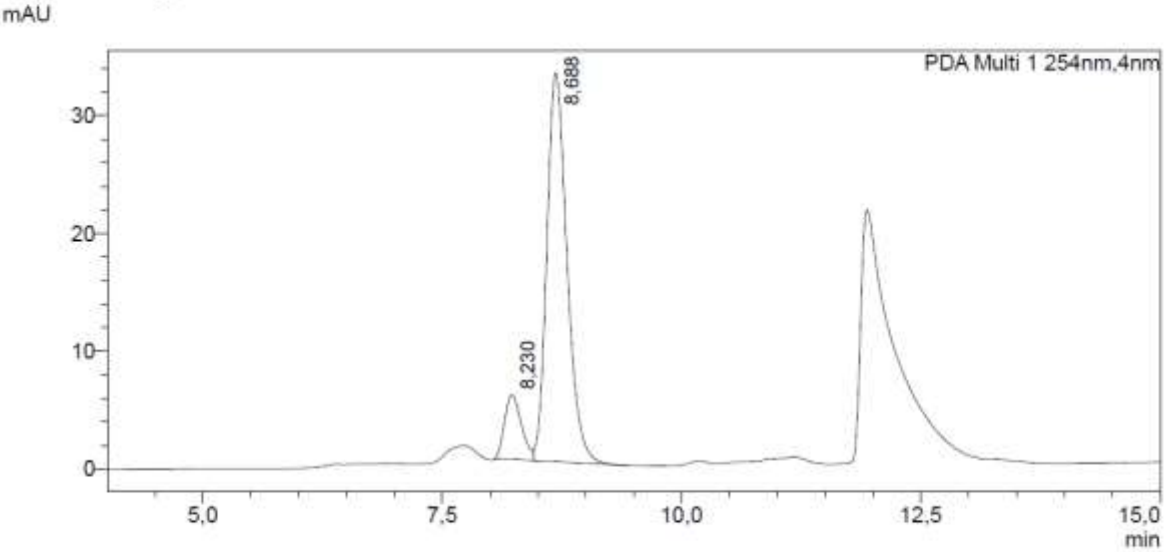


## <Peak Table>

PDA Ch1 254nm

| Peak# | Ret. Time | Height | Conc. | Area% |
|---|---|---|---|---|
| 1 | 8,230 | 5463 | 0,000 | 12,336 |
| 2 | 8,688 | 33011 | 0,000 | 87,664 |
| Total | | 38474 | | 100,000 |

**Supplementary Figure 155** CSP-HPLC trace of **(*R*)-4b** obtained from **(*Z*)-3b**

## <Chromatogram>

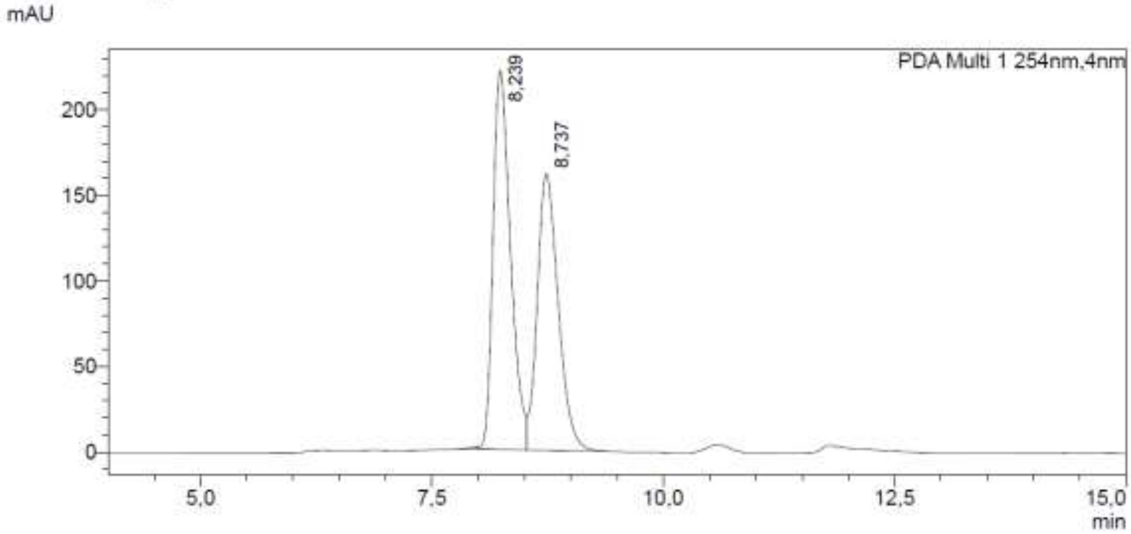


Peak Table

PDA Ch1 254nm

| Peak# | Ret. Time | Area | Height | Area% |
|---|---|---|---|---|
| 1 | 8,239 | 2912684 | 221165 | 53,414 |
| 2 | 8,737 | 2540393 | 161556 | 46,586 |
| Total | | 5453077 | 382722 | 100,000 |

**Supplementary Figure 156** CSP-HPLC trace of **(*rac*)-4b**

# Supplementary methods

## General information

All reactions using oxygen- and/or moisture-sensitive materials were carried out with anhydrous solvents (*vide infr*a) under a nitrogen atmosphere using oven dried glassware and standard Schlenk techniques. Reactions were monitored by $^{1}$H NMR. Purification of the products, when necessary, was performed by flash-column chromatography using Merck 60 Å 230-400 mesh silica gel. NMR data was collected on Bruker Avance NEO 600 ($^{1}$H at 600.0 MHz; $^{13}$C at 150.87MHz), equipped with a Prodigy Cryo-probe, Varian Inova 500 ($^{1}$H at 500.0 MHz; $^{13}$C at 125.72 MHz, $^{19}$F at 470.37 MHz), equipped with an Indirect Detection probe and Varian VXR400 ($^{1}$H at 400.0 MHz; $^{13}$C at 100.58 MHz, $^{19}$F at 376.29, $^{31}$P at 161.94 MHz), equipped with a 5 mm *z*-gradient broadband probe. Chemical shifts are reported in parts per million (ppm) relative to residual solvent peak ($CDCl_3$, $^{1}$H: 7.26 ppm; $^{13}$C: 77.16 ppm). Coupling constants are reported in Hertz. Multiplicity is reported with the usual abbreviations (s: singlet, bs: broad singlet, d: doublet, dd: doublet of doublets, ddd: doublet of doublet of doublets, t: triplet, td: triplet of doublets, q: quartet, dq: doublet of quartet, p: pentet, sex: sextet, hept: heptet, m: multiplet). Exact mass spectra were recorded on a LTQ Orbitrap XL apparatus with ESI ionization. Enantiomeric excesses (ee's)were determined by Chiral HPLC analysis using a Shimadzu LC-10AD*VP* HPLC equipped with a Shimadzu SPD-M10A*VP* diode array detector and by Waters Acquity UPC2 system with PDA detector and QDA mass detector. For the X-ray measurement a Bruker-AXS D8 Venture diffractometer was used. The structures were solved by direct methods using *SHELXT*[1] and refinement of the structure was performed using *SHELXL*.[2] *E-Z* photoisomerization experiments were performed using Spectroline model ENC-280C/FE lamp ($\lambda$max = 365 nm, ± 30nm).

## Chemicals

Unless otherwise indicated, reagents and substrates were purchased from commercial sources and used as received. Solvents not required to be dry were purchased as technical grade and used as received. Dry solvents were freshly collected from a dry solvent purification system prior to use. Inert atmosphere experiments were performed with standard Schlenk techniques with dried ($P_2O_5$) nitrogen gas. Grignard reagents were purchased from Sigma-Aldrich and used as received (EtMgBr (3M in $Et_2O$), ButMgBr, nPropylMgCl, HexMgBr, *i*PentMgBr, *i*ButMgBr, cyclopentylMgBr (2M in $Et_2O$). All other Grignard reagents were prepared from the corresponding alkyl bromides and Mg activated with $I_2$ in $Et_2O$ and concentration was determine by NMR titration method,[3] (but-3-en-1-ylMgBr (2M in $Et_2O$), pent-4-en-1-ylMgBr (1.8M in $Et_2O$), phenethylMgBr (2.6 M in $Et_2O$). Unless otherwise noted substrates were prepared by literature reported methods (*vide infra*). Chiral ligands (**L1**-**L6** and **L10**-**L11**) were purchased from Sigma Aldrich and Solvias. All reported compounds were characterized by $^{1}$H and $^{13}$C NMR and compared with literature data. All new compounds were fully characterized by $^{1}$H and $^{13}$C NMR and HRMS techniques. The absolute configurations of products were determined on the basis of single-crystal X-ray diffraction analysis of compound **2a'** derived from **2a** and the stereochemistry was assign to be *S*.

## Synthesis and characterizations of substrates 1a and 1b

**(*E*)-4-styrylpyridine (1a)**

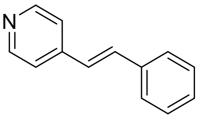

Compound **1a** was synthesized according to the literature procedure.[4] The product was obtained as a white solid after flash-column chromatography ($SiO_2$, pentane:EtOAc, 80:20, v/v). Yield = 63%. The NMR data are in agreement with the one present in literature.[5]

**$^1$H NMR** (400 MHz, $CDCl_3$) δ 8.58 (d, *J* = 5.1 Hz, 2H), 7.55 (d, *J* = 7.0 Hz, 2H), 7.44 – 7.34 (m, 5H), 7.31 (d, *J* = 16.0 Hz, 1H), 7.02 (d, *J* = 16.3 Hz, 1H).

**$^{13}$C NMR** (101 MHz, $CDCl_3$) δ 150.3, 144.8, 136.3, 133.3, 129.0, 128.9, 127.2, 126.1, 121.0.

**HRMS (ESI$^+$):** *m/z* calcd. for $C_{13}H_{12}N$ ([M+H$^+$]) 182.09643, found 182.09655.

**(*E*)-4-(4-(trifluoromethyl)styryl)pyridine (1b)**

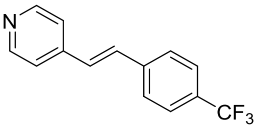

Compound **1b** was synthesized according to the literature procedure.[4] The product was obtained as a white solid after flash-column chromatography ($SiO_2$, pentane:EtOAc, 60:40, v/v). Yield = 11%. The NMR data are in agreement with the one present in literature.[6]

**$^1$H NMR** (400 MHz, $CDCl_3$) δ 8.62 (d, *J* = 5.2 Hz, 2H), 7.64 (s, 4H), 7.39 (d, *J* = 5.0 Hz, 2H), 7.32(d, *J* = 16.3 Hz 1H), 7.10 (d, *J* = 16.4 Hz, 1H).

**$^{13}$C NMR** (101 MHz, $CDCl_3$) δ 150.3, 143.9, 139.6 (q, *J* = 1.3 Hz), 131.5, 130.3 (q, *J* = 32.5 Hz), 128.5, 127.1, 125.8 (q, *J* = 3.9 Hz), 124.1 (q, *J* = 272.0 Hz), 121.0.

**$^{19}$F NMR** (376 MHz, $CDCl_3$) δ -62.61 .

## Synthesis and characterizations of substrates 1c-1d and 3a-3g

**(*E*)-4-(4-(tert-butyldimethylsilyl)but-1-en-1-yl)pyridine (1c)**

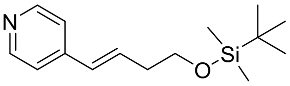

Compound **1c** was synthesized according to the literature procedure.[4] The product was isolated as colourless oil after flash-column chromatography ($SiO_2$, pentane:EtOAc, 80:20 → 60:40, v/v). Yield = 86%

**$^1$H NMR** (400 MHz, $CDCl_3$) δ 8.45 (d, *J* = 6.2 Hz, 2H), 7.14 (d, *J* = 6.2 Hz, 2H), 6.44 (dt, *J* = 16.1, 6.8 Hz, 1H), 6.32 (d, *J* = 16.0 Hz, 1H), 3.70 (t, *J* = 6.5 Hz, 2H), 2.44 (q, *J* = 6.6 Hz, 2H), 0.85 (s, 9H), 0.02 (s, 6H).

**$^{13}$C NMR** (101 MHz, $CDCl_3$) δ 150.0, 144.9, 132.7, 129.5, 120.6, 62.4, 36.6, 25.9, 18.3, -5.2.

**HRMS (ESI$^+$):** *m/z* calcd. for $C_{15}H_{26}NOSi$ ([M+H$^+$]) 264.17782, found 264.17769.

**(*E*)-4-(oct-1-en-1-yl)pyridine (1d)**

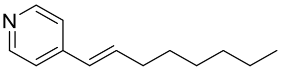

Compound **1d** was synthesized according to the literature procedure.[4] The product was isolated as colourless oil after flash-column chromatography ($SiO_2$, pentane:EtOAc, 80:20 → 60:40, v/v) Yield = 86%

**$^1$H NMR** (400 MHz, $CDCl_3$) δ 8.43 (d, *J* = 6.1 Hz, 2H), 7.12 (d, *J* = 6.1 Hz, 2H), 6.40 (dt, *J* = 15.5, 6.9 Hz, 1H), 6.23 (d, *J* = 15.9 Hz, 1H), 2.16 (q, *J* = 7.3 Hz 2H), 1.41 (p, *J* = 7.2 Hz, 2H), 1.34 – 1.16 (m, 6H), 0.84 (t, *J* = 6.7 Hz, 3H).
**$^{13}$C NMR** (101 MHz, $CDCl_3$) 150.0, 145.1, 136.3, 127.6, 120.5, 33.0, 31.7, 28.9, 22.6, 14.1.
**HRMS (ESI$^+$):** *m/z* calcd. for $C_{13}H_{20}N$ ([M+H$^+$]) 190.15903, measured mass: 190.15890.

**(*E*)-2-(4-(tert-butyldimethylsilyl)but-1-en-1-yl)pyridine (3a)**

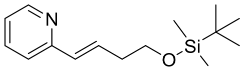


Compound **3a** was synthesized according to the literature procedure.[4] The product was isolated as colourless oil after flash-column chromatography ($SiO_2$, pentane:EtOAc, 97:3 → 93:7, v/v), Yield = 90%
**$^1$H NMR** (400 MHz, $CDCl_3$) δ 8.51 (d, *J* = 4.9 Hz, 1H), 7.58 (t, *J* = 7.7 Hz, 1H), 7.24 (d, *J* = 6.7 Hz, 1H), 7.14 – 7.02 (m, 1H), 6.70 (dt, *J* = 15.8, 7.0 Hz, 1H), 6.53 (d, *J* = 15.8 Hz, 1H), 3.75 (t, *J* = 6.9 Hz, 2H), 2.48 (q, *J* = 6.9 Hz, 2H), 0.89 (s, 9H), 0.05 (s, 6H).
**$^{13}$C NMR** (101 MHz, $CDCl_3$) δ 156.0, 149.5, 136.5, 132.0, 131.9, 121.8, 121.0, 62.8, 36.6, 26.1, 18.5, -5.1.
**HRMS (ESI$^+$):** *m/z* calcd. for $C_{15}H_{26}NOSi$ ([M+H$^+$]) 264.17782, measured mass: 264.17849.

***(E)-2-styryl-5-(trifluoromethyl)pyridine*** **(3b)**

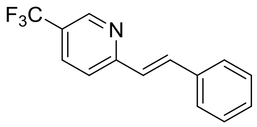


Compound **3b** was synthesized according to the literature procedure.[4] The product was isolated as a white solid after flash-column chromatography ($SiO_2$, pentane:EtOAc, 95:5, v/v), Yield = 84%
**$^1$H NMR** (400 MHz, $CDCl_3$) δ 8.85 (s, 1H), 7.90 (d, *J* = 7.1 Hz, 1H), 7.77 (d, *J* = 16.0 Hz, 1H), 7.61 (d, *J* = 7.5 Hz, 2H), 7.49 (d, *J* = 8.2 Hz, 1H), 7.40 (t, *J* = 7.4 Hz, 2H), 7.37 – 7.30 (m, 1H), 7.21 (d, *J* = 16.1 Hz, 1H).
**$^{13}$C NMR** (101 MHz, $CDCl_3$) δ 158.9, 146.5 (q, *J* = 4.0 Hz), 136.0, 135.6, 133.7 (q, *J* = 3.6 Hz), , 129.0, 128.8, 127.4, 126.5, , 124.3 (q, *J* = 32.9 Hz), 123.7 (q, *J* = 271.8 Hz), 121.4.
**$^{19}$F NMR** (376 MHz, $CDCl_3$) δ -62.2.
**HRMS (ESI$^+$):** *m/z* calcd. for $C_{14}H_{11}F_3N$ ([M+H$^+$]) 250.08381, measured mass: 250.08355

**(*E*)-2-(oct-1-en-1-yl)-5-(trifluoromethyl)pyridine (3c)**

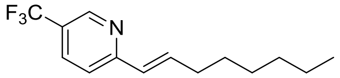


Compound **3c** was synthesized according to the literature procedure.[4] The product was isolated as colourless oil after flash-column chromatography ($SiO_2$, pentane:EtOAc, 99:1, v/v), Yield = 56%
**$^1$H NMR** (400 MHz, $CDCl_3$) δ 8.76 (d, *J* = 2.4 Hz, 1H), 7.81 (dd, *J* = 8.3, 2.3 Hz, 1H), 7.32 (d, *J* = 8.3 Hz, 1H), 6.89 (dt, *J* = 15.6, 7.0 Hz, 1H), 6.52 (d, *J* = 15.6 Hz, 1H), 2.29 (q, *J* = 7.1 Hz, 2H), 1.51 (p, *J* = 7.2 Hz, 2H), 1.42–1.21 (m, 6H), 0.88 (t, *J* = 6.7 Hz, 3H).
**$^{13}$C NMR** (101 MHz, $CDCl_3$) δ 159.5, 146.4 (q, *J* = 4.2 Hz), 139.6, 133.9, 133.7, 133.5 (q, *J* = 3.5 Hz) , 128.9, 128.8, 128.6, 128.5, 124.1 (q, *J* = 32.9 Hz), 123.9 (q, *J* = 271.75 Hz), 120.4, 33.0, 31.8, 29.1, 28.9, 22.7, 14.1.

$^{19}$**F NMR** (376 MHz, $CDCl_3$) δ -62.3.
**HRMS (ESI$^+$):** *m/z* calcd. for $C_{14}H_{18}F_3N$ ([M+H$^+$]) 258.14641, measured mass: 258.14651

**Ethyl (*E*)-6-(oct-1-en-1-yl)nicotinate (3d)**

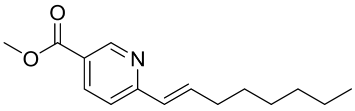

Compound **3d** was synthesized according to the literature procedure.[4] As the product was isolated as the corresponding carboxylic acid it was subjected to the esterification protocol: the acid was dissolved in MeOH and thionylchloride was added dropwise at 0 °C, reaction was warmed to room temperature and kept stirring for additional 2h. The reaction mixture was poured in $H_2O$ and extracted with EtOAc. The organic layer was then washed with $NaHCO_3$ and dried on $MgSO_4$, volatiles were removed on rotary evaporator. The compound **3d** was obtained as colourless oil after flash-column chromatography ($SiO_2$, pentane:EtOAc, 90:10, v/v), Yield = 56%
$^1$**H NMR** (400 MHz, $CDCl_3$) δ 9.07 (d, *J* = 2.2 Hz, 1H), 8.13 (dd, *J* = 8.2, 2.2 Hz, 1H), 7.23 (d, *J* = 8.2 Hz, 1H), 6.86 (dt, *J* = 15.6, 7.0 Hz, 1H), 6.48 (d, *J* = 15.7 Hz, 1H), 3.88 (s, 3H), 2.24 (q, *J* = 7.0 Hz, 2H), 1.46 (p, *J* = 6.9 Hz, 2H), 1.38 – 1.15 (m, 6H), 0.84 (t, *J* = 6.7 Hz, 3H).
$^{13}$**C NMR** (101 MHz, $CDCl_3$) δ 165.9, 159.8, 150.8, 139.5, 137.5, 129.3, 123.5, 120.4, 52.2, 33.1, 31.7, 29.0, 28.8, 22.6, 14.1.
**HRMS (ESI$^+$):** *m/z* calcd. for $C_{15}H_{22}NO_2$ ([M+H$^+$]) 248.16451, found 248.16433

**(*E*)-6-(oct-1-en-1-yl)nicotinonitrile (3e)**

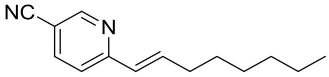

Compound **3e** was synthesized according to the literature procedure.[4] The product was isolated as colourless oil after flash-column chromatography ($SiO_2$, pentane: $CH_2Cl_2$, 60:40 → 50:50, v/v), Yield = 84%
$^1$**H NMR** (400 MHz, $CDCl_3$) δ 8.68 (d, *J* = 2.1 Hz, 1H), 7.77 (dd, *J* = 8.2, 2.2 Hz, 1H), 7.23 (d, *J* = 8.2 Hz, 1H), 6.90 (dt, *J* = 15.7, 7.1 Hz, 1H), 6.43 (d, *J* = 15.6 Hz, 1H), 2.22 (q, *J* = 7.2 Hz, 2H), 1.43 (p, *J* = 6.9 Hz, 2H), 1.36–1.11 (m, 6H), 0.81 (t, *J* = 6.8 Hz, 3H).
$^{13}$**C NMR** (101 MHz, $CDCl_3$) δ 159.2, 152.1, 141.1, 139.4, 128.6, 120.5, 117.1, 106.8, 32.9, 31.6, 28.9, 28.6, 22.5, 14.0.
**HRMS (ESI$^+$):** *m/z* calcd. for $C_{14}H_{19}N_2$ ([M+H$^+$]) 215.15428, found 215.15454

**(*E*)-5-chloro-2-(oct-1-en-1-yl)pyridine (3f)**

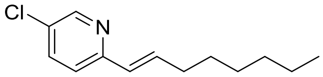

Compound **3f** was synthesized according to the literature procedure.[4] The product was isolated as colourless oil after flash-column chromatography ($SiO_2$, pentane:EtOAc, 99:1 v/v), Yield = 79%
$^1$**H NMR** (400 MHz, $CDCl_3$) δ 8.44 (s, 1H), 7.53 (dt, *J* = 8.4, 2.9 Hz, 1H), 7.15 (dd, *J* = 8.5, 2.8 Hz, 1H), 6.70 (dt, *J* = 14.1, 7.0 Hz, 1H), 6.41 (d, *J* = 13.9 Hz, 1H), 2.23 (q, *J* = 7.2 Hz, 2H), 1.47 (p, 2H), 1.40 – 1.16 (m, 6H), 0.87 (t, *J* = 6.2 Hz, 3H).
$^{13}$**C NMR** (101 MHz, $CDCl_3$) δ 154.3, 148.1, 136.9, 136.0, 129.4, 128.6, 121.4, 32.8, 31.7, 29.0, 28.9, 22.6, 14.1.
**HRMS (ESI$^+$):** *m/z* calcd. for $C_{13}H_{19}ClN$ ([M+H$^+$]) 224.12005, found 224.12066

**(*E*)-5-bromo-2-(oct-1-en-1-yl)pyridine (3g)**

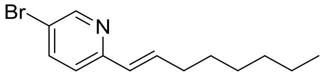

Compound **3g** was synthesized according to the literature procedure.[4] The product was isolated as colourless oil after flash-column chromatography ($SiO_2$, pentane:EtOAc, 99:1, v/v), Yield = 67%

**$^1$H NMR** (400 MHz, $CDCl_3$) δ 8.55 (d, *J* = 2.3 Hz, 1H), 7.68 (dd, *J* = 8.4, 2.3 Hz, 1H), 7.11 (d, *J* = 8.4 Hz, 1H), 6.72 (dt, *J* = 16.0, 6.9 Hz, 1H), 6.40 (d, *J* = 15.7 Hz, 1H), 2.23 (q, *J* = 7.3 Hz, 2H), 1.48 (m, 2H), 1.39–1.16 (m, 6H), 0.87 (t, *J* = 6.9 Hz, 3H).

**$^{13}$C NMR** (101 MHz, $CDCl_3$) δ 154.6, 150.3, 138.98 137.1, 128.7, 121.9, 118.0, 32.8, 31.7, 28.9, 28.8, 22.6, 14.1.**HRMS (ESI$^+$):** *m/z* calcd. for $C_{13}H_{19}BrN$ ([M+H$^+$]) 268.06954, found 268.06967

**(*E*)-2-(2-(pyridin-4-yl)vinyl)pyridine (12)**

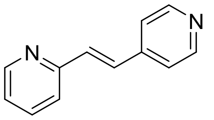

Compound **12** was synthesized according to the literature procedure.[4,7] The product was obtained as a white solid after flash-column chromatography (Neutral alumina, pentane:EtOAc, 80:20→ 0:100, v/v). Yield = 11 %. The NMR data are in agreement with the ones present in literature. [8]

**$^1$H NMR** (400 MHz, $CDCl_3$) δ 8.64 (d, *J* = 4.8 Hz, 1H), 8.60 (d, *J* = 4.9 Hz, 2H), 7.70 (t, 1H), 7.58 (d, *J* = 16.1 Hz, 1H), 7.44 – 7.38 (m, 3H), 7.33 (d, *J* = 16.1 Hz, 1H), 7.22 (dd, 1H).

**$^{13}$C NMR** (101 MHz, $CDCl_3$) δ 154.4, 150.3 (2C), 149.9, 144.0, 136.8, 132.2, 130.0, 123.1, 123.0, 121.3(2C).

## General procedure D for the Cu-catalysed asymmetric Grignard addition to (E)-2-(2-(pyridin-4-yl)vinyl)pyridine (12).

In a heat dried Schlenk tube equipped with septum and magnetic stirring bar, CuBr·$SMe_2$ (0.10 equiv), and ligand (*R,S$_p$*)-**L1** (0.12 equiv) were dissolved in $CH_2Cl_2$ (1 mL/0.1mmol of substrate) and stirred under nitrogen atmosphere for 15 min. The substrate (1.0 equiv) was added at once. After stirring for 5 min. at RT the reaction mixture was cooled to -78 °C and TMSOTf, TMSBr or $BF_3$·$OEt_2$ (1.5 equiv) was added followed by EtMgBr (2.0 equiv). After stirring at -78 °C for 15h, the reaction was quenched with MeOH (1 mL) followed by saturated aqueous solution of $NH_4Cl$ and warmed to RT. The reaction was basified with 0.2 ml of saturated aqueous solution of $NaHCO_3$ and extracted with $CH_2Cl_2$ (3 × 10 mL). Combined organic phases were dried over $MgSO_4$, filtered and solvents were evaporated on rotary evaporator. The oily crude was purified by chromatography on neutral alumina using mixture of pentane and EtOAc (gradient 9:1 to 4:1, v/v) as eluent.

## General procedure E for the synthesis of racemic product from substrate 12.

In a heat dried Schlenk tube equipped with septum and magnetic stirring bar. The substrate (1.0 equiv) was dissolved in $CH_2Cl_2$ (1 mL/0.1mmol of substrate).The reaction mixture was cooled to -78 °C and TMSOTf (3.0 equiv) was added followed by EtMgBr (2.0 equiv). After stirring at -78 °C for 15h, the reaction was quenched as above and purified by flash chromatography on neutral alumina using mixture of pentane and EtOAc (80:20,v/v) as eluent.

## Synthesis and characterizations of products (2a - 2d, 4a – 4g, and 5a - 5j)

**(*S*)-4-(2-phenylbutyl)pyridine (2a)**

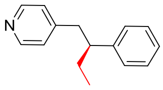

The reaction was performed using general procedure A, with 0.1 mmol **1a**, TMSOTf (0.3 mmol, 3.0 equiv), EtMgBr (3M in $Et_2O$, 0.3 mmol, 3.0 equiv), CuBr·$SMe_2$ (0.01 mmol, 10 mol%), ligand (*R,S$_p$*)-**L1** (0.012 mmol, 12 mol%) in 1 mL $CH_2Cl_2$. Product **2a** was obtained as pale-yellow oil after flash-column chromatography ($SiO_2$, Pentane:EtOAc 80:20, v/v), [94% yield, 93% ee].

Note: Using TMSBr as Lewis acid product **2a** was isolated with 85% yield and > 99.9% ee.

**$^1$H NMR** (400 MHz, $CDCl_3$) δ 8.39 (d, *J* = 5.0 Hz, 2H), 7.29–7.21 (m, 2H), 7.21–7.12 (m, 1H), 7.06 (d, *J* = 6.8 Hz, 2H), 6.91 (d, *J* = 6.1 Hz, 2H), 2.97–2.78 (m, 2H), 2.78 – 2.65 (m, 1H), 1.79–1.58 (m, 2H), 0.79 (t, *J* = 7.3 Hz, 3H).

**$^{13}$C NMR** (101 MHz, $CDCl_3$) δ 149.8, 149.6, 143.9, 128.5, 127.9, 126.5, 124.7, 49.2, 42.8, 28.9, 12.1.

**HRMS (ESI$^+$):** *m/z* calcd. for $C_{15}H_{18}N$ ([M+H$^+$]) 212.14338, found 212.14315

**CSP-HPLC:** (254 nm, Chiralcel OZ-H, *n*-heptane:*i*PrOH = 95:5, 40 °C, 1.0 ml/min.), $t_R$ = 9.61 min (major), $t_R$ = 10.27 min (minor).

**(*S*)-4-(2-(4-(trifluoromethyl)phenyl)butyl)pyridine (2b)**

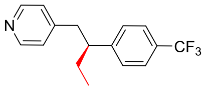


The reaction was performed using general procedure A, with 0.1 mmol **1b**, TMSOTf (0.3 mmol, 3.0 equiv), CuBr·$SMe_2$ (0.01 mmol, 10 mol%), ligand (*R,S$_p$*)-**L1** (0.012 mmol, 12 mol%) in 1 mL $CH_2Cl_2$. EtMgBr (3M in $Et_2O$, 0.3 mmol, 3.0 equiv) diluted to 0.35 mL toluene and added by syringe pump for 2h. Product **2b** was obtained as pale-yellow oil after flash-column chromatography ($SiO_2$, Pentane:EtOAc 80:20, v/v), [87% yield, 89% ee].

**$^1$H NMR** (400 MHz, $CDCl_3$) δ 8.41 (s, 2H), 7.50 (d, *J* = 8.0 Hz, 2H), 7.17 (d, *J* = 8.1 Hz, 2H), 6.90 (d, *J* = 5.0 Hz, 2H), 3.05-2.89 (m, 1H), 2.87–2.77 (m, 2H), 1.83–1.60 (m, 2H), 0.79 (t, *J* = 7.4 Hz, 3H).

**$^{13}$C NMR** (101 MHz, $CDCl_3$) δ 149.74 , 149.68, 149.1, 148.1, 128.2, 125.7 (q, *J* = 5.7 Hz), 125.4 (q, *J* = 3.8 Hz), 124.5, 49.1, 42.5, 28.8, 12.1.

**$^{19}$F NMR** (376 MHz, $CDCl_3$) δ -62.4.

**HRMS (ESI$^+$):** *m/z* calcd. for $C_{16}H_{17}F_3N$ ([M+H$^+$]) 280.13076, found 280.13146

**CSP-HPLC:** (206 nm, Chiralcel OZ-H, *n*-heptane:*i*PrOH = 95:5, 40 °C, 0.5 ml/min.), $t_R$ = 21.72 min (major), $t_R$ = 22.58 min (minor).

**(*S*)-4-(4-(tert-butyldimethylsilyl)-2-ethylbutyl)pyridine (2c)**

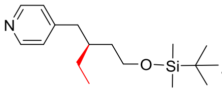


The reaction was performed using general procedure A, with 0.1 mmol **1c**, $BF_3$·$OEt_2$ (0.2 mmol, 2.0 equiv), EtMgBr (3M in $Et_2O$, 0.3 mmol, 3.0 equiv), CuBr·$SMe_2$ (0.01 mmol, 10 mol%), ligand (*R,S$_p$)*-**L1** (0.012 mmol, 12 mol%) in 1 mL $CH_2Cl_2$. Product **2c** was obtained as pale-yellow oil after flash-column chromatography ($SiO_2$, Pentane:EtOAc 90:10 → 85:15, v/v), [82% yield, 97% ee, ee measured after deprotection, see compound **2c'**]

**$^1$H NMR** (400 MHz, $CDCl_3$) δ 8.49 (s, 2H), 7.09 (d, *J* = 4.7 Hz, 2H), 3.79–3.49 (m, 2H), 2.55 (d, *J* = 7.2 Hz, 2H), 1.88–1.67 (m, 1H), 1.62–1.36 (m, 2H), 1.37–1.20 (m, 2H), 0.90 (t, *J* =6.9 Hz, 3H), 0.88 (s, 9H), 0.02 (d, *J* = 3.0 Hz, 6H).

**$^{13}$C NMR** (101 MHz, $CDCl_3$) δ 150.8, 149.6, 124.9, 61.2, 39.7, 37.3, 36.0, 26.1, 25.8, 18.4, 10.8, -5.2.

**HRMS (ESI$^+$):** *m/z* calcd. for $C_{17}H_{32}NOSi$ ([M+H$^+$]) 294.22477, found 294.22483.

**(*S*)-3-(pyridin-4-ylmethyl)pentan-1-ol (2c')**

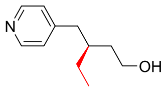

In flame dried Schelnk, 0.17 mmol of **2c** were dissolved in THF (0.3 ml). The reaction mixture was cooled to 0 °C and a TBAF solution (1M in THF) was added dropwise. The reaction mixture was stirred for 4h while warming to RT. The crude was quenched with $H_2O$ and extracted with EtOAc. The organic layer was dried on $MgSO_4$. Product **2c'** was obtained as a pale-yellow oil after flash-column chromatography ($SiO_2$ EtOAc → MeOH) [36% yield, 97% ee].

**$^1$H NMR** (400 MHz, $CDCl_3$) δ 8.44 (d, *J* = 4.9 Hz, 2H), 7.09 (d, *J* = 5.0 Hz, 2H), 3.74–3.57 (m, 2H), 2.69–2.46 (m, 2H), 1.96 (s, 1H), 1.86–1.71 (m, 1H), 1.65–1.43 (m, 2H), 1.41–1.18 (m, 2H), 0.90 (t, *J* = 7.4 Hz, 3H).

**$^{13}$C NMR** (101 MHz, $CDCl_3$) δ 150.6, 149.6, 124.8, 60.8, 39.7, 37.3, 36.0, 25.8, 10.8.

**HRMS (ESI$^+$):** *m/z* calcd. for $C_{11}H_{18}NO$ ([M+H$^+$]) 180.13829, found 180.13824.

**CSP-HPLC:** (254 nm, Chiralcel AY-H, *n*-heptane:*i*PrOH = 95:5, 40 °C, 0.5 ml/min.), $t_R$ = 22.89 min (minor), $t_R$ = 23.99 min (major).

**(*R*)-4-(2-ethyloctyl)pyridine (2d)**

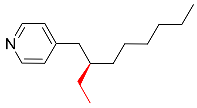

The reaction was performed using general procedure A, with 0.1 mmol **1d**, TMSOTf (0.3 mmol, 3.0 equiv), EtMgBr (3M in $Et_2O$, 0.3 mmol, 3.0 equiv), CuBr·$SMe_2$ (0.01 mmol, 10 mol%), ligand (*R,S$_p$*)-**L1** (0.012 mmol, 12 mol%) in 1 mL $CH_2Cl_2$. Product **2d** was obtained as pale-yellow oil after flash-column chromatography ($SiO_2$, Pentane:EtOAc 80:20, v/v), [86% yield, 93% ee].

**$^1$H NMR** (400 MHz, $CDCl_3$) δ 8.48 (s, 2H), 7.07 (d, *J* = 4.6 Hz, 2H), 2.51 (d, *J* = 7.1 Hz, 2H), 1.66–1.52 (m, 1H), 1.42–1.11 (m, 12H), 0.86 (t, *J* = 7.2 Hz, 6H).

**$^{13}$C NMR** (101 MHz, $CDCl_3$) δ 151.1, 149.6, 124.8, 40.6, 39.7, 32.8, 32.0, 29.7, 26.7, 25.6, 22.8, 14.2, 10.9.

**HRMS (ESI$^+$):** *m/z* calcd. for $C_{15}H_{26}N$ ([M+H$^+$]) 220.20598, found 220.20575

**CSP-HPLC:** (254 nm, Chiralcel OD-H, *n*-heptane:*i*PrOH = 99.2:0.8, 40 °C, 0.5 ml/min.), $t_R$ = 25.37 min (major), $t_R$ = 26.11 min (minor).

**(*S*)-2-(4-(tert-butyldimethylsilyl)-2-ethylbutyl)pyridine (4a)**

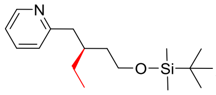

The reaction was performed using general procedure A, with 0.1 mmol **3a**, TMSOTf (0.3 mmol, 3.0 equiv), CuBr·$SMe_2$ (0.01 mmol, 10 mol%), ligand (*R,S$_p$*)-**L1** (0.012 mmol, 12 mol%) in 1 mL $CH_2Cl_2$. EtMgBr (3M in $Et_2O$, 0.4 mmol, 4.0 equiv) diluted to 0.45 mL in toluene and added over a period of 2h. Product **4a** was obtained as pale-yellow oil after flash-column chromatography ($SiO_2$, Pentane:EtOAc 98:2, v/v), [74% yield, 89% ee].

**$^1$H NMR** (400 MHz, $CDCl_3$) δ 8.52 (d, *J* = 4.9 Hz, 1H), 7.56 (td, *J* = 7.7, 1.9 Hz, 1H), 7.16–7.04 (m, 2H), 3.68–3.53 (m, 2H), 2.73 (qd, *J* = 13.5, 7.3 Hz, 2H), 1.98–1.88 (m, 1H), 1.61–1.42 (m, 2H), 1.40–127 (m, 2H), 0.89 (t, *J* = 7.4 Hz, 3H), 0.86 (s, 9H), 0.00 (d, *J* = 3.4 Hz, 6H).

**$^{13}$C NMR** (101 MHz, $CDCl_3$) δ 161.7, 149.3, 136.1, 123.7, 121.0, 61.6, 43.0, 37.1, 36.2, 26.2, 26.1, 18.4, 10.9, -5.1.

**HRMS (ESI$^+$):** *m/z* calcd. for $C_{17}H_{32}NOSi$ ([M+H$^+$]) 294.22477, found 294.22502

**CSP-HPLC:** (254 nm, Chiralcel OD-H, *n*-heptane:*i*PrOH = 99.2:0.8, 40 °C, 0.5 ml/min.), $t_R$ = 9.96 min (minor), $t_R$ = 10.55 min (major).

**(S)-2-(2-phenylbutyl)-5-(trifluoromethyl)pyridine (4b)**

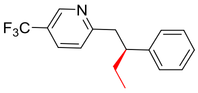

The reaction was performed using general procedure B, with 0.1 mmol **3c**, $BF_3$·$OEt_2$ (0.15 mmol, 1.5 equiv), EtMgBr (3M in $Et_2O$, 0.15 mmol, 1.5 equiv), CuBr·$SMe_2$ (0.005 mmol, 5 mol%), ligand (*R,S$_p$*)-**L1** (0.006 mmol, 6 mol%) in 1 mL $Et_2O$. Product **4c** was obtained as pale-yellow oil after flash-column chromatography ($SiO_2$, Pentane:EtOAc 99:1, v/v), [52% yield, 86% ee]

**$^1$H NMR** (400 MHz, $CDCl_3$) δ 8.78 (d, *J* = 2.3 Hz, 1H), 7.79 (dd, *J* = 8.2, 2.3 Hz, 1H), 7.23 (d, *J* = 8.1 Hz, 1H), 2.77 (d, *J* = 7.2 Hz, 2H), 1.84 (hept, *J* = 6.5 Hz, 1H), 1.40–1.10 (m, 12H), 0.86 (q, *J* = 7.3 Hz, 6H).

**$^{13}$C NMR** (101 MHz, $CDCl_3$) δ 164.8, 146.0 (q, *J* = 4.0 Hz), 143.91, 132.9 (q, *J* = 3.4 Hz), 128.31, 127.68, 126.26, , 124.00 (q, *J* = 32.7 Hz), 123.25,122.3 (q, *J* = 271.8 Hz) 48.04, 45.57, 28.95, 12.02.

**$^{19}$F NMR** (376 MHz, $CDCl_3$) δ -62.3.

**HRMS (ESI$^+$):** *m/z* calcd. for $C_{16}H_{17}F_3N$ ([M+H$^+$]) 280.13076, found 280.13144

**CSP-HPLC:** (254 nm, Chiralcel OJ-H, *n*-heptane:*i*PrOH = 99.5:0.5, 40 °C, 0.5 ml/min.), $t_R$ = 8.18 min (major), $t_R$ = 8.64 (minor).

**(*R*)-2-(2-ethyloctyl)-5-(trifluoromethyl)pyridine (4c)**

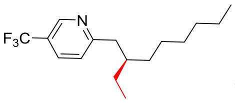

The reaction was performed using general procedure B, with 0.38 mmol **3c**, $BF_3$·$OEt_2$ (0.57 mmol, 1.5 equiv), EtMgBr (3M in $Et_2O$, 0.57 mmol, 1.5 equiv), CuBr·$SMe_2$ (0.019 mmol, 5 mol%), ligand (*R,S$_p$*)-**L1** (0.023 mmol, 6 mol%) in 4 mL $Et_2O$. Product **4c** was obtained as pale-yellow oil after flash-column chromatography ($SiO_2$, Pentane:EtOAc 99:1, v/v), [60% yield, 99% ee, ee measured after further functionalization, see compound **10)**]

**$^1$H NMR** (400 MHz, $CDCl_3$) δ 8.78 (d, *J* = 2.3 Hz, 1H), 7.79 (dd, *J* = 8.2, 2.3 Hz, 1H), 7.23 (d, *J* = 8.1 Hz, 1H), 2.77 (d, *J* = 7.2 Hz, 2H), 1.84 (hept, *J* = 6.5 Hz, 1H), 1.40–1.10 (m, 12H), 0.86 (q, *J* = 7.3 Hz, 6H).

**$^{13}$C NMR** (101 MHz, $CDCl_3$) δ 166.4, 146.2 (q, *J* = 4.1 Hz), 133.1 (q, *J* = 3.4 Hz), 124.0 (q, *J* = 32.9 Hz), 124.0 (q, *J* = 271.9 Hz), 123.3, 42.9, 40.1, 32.9, 32.0, 29.7, 26.6, 25.8, 22.8, 14.2, 10.8 .

**$^{19}$F NMR** (376 MHz, $CDCl_3$) δ -62.3.

**HRMS (ESI$^+$):** *m/z* calcd. for $C_{16}H_{25}F_3N$ ([M+H$^+$]) 288.19336, found 288.19397

**Methyl (*R*)-6-(2-ethyloctyl)nicotinate (4d)**

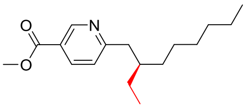

The reaction was performed using general procedure B, with 0.1 mmol **3d**, $BF_3$·$OEt_2$ (0.11 mmol, 1.1 equiv), EtMgBr (3M in $Et_2O$, 0.12 mmol, 1.2 equiv), CuBr·$SMe_2$ (0.01 mmol, 10 mol%), ligand (*R,S$_p$*)-**L1** (0.012 mmol, 12 mol%) in 1 mL $Et_2O$. Product **4d** was obtained as pale-yellow oil after flash-column chromatography ($SiO_2$, Pentane:EtOAc 90:10, v/v), [93% yield, 89% ee]

**$^1$H NMR** (400 MHz, $CDCl_3$) δ 9.12 (d, *J* = 2.2 Hz, 1H), 8.16 (dd, *J* = 8.1, 2.3 Hz, 1H), 7.19 (d, *J* = 8.1 Hz, 1H), 3.92 (s, 3H), 2.76 (d, *J* = 7.6 Hz, 2H), 1.91–1.77 (m, 1H), 1.38–1.13 (m, 12H), 0.85 (td, *J* = 7.2, 4.8 Hz, 6H).

**$^{13}$C NMR** (101 MHz, $CDCl_3$) δ 167.0, 166.2, 150.6, 137.1, 123.5, 123.3, 52.3, 43.1, 40.1, 33.0, 32.0, 29.8, 26.6, 25.9, 22.8, 14.2, 10.8.

**HRMS (ESI$^+$):** *m/z* calcd. for $C_{17}H_{28}NO_2$ ([M+H$^+$]) 278.21146, found 278.21169

**CSP-HPLC:** (238 nm, Chiralcel OZ-H, *n*-heptane:*i*PrOH = 99.2:0.8, 40 °C, 0.5 ml/min.), $t_R$ = 53.02 min (minor), $t_R$ = 59.09 min (major).

**(*R*)-6-(2-ethyloctyl)nicotinonitrile (4e)**

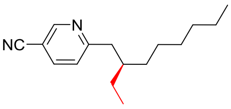


The reaction was performed using general procedure B, with 0.3 mmol **3e**, $BF_3$·$OEt_2$ (0.45 mmol, 1.5 equiv), EtMgBr (3M in $Et_2O$, 0.6 mmol, 2.0 equiv), CuBr·$SMe_2$ (0.03 mmol, 10 mol%), ligand (*R,S$_p$*)-**L1** (0.036 mmol, 12 mol%) in 3mL $CH_2Cl_2$. Product **4e** was obtained as pale-yellow oil after flash-column chromatography ($SiO_2$, Pentane:EtOAc 98:2, v/v), [64% yield, 87% ee]

**$^1$H NMR** (400 MHz, $CDCl_3$) δ 8.81 (s, 1H), 7.84 (dd, *J* = 8.1, 2.2 Hz, 1H), 7.24 (d, *J* = 8.0 Hz, 1H), 2.77 (d, *J* = 7.2 Hz, 2H), 1.84 (p, *J* = 6.4 Hz, 1H), 1.36–1.16 (m, 12H), 0.87 (m, 6H).

**$^{13}$C NMR** (101 MHz, $CDCl_3$) δ 167.0, 152.2, 139.1, 123.7, 117.2, 107.1, 43.2, 40.1, 33.0, 32.0, 29.9, 26.6, 25.9, 22.8, 14.2, 10.8.

**HRMS (ESI$^+$):** *m/z* calcd. for $C_{16}H_{25}N_2$ ([M+H$^+$]) 245.20123, found 245.20186

**CSP-HPLC:** (224 nm, Chiralcel AY-H, *n*-heptane:*i*PrOH = 99.7:0.3, 40 °C, 0.25 ml/min.), $t_R$ = 24.34 min (major), $t_R$ = 30.96min (minor).

**(*R*)-5-chloro-2-(2-ethyloctyl)pyridine (4f)**

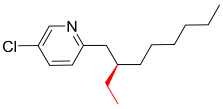


The reaction was performed using general procedure B, with 0.1 mmol **3f**, $BF_3$·$OEt_2$ (0.15 mmol, 1.5 equiv), EtMgBr (3M in $Et_2O$, 0.2 mmol, 2.0 equiv), CuBr·$SMe_2$ (0.01 mmol, 10 mol%), ligand (*R,S$_p$*)-**L1** (0.012 mmol, 12 mol%) in 1 mL $CH_2Cl_2$. Product **4f** was obtained as pale-yellow oil after flash-column chromatography ($SiO_2$, Pentane:EtOAc 98:2, v/v), [59% yield, 95% ee]

**$^1$H NMR** (400 MHz, $CDCl_3$) δ 8.48 (d, *J* = 2.5 Hz, 1H), 7.54 (dd, *J* = 8.3, 2.5 Hz, 1H), 7.06 (d, *J* = 8.3 Hz, 1H), 2.67 (d, *J* = 7.3 Hz, 2H), 1.83–1.70 (m, 1H), 1.41–1.16 (m, 12H), 0.86 (t, *J* = 7.3 Hz, 6H).

**$^{13}$C NMR** (101 MHz, $CDCl_3$) δ 160.3, 148.1, 135.8, 129.2, 124.4, 42.1, 40.1, 32.9, 32.0, 29.8, 26.6, 25.7, 22.8, 14.3, 10.8.

**HRMS (ESI$^+$):** *m/z* calcd. for $C_{15}H_{22}ClN$ ([M+H$^+$]) 254.16700, found 254.16497

**CSP-HPLC:** (217 nm, Chiralcel AD-H, *n*-heptane:*i*PrOH = 99.9:0.1, 40 °C, 0.25 ml/min.), $t_R$ = 20.68 min (major), $t_R$ = 21.56 min (minor).

**(*R*)-5-bromo-2-(2-ethyloctyl)pyridine (4g)**

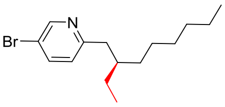


The reaction was performed using general procedure B, with 0.1 mmol **3g**, $BF_3$·$OEt_2$ (0.15 mmol, 1.5 equiv), EtMgBr (3M in $Et_2O$, 0.2 mmol, 2.0 equiv), CuBr·$SMe_2$ (0.01 mmol, 10 mol%), ligand (*R,S$_p$*)-**L1** (0.012 mmol, 12 mol%) in 1 mL $CH_2Cl_2$. Product **4g** was obtained as pale-yellow oil after flash-column chromatography ($SiO_2$, Pentane:EtOAc 98:2, v/v), [75% yield, 99% ee].

**$^1$H NMR** (400 MHz, $CDCl_3$) δ 8.58 (dd, *J* = 2.5, 0.7 Hz, 1H), 7.68 (dd, *J* = 8.2, 2.5 Hz, 1H), 7.01 (dd, *J* = 8.2, 0.7 Hz, 1H), 2.65 (d, *J* = 7.2 Hz, 2H), 1.77 (p, *J* = 7.1 Hz, 1H), 1.37–1.17 (m, 12H), 0.86 (t, *J* = 7.2 Hz, 6H).

**$^{13}$C NMR** (101 MHz, $CDCl_3$) δ 160.7, 150.3, 138.6, 125.0, 117.8, 42.2, 40.1, 32.9, 32.0, 29.8, 26.6, 25.8, 22.8, 14.2, 10.8.

**HRMS (ESI$^+$):** *m/z* calcd. for $C_{15}H_{25}BrN$ ([M+H$^+$]) 298.11649, found 298.11762

**Chiral SFC:** (216 nm, Phenomenex Lux Amylose-1 (3.0 x 150 mm; 3µm), Mobile phase A: $CO_2$ Mobile phase B: Methanol (linear gradient from 98:02 to 90:10 in 3 min. then flush with 60:40, 40 °C, Pump Flow: 1.0ml/min), $t_R$ = 1.99 min (major), $t_R$ = 2.11 min (minor).

**(*S*)-4-(2-phenylpentyl)pyridine (5a)**

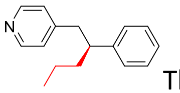 The reaction was performed using general procedure A, with 0.1 mmol **1a**, TMSOTf (0.3 mmol, 3.0 equiv), *n*-PrMgCl (2M in $Et_2O$, 0.3 mmol, 3.0 equiv), CuBr·$SMe_2$ (0.01 mmol, 10 mol%), ligand (*R,S$_p$*)-**L1** (0.012 mmol, 12 mol%) in 1 mL $CH_2Cl_2$. Product **5a** was obtained as pale-yellow oil after flash-column chromatography ($SiO_2$, Pentane:EtOAc 80:20, v/v), [75% yield, 93% ee]

**$^1$H NMR** (400 MHz, $CDCl_3$) δ 8.39 (s, 2H), 7.28–7.20 (m, 2H), 7.20–7.13 (m, 1H), 7.10–7.02 (m, 2H), 6.91 (d, *J* = 5.3 Hz, 2H), 2.93–2.77 (m, 3H), 1.69–1.59 (m, 2H), 1.28–1.10 (m, 2H), 0.83 (t, *J* = 7.3 Hz, 3H).

**$^{13}$C NMR** (101 MHz, $CDCl_3$) δ 149.8, 149.5, 144.2, 128.5, 127.8, 126.4, 124.7, 47.1, 43.2, 38.3, 20.7, 14.1.

**HRMS (ESI$^+$):** *m/z* calcd. for $C_{16}H_{20}N$ ([M+H$^+$]) 226.15903, found 226.15897

**CSP-HPLC:** (254 nm, Chiralcel OZ-H, *n*-heptane:*i*PrOH = 98:02, 40 °C, 0.5 ml/min.), $t_R$ = 29.04 min (major), $t_R$ = 31.39 min. (minor)

**(*S*)-4-(2-phenyloctyl)pyridine (5b)**

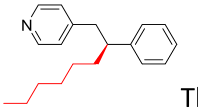 The reaction was performed using general procedure A, with 0.1 mmol **1a**, TMSOTf (0.3 mmol, 3.0 equiv), *n*-HexylMgBr (2M in $Et_2O$, 0.3 mmol, 3.0 equiv), CuBr·$SMe_2$ (0.01 mmol, 10 mol%), ligand (*R,S$_p$*)-**L1** (0.012 mmol, 12 mol%) in 1 mL $CH_2Cl_2$. Product **5b** was obtained as pale-yellow oil after flash-column chromatography ($SiO_2$, Pentane:EtOAc 85:15 → 80:20, v/v), [81% yield, 95% ee]

**$^1$H NMR** (400 MHz, $CDCl_3$) δ 8.39 (s, 2H), 7.23 (d, *J* = 7.5 Hz, 2H), 7.20–7.13 (m, 1H), 7.08–7.01 (m, 2H), 6.90 (d, *J* = 5.3 Hz, 2H), 2.96–2.71 (m, 3H), 1.65 (q, *J* = 7.2, 6.4 Hz, 2H), 1.38–1.04 (m, 8H), 0.83 (t, *J* = 7.0 Hz, 3H).

**$^{13}$C NMR** (101 MHz, $CDCl_3$) δ 149.8, 149.5, 144.3, 128.4, 127.8, 126.4, 124.7, 47.4, 43.2, 36.1, 31.8, 29.4, 27.6, 22.7, 14.2.

**HRMS (ESI$^+$):** *m/z* calcd. for $C_{19}H_{26}N$ ([M+H$^+$]) 268.20598, found 268.20666

**CSP-HPLC:** (254 nm, Chiralcel OZ-H, *n*-heptane:*i*PrOH = 97:03, 40 °C, 0.5 ml/min.), $t_R$ = 18.40 min (major), $t_R$ = 19.47 min. (minor).

**(*S*)-4-(5-methyl-2-phenylhexyl)pyridine (5c)**

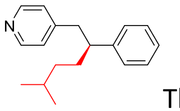 The reaction was performed using general procedure A, with 0.1 mmol **1a**, TMSOTf (0.3 mmol, 3.0 equiv), *i*-PentylMgBr (2M in $Et_2O$, 0.3 mmol, 3.0 equiv), CuBr·$SMe_2$ (0.01 mmol, 10 mol%), ligand (*R,S$_p$*)-**L1** (0.012 mmol, 12 mol%) in 1 mL $CH_2Cl_2$. Product **5c** was obtained as pale-yellow oil after flash-column chromatography ($SiO_2$, Pentane:EtOAc, 90:10, v/v), [65% yield, 97% ee]

**$^1$H NMR** (400 MHz, $CDCl_3$) δ 8.38 (d, *J* = 6.0 Hz, 2H), 7.25–7.20 (m, 2H), 7.19–7.14 (m, 1H), 7.05 (d, *J* = 6.8 Hz, 2H), 6.89 (d, *J* = 6.1 Hz, 2H), 2.96–2.70 (m, 3H), 1.72–1.60 (m, 2H), 1.47 (hept, *J* = 6.7 Hz, 1H), 1.17–0.91 (m, 2H), 0.84–0.76 (m, 6H).

$^{13}$**C NMR** (101 MHz, $CDCl_3$) δ 149.9, 149.5, 144.3, 128.5, 127.8, 126.4, 124.7, 47.7, 43.2, 36.8, 33.9, 28.2, 22.9, 22.5.

**HRMS (ESI$^+$):** *m/z* calcd. for $C_{18}H_{24}N$ ([M+H$^+$]) 254.19033, found 254.19030,

**CSP-HPLC:** (254 nm, Chiralcel OZ-H, *n*-heptane:*i*PrOH = 99.2:0.8, 40 °C, 0.5 ml/min.), $t_R$ = 47.10 min (major), $t_R$ = 51.82 min. (minor).

**(*S*)-4-(2-isobutyloctyl)pyridine (5d)**

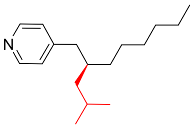

The reaction was performed using general procedure A, with 0.1 mmol **1d**, TMSOTf (0.3 mmol, 3.0 equiv), *i*-butylMgBr (2M in $Et_2O$, 0.3 mmol, 3.0 equiv), CuBr·$SMe_2$ (0.01 mmol, 10 mol%), ligand (*R,S$_p$*)-**L1** (0.012 mmol, 12 mol%) in 1 mL $CH_2Cl_2$. Product **5d** was obtained as pale-yellow oil after flash-column chromatography ($SiO_2$, Pentane:EtOAc 80:20 v/v), [56% yield, 64% ee]

$^1$**H NMR** (400 MHz, $CDCl_3$) δ 8.48 (s, 2H), 7.07 (d, *J* = 5.7 Hz, 2H), 2.50 (d, *J* = 6.9 Hz, 2H), 1.77–1.56 (m, 2H), 1.38-1.08 (m, 11H), 1.08–0.97 (m, 1H), 0.91–0.83 (m, 6H), 0.81 (d, *J* = 6.5 Hz, 3H).

$^{13}$**C NMR** (101 MHz, $CDCl_3$) δ 151.0, 149.6, 124.9, 43.4, 40.4, 36.8, 33.5, 32.0, 29.7, 26.4, 25.4, 23.1, 22.8, 14.2.

**HRMS (ESI$^+$):** *m/z* calcd. for $C_{17}H_{30}N$ ([M+H$^+$]) 248.23728, found 248.23704

**CSP-HPLC:** (254 nm, Chiralcel OZ-H, *n*-heptane:*i*PrOH = 99.5:0.5, 40 °C, 0.5 ml/min.), $t_R$ = 39.87 min (minor), $t_R$ = 42.18 min. (major).

**(*R*)-4-(2-cyclopentyloctyl)pyridine (5e)**

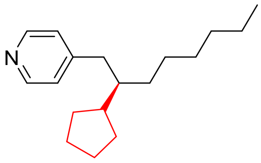

The reaction was performed using general procedure A, with 0.1 mmol **1d**, TMSOTf (0.3 mmol, 3.0 equiv), cyclopentylMgBr (2M in $Et_2O$, 0.4 mmol, 4.0 equiv), CuBr·$SMe_2$ (0.01 mmol, 10 mol%), ligand (*R,S$_p$*)-**L1** (0.012 mmol, 12 mol%) in 1 mL $CH_2Cl_2$. Product **5e** was obtained as pale-yellow oil after flash-column chromatography ($SiO_2$, Pentane:EtOAc 80:20 v/v), [54% yield, 89% ee]

$^1$**H NMR** (400 MHz, $CDCl_3$) δ 8.47 (s, 2H), 7.08 (d, *J* = 4.8 Hz, 2H), 2.77–2.34 (m, 2H), 1.94–1.38 (m, 8H), 1.38–1.10 (m, 12H), 0.86 (t, *J* = 6.9 Hz, 3H).

$^{13}$**C NMR** (101 MHz, $CDCl_3$) δ 151.4, 149.6, 124.8, 44.1, 43.2, 38.3, 31.9, 31.0, 30.7, 30.1, 29.8, 26.3, 25.6, 25.6, 22.8, 14.2.

**HRMS (ESI$^+$):** *m/z* calcd. for $C_{18}H_{29}N$ ([M+H$^+$]) 260.23728, found 260.23713

**CSP-HPLC:** (254 nm, Chiralcel OJ-H, *n*-heptane:*i*PrOH = 99.6:0.4, 40 °C, 0.35 ml/min.), $t_R$ = 12.95 min (major), $t_R$ = 13.86 min. (minor)

**(*S*)-4-(2-ethylhex-5-en-1-yl)pyridine (5f)**

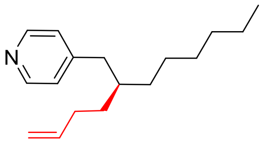

The reaction was performed using general procedure A, with 0.1 mmol **1d**, TMSOTf (0.3 mmol, 3.0 equiv), but-3-en-1-ylMgBr (2M in $Et_2O$, 0.3 mmol, 3.0 equiv), CuBr·$SMe_2$ (0.01 mmol, 10 mol%), ligand (*R,S$_p$*)-**L1** (0.012 mmol, 12 mol%) in 1 mL $CH_2Cl_2$. Product **5f** was obtained as pale-yellow oil after flash-column chromatography ($SiO_2$, Pentane:EtOAc 90:10 v/v), [91% yield, 93% ee]

**$^{1}$H NMR** (400 MHz, $CDCl_3$) δ 8.47 (d, *J* = 5.1 Hz, 2H), 7.06 (d, *J* = 5.4 Hz, 2H), 5.74 (ddt, *J* = 16.9, 10.1, 6.6 Hz, 1H), 5.07–4.82 (m, 2H), 2.52 (d, *J* = 7.1 Hz, 2H), 2.13–1.95 (m, 2H), 1.68 (m, 1H), 1.42–1.14 (m, 12H), 0.87 (t, *J* = 6.8 Hz, 3H).
**$^{13}$C NMR** (101 MHz, $CDCl_3$) δ 150.8, 149.7, 138.8, 124.8, 114.6, 39.9, 38.5, 33.1, 32.5, 31.9, 31.0, 29.7, 26.5, 22.8, 14.2.
**HRMS (ESI$^+$):** *m/z* calcd. for $C_{17}H_{28}N$ ([M+H$^+$]) 246.22163, found 246.22174
**CSP-HPLC:** (215 nm, Chiralcel OD-H, *n*-heptane:*i*PrOH = 99.2:0.8, 40 °C, 0.35 ml/min.), $t_R$ = 25.89 min (major), $t_R$ = 26.54 min. (minor).
**(*S*)-4-(2-phenylhept-6-en-1-yl)pyridine (5g)**

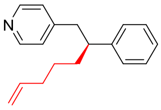

The reaction was performed using general procedure A, with 0.1 mmol **1a**, TMSOTf (0.3 mmol, 3.0 equiv), pent-4-en-1-ylMgBr (1.8M in $Et_2O$, 0.3 mmol, 3.0 equiv), CuBr·$SMe_2$ (0.01 mmol, 10 mol%), ligand (*R,S$_p$*)-**L1** (0.012 mmol, 12 mol%) in 1 mL $CH_2Cl_2$. Product **5g** was obtained as pale-yellow oil after flash-column chromatography ($SiO_2$, Pentane:EtOAc, 90:10, v/v), [66% yield, 90% ee]
**$^{1}$H NMR** (400 MHz, $CDCl_3$) δ 8.41 (s, 2H), 7.31–7.20 (m, 2H), 7.21–7.12 (m, 1H), 7.06 (d, *J* = 6.8 Hz, 2H), 6.91 (s, 2H), 5.70 (ddt, *J* = 16.9, 10.2, 6.7 Hz, 1H), 5.00–4.80 (m, 2H), 2.97–2.72 (m, 3H), 2.10–1.89 (m, 2H), 1.73–1.56 (m, 2H), 1.36–1.17 (m, 2H).
**$^{13}$C NMR** (101 MHz, $CDCl_3$) δ 149.7, 149.6, 144.0, 138.7, 128.5, 127.8, 126.5, 124.7, 114.7, 47.3, 43.2, 35.5, 33.8, 26.9.
**HRMS (ESI$^+$):** *m/z* calcd. for $C_{18}H_{22}N$ ([M+H$^+$]) 252.17468, found 252.17467
**CSP-HPLC:** (254 nm, Chiralcel OZ-H, *n*-heptane:*i*PrOH = 99.0:1.0, 40 °C, 0. 5 ml/min.), $t_R$ = 51.93 min (major), $t_R$ = 56.29 min. (minor)
**(*S*)-4-(2-phenethyloctyl)pyridine (5h)**

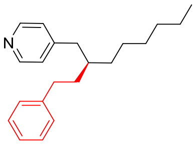

The reaction was performed using general procedure A, with 0.1 mmol **1d**, TMSOTf (0.3 mmol, 3.0 equiv), phenethylMgBr (2.6 M in $Et_2O$, 0.3 mmol, 3.0 equiv), CuBr·$SMe_2$ (0.01 mmol, 10 mol%), ligand (*R,S$_p$*)-**L1** (0.012 mmol, 12 mol%) in 1 mL $CH_2Cl_2$. Product **5h** was obtained as pale-yellow oil after flash-column chromatography ($SiO_2$, Pentane:EtOAc, 90:10 v/v), [89% yield, 97% ee]
**$^{1}$H NMR** (400 MHz, $CDCl_3$) δ 8.47 (d, *J* = 6.0 Hz, 2H), 7.35–7.21 (m, 2H), 7.22–7.14 (m, 1H), 7.12 (d, *J* = 6.7 Hz, 2H), 7.05 (d, *J* = 6.0 Hz, 2H), 2.74–2.48 (m, 3H), 1.75–1.66 (m, 2H), 1.57 (dq, *J* = 9.8, 6.4 Hz, 2H), 1.41–1.16 (m, 10H), 0.88 (t, *J* = 6.8 Hz, 3H).
**$^{13}$C NMR** (101 MHz, $CDCl_3$) δ 150.7 149.6, 142.5, 128.5, 128.4, 125.9, 124.8, 40.0, 38.6, 35.1, 33.2, 33.1, 32.0, 29.7, 26.5, 22.8, 14.2.
**HRMS (ESI$^+$):** *m/z* calcd. for $C_{21}H_{30}N$ ([M+H$^+$]) 296.23728, found 296.23820
**CSP-HPLC:** (254 nm, Chiralcel OD-H, *n*-heptane:*i*PrOH = 99.5:0.5, 40 °C, 0. 5 ml/min.), $t_R$ = 78.87 min (major), $t_R$ = 87.78 min. (minor).
**(*R*)-4-(2-methyloctyl)pyridine (5i)**

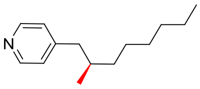

The reaction was performed using general procedure A, (reaction was carried out at 0°C for 5h, MeMgBr was diluted to 0.5 mL total volume and added to reaction mixture by syringe pump for 30 min) with 0.1 mmol **1d**, TMSOTf (0.3 mmol, 3.0 equiv), MeMgBr (3 M in $Et_2O$, 0.3 mmol, 3.0 equiv), CuBr·$SMe_2$ (0.01 mmol, 10 mol%), ligand (*R,S$_p$*)-**L1** (0.012 mmol, 12 mol%) in 1 mL $CH_2Cl_2$.

Product **5i** was obtained as pale-yellow oil after flash-column chromatography ($SiO_2$, Pentane:EtOAc, 90:10 v/v), [50% yield, 93% ee]
**$^1$H NMR** (400 MHz, $CDCl_3$) δ 8.47 (d, *J* = 5.1 Hz, 2H), 7.07 (d, *J* = 6.0 Hz, 2H), 2.67–2.30 (m, 2H), 1.74 (h, *J* = 7.2 Hz, 1H), 1.40–1.09 (m, 10H), 0.87 (t, *J* = 6.8 Hz, 3H), 0.84 (d, *J* = 6.6 Hz, 3H).
**$^{13}$C NMR** (101 MHz, $CDCl_3$) δ 150.9, 149.5, 124.8, 43.1, 36.8, 34.5, 32.0, 29.6, 27.1, 22.8, 19.5, 14.2.
**HRMS (ESI$^+$):** *m/z* calcd. for $C_{14}H_{24}N$ ([M+H$^+$]) 206.19033, found 206.19015
**CSP-HPLC:** (254 nm, Chiralcel OB-H, *n*-heptane:*i*PrOH = 99.8:0.2, 40 °C, 0. 5 ml/min.), $t_R$ = 10.22 min (major), $t_R$ = 10.59 min. (minor).

**(*R*)-4-(2-phenyloctyl)pyridine (5j)**

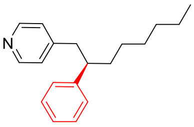

The reaction was performed using general procedure A, with 0.1 mmol **1d**, TMSOTf (0.3 mmol, 3.0 equiv), phenylMgBr (3 M in $Et_2O$, 0.3 mmol, 3.0 equiv), CuBr·$SMe_2$ (0.01 mmol, 10 mol%), ligand (*R*)-**L10** (0.012 mmol, 12 mol%) in 1 mL $CH_2Cl_2$. Product **5j** was obtained as pale-yellow oil after flash-column chromatography ($SiO_2$, Pentane:EtOAc, 97:03 v/v), [84% yield, 0% ee]
**$^1$H NMR** (400 MHz, $CDCl_3$), δ 8.38 (d, *J* = 5.5 Hz, 2H), 7.27–7.19 (m, 2H), 7.17 (d, *J* = 7.2 Hz, 1H), 7.08–7.02 (m, 2H), 6.90 (d, *J* = 6.0 Hz, 2H), 2.95–2.73 (m, 3H), 1.72–1.59 (m, 2H), 1.28–1.10 (m, 8H), 0.83 (t, *J* = 6.9 Hz, 3H).
**$^{13}$C NMR** (101 MHz, $CDCl_3$), δ 149.9, 149.5, 144.2, 128.4, 127.8, 126.4, 124.7, 47.4, 43.2, 36.1, 31.8, 29.4, 27.6 22.7, 14.2.
**HRMS (ESI$^+$):** *m/z* calcd. for $C_{19}H_{26}N$ ([M+H$^+$]) 268.20598, found 268.20686
**CSP-HPLC:** (206 nm, Chiralcel OZ-H, *n*-heptane:*i*PrOH = 97:03, 40 °C, 0.5 ml/min.), $t_R$ = 18.39 min, $t_R$ = 19.39 min.

**(*S*)2-(1-(pyridin-4-yl)butan-2-yl)pyridine (13)**

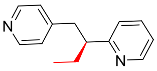

The reaction was performed using general procedure D, with 0.1 mmol **12**, TMSOTf (0.15 mmol, 1.5 equiv), EtMgBr (3M in $Et_2O$, 0.2 mmol, 2.0 equiv), CuBr·$SMe_2$ (0.01 mmol, 10 mol%), ligand (*R,S$_p$*)-**L1** (0.012 mmol, 12 mol%) in 1 mL $CH_2Cl_2$. Product **13** was obtained as pale-yellow oil after column chromatography. [66% yield, 39% ee]
**$^1$H NMR** (400 MHz, $CDCl_3$), δ 8.59–8.54 (m, *J* = 5.5 Hz, 1H), 8.40–8.31 (d, 2H), 7.53–7.44 (m, 1H), 7.12–7.05 (m, 1H), 6.96–6.86 (m, 3H), 3.11–3.00 (m, 2H), 2.99–2.86 (m, 2H), 1.87–1.67 (m, 1H), 0.84–0.74 (t, 3H).
**$^{13}$C NMR** (101 MHz, $CDCl_3$), δ 163.1, 149.9, 149.64, 149.58(2C), 136.2, 124.6(2C), 123.6, 121.6, 50.8, 41.1, 28.2, 12.1.**HRMS (ESI$^+$):** *m/z* calcd. for $C_{14}H_{17}N_2$ ([M+H$^+$]) 213,13863, found 213,13883
**CSP-HPLC:** (210 nm, Chiralcel OZ-H, *n*-heptane:*i*PrOH = 95:05, 40 °C, 1.0 ml/min.), $t_R$ = 19.11 min (major), $t_R$ = 21.65 min (minor).

## General procedures for reactions at higher temperature, large-scale reactions, and reaction using recovered catalyst (Complex)

***Procedure for reaction at room temperature***: In a heat dried Schlenk tube equipped with septum and magnetic stirring bar, was added CuBr·$SMe_2$ (0.01 mmol, 0.1 equiv) and ligand **L1** (0.012 mmol, 0.12 equiv) dissolved in $CH_2Cl_2$ (0.7 mL) and stirred at RT under nitrogen atmosphere for 15 min. The substrate **1a** (0.1 mmol, 1.0 equiv) was added at once using 0.3 mL $CH_2Cl_2$ and stirred for 5 min. To the resulting reaction mixture, at RT, TMSOTf (0.3 mmol, 3.0 equiv) was added. After stirring for 5

min. EtMgBr (0.3 mmol, 3.0 equiv, diluted total volume of 1 mL in $Et_2O$) was added by syringe pump for 30 min. Reactions was quenched after stirring for additional 5 min. and the product **2a** was isolated as in general procedure A. [91% yield, 79% ee]

***Procedure for reaction at 0 °C:*** In a heat dried Schlenk tube equipped with septum and magnetic stirring bar, was added CuBr·$SMe_2$ (0.01 mmol, 0.1 equiv) and ligand **L1** (0.012 mmol, 0.12 equiv) dissolved in $CH_2Cl_2$ (0.7 mL) and stirred at RT under nitrogen atmosphere for 15 min. The substrate **1a** (0.1 mmol, 1.0 equiv) was added at once using 0.3 mL $CH_2Cl_2$ and stirred for 5 min. Reaction mixture cooled to 0 °C and TMSOTf (0.3 mmol, 3.0 equiv) was added. After stirring for 5 min. EtMgBr (0.3 mmol, 3.0 equiv, diluted total volume of 1 mL in $Et_2O$) was added by syringe pump for 30 min. Reactions was quenched after stirring for additional 5 min. and the product **2a** was isolated as in general procedure A. [96% yield, 83% ee]

***Procedure for large-scale reaction and recovery of chiral catalyst:*** Large scale reaction was performed using general procedure A, with only difference of reaction scale (0.1 mmol vs 3.0 mmol). After usual workup, catalyst was isolated in the form of complex by column chromatography ($SiO_2$, Pentane:EtOAc, 80:20 v/v). [recovered yield of complex = 81%; yield of product = 94%, ee = 94%].

***Procedure for reuse of chiral catalyst:*** In a heat dried Schlenk tube equipped with septum and magnetic stirring bar, the recovered complex (0.01 mmol, 0.1 equiv) from above reaction was dissolved in $CH_2Cl_2$ (0.7 mL) and stirred under nitrogen atmosphere for 15 min. The substrate **1a** (0.1 mmol, 1.0 equiv) was added at once using 0.3 mL $CH_2Cl_2$. After stirring for 5 min. at RT the reaction mixture was cooled to -78 °C and TMSOTf (0.3 mmol, 3.0 equiv) was added followed by EtMgBr (0.3 mmol, 3.0 equiv). After stirring at -78 °C for 16h, the reaction was quenched and product **2a** was isolated as in general procedure A. [93% yield, 88% ee]

## Synthesis and characterization of functionalized pyridines (6-10).

### (*S*)-4-(2-phenylbutyl)piperidine (6)

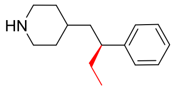

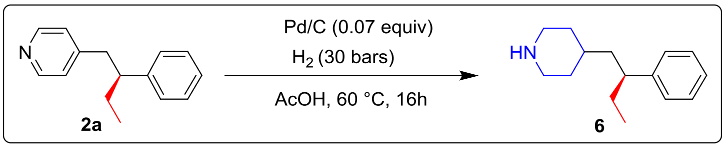


**Procedure:** A 4 mL glass vial with a magnetic stirring bar was charged with 5% Palladium on carbon (35 mg, approx. 0.016 mmol, 0.07 equiv), chiral pyridine derivative **2a** (50 mg, 0.23 mmol, 1 equiv) and AcOH (0.5 mL). The vial is placed in a 5mL high-pressure autoclave, closed, and evacuated with hydrogen 3-times. The autoclave was charged with 30 bars of hydrogen and kept stirring for 16 h at 80 °C. After cooling, hydrogen gas was carefully released, vial was removed from autoclave, and reaction mixture was transfer to a pad of celite and washed several times with EtOAc. Collected fractions were dried on rotary evaporator, dissolved in 1 mL of EtOAc and passed through a small pad of alumina in Pasteur pipette, flushed with EtOAc. Volatiles were removed on rotary evaporator to obtained almost clean product **6** as clear oil (49 mg, quantitative yield).

**$^1$H NMR** (400 MHz, $CDCl_3$) δ 7.32–7.24 (m, 2H), 7.21–7.15 (m, 1H), 7.14–7.10 (m, 2H), 3.04– 2.90 (m, 2H), 2.59–2.37 (m, 3H), 1.81–1.67 (m, 4H), 1.67–1.42 (m, 4H), 1.14–0.96 (m, 1H), 0.74 (t, $J$ = 7.4 Hz, 3H).

**$^{13}$C NMR** (101 MHz, $CDCl_3$) δ 145.9, 128.3, 127.8, 125.9, 46.8, 46.8, 44.4, 44.3, 34.6, 33.7, 33.4, 30.4, 12.3.

**HRMS (ESI$^+$):** *m/z* calcd. for $C_{15}H_{24}N$ ([M+H$^+$]) 218.19033, measured mass:218.19026

### (*R*)-2-(2-ethyloctyl)-5-(1H-tetrazol-5-yl)pyridine (7)

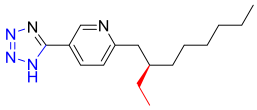

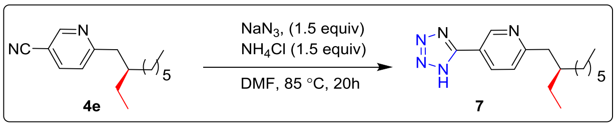


**Procedure**: A heat dried Schlenk tube equipped with glass stopper and magnetic stirring bar was charged under nitrogen with, chiral pyridine derivative **4e** (24.4 mg, 1 equiv), sodium azide (19.5 mg, 0.3 mmol, 3 equiv), ammonium chloride (16.1 mg, 0.3 mmol, 3 equiv) and suspended in 1.5 mL of dry DMF. The Schlenk was closed under nitrogen and kept stirring at 90 °C for 20 h. After cooling to room temperature was added 2N HCL (1 mL) and $H_2O$ (1 mL), then carefully neutralizes to pH 7 by adding NaOH solution and extracted with EtOAc (10 mL × 3). Combined organic phases were dried over $Na_2SO_4$, filtered and volatiles were removed on rotary evaporator. The oily crude was purified by flash-column chromatography, non-polar impurities were removed by flushing with mixture of pentane:EtOAc (80:20) and product was eluted with EtOAc. Volatiles were removed on rotary evaporator and product **7** was isolated as oil (23.8 mg, 82% yield).

**$^1$H NMR** (400 MHz, $CDCl_3$) δ 9.65 (s, 1H), 9.44 (s, 1H), 8.59 (d, $J$ = 8.0 Hz, 1H), 7.44 (d, $J$ = 8.1 Hz, 1H), 2.90 (d, $J$ = 7.3 Hz, 2H), 1.96–1.77 (m, 1H), 1.36–1.09 (m, 12H), 0.93–0.72 (m, 6H).

**$^{13}$C NMR** (101 MHz,$CDCl_3$) δ 163.2, 157.3, 145.7, 136.9, 125.2, 121.8, 41.8, 40.5, 32.9, 31.9, 29.7, 26.6, 25.7, 22.7, 14.2, 10.7.

**HRMS (ESI$^+$):** *m/z* calcd. for $C_{16}H_{26}N_5$ ([M+H$^+$]) 288.21827, found 288.21844

**ethyl (*R,E*)-3-(6-(2-ethyloctyl)pyridin-3-yl)acrylate (8)**

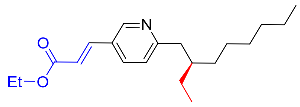


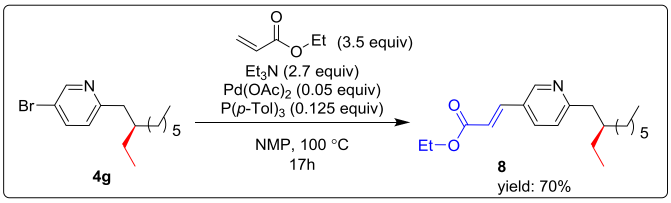


**Procedure**: A heat dried Schlenk tube equipped with rubber septum and magnetic stirring bar was charged under nitrogen with, $Pd(OAc)_2$ (2.8 mg, 0.0125 mmol, 0.05 equiv) and Tri(*p*-tolyl)phosphine (9.5 mg, 0.0315 mmol, 0.125 equiv), dissolved in N-methylpyrrolidinone (1mL) and added ethyl acrylate (345 mg/ 375 µL, 0.86 mmol, 3.5 equiv). The resulting reaction mixture was evacuated 3-times by vacuum-nitrogen cycle, and heated to 90 °C under nitrogen atmosphere for 15 minutes. To this mixture was added triethylamine (94 µL, 0.675 mmol, 2.7 equiv) and a chiral pyridine derivative **4g** (75 mg, 0.25 mmol, 1 equiv) in 1 mL n-methylpyrrolidinone dropwise. Rubber septum was quickly replaced with glass stopper and reaction kept stirring at 102 °C overnight (17h). After cooling, 1 mL water was added followed by 5 mL EtOAc and filtered through a pad of celite, washed several times with water and EtOAc. Organic phase was separated and aqueous phase was extracted twice with EtOAc (10 mL). Combined organic phases were dried over $MgSO_4$, filtered and volatiles were evaporated on rotary evaporator. The resulting oily crude was purified by flash-column chromatography ($SiO_2$, Pentane:EtOAc 96:4, v/v). The product **8** was isolated as pale-yellow oil (59 mg, 70% yield).

**$^1$H NMR** (400 MHz, $CDCl_3$) δ 8.63 (d, *J* = 2.2 Hz, 1H), 7.71 (dd, *J* = 8.1, 2.3 Hz, 1H), 7.64 (d, *J* = 16.1 Hz, 1H), 7.13 (d, *J* = 8.1 Hz, 1H), 6.44 (d, *J* = 16.0 Hz, 1H), 4.25 (q, *J* = 7.1 Hz, 2H), 2.71 (d, *J* = 7.2 Hz, 2H), 1.81 (hept, *J* = 6.0 Hz, 1H), 1.32 (t, *J* = 7.2 Hz, 3H), 1.31–1.15 (m, 12H), 0.90–0.74 (m, 6H).

**$^{13}$C NMR** (101 MHz, $CDCl_3$) δ 166.6, 164.2, 149.4, 141.2, 134.2, 127.5, 123.8, 119.3, 60.8, 42.8, 40.1, 33.0, 32.0, 29.7, 28.9, 26.6, 25.8, 14.4, 14.2, 10.8.

**HRMS (ESI$^+$):** *m/z* calcd. for $C_{20}H_{32}NO_2$ ([M+H$^+$]) 318.24330, found 318.24289

**(*R*)-5-bromo-2-(2-ethyloctyl)-4-isopropylpyridine (9)**

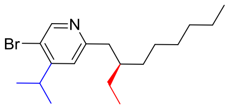


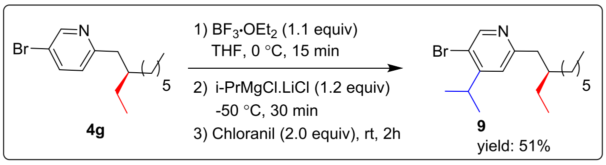


Product **9** was prepared by literature procedure.[9]

**Procedure**: In a flame dried Schlenk solution of compound **4g** (29.8 mg, 0.1 mmol, 1.0 equiv, in 0.2 ml of THF) is added and cooled to 0 °C followed by dropwise addition of $BF_3{\cdot}OEt_2$ (14 µl, 0.11 mmol, 1.1 equiv) and stirred for 30 minutes. The reaction mixture is cooled to -50 °C and *i*PrMgCl·LiCl (1.3M in THF, 100 µl, 0.13 mmol, 1.3 equiv) is added dropwise. The reaction mixture is allowed to stir at the

same temperature for 2 h. Chloranil (49.2 mg, 0.2 mmol, 2.0 equiv) was added next and the reaction was warmed to RT and stirred overnight. The reaction was quenched with 3M solution of NaOH (1 ml) and extracted with diethyl ether (5 mL × 3). Combined organic phases were dried on $MgSO_4$, filtered, and volatiles were removed on rotary evaporator to obtain oily crude, which was purified by flash-column chromatography (Pentane:EtOAc 99:1) to obtain heteroaryl product **9** as pale-yellow oil (17.3 mg, 51% yield)

**$^1$H NMR** (400 MHz, $CDCl_3$) δ 8.53 (s, 1H), 6.99 (s, 1H), 3.27 (hept, *J* = 6.9 Hz, 1H), 2.74–2.54 (m, 2H), 1.88–1.70 (m, 1H), 1.44–1.04 (m, 18H), 0.94–0.81 (m, 6H).
**$^{13}$C NMR** (101 MHz, $CDCl_3$) δ 161.2, 155.7, 151.2, 121.9, 119.9, 42.3, 40.0, 32.9, 32.6, 32.0, 29.8, 26.6, 25.9, 22.8, 22.1, 14.2, 10.9.
**HRMS (ESI$^+$):** *m/z* calcd. for $C_{18}H_{31}BrN$ ($[M+H^+]$) 340.16344, found 340.16361

**(*R*)-2-(2-ethyloctyl)-4-isobutoxy-5-(trifluoromethyl)pyridine (10)**

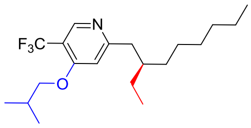


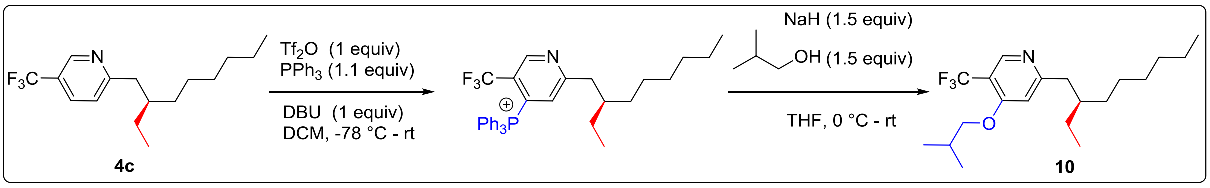


Product **10** was prepared in 2-steps by modifying literature procedure.[10]

<u>**Step-I**, Preparation of phosphonium salt</u>: A heat dried Schlenk tube equipped with rubber septum and magnetic stirring bar was charged under nitrogen with, chiral pyridine derivative **4c** (50 mg, 0.17 mmol, 1 equiv) in 1 mL $CH_2Cl_2$. The reaction mixture was cooled to -78 °C and $Tf_2O$ (49.1 mg/approx. 30 µL, 0.17 mmol, 1 equiv) was added dropwise. The reaction was stirred for 30 minutes and $PPh_3$ (49.1 mg, 0.187 mmol, 1.1 equiv) in 0.5 mL $CH_2Cl_2$ was added. The reaction was stirred for a further 30 minutes at -78 °C, and organic base 1,8-Diazabicyclo[5.4.0]undec-7-ene (DBU) (26.5 mg/approx 26 µL, 0.17 mmol, 1 equiv) was added. The resulting reaction mixture was warmed to RT and stirred for 30 min. The reaction mixture was quenched by adding 1 mL $H_2O$. The mixture was diluted with $CH_2Cl_2$ and $H_2O$, the resulting organic phase was removed and washed three times with $H_2O$. The organic layer was dried over $MgSO_4$, filtered and volatiles were removed on rotary evaporator. The resulting crude was analysed by NMR which showed 76 % conversion towards phosphonium salt. The crude was used in next step without further purification.

<u>**Step-II**, Preparation of heteroaryl ether</u>: A heat dried Schlenk tube equipped with rubber septum and magnetic stirring bar was charged under nitrogen with, sodium hydride (60% dispersion in mineral oil, 10.2 mg, 0.25 mmol, 1.5 equiv) and 1 mL THF. Cooled to 0 °C and 2-methylpropan-1-ol (23.5 µL, 0.25 mmol, 1.5 equiv) was added dropwise. The reaction was stirred for 30 minutes and the crude phosphonium salt from step-I (1.0 equiv) in 0.5 mL THF was added in one portion. The ice bath was removed and the reaction stirred for 15 hours while warming to room temperature. The reaction was quenched by adding 1 mL $H_2O$, diluted by $CH_2Cl_2$ and $H_2O$, the aqueous phase was separated and extracted with $CH_2Cl_2$ three times. The combined organic phases were washed with brine, dried over $MgSO_4$, filtered and volatiles were removed on rotary evaporator. The residue was purified by flash-column chromatography (Pentane:EtOAc 99:1) to obtain heteroaryl ether product **10** as pale-yellow oil (38.8 mg, 63% yield).

$^{1}$**H NMR** (400 MHz, $CDCl_3$) δ 8.55 (s, 1H), 6.67 (s, 1H), 3.84 (d, *J* = 6.2 Hz, 2H), 2.69 (d, *J* = 7.2 Hz, 2H), 2.23–2.06 (m, *J* = 6.6 Hz, 1H), 1.93–1.73 (m, 1H), 1.41–1.12 (m, 12H), 1.05 (d, *J* = 6.7 Hz, 6H), 0.94–0.77 (m, 6H).

$^{13}$**C NMR** (101 MHz, $CDCl_3$) δ 168.4 , 163.3 (h, *J* = 2.5, 1.3 Hz), 147.5 (q, *J* = 5.6 Hz), 123.6 (q, *J* = 272.2 Hz), 113.1 (q, *J* = 31.0 Hz), 107.2, 74.8, 43.4, 39.9, 32.9, 32.0, 29.8, 28.2, 26.6, 25.8, 22.8, 19.0, 14.2, 10.8.

**HRMS (ESI$^+$):** *m/z* calcd. for $C_{20}H_{33}F_3NO$ ([M+H$^+$]) 360.25088, found 360.25194

**CSP-HPLC:** (254 nm, Chiralcel OB-H, *n*-heptane:*i*PrOH = 99.8:0.2, 40 °C, 0. 5 ml/min.), $t_R$ = 19.61 min (major), $t_R$ = 20.72 min. (minor).

## Synthesis and characterization of Cu-complexes derived from ligand L3 and L9

### (*R*)-1-[(*S$_P$*)-2-(Dicyclohexylphosphino)ferrocenyl]-ethyl-di(3,5-xylyl)phosphine-Cu complex (Cu-L3)

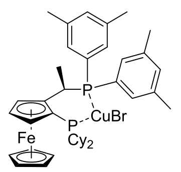


Copper complex **Cu-L3** was synthesized according to the literature procedure.[11]

$^{1}$**H NMR** ($CDCl_3$, 400 MHz):δ 7.33 (d, J = 9.2 Hz, 2H), 7.16 (d, J = 9.1 Hz, 2H), 6.97 (s, 1H), 6.89 (s, 1H), 4.33 (s, 1H), 4.29 (s, 1H), 4.21 (s, 1H), 4.02 (s, 5H), 3.57 (m, 1H), 2.57 (m, 1H), 2.29 (s, 6H), 2.19 (s, 6H), 2.03 – 0.87 (m, 25H).

$^{13}$**C NMR** ($CDCl_3$, 100.58 MHz): δ 138.1 (d, *J* = 9.3 Hz), 137.7 (d, *J* = 9.6 Hz), 132.5 (dd, *J* = 19.0, 8.2 Hz), 132.0 (d, *J* = 16.2 Hz), 131.8, 131.6 (d, *J* = 16.4 Hz), 131.6 , 130.1 (m), 128.7, 125.6, 93.6 (d, *J* = 24.4 Hz), 74.4 (d, *J* = 18.6 Hz), 73.4, 68.9, 39.4 (dd, *J* = 11.0, 5.7 Hz), 35.5 (m), 33.7 (d, *J* = 11.1 Hz), 31.8 (d, *J* = 10.9 Hz), 30.3 (dd, *J* = 14.3, 6.7 Hz), 29.8 , 28.1 (d, *J* = 16.5 Hz), 27.3 (d, *J* = 8.4 Hz), 26.8 (d, *J* = 12.3 Hz), 26.1 (d, *J* = 25.5 Hz), 24.3, 21.4 (d, *J* = 19.8 Hz), 18.6.

$^{31}$**P NMR** ($CDCl_3$, 161.94 MHz): δ 13.47.

**HRMS (ESI$^+$):** *m/z* calcd. for $C_{40}H_{52}BrCuFeP_2$ ([M+H$^+$]) 792.13676, found 792.13707

### (*R*)-1-[(*S$_P$*)-2-(Dicyclohexylphosphino)ferrocenyl]-ethyl-di[3,5-bis-(trifluoromethyl)phenyl] phosphine-Cu complex (Cu-L9)

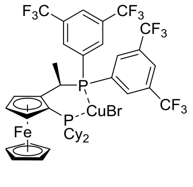


Copper complex **Cu-L9** was synthesized according to the literature procedure.[11] The analytical data were found to be in accordance with those reported in the literature.[11]

$^{1}$**H NMR** ($CDCl_3$, 400 MHz): δ 8.28 (s, 2*H*), 7.89 (s, 2*H*), 7.85 (s, 1*H*), 7.37 (s, 1H), 4.30 (s, 1*H*), 4.23 (s, 1*H*), 4.18 (s, 5*H*), 4.12 (s, 1*H*), 3.86 (q, 1*H*), 1.0–2.0 (m, 25*H*).

$^{31}$**P NMR** ($CDCl_3$, 161.94 MHz): δ 14.31 (br. d, J = 155.3 Hz), −9.53 (br. d, J = 149.9 Hz).

$^{19}$**F NMR** ($CDCl_3$, 376.29 MHz): δ -63.1.

## Mechanistic studies

### *NMR spectroscopy (supplementary figures 98, 99)*

Confirmation of the reactivity of pyridine substrates and the effect of LAs was obtained from 1H NMR spectroscopic studies. We investigated the interaction between various reagents (LAs and EtMgBr) and alkenyl-pyridine substrates **1a** and **3b**. The corresponding samples were prepared in $CD_2Cl_2$ at -78 °C and the spectra were recorded at -60 °C (Supplementary Figures 98 and 99). In particular we followed the downfield shifts of olefinic protons (indicated on the spectra as **c** and **d** for **1a**, and **d** and **e** for **3b**) which are indicative of a reduction of the electronic density around those protons.

**Pyridine-Lewis acid and pyridine-Grignard complexes:**

In order to determine the activation mode of pyridines (**1a** and **3b**) in the presence of different Lewis acids, a set of experiments was carried out. Complexes of **1a** with TMSOTf, TMSCl, $BF_3{\cdot}Et_2O$, and EtMgBr, and **3b** with $BF_3{\cdot}Et_2O$ and EtMgBr were prepared separately by following the general procedure below and analyzed by $^1$H NMR spectroscopy.

**General procedure for preparation of pyridine-LA and pyridine-EtMgBr complexes.**

In a heat dried Schlenk tube equipped with septum and magnetic stirring bar, pyridine substrate **1a** or **3b** (0.1 mmol, 1 equiv) was dissolved in $CD_2Cl_2$ (1 mL) under $N_2$ atmosphere at room temperature, the reaction mixture was then cooled to -78 °C and Lewis acid or EtMgBr (0.15-0.2 mmol, 1.5-2.0 equiv) was added to this solution. After stirring for 10 minutes, the resulting reaction mixture was rapidly transferred by syringe in a dry NMR tube under $N_2$ atmosphere at -78 °C. A new species formed was immediately checked by $^1$H NMR spectroscopy at -60 °C.

**1a-TMSOTf complex** was prepared by following the general procedure above, with **1a** (18.1 mg, 0.1 mmol) in $CD_2Cl_2$ (1 mL) and TMSOTf (36 μL, 0.2 mmol). After stirring for 10 minutes the resulting turbid reaction mixture was immediately measured by $^1$H NMR spectroscopy at -60 °C.
**1a-TMSCl complex** was prepared by following the general procedure above, with **1a** (18.1 mg, 0.1 mmol) in $CD_2Cl_2$ (1 mL) and TMSCl (26 μL, 0.2 mmol). After stirring for 10 minutes the resulting reaction mixture was immediately measured by $^1$H NMR spectroscopy at -60 °C.
**1a-$BF_3{\cdot}OEt_2$ complex** was prepared by following the general procedure above, with **1a** (18.1 mg, 0.1 mmol) in $CD_2Cl_2$ (1 mL) and $BF_3{\cdot}OEt_2$ (25 μL, 0.2 mmol). After stirring for 10 minutes the resulting reaction mixture was immediately measured by $^1$H NMR spectroscopy at -60 °C.
**1a-EtMgBr complex** was prepared by following the general procedure above, with **1a** (18.1 mg, 0.1 mmol) in $CD_2Cl_2$ (1 mL) and EtMgBr (60 μL, 0.2 mmol). After stirring for 10 minutes the resulting reaction mixture was immediately measured by $^1$H NMR spectroscopy at -60 °C.
**3b-$BF_3{\cdot}OEt_2$ complex** was prepared by following the general procedure above, with **3b** (24.9 mg, 0.1 mmol) in $CD_2Cl_2$ (1 mL) and $BF_3{\cdot}OEt_2$ (19 μL, 0.15 mmol). After stirring for 10 minutes the resulting reaction mixture was immediately measured by $^1$H NMR spectroscopy at -60 °C.
**3b-EtMgBr complex** was prepared by following the general procedure above, with **3b** (24.9 mg, 0.1 mmol) in $CD_2Cl_2$ (1 mL) and EtMgBr (60 μL, 0.2 mmol). After stirring for 10 minutes the resulting reaction mixture was immediately measured by $^1$H NMR spectroscopy at -60 °C.

### *Nature of enolates formed at the end of ACA reaction (Supplementary figures 100-104)*

The structure of the final product in the reaction mixture before its quench, namely the structure of the product enolate, was determined by NMR spectroscopic analysis in $CD_2Cl_2$.

In a flame-dried Schlenk tube equipped with septum and magnetic stirring bar, $CuBr·SMe_2$ (0.10 equiv), and ($R$,$S_p$)-**L1** (0.12 equiv) were dissolved in $CD_2Cl_2$ (1 mL/0.1mmol of substrate) and stirred under nitrogen atmosphere for 15 min. The substrate (1.0 equiv) was added at once. After stirring for 5 min. at RT the reaction mixture was cooled to -78 °C and TMSOTf (3.0 equiv) was added followed by EtMgBr (3.0 equiv). After stirring at -78 °C for 15h, , the mixture was transferred to a NMR tube followed by measurement at -60 °C. The final product in the reaction mixture was identified as a TMS-enolate based on a $^1$H NMR, 2D NOESY and 1D NOESY experiments.

**$^1$H NMR** (500 MHz, $CD_2Cl_2$) δ 7.33–7.05 (m, 5H), 6.17 (d, $J$ = 7.9 Hz, 1H), 6.04 (d, $J$ = 7.7 Hz, 1H), 5.57 (dd, $J$ = 8.0, 2.3 Hz, 1H), 5.47 (dd, $J$ = 7.9, 2.3 Hz, 1H), 4.35 (d, $J$ = 8.8 Hz, 1H), 3.13 (q, $J$ = 8.0 Hz, 1H), 1.57 (m, 2H), 0.88 (t, $J$ = 8.0 Hz, 3H), 0.20 (s, 9H).$^1$H-, 1D-NOESY (respectively **Supplementary Fig. 100** and **103**) and 2d-NOESY (**Supplementary Fig. 101** and expansion in **Supplementary Fig. 102**) have been used to determine the structure of TMS-Enolate of **2a**

## *Double bond (E)/(Z) isomerization experiments (Supplementary table 2)*

The influence of the alkene (($Z$)/($E$)) geometry on the enantioselective CA of EtMgBr to **1a** and **3b** was examined. When subjecting **(*Z*)-1a** to the optimised protocol the product was obtained with a markedly lower 42% ee, and with an absolute configuration opposite to that observed with **(*E*)-1a** (Supplementary table 2, compare entries 1 and 2). We also noticed that the reactivity of **(*Z*)-1a** is rather low compared to **(*E*)-1a**: the catalytic reaction was slower than with **(*E*)-1a** and no background reaction was observed in the absence of Cu-catalyst; partial ($Z$)-($E$) isomerization of the substrate **1a** did occur, however (Supplementary table 2, entry 3). The reduction in ee in the catalytic reaction could also be related to the partial isomerization of the substrate double bond during the reaction.
To test this hypothesis we quenched the reaction before reaching full conversion and analysed both the remaining alkene substrate **1a**, as well as the ee of the addition product (Supplementary table 2, entry 4). The CA product was obtained with similar 42% ee, indicating that the decreased enantioselectivity is intrinsic to the ($Z$)-geometry of the substrate and not due to (Z)-(E) isomerization. However, the remaining substrate was partially isomerised to the more stable ($E$)-isomer. To understand what causes the isomerization we carried out additional experiments that indicated that as long as TMSOTf is present in the media (whether alone, in combination with EtMgBr, or with Cu-catalyst and MeMgBr which is too unreactive for CA addition) significant isomerization of the substrate **1a** occurs within 15h (Supplementary table 2, entries 6, 3 and 7). Interestingly, the isomerization level is the highest with TMSOTf alone, which perhaps relates to the fact that in the absence of any CA, TMSOTf reacts effectively with the Grignard reagent, reducing the amount of TMSOTf present to affect the isomerization. Similar experiments were conducted with substrate **3b**, which requires $BF_3·OEt_2$ as a LA for CA (Supplementary table 2, entries 8-13). In this case, both the **(*Z*)-3b** and **(*E*)-3b** substrates provided the CA product with only small differences in the ee values and with opposite configurations (Supplementary table 2, entries 8 and 9). Additional control experiments (entries 10-13) proved that there is nearly no (Z)/(E) isomerization of the substrate in the presence of various reaction components, thus confirming that the lower enantioselectivity in this case is also an intrinsic feature of this substrate with this catalytic methodology.

### Preparation of (Z)-1a by photoisomerization

A solution **(*E*)-1a** in 5 screw cap glass vials (each vial contain 25 mg in 2.5 mL $CH_2Cl_2$) was irradiated with 365-nm light [Spectroline model ENC-280C/FE lamp] for 5h (NMR monitoring). The solvent was

evaporated in vacuo to provide a mixture of **(*Z*)-1a** and **(*E*)-1a** in 76:14 ratio. After flash column chromatography **(*Z*)-1a** was isolated with 99.6% purity remaining 0.4% was **(*E*)-1a** ($SiO_2$, Pentane:EtOAc 80:20 v/v).

**$^1$H NMR** ($CDCl_3$, 400 MHz): δ 8.45 (d, *J* = 5.0 Hz, 2H), 7.27–7.23 (m, 3H), 7.23–7.18 (m, 2H), 7.11 (d, *J* = 6.1 Hz, 2H), 6.80 (d, *J* = 12.2 Hz, 1H), 6.50 (d, *J* = 12.2 Hz, 1H).

**$^{13}$C NMR** ($CDCl_3$, 100.58 MHz): δ 149.9, 145.2, 136.3, 134.2, 128.9, 128.6, 128.0, 127.7, 123.7.

### *Preparation of (Z)-3b by photoisomerization*

A solution **(*E*)-3b** in 5 screw cap glass vials (each vial contain 25 mg in 2.5 mL $CH_2Cl_2$) was irradiated with 365-nm light [Spectroline model ENC-280C/FE lamp] for 24h (NMR monitoring). The solvent was evaporated in vacuo to provide a mixture of **(*Z*)-3b** and **(*E*)-3b** in 64:36 ratio. After flash column chromatography **(*Z*)-3b** was isolated was isolated with 99.0% purity remaining 1.0% was ($SiO_2$, Pentane:EtOAc 98:2 v/v).

**$^1$H NMR** ($CDCl_3$, 400 MHz): δ 8.84 (s, 1H), 7.65 (dd, *J* = 8.4, 2.4 Hz, 1H), 7.35–7.16 (m, 6H), 6.97 (d, *J* = 12.4 Hz, 1H), 6.71 (d, *J* = 12.5 Hz, 1H).

**$^{13}$C NMR** ($CDCl_3$, 100.58 MHz): δ 159.97, 159.96, 146.6 (q, *J* = 4.2 Hz), 136.2, 135.7, 132.8 (q, *J* = 3.5 Hz), 129.4, 128.9, 128.9, 128.6, 128.2, 124.5 (q, *J* = 33.0 Hz), 124.3 (q, *J* = 271.5 Hz), 123. 6.

**$^{19}$F NMR** (376 MHz, $CDCl_3$) δ -62.6

### *Catalytic asymmetric addition of EtMgBr to (Z)-1a*

The reaction was performed using general procedure A, with 0.1 mmol **(Z)-1a**, TMSOTf (0.3 mmol, 3.0 equiv), EtMgBr (3M in $Et_2O$, 0.3 mmol, 3.0 equiv), CuBr·$SMe_2$ (0.01 mmol, 10 mol%), ligand (R,$S_p$)-**L1** (0.012 mmol, 12 mol%) in 1 mL $CH_2Cl_2$. Product **(R)-2a** was obtained as pale-yellow oil after flash-column chromatography ($SiO_2$, Pentane:EtOAc 90:10 v/v), [72% yield, 42% ee].

### *(E)/(Z) isomerization experiments of (Z)-1a with TMSOTf*

**(*Z*)-1a** (9.06mg, 0.05 mmol) was placed in a heat dried Schlenk tube equipped with rubber septum and magnetic stirring bar. After addition of $CH_2Cl_2$ (0.5mL) the mixture was cooled to -78 °C and TMSOTf (33.35 mg/ 27.15 µL, 0.15 mmol, 3 equiv) was added. After stirring for 15h at -78 °C the reaction mixture was quenched by adding MeOH (1 mL) followed by addition of saturated aqueous $NH_4Cl$ solution and warmed to RT. The resulting mixture was extracted with $CH_2Cl_2$ (2 × 10mL). Combined organic phases were dried over $MgSO_4$, filtered and solvents were evaporated. The (*E*)/(*Z*) ratio of **1a** in the resulting crude mixture was determined by $^1$H-NMR and was found to be 24:76.

### *(E)/(Z) isomerization experiments of (Z)-1a with EtMgBr*

**(*Z*)-1a** (9.06 mg, 0.05 mmol) was placed in a heat dried Schlenk tube equipped with rubber septum and magnetic stirring bar. After addition of $CH_2Cl_2$ (0.5 mL) the mixture was cooled to -78 °C and EtMgBr (3M in $Et_2O$, 50 µL, 0.15 mmol, 3 equiv) was added. After stirring for 15h at -78 °C the reaction mixture was quenched by adding MeOH (1 mL) followed by addition of saturated aqueous $NH_4Cl$ solution and warmed to RT. The resulting mixture was extracted with $CH_2Cl_2$ (2 × 10 mL). Combined organic phases were dried over $MgSO_4$, filtered and solvents were evaporated. The (*E*)/(*Z*) ratio of **1a** in the resulting crude mixture was determined by $^1$H-NMR and was found to be 02:98.

#### *(E)/(Z) isomerization experiments of (Z)-1a with EtMgBr and TMSOTf*

**(*Z*)-1a** (9.06 mg, 0.05 mmol) was placed in a heat dried Schlenk tube equipped with rubber septum and magnetic stirring bar. After addition of $CH_2Cl_2$ (0.5 mL) the mixture was cooled to -78 °C and TMSOTf (33.35 mg/ 27.15 µL, 0.15 mmol, 3 equiv) and EtMgBr (3M in $Et_2O$, 50 µL, 0.15 mmol, 3 equiv) were added. After stirring for 15h at -78 °C the reaction mixture was quenched by adding

MeOH (1 mL) followed by addition of saturated aqueous $NH_4Cl$ solution and warmed to RT. The resulting mixture was extracted with $CH_2Cl_2$ (2 × 10mL). Combined organic phases were dried over $MgSO_4$, filtered and solvents were evaporated. The (*E*)/(*Z*) ratio of **1a** in the resulting crude mixture was determined by $^1$H-NMR and was found to be 18:82.

**(*E*)/(*Z*) isomerization experiments of (*Z*)-1a with Cu·SMe$_2$ and (*R, S$_p$*)-L1**

In a heat dried Schlenk tube equipped with septum and magnetic stirring bar, Cu·SMe$_2$ (1.025 mg, 0.005 mmol, 0.1 equiv) and (*R, S$_p$*)-L1 (3.57 mg, 0.006 mmol, 0.12 equiv) were dissolved in $CH_2Cl_2$ (0.5 mL) and stirred under nitrogen atmosphere for 15 min. Substrate (1.0 equiv) ***(Z)*-1a** (9.06 mg, 0.05 mmol) was added at once. After stirring for 5 min. at RT the reaction mixture was cooled to -78 °C. After stirring at -78 °C for 16h, the reaction was quenched with MeOH (1 mL) followed by saturated aqueous solution of $NH_4Cl$ and warmed to RT. The resulting mixture was extracted with $CH_2Cl_2$ (2 × 10 mL). Combined organic phases were dried over $MgSO_4$, filtered and solvents were evaporated. The (*E*)/(*Z*) ratio of **1a** in the resulting crude mixture was determined by $^1$H-NMR and was found to be 02:98.

***(E)/(Z) isomerization experiments of (Z)-1a with CuBr·SMe$_2$, (R, S$_p$)-L1, TMSOTf, and MeMgBr***

In a heat dried Schlenk tube equipped with septum and magnetic stirring bar, Cu·SMe$_2$ (2.05 mg, 0.01 mmol, 0.1 equiv) and (*R, S$_p$*)-L1 (7.14 mg, 0.012 mmol, 0.12 equiv) were dissolved in $CH_2Cl_2$ (1 mL) and stirred under nitrogen atmosphere for 15 min. Substrate (1.0 equiv) ***(Z)*-1a** (18 mg, 0.1 mmol, 1 equiv) was added at once. After stirring for 5 min. at RT the reaction mixture was cooled to -78 °C. and TMSOTf (66.7 mg/ 54 µL, 0.3 mmol, 3 equiv) was added followed by MeMgBr (2M in $Et_2O$, 100 µL, 0.3 mmol, 3 equiv). After stirring at -78 °C for 16h, the reaction was quenched with MeOH (1 mL) followed by saturated aqueous solution of $NH_4Cl$ and warmed to RT. The resulting mixture was extracted with $CH_2Cl_2$ (2 × 10mL). Combined organic phases were dried over $MgSO_4$, filtered and solvents were evaporated. The (*E*)/(*Z*) ratio of **1a** in the resulting crude mixture was determined by $^1$H-NMR and was found to be 14:86.

## *Catalytic asymmetric addition of EtMgBr to (Z)-3b*

The reaction was performed using general procedure B, with 0.05 mmol **(*Z*)-3b**, $BF_3$·$OEt_2$ (0.075 mmol, 1.5 equiv), EtMgBr (3M in $Et_2O$, 0.1 mmol, 2.0 equiv), CuBr·SMe$_2$ (0.005 mmol, 10 mol%), ligand (*R,S$_p$*)-**L1** (0.006 mmol, 12 mol%) in 0.5 mL $Et_2O$. After 15h at -78 °C reaction reached 34% conversion and product **(*R*)-4b** was not isolated and directly injected in HPLC to determine the ee of the reaction that was found to be 76%.

## *(E)/(Z) isomerization experiments of (Z)-1a with BF$_3$·OEt$_2$*

**(*Z*)-3b** (12.45mg, 0.05 mmol) was placed in a heat dried Schlenk tube equipped with rubber septum and magnetic stirring bar. After addition of $Et_2O$ (0.5mL) the mixture was cooled to -78 °C and $BF_3$·$OEt_2$ (10.6 mg/ 10 µL, 0.075 mmol, 1.5 equiv) was added. After stirring for 15h at -78 °C the reaction mixture was quenched by adding MeOH (1 mL) followed by addition of saturated aqueous $NH_4Cl$ solution and warmed to RT. The resulting mixture was extracted with $CH_2Cl_2$ (2 × 10mL). Combined organic phases were dried over $MgSO_4$, filtered and solvents were evaporated. The (*E*)/(*Z*) ratio of **3b** in the resulting crude mixture was determined by $^1$H-NMR and was found to be 01:99.

## *(E)/(Z) isomerization experiments of (Z)-3b with EtMgBr*

**(*Z*)-3b** (12.45mg, 0.05 mmol) was placed in a heat dried Schlenk tube equipped with rubber septum and magnetic stirring bar. After addition of $Et_2O$ (0.5mL) the mixture was cooled to -78 °C and EtMgBr (3M in $Et_2O$, 30 µL, 0.1 mmol, 2.0 equiv) was added. After stirring for 15h at -78 °C the reaction mixture was quenched by adding MeOH (1 mL) followed by addition of saturated aqueous $NH_4Cl$ solution and warmed to RT. The resulting mixture was extracted with $CH_2Cl_2$ (2 × 10mL). Combined organic phases were dried over $MgSO_4$, filtered and solvents were evaporated. The (*E*)/(*Z*) ratio of **3b** in the resulting crude mixture was determined by $^1$H-NMR and was found to be 01:99.

***(E)/(Z) isomerization experiments of (Z)-3b with EtMgBr and $BF_3$·$OEt_2$***

**(*Z*)-3b** (12.45 mg, 0.05 mmol) was placed in a heat dried Schlenk tube equipped with rubber septum and magnetic stirring bar. After addition of $Et_2O$ (0.5 mL) the mixture was cooled to -78 °C and $BF_3$·$OEt_2$ (10.6 mg/ 10 µL, 0.075 mmol, 1.5 equiv) and EtMgBr (3M in $Et_2O$, 30 µL, 0.1 mmol, 2.0 equiv) were added. After stirring for 15h at -78 °C the reaction mixture was quenched by adding MeOH (1 mL) followed by addition of saturated aqueous $NH_4Cl$ solution and warmed to RT. The resulting mixture was extracted with $CH_2Cl_2$ (2 × 10 mL). Combined organic phases were dried over $MgSO_4$, filtered and solvents were evaporated. The (*E*)/(*Z*) ratio of **3b** in the resulting crude mixture was determined by $^1$H-NMR and was found to be 04:96.

***(E)/(Z) isomerization experiments of (Z)-3b with Cu·$SMe_2$ and (R, $S_p$)-L1***

In a heat dried Schlenk tube equipped with septum and magnetic stirring bar, Cu·$SMe_2$ (1.025 mg, 0.005 mmol, 0.1 equiv) and (*R, $S_p$*)-**L1** (3.57 mg, 0.006 mmol, 0.12 equiv) were dissolved in $Et_2O$ (0.5 mL) and stirred under nitrogen atmosphere for 15 min. Substrate (1.0 equiv) ***(Z)*-3b** (12.45 mg, 0.05 mmol) was added at once. After stirring for 5 min. at RT the reaction mixture was cooled to -78 °C. After stirring at -78 °C for 16h, the reaction was quenched with MeOH (1 mL) followed by saturated aqueous solution of $NH_4Cl$ and warmed to RT. The resulting mixture was extracted with $CH_2Cl_2$ (2 × 10mL). Combined organic phases were dried over $MgSO_4$, filtered and solvents were evaporated. The (*E*)/(*Z*) ratio of **3b** in the resulting crude mixture was determined by $^1$H-NMR and was found to be 05:95.

***(E)/(Z) isomerization experiments of (Z)-1a with Cu·$SMe_2$, (R, $S_p$)-L1, MeMgBr and $BF_3$·$OEt_2$***

In a heat dried Schlenk tube equipped with septum and magnetic stirring bar, Cu·$SMe_2$ (1.025 mg, 0.005 mmol, 0.1 equiv) and (*R, $S_p$*)-**L1** (3.57 mg, 0.006 mmol, 0.12 equiv) were dissolved in $Et_2O$ (0.5 mL) and stirred under nitrogen atmosphere for 15 min. Substrate (1.0 equiv) ***(Z)*-3b** (12.45 mg, 0.05 mmol) was added at once. After stirring for 5 min. at RT the reaction mixture was cooled to -78 °C. After stirring for 5 min. at RT the reaction mixture was cooled to -78 °C. and $BF_3$·$OEt_2$ (10.6 mg/ 10 µL, 0.075 mmol, 1.5 equiv) was added followed by MeMgBr (3M in $Et_2O$, 30 µL, 0.1 mmol, 2 equiv). After stirring at -78 °C for 16h, the reaction was quenched with MeOH (1 mL) followed by saturated aqueous solution of $NH_4Cl$ and warmed to RT. The resulting mixture was extracted with $CH_2Cl_2$ (2 × 10 mL). Combined organic phases were dried over $MgSO_4$, filtered and solvents were evaporated. The (*E*)/(*Z*) ratio of **3b** in the resulting crude mixture was determined by $^1$H-NMR and was found to be 05:95.

## Supplementary Tables

### Supplementary Table 1. Ligand screening in the optimization of reaction conditions[#]

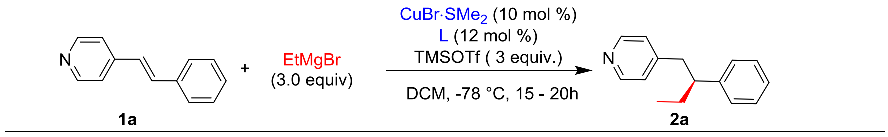


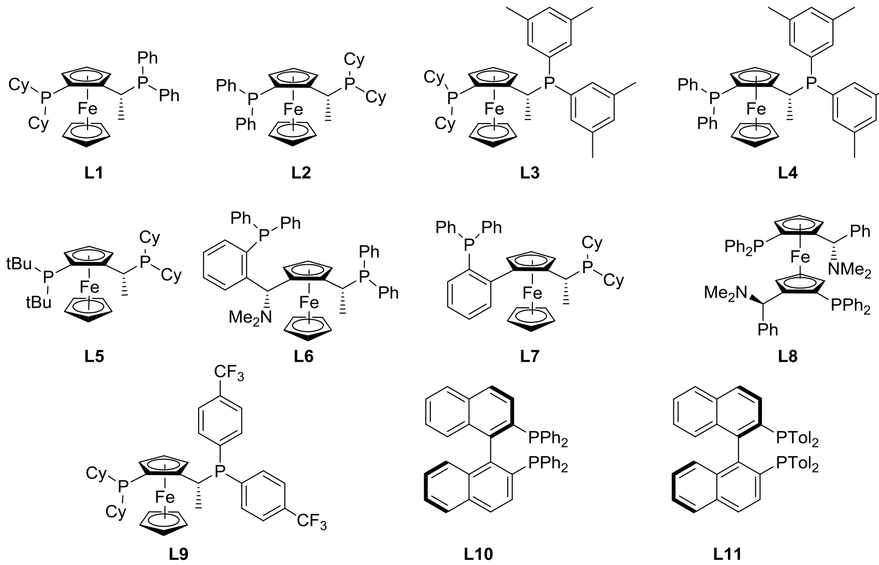


| Entry | L | Conv.(%) | Yield (%)* | *ee* (%)± |
|---|---|---|---|---|
| 1 | L1 | 100 | 94 | 93 |
| 2 | L2 | 76 | 61 | 45 |
| 3 | L3 | 93 | 80 | 81 |
| 4 | L4 | 95 | 92 | 90 |
| 5 | L5 | 75 | 38 | 0 |
| 6 | L6 | ND | 20 | 87 |
| 7 | L7 | 67 | 46 | 0 |
| 8 | L8 | 48 | 26 | 0 |
| 9 | L9 | 81 | 70 | 39 |
| 10 | L10 | 62 | 32 | 10 |
| 11 | L11 | 80 | 58 | 20 |

[#]Unless otherwise specified, a mixture of **1a** (0.1 mmol) in the presence of the catalyst (10 mol%), Lewis acid (3.0 equiv) and Grignard reagent (3.0 equiv) in the indicated solvent (1.0 mL) was stirred at -78 °C for 15 - 20 h. *Isolated yield ±Determined by Chiral-HPLC;

### Supplementary Table 2. CA and (*Z*)/(*E*) isomerization of substrates 1a and 3b*

| Entry | Alkene | LA | Cu/**L1** (%) | RMgBr | Conv. (%)± | (*Z*):(*E*)(%)‡ | *ee* (%)# |
|---|---|---|---|---|---|---|---|
| 1[ˀ] | (*E*)-**1a** | TMSOTf | 10 | EtMgBr | 100 | - | 93 (*S*) |
| 2 | (*Z*)-**1a** | TMSOTf | 10 | EtMgBr | 100 | - | 42 (*R*) |
| 3 | (*Z*)-**1a** | TMSOTf | - | EtMgBr | 0 | 82 : 18 | - |
| 4[¤] | (*Z*)-**1a** | TMSOTf | 10 | EtMgBr | 75 | 5 : 1 | 42 (*R*) |
| 5 | (*Z*)-**1a** | - | - | EtMgBr | 0 | 98 : 02 | - |

| | | | | | | | |
|---|---|---|---|---|---|---|---|
| 6 | (*Z*)-**1a** | TMSOTf | - | - | - | 76 : 24 | - |
| 7 | (*Z*)-**1a** | TMSOTf | 10 | MeMgBr | 0 | 86 : 14 | - |
| 8 | (*E*)-**3b** | $BF_3{\cdot}OEt_2$ | 5 | EtMgBr | 100 | - | 86 (*S*) |
| 9 | (*Z*)-**3b** | $BF_3{\cdot}OEt_2$ | 5 | EtMgBr | 34 | 99 : 02 | 76 (*R*) |
| 10 | (*Z*)-**3b** | $BF_3{\cdot}OEt_2$ | - | EtMgBr | 0 | 94 : 06 | - |
| 11 | (*Z*)-**3b** | $BF_3{\cdot}OEt_2$ | - | - | - | 99 : 01 | - |
| 12 | (*Z*)-**3b** | | - | EtMgBr | 0 | 99 : 01 | - |
| 13 | (*Z*)-**3b** | $BF_3{\cdot}Et_2O$ | 10 | MeMgBr | 0 | 95 : 05 | - |

All the isomerization studies were carried out at -78 °C in DCM for 15h unless mentioned otherwise. [*](*Z*)-**1a** and (*Z*)-**3b** were prepared by photoisomerization of (*E*)-**1a** and (*E*)-**3b**, respectively (for details see SI). [±] Conversions were determined by $^{1}$H NMR, [‡]Ratio (*Z*) : (*E*) was determined by $^{1}$H NMR, [#]Determined by chiral HPLC, [ˀ]Similar results were obtained quenching the reaction after 30 minutes, [¤]Reaction quenched after 1h.

## Supplementary Notes

## Supplementary Note 1

Superstoichiometric amounts of Grignard reagents (3 equiv.) and Lewis acid (3 equiv.) were selected as optimal conditions for TMSOTf promoted reactions. However excess amounts are only required for the most unreactive substrates. When the reactivity of the substrate is increasing (for instance going from aromatic to aliphatic 4-substitued alkenyl pyridines) the amounts can also be reduced to 1.2-1.5 equiv. The reason behind this is the following: there is a competing reaction between Grignard reagents and LA (at -78 $^{o}$C, rather fast) leading to the formation of silane with TMSOTf and borate with $BF_3$. As a consequence of this side reaction the effective concentration of these reagents is decreasing. This side reaction is more pronounced when the substrate is unreactive, since CA addition is slower with non-activated substrates. Because TMSOTf is the most efficient LA for unactivated substrates, an increased amount of it, together with the Grignard reagent, is required. Furthermore, we believe that both reagents are involved in the rate limiting step and as a result the increase in their concentration will accelerate the CA.

**Supplementary Note 2** **2a'** was prepared by bubbling HCl gas to the solution of **2a** in diethyl ether. Obtained precipitate was isolated by evaporating diethyl ether. Recrystallization was carried out in EtOAc and heptane

## Supplementary References


[1] Sheldrick, G. SHELXT - Integrated space-group and crystal-structure determination. *Acta. Crystallographica. Section A* **71**, 3-8 (2015).

[2] Sheldrick, G. A. short history of SHELX. *Acta. Crystallographica. Section A* **64**, 112-122 (2008).

[3] Hoye, T. R., Eklov, B. M., Voloshin, M. No-D NMR Spectroscopy as a Convenient Method for Titering Organolithium (RLi), RMgX, and LDA Solutions. *Org. Lett.* **6**, 2567-2570 (2004).

[4] Jumde, R. P., Lanza, F., Veenstra, M. J., Harutyunyan, S. R. Catalytic asymmetric addition of Grignard reagents to alkenyl-substituted aromatic N-heterocycles. *Science* **352**, 433-437 (2016).

[5] Zhang, Z., Wang, Z. Diatomite-supported Pd nanoparticles: an efficient catalyst for Heck and Suzuki reactions. *J. Org. Chem.* **71**, 7485-7487 (2006).

[6] Lucenti, E., Cariati, E., Dragonetti, C., Manassero, L., Tessore, F. Effect of the Coordination to the "$Os_3(CO)_{11}$" Cluster Core on the Quadratic Hyperpolarizability of trans-4-(4'-X-styryl)pyridines (X = $NMe_2$, t-Bu, $CF_3$) and trans,trans-4-(4'-NMe2-phenyl-1,3-butadienyl)pyridine. *Organometallics* **23**, 687-692 (2004).

[7] I. N. Houpis, J. Lee, I. Dorziotis, A. Molina, B. Reamer, R. P. Volante, P. J. Reider, Ni catalyzed nucleophilic conjugate additions of Grignard and organozincate reagents to substituted 4-vinylpyridines. General synthesis of phosphodiesterase IV inhibitors. *Tetrahedron* **54**, 1185–1195 (1998).

[8] *trans-1-(2-Pyridyl)-2-(4-pyridyl)-ethylene, 97%.* Sigma-Aldrich, http://www.sigmaaldrich.com/catalog/product/aldrich/197459?lang (2017)

[9] Chen, Q., Mollat du Jourdin, X., Knochel, P. Transition-metal-free $BF_3$-mediated regioselective direct alkylation and arylation of functionalized pyridines using Grignard or organozinc reagents. *J. Am. Chem. Soc.* **135**, 4958-4961 (2013).

[10] Hilton, M. C., Dolewski, R. D., McNally, A. Selective Functionalization of Pyridines via Heterocyclic Phosphonium Salts. *J Am Chem Soc* **138**, 13806-13809 (2016).

[11] Bruker, (2012). *APEX2* (v2012.4-3), *SAINT* (Version 8.18C) and *SADABS* (Version 2012/1). Bruker AXS Inc., Madison, Wisconsin, USA
