# Supplementary material for: Highly enantioselective catalytic synthesis of chiral pyridines
Source: Nat Commun. 2017 Dec 12;8:2058. doi: 10.1038/s41467-017-01966-7 (PMC5727103; doi:10.1038/s41467-017-01966-7)
Supplement: Supplementary file 2 — Description of Additional Supplementary Files [file 41467_2017_1966_MOESM2_ESM.pdf]

Description of Additional Supplementary Files

File Name: Supplementary Data 1
Description: Crystallographic information file for X-ray
